# A Polarity-Mismatched Photocatalytic Cross-Coupling Enables Diversity-Oriented Synthesis of aza-Heterocycles

**DOI:** 10.1021/acs.orglett.5c04971

**Published:** 2026-02-03

**Authors:** Joanna Urbańczyk, Aidan P. McKay, David B. Cordes, Tomas Lebl, Miles H. Aukland, Allan J. B. Watson

**Affiliations:** † EaStCHEM, School of Chemistry, 7486 University of St Andrews, St Andrews, Fife KY16 9ST, United Kingdom; ‡ Chemical Development, Pharmaceutical Technology & Development, Operations, AstraZeneca, Macclesfield SK10 2NA, United Kingdom

## Abstract

Diversity-oriented synthesis (DOS) is an attractive approach for the design of functional molecules with (homo)­allylic amines representing a particularly attractive DOS platform. Herein, we demonstrate the application of newly developed photocatalytic cross-nucleophilic coupling to provide rapid access to (homo)­allylic amines, which can be smoothly converted to a range of heterocyclic scaffolds. Employing this approach, a variety of aza-heterocycles were accessed, including α-haloaziridines, pyrrolidines, and oxazinan-2-ones, with structural diversity examined by using uniform manifold approximation and projection (UMAP).

## Graphical abstract

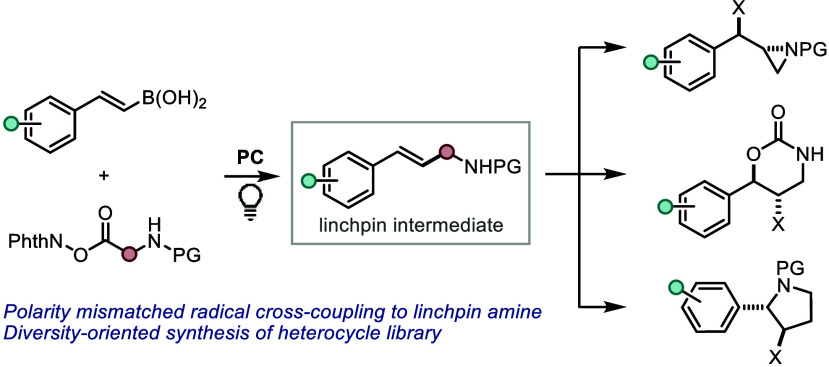

Diversity-oriented synthesis (DOS) is an attractive approach to populate chemical space and, by extension, engage a broader array of biological targets.^1^ Fundamentally, DOS converts a single or small group of similar compounds into a larger library, exhibiting diversity in structural connectivity and topology.

DOS has been extensively employed within the context of synthetic chemistry,^2^ chiefly to accelerate drug discovery.^3^ Contemporary approaches have connected DOS with cheminformatics,^4^ using algorithms to map chemical space of existing libraries and during a priori design phases.

The two main strategies for DOS are (i) reagent-based and (ii) substrate-based approaches (also termed folding).^5^ The former generates diversity through the application of different reagents or conditions to a single substrate, while the latter uses differences in substrate structures to afford a range of product scaffolds under common reaction conditions.

*Trans*-cinnamylamines, despite their relative simplicity, offer a potentially powerful starting point for DOS.^6^ However, the commercial availability of amines of this type is limited.^7^ Classical strategies for accessing these scaffolds include Heck and Tsuji–Trost reactions. These have been supplemented by recent approaches including Pd-catalyzed direct amination of allylic alcohols^8^ and visible-light-mediated Pd-catalyzed homologative three-component synthesis.^9^

Recently, we developed a rare photocatalytic polarity-mismatched C­(sp^3^)–C­(sp^2^) nucleophile cross-coupling of redox-active esters and alkenyl boronic acids (Scheme 1a),^10^ which employed NHPI esters as radical precursors.^11^

**Scheme 1:**
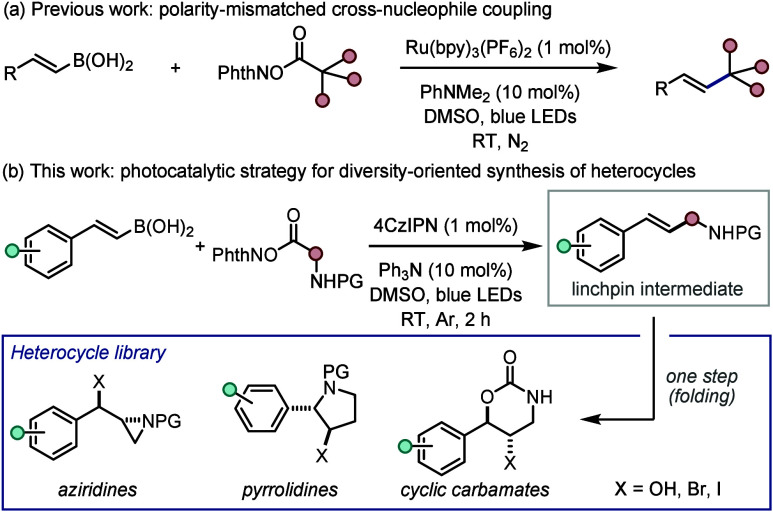
(a) Polarity-Mismatched Photocatalytic Coupling and (b) Its Application in DOS

Herein, we detail the utilization of this polarity-mismatched coupling process as a platform for the DOS of heterocyclic scaffolds from simple starting materials (Scheme 1b).

A benchmark system comprising styreneboronic acid **1** and NHPI ester **2** was used for reaction development (Table 1), with optimized conditions established, giving **3** in a good yield (Entry 1). These conditions compared favorably to our previous conditions,^10b^ which gave **3** in comparable yield (Entry 2), but using a metal-free photocatalyst.

**Table 1:**
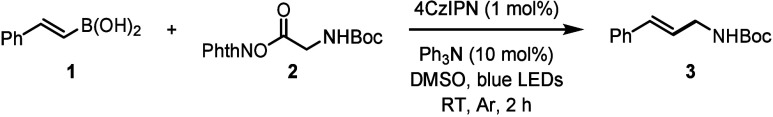
Reaction Development^a^

Entry	Deviation from ‘Standard Conditions’	**3** (%)^b^
1	-	73
2	PhNMe_2_	70
3	No additive	20
4	No catalyst, no additive	0
5	Ru(bpy)_3_(PF_6_)_2_ as catalyst	65
6	Ir(ppy)_3_ as catalyst	39
7	30 min reaction time	15
8	4 h reaction time	57

a Reaction conditions: NHPI (0.20 mmol, 1.0 equiv), styrene boronic acid (0.40 mmol, 2.0 equiv), catalyst (1.0 mol %), additive (10 mol %), DMSO (0.20 M).

b Determined by ^1^H NMR analysis of the crude reaction mixture using TCE or nitromethane as an internal standard.

We have previously noted the role of the aniline-based additives as an electron shuttle in this reaction, so we were interested to note reactivity in the absence of amine additive, which may imply some catalyst turnover enabled by the nitrogen-containing group in the starting material or product (Entries 1, 2, and 3). Removal of the catalyst and additive gave no reaction (Entry 4). Examination of alternative photocatalysts revealed better performance of the organic photocatalyst, 4CzIPN, as compared with common transition-metal-based catalysts (Entries 5 and 6). The origin of this difference is unclear but consistent with our previous observations.^10^ The reaction time was optimal at 2 h, with a decrease in yield after prolonged exposure to blue light suggesting a certain degree of product instability under the reaction conditions (Entries 7 and 8). Other conditions such as light source, solvent, and additive identity as well as alternative nitrogen protecting groups were evaluated (for full optimization data, see the SI).

The optimized conditions were effective toward generating a small library of allylamine and homoallylamine products (Scheme 2). We observed improved tolerance for steric bulk at the α-amino position in comparison to our previous system (**2**, **10**, and **11**).^10a^ Electron-withdrawing substituents on the benzene ring were well tolerated (**16**). An example of the product containing an electron-donating group was also accessed (**8**). Removal of the NH gave consistent increases in the product yield (**6**, **12**–**14**). One-carbon homologation of the system was equally well tolerated, achieving excellent yields (**17**, **19**–**23**). Cyclic amines were also accommodated (**25**, **26**).

**Scheme 2:**
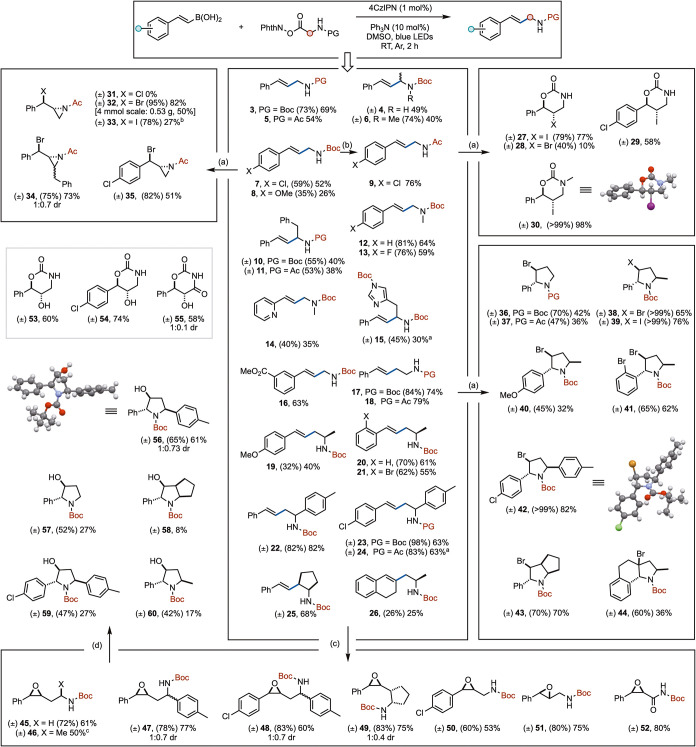
Polarity Mismatched Radical Cross-Coupling Enables DOS of Heterocycles[Fn sch2-fn5]

a Compound obtained using conditions from Brals et al.: NHPI (0.20 mmol, 1.0 equiv), styrene boronic acid (0.40 mmol, 2.0 equiv), Ru­(bpy)_3_(PF_6_)_2_ (1 mol %), PhNMe_2_ (10 mol %), dry and degassed DMSO (0.2 M), blue LED, RT, Ar, 3 h.^10a^

b Compound is light/temperature sensitive and degrades on purification.
c dr could not be determined.
d Reaction conditions: (a) NXS (1.20–3.00 equiv), MeCN (0.1 M), RT, Ar, 4–20 h; (b) TFA/DCM (1:1, 0.1 M), RT, 20 h, then Ac_2_O (1.30 equiv), Na_2_CO_3_ (3.00 equiv), THF/H_2_O (10:1, 0.1 M), RT, 20 h; (c) *m*CPBA (3.00 equiv), THF (0.1 M), RT, Ar, 20 h; (d) BF_3_·OEt_2_ (1.00–2.00 equiv), Et_2_O (0.1 M), RT, Ar, 5–20 h. Yield in parentheses calculated via ^1^H NMR analysis of the crude reaction mixture using TCE or nitromethane as an internal standard. Products isolated as a single diastereomer unless otherwise noted.

Using this straightforward approach to allyl- and homoallylamines allowed rapid exploration of chemical space via DOS. Treatment of Boc-protected amines **1**, **6**, and **7** with NIS gave halo-oxazolidin-2-ones **27**, **29**, and **30**, respectively, with moderate to excellent yields,^12^ with stereochemistry unambiguously confirmed using SCXRD. NBS likewise delivered the corresponding bromo-oxazinan-2-one **28** in lower yield.

Access to aziridines required the use of the corresponding acetamide. This was readily achieved either by use of the acetamide in the photochemical coupling or by protecting group switching, if desired (both operate smoothly). Treatment of **5** with NXS reagents delivered the α-bromoaziridine **32** and α-iodoaziridine **33** in good yield; however, **33** was found to be sensitive to degradation during purification. The same conditions were used to prepare aziridines **34** and **35**.

The pyrrolidine moiety is prolific in medicinal chemistry.^13^ Subjecting homoallyl amines **17**–**21**, **23**, **25** and **26** to NXS afforded substituted pyrrolidines in good to excellent yields (**36**–**44**). In this case, pyrrolidine products were delivered regardless of the protecting group used (e.g., **36** vs **37**). Bicyclic [4.3.0] and [3.3.0] systems are found in many natural products and are increasingly common in medicinal chemistry.^14^ These scaffolds are readily accessed through the same approach (**43**, **44**). Stereochemistry was again confirmed by SCXRD.

Lastly, **1**, **7**, **17**, **22**, **23**, and **25** were smoothly converted to the corresponding epoxides (**45**–**52**) in good yield, which provided access to hydroxy-oxazolidin-2-ones (**53**–**55**).

Finally, to visualize the structural diversity generated through this DOS approach, we used uniform manifold approximation and projection (UMAP).^15^ This machine learning technique reduces the multidimensional data to a two-dimensional graph based on similarities in constituent atoms and their connectivity. All compounds generated during the study were processed to form the plot shown in Figure 1. As expected, each category of structures appears as a separate clusters. The allylic amines occupy the top of the graph (burgundy) and span across the largest aera, which is understandable, as the largest substrate scope was explored here.

**Figure 1:**
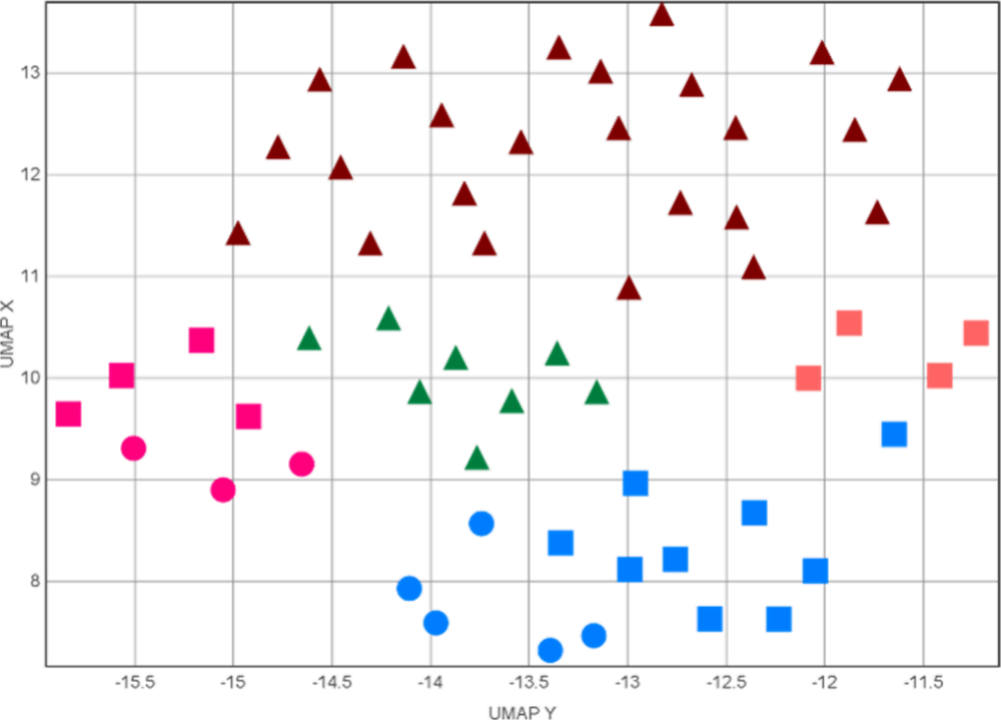
Uniform manifold approximation and projection (UMAP) visualization of structures accessed in this study. Burgundy - linchpin intermediates, orange - aziridines, green - epoxides, blue - pyrrolidines, and pink - oxazinan-2-ones. Triangles - (homo)­allylic amines and epoxides, circles - hydroxyvariants, and squares - halovariants.

The epoxide intermediates (green) can be seen in the middle of the plot closely related to the oxazine-2-ones on the left-hand side (pink). Aziridines are shown in the middle right (orange), and pyrrolidines occupy the bottom center part of the plot (blue). The one pyrrolidine data point, which appears to cluster with the aziridines, represents compound **37**, which shares the Ac protecting group with the accessed aziridines (as opposed to Boc used for the rest of the pyrrolidines). It is worth noting that the algorithm displays little difference between the hydroxy (spheres) and halovariants (squares) of both oxazine-2-one and pyrrolidine structures.

In summary, we have demonstrated the application of photocatalytic cross-nucleophile couplings to provide rapid access to stereodefined (homo)­allylic amines. We have also shown their utility in synthesis of diverse scaffolds using a range of cyclization techniques and created a library of structurally diverse aza-heterocyclic compounds (α-haloaziridines, pyrrolidines, and oxazine-2-ones) of relevance to medicinal chemistry. Finally, we have illustrated the diversity of the accessed structures by plotting the similarity reduced to two dimensions by a uniform manifold approximation and projection technique, which showed clustering of the same types of structures and logical trends in correlations between them.

## Supplementary Material



## Data Availability

The data underlying this study are available in the published article, in its Supporting Information, and openly available at the University of St Andrews at 10.17630/0917aa4e-fd72-4d2f-aa85-d49b1d06d5a5.

## Notes

Reaction optimization, compound characterization data, crystal structure data, DFT calculations, and NMR spectra (PDF)
