# Supplementary material for: A Polarity-Mismatched Photocatalytic Cross-Coupling Enables Diversity-Oriented Synthesis of aza-Heterocycles
Source: Org Lett. 2026 Feb 3;28(6):1953–7. doi: 10.1021/acs.orglett.5c04971 (PMC12910714; doi:10.1021/acs.orglett.5c04971)
Supplement: Supplementary file 1 [file ol5c04971_si_001.pdf]

Supporting Information

A polarity-mismatched photocatalytic cross-coupling enables diversity-oriented synthesis of aza-heterocycles

Joanna Urbańczyk,[a] Aidan P. McKay,[a] David B. Cordes,[a] Tomas Lebl,[a] Miles H. Aukland,[†b] Allan J. B. Watson[a*]

[a] EaStCHEM, School of Chemistry, University of St Andrews, St Andrews, Fife, KY16 9ST, United Kingdom.
[b] Chemical Development, Pharmaceutical Technology & Development, Operations, AstraZeneca, Macclesfield, United Kingdom.

*Email: aw260@st-andrews.ac.uk

Contents:

1. General Information 2
   a. Purification of Solvents and Reagents 2
   b. Experimental Details 2
   c. Purification of Products 2
   d. Analysis of Products 2
   e. X-Ray Diffraction Data 3
2. General Procedures 7
   f. General Procedure A– Amino Acid Protection 7
   g. General Procedure B – NHPI Ester Synthesis 7
   h. General Procedure C – Photocatalytic Coupling 7
   i. General Procedure D – Reprotection of Intermediates 8
   j. General Procedure E – NXS-Mediated Cyclisation 8
   k. General Procedure F – Epoxidation 9
   l. General Procedure G – Intramolecular Epoxide Opening 9
   m. Scale-up Synthesis of Aziridine 9
3. Photocoupling Optimisation 11
4. Characterisation Data 13
5. DFT Calculations 57
6. References 64
7. Spectra 66

General Information

Purification of Solvents and Reagents

Reagents and solvents were obtained from commercial suppliers and used without further purification unless otherwise stated. Dry solvents (THF, CH_2Cl_2, Et_2O, MeCN) were obtained *via* a PureSolv SPS–400–5 system and stored over activated 4 Å molecular sieves under inert gas. Degassing of DMSO was performed by argon bubbling for 30 minutes.

Experimental Details

Reactions were carried out under inert atmosphere unless otherwise stated. Reactions were carried out using standard borosilicate laboratory glassware, microwave vials or 4 mL photoreactor vials, which had been dried in an oven at 180 °C or flame-dried before use. Room temperature reactions are assumed to be 18 – 25 °C. Reactions at 0 °C were performed in ice/water baths. Reactions carried out at elevated temperature were performed using a temperature-regulated hotplate-stirrer fitted with a thermocouple and a sand bath. Reactions under light irradiation were carried out in a photobox equipped with an Kessil PR160-456 nm (referred to as 'blue light' below) set to 100% intensity and a cooling fan.

Purification of Products

TLC was carried out using Merck aluminium-backed silica gel plates coated with F254 fluorescent indicator, analysed under UV light and/or developed using ethanolic vanillin solutions and applying heating as appropriate. Column chromatography was performed with compressed air using silica gel (40 – 62 µm, Fluorochem) and porosity grade 2 or 3 sintered disks. Basified silica was prepared by flushing the silica gel with 3% Et_3N in cyclohexane before loading the column.

Analysis of Products

^{1}H, ^{13}C and ^{19}F NMR were obtained using: Bruker AV 400 fitted with a BBFO probe (^{1}H 400 MHz; ^{13}C 101 MHz, ^{11}B 128 MHz), a Bruker AVII 400 fitted with a BBFO probe (^{1}H 400 MHz; ^{13}C 101 MHz), a Bruker AVIII HD 500 fitted with a SmartProbe BBFO+ probe (^{1}H 500 MHz; ^{13}C 126 MHz), or a Bruker AVIII 500 fitted with a Prodigy BBFO probe (^{1}H 500 MHz; ^{13}C 126 MHz; ^{19}F 471 MHz). ^{13}C, ^{19}F were ^{1}H decoupled. All chemical shifts (δ) are reported in parts per million (ppm) relative to the residual solvent peak, with $CDCl_3$ referenced at 7.26 ppm (^{1}H) and 77.0 ppm (^{13}C), DMSO-d_6 referenced at 2.50 (^{1}H) and 39.5 ppm (^{13}C) and acetone-d_6 referenced at 2.05 (^{1}H) and 29.8, 206.3 (^{13}C). Multiplicity is given as br (broad), s (singlet), d (doublet), t (triplet), q (quartet), quint. (quintet), hept. (heptet), or m (multiplet), or combinations thereof. Signals which overlap with one another are described as multiplets. All coupling constants, J, are quoted in Hz and are $^{3}J_{HH}$ unless otherwise stated. Selective irradiation in 1D NOE experiments was used to confirm the appearance of two distinct sets of signals for pyrrolidines arising due to rotamers and determine relative stereochemistry of the products.[1]

IR spectra were recorded using a Shimadzu IT Affinity–1 Fourier transform IR spectrophotometer with a Specac Quest ATR (diamond puck). Spectra were recorded as films (using diethyl ether) or as solids, as specified. Transmittance was recorded with maximal absorption wavenumbers given as cm^{-1}.

Mass spectra were recorded on a Bruker microTOF benchtop ESI with either positive or negative electrospray ionisation or EI using a Thermo Mat 900XP, Double Focussing Hi-resolution mass spectrometer or 7T Fourier Transform Ion Cyclotron Resonance mass spectrometer at the University of Edinburgh mass spectrometry facility (SIRCAMS) or on Thermo Scientific Orbitrap Exploris MX at the University of St Andrews.

X-Ray Diffraction Data

Crystals for the X-ray diffraction analysis were prepared by slow evaporation from ethyl acetate (**42** and **56**) or acetone (**30**).

X-ray diffraction data for compounds **30** and **56** were collected at 100 K using either a Rigaku MM-007HF High Brilliance RA generator/confocal optics/XtaLAB P200 or a Rigaku Synergy-R/confocal optics/HyPix-Arc 100 [Cu Kα radiation (λ = 1.54187 Å)]. Diffraction data for compound **42** were collected at 100 K using a Rigaku FR-X Ultrahigh Brilliance Microfocus RA generator/confocal optics/XtaLAB P200 [Mo Kα radiation (λ = 0.71073 Å)]. Intensity data for all compounds analysed were collected (using a calculated strategy) and processed (including correction for Lorentz, polarization and absorption) using CrysAlisPro.[2] Structures were solved by dual-space methods (SHELXT)[3] and refined by full-matrix least-squares against F^2 (SHELXL-2019/3).[4] Non-hydrogen atoms were refined anisotropically, and hydrogen atoms were refined using a riding model, except for the OH hydrogen in **56** which was located from the difference Fourier map and refined isotropically subject to a distance restraint. The structure of **30** showed disorder in the central oxazinan-2-one ring which was modelled using two positions and refined with geometric and thermal restraints. Crystals of **56** were affected by non-merohedral twinning; the second component being rotated by 179.96° around [-0.09 0.00 1.00] (reciprocal) or [0.00 -0.00 1.00] (direct), giving a twin law of [-1 -0.0140 -0.1750 0.0001 -1.0001 -0.0012 -0.0014 1.0003] and a refined twin fraction of 0.52. All calculations were performed using the Olex2 interface.[5] Selected crystallographic data are presented in Table 1. CCDC 2493539-2493541 contains the supplementary crystallographic data for this paper. These data can be obtained free of charge from The Cambridge Crystallographic Data Centre via www.ccdc.cam.ac.uk/structures.

Table 1 Selected crystallographic data.

	**30**	**42**	**56**
formula	$C_{11}H_{12}INO_2$	$C_{22}H_{25}NO_2ClBr$	$C_{22}H_{27}NO_3$
fw	317.12	450.79	353.44
crystal description	Colourless prism	Colourless prism	Colourless blade
crystal size [mm^3]	0.12×0.07×0.03	0.14×0.10×0.05	0.263×0.050×0.017
temperature [K]	100	100	100
space group	$P2_1/n$	$P\bar{1}$	$C2/c$

a [Å]	10.4869(5)	6.27536(15)	53.6806(7)
b [Å]	5.8600(2)	9.7775(3)	5.85384(5)
c [Å]	19.1456(10)	17.4257(4)	24.3925(3)
α [°]		101.964(2)	
β [°]	103.737(5)	94.990(2)	92.3544(12)
γ [°]		92.262(2)	
vol [Å]^3	1142.91(10)	1040.18(5)	7658.57(16)
Z	4	2	16
ρ (calc) [g/cm^3]	1.843	1.439	1.226
μ [mm^{-1}]	21.866	2.121	0.644
F(000)	616	464	3040
reflections collected	40834	58957	92015
independent reflections (R_{int})	2365 (0.1212)	6306 (0.0552)	12482 (0.0528)
parameters, restraints	156, 18	248, 0	486, 2
GoF on F^2	1.110	1.056	1.198
R_1 [$I > 2\sigma(I)$]	0.0618	0.0314	0.0733
wR_2 (all data)	0.1688	0.0821	0.1645
largest diff. peak/hole [e/Å^3]	1.210, -2.410	0.702, -0.323	0.546, -0.237

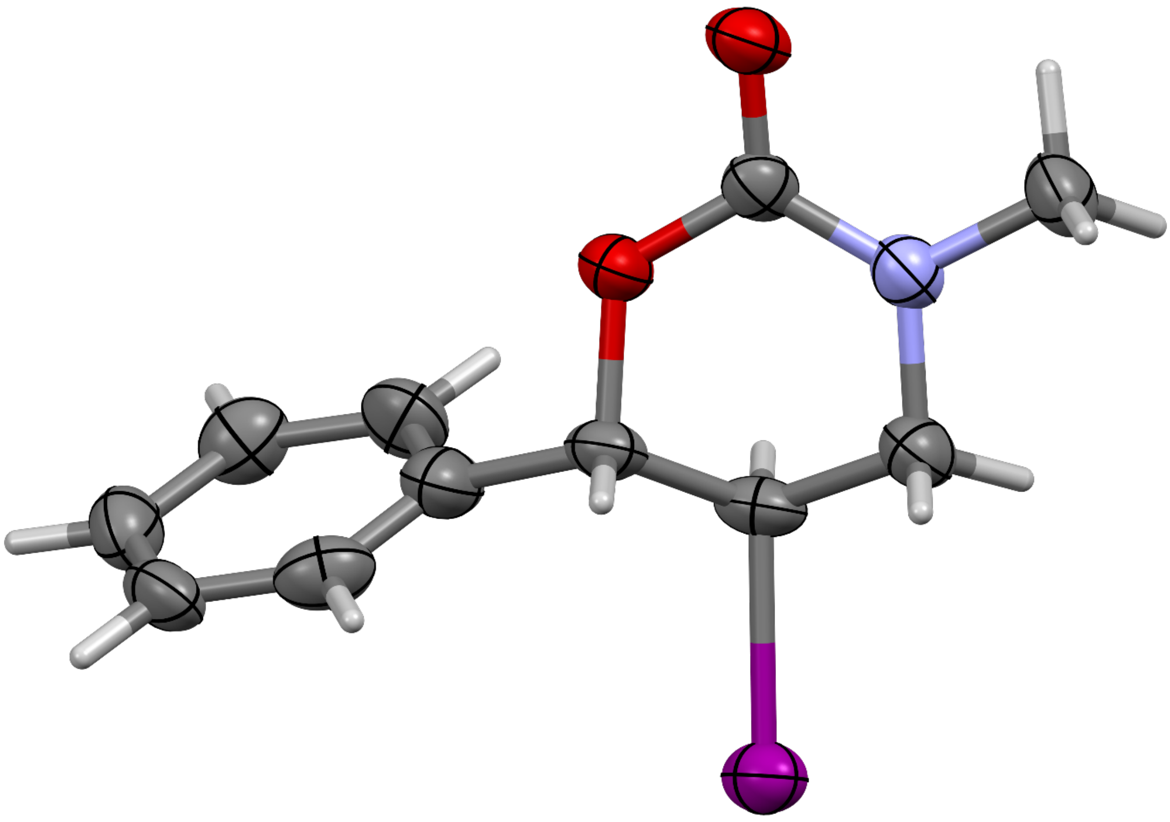

Figure S1. Thermal ellipsoid plot of the structure of **30**, with 50 % ellipsoid probability. Minor component of disorder omitted for clarity.

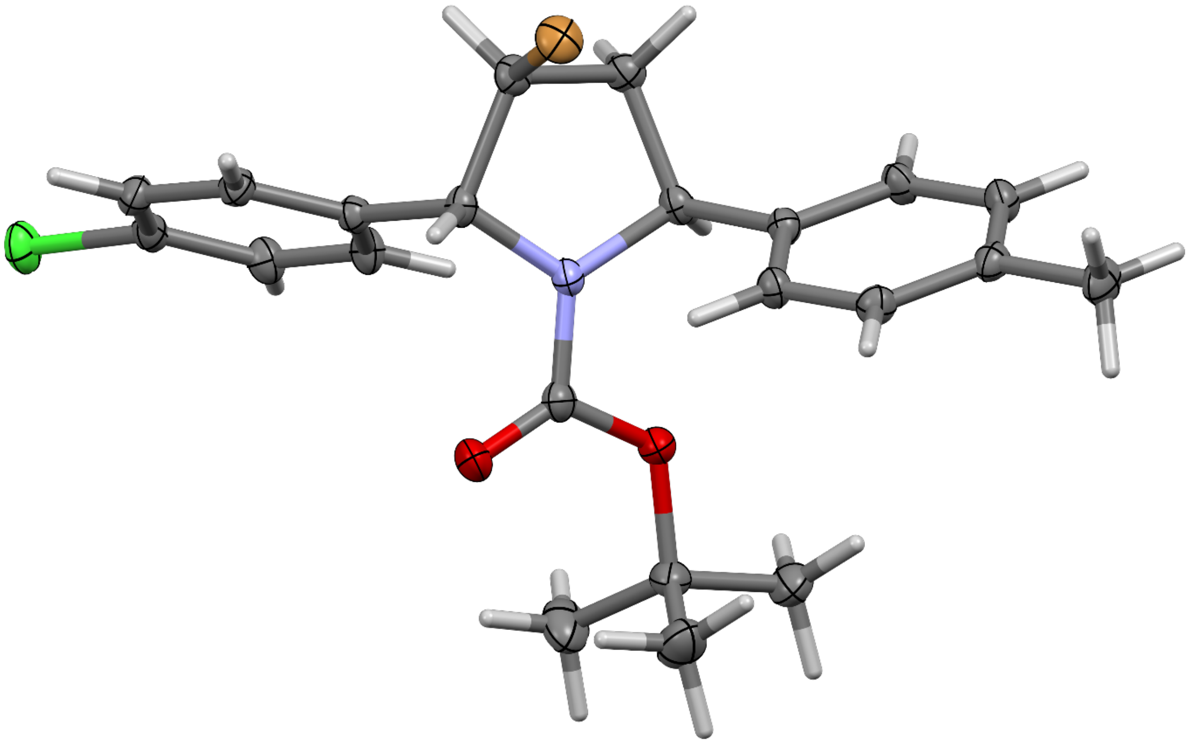

Figure S2. Thermal ellipsoid plot of the structure of **42**, with 50 % ellipsoid probability.

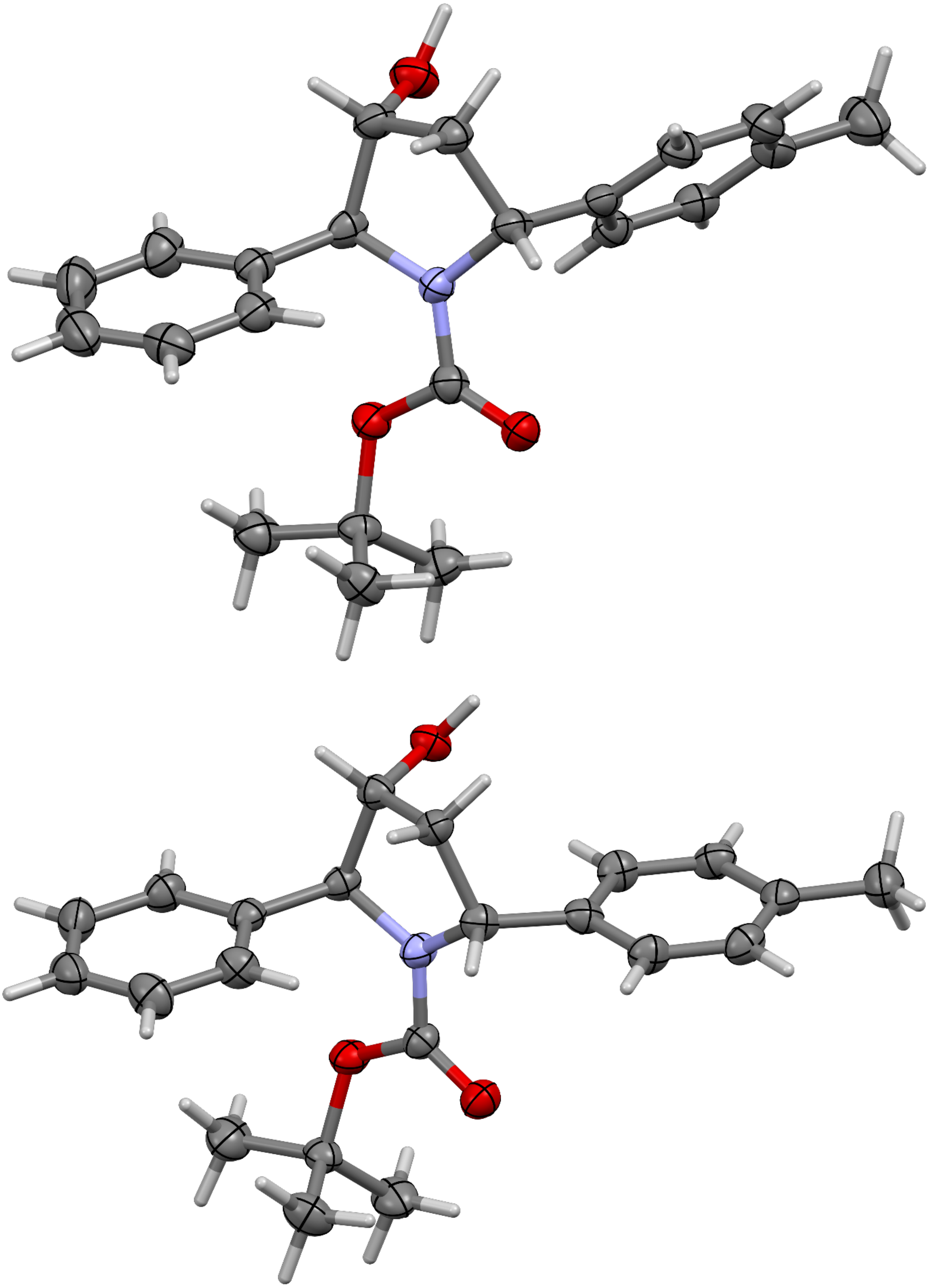

Figure S3. Thermal ellipsoid plots of both independent molecules in the structure of **56**, with 50 % ellipsoid probability.

General Procedures

General Procedure A– Amino Acid Protection

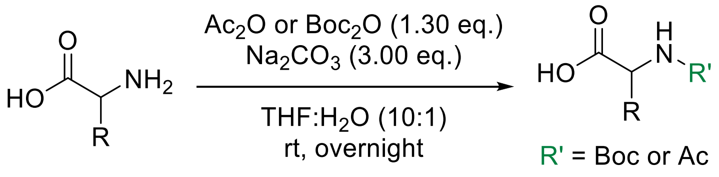

A round-bottom flask equipped with a Teflon-coated stir bar was charged with the requisite amino acid (1.00 eq.) and THF:H_2O (10:1, 0.5 M). Na_2CO_3 (3.00 eq.) and Ac_2O or Boc_2O (1.30 eq.) were then sequentially added to the flask. The mixture was stirred at room temperature overnight. The reaction mixture was acidified to pH 2 using aq. HCl (2 M) and extracted with ethyl acetate (2 × 2 mL/mmol amino acid). The combined organic extracts were dried over Na_2SO_4, filtered and the solvent was removed *in vacuo* to afford the desired product in sufficient purity as to not require further purification.

General Procedure B – NHPI Ester Synthesis

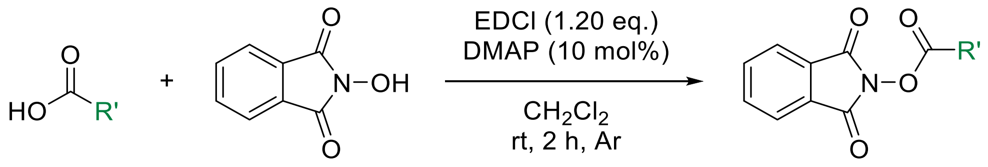

A flame-dried Schlenk flask equipped with a Teflon-coated stir bar was charged with the protected amino acid derivative (1.00 eq.), EDC hydrochloride (1.20 eq.), and 4-dimethylaminopyridine (10 mol%). The flask was sealed, evacuated, and backfilled with Ar 3 times before CH_2Cl_2 (0.1 M) was added. *N*-Hydroxyphthalimide (1.00 eq.) was then added portionwise to the reaction mixture under Ar flow. The mixture was stirred at room temperature for 2 h. The reaction mixture was then washed with aq. HCl (1 M, 5 mL/mmol amino acid derivative). The organic phase was collected and washed with brine (5 mL/mmol amino acid derivative) before being dried over Na_2SO_4 and filtered. The solvent was removed *in vacuo* to afford the crude product which was subsequently purified by trituration with hexane followed by vacuum filtration.

General Procedure C – Photocatalytic Coupling

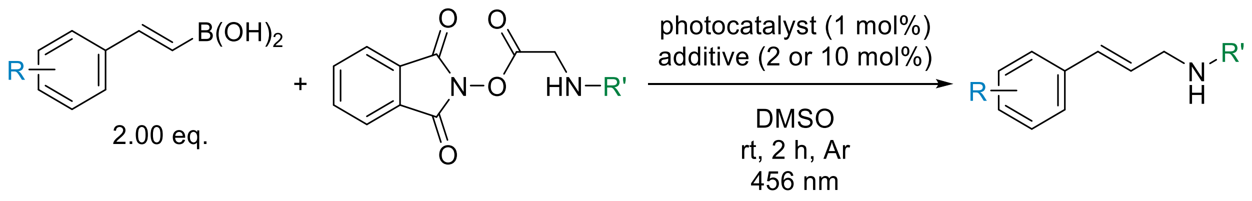

An oven-dried photoreactor vial, equipped with a Teflon-coated stir bar, was charged with the requisite NHPI ester (1.00 eq.), styrylboronic acid derivative (2.00 eq.), photocatalyst (1 mol%) and additive (2 or 10 mol%, *added at this stage if solid*). The flask was sealed, evacuated, and backfilled with Ar 3 times before dry, degassed DMSO (0.2 M) was added followed by additive

(2 or 10 mol%, *added at this stage if liquid*). The cap was wrapped with parafilm and the mixture was stirred for 2 hours under blue light under Ar. The reaction mixture was partitioned between diethyl ether (25 mL/mmol NHPI ester) and brine (25 mL/mmol NHPI ester). Organics were extracted with diethyl ether (2 × 50 mL/mmol NHPI ester). The combined organic extracts were washed with brine (75 mL/mmol NHPI ester), before being dried over Na_2SO_4 and filtered. The solvent was removed *in vacuo* to afford the crude product which was subsequently purified by flash column chromatography (basified silica gel, *see below for individual solvent systems*).

General Procedure D – Reprotection of Intermediates

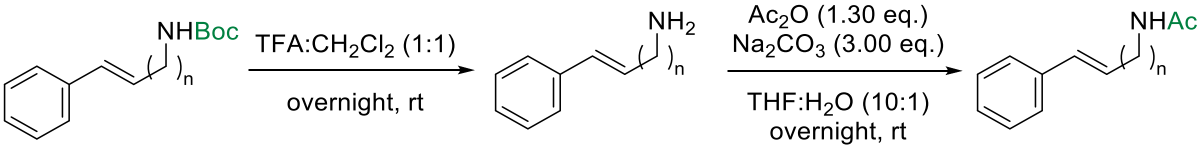

In a 4 mL vial equipped with a Teflon-coated stir bar, the Boc protected (homo)-*trans*-cinnamylamine (1.00 eq.) was dissolved in TFA:CH_2Cl_2 (1:1, 0.1 M) and the mixture was stirred overnight at room temperature. The mixture was then basified with aq. NaOH (1 M), extracted with CH_2Cl_2 (3 × 20 mL) dried over Na_2SO_4, filtered and the solvent was removed *in vacuo*. The crude was dissolved in THF:H_2O (10:1, 0.1 M). Na_2CO_3 (3.00 eq.) and Ac_2O (1.30 eq.) were added. The mixture was stirred at room temperature overnight. The mixture was acidified to pH 2 with aq. HCl (2 M) and extracted with diethyl ether (3 × 15 mL/mmol (homo)-*trans*-cinnamylamine). The combined organic extracts were dried over Na_2SO_4 and filtered. The solvent was removed *in vacuo* to afford the crude product which was subsequently purified by flash column chromatography (basified silica gel, *see below for individual solvent systems*).

General Procedure E – NXS-Mediated Cyclisation

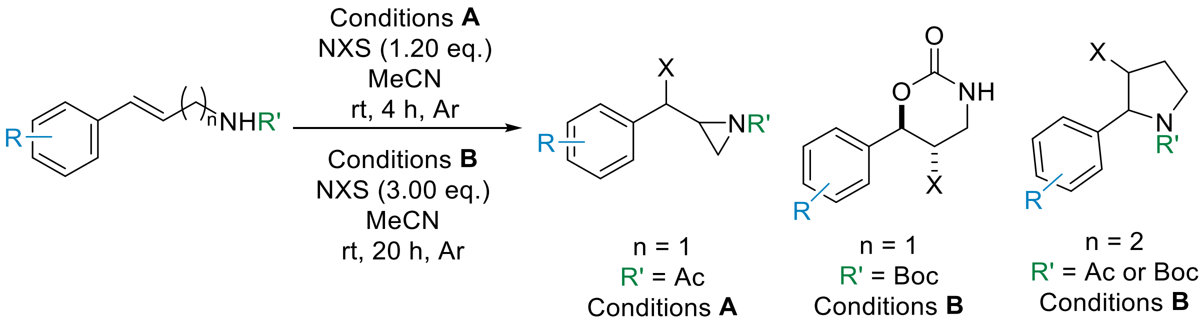

A 4 mL vial or a two-necked round bottom flask equipped with a Teflon-coated stir bar was charged with the requisite (homo)-*trans*-cinnamylamine (1.00 eq.), and NXS (1.20 eq., for aziridine formation, or 3.00 eq., otherwise) and capped with a septum cap. The flask was sealed, evacuated, and backfilled with Ar three times before MeCN (0.1 M) was added. The mixture was stirred then at room temperature for 4 (for aziridine formation) or 20 h (otherwise). The reaction mixture was then washed with 10% aq. $Na_2S_2O_3$ solution (15 mL/mmol (homo)-*trans*-cinnamylamine). The mixture was extracted with Et_2O (3 × 15 mL/mmol (homo)-*trans*-cinnamylamine), dried over Na_2SO_4 and filtered. The solvent was removed *in vacuo* to afford

the crude product which was subsequently purified by flash column chromatography (basified silica gel, *see below for individual solvent systems*).

General Procedure F – Epoxidation

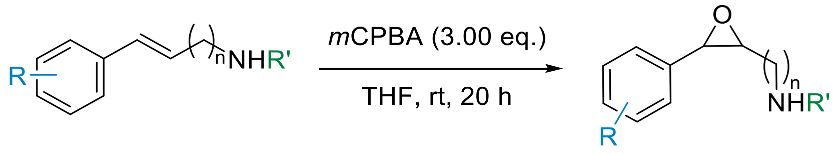

A round bottom flask equipped with a Teflon-coated stir bar was charged with the protected *trans*-cinnamylamine derivative (1.00 eq.). THF (0.1 M) was added followed by *m*CPBA (3.00 eq.). The mixture was stirred at room temperature for 20 h. The reaction mixture was then washed with sat. aq. $Na_2S_2O_3$ solution (25 mL/mmol (homo)-*trans*-cinnamylamine). The mixture was extracted with EtOAc (3 × 15 mL/mmol (homo)-*trans*-cinnamylamine), washed with brine (25 mL/mmol (homo)-*trans*-cinnamylamine), dried over $MgSO_4$ and filtered. The solvent was removed *in vacuo* to afford the crude product which was subsequently purified by flash column chromatography (basified silica gel, *see below for individual solvent systems*).

General Procedure G – Intramolecular Epoxide Opening

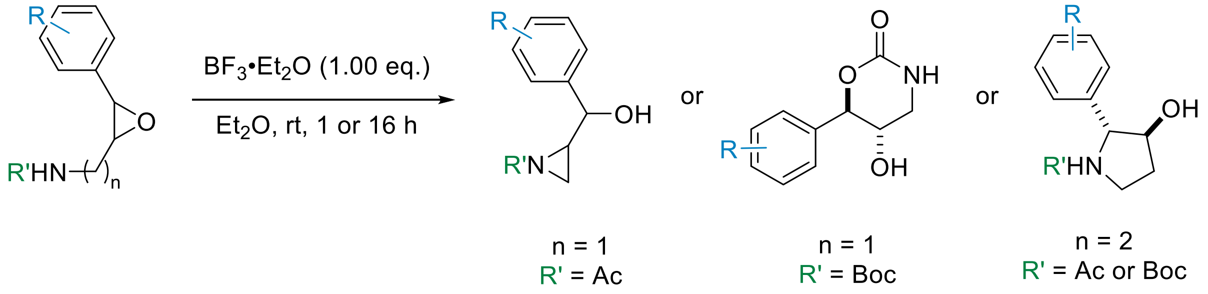

A round bottom flask equipped with a Teflon-coated stir bar was charged with the requisite epoxide (1.00 eq.). Et_2O (0.1 M) was added followed by boron trifluoride diethyl etherate (1.00 eq.).

For n = 1, R' = Ac and n = 2, R' = Ac or Boc: The reaction mixture was stirred at room temperature for 1 h. The reaction mixture was then washed with sat. aq. $Na_2S_2O_3$ solution (25 mL/mmol epoxide). The mixture was extracted with EtOAc (3 × 15 mL/mmol epoxide), washed with brine (25 mL/mmol epoxide), dried over Na_2SO_4 and filtered. The solvent was removed *in vacuo* to afford the crude product which was subsequently purified by flash column chromatography (basified silica gel, *see below for individual solvent systems*).

For n = 1, R' = Boc: The reaction mixture was stirred at room temperature for 20 h. The white precipitate formed and was filtered to afford the desired product in sufficient purity as to not require further purification.

Scale-up Synthesis of Aziridine

The reaction was performed with fumehood lights switched off and all containers were wrapped in aluminum foil. The product was stored in the freezer.

A flame-dried 100 mL two-necked round bottom flask equipped with a Teflon-coated stir bar was charged with the *N*-cinnamylacetamide (0.731 g, 4.17 mmol, 1.00 eq.), and 1-bromopyrrolidine-2,5-dione (0.890 g, 5.00 mmol, 1.20 eq.) and capped with a Suba seal. The flask was purged using a vacuum – Ar cycle (3 times). Dry MeCN (40 mL) was added. The reaction mixture was left to stir at room temperature for 4 hours. The reaction mixture was then washed with 10% aq. $Na_2S_2O_3$ solution (20 mL). The organics were extracted with Et_2O (3 × 20 mL), dried over Na_2SO_4, filtered and the solvent was removed *in vacuo*. The crude was purified by column chromatography (basified SiO_2, 0 to 20% ethyl acetate in cyclohexane) to give 1-(2-(bromo(phenyl)methyl)aziridin-1-yl)ethan-1-one (0.53 g, 50%) as a pale yellow oil.

Photocoupling Optimisation

Table 2 Control reactions employing Ru-based catalyst.

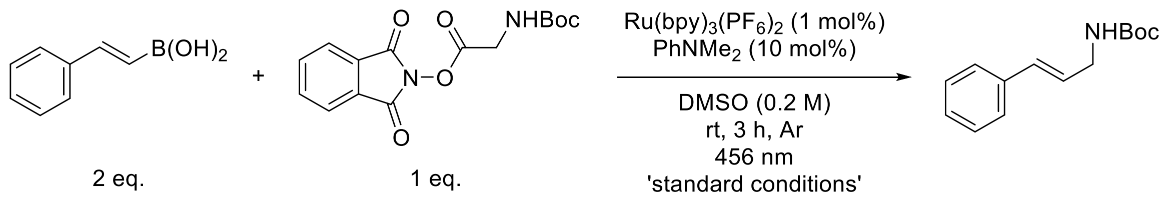

Entry	Deviation from standard conditions	Yield[a]
1	-	58%[b]
2	50% lamp intensity	47%
3	Starting material ratio inversed	28%

Reaction conditions: NHPI (0.20 mmol, 1.0 equiv.), styrene boronic acid (0.40 mmol, 2.0 equiv.), $Ru(bpy)_3(PF_6)_2$ (1 mol%), $PhNMe_2$ (10 mol%) in dry, degassed DMSO (2 mL). [a]NMR yield determined through ^1H NMR analysis of the crude reaction mixture using trichloroethylene or nitromethane as an internal standard. [b]Isolated yield by Brals *et al.*[6]

Table 3 Control reactions employing organophotocatalyst and comparative matallophotoredox results.

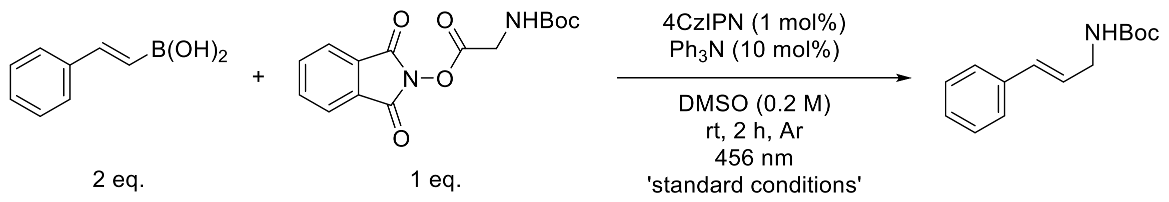

Entry	Deviation from 'Standard Conditions'	Yield[a]
1	-	73%
2	$PhNMe_2$	70%
3	No additive	20%
4	No catalyst, no additive	0%
5	$Ru(bpy)_3(PF_6)_2$ as catalyst	65%
6	$Ir(ppy)_3$ as catalyst	39%
7	30 min	15%
8	4 h	57%

Reaction conditions: NHPI (0.20 mmol, 1.0 equiv.), styrene boronic acid (0.40 mmol, 2.0 equiv.), 4CzIPN (1 mol%), Ph_3N (10 mol%) in dry, degassed DMSO (2 mL). [a]NMR yield determined through ^1H NMR analysis of the crude reaction mixture using trichloroethylene or nitromethane as an internal standard.

Table 4 N-Protecting Group Screen.

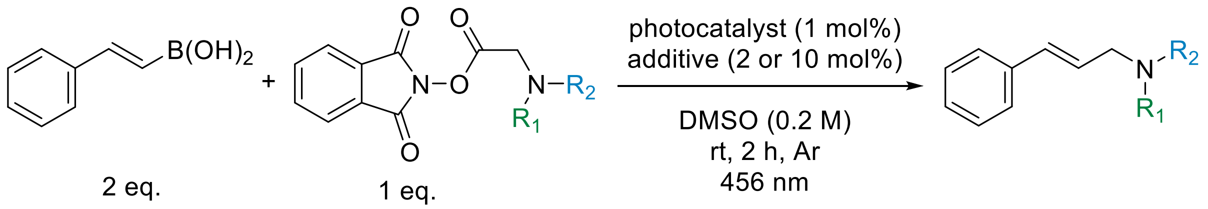

Entry	R_1	R_2	Yield
1	Bn	Bn	0%[a]
2	H	Ac	53%[a]<br>45%[b]
3	H	Boc	50%[a]
4	Me	Boc	75%[a]

[a] conditions: 4CzIPN (1 mol%), Ph_3N (2 mol%), DMSO (0.2 M), Ar, 2 h, rt, 456 nm;
[b] conditions: $Ru(bpy)_3(PF_6)_2$ (1 mol%), $PhNMe_2$ (10 mol%), DMSO (0.2 M), Ar, 3 h, rt, 456 nm.
NMR yield determined through ^1H NMR analysis of the crude reaction mixture using trichloroethylene or nitromethane as an internal standard.

Table 5 Further control reactions.

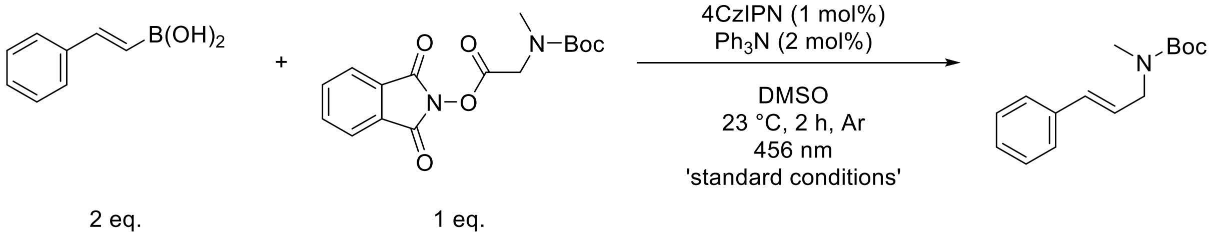

Entry	Deviation from the 'standard conditions'	Yield[a]
1	–	75% (67%)
2	Bench light instead of blue LED	0%
3	$Ir(dF(CF_3)ppy)_2dtbbpy(PF_6)$ as catalyst	8%
4	$Ir(dF(CF_3)ppy)_2dtbbpy(PF_6)$ as catalyst<br>370 nm instead of 456 nm	3%
5	$Ru(bpy)_3(PF_6)_2$ as catalyst	6%
6	$Ru(bpy)_3(PF_6)_2$ as catalyst<br>$PhNMe_2$ (10 mol%) as additive	70%
7	Catechol (10 mol%) as additive	60%
8	Phthalimide (10 mol%) as additive	8%
9	$PhNMe_2$ (10 mol%) as additive	83%
10	10 mol% additive loading	84% (63%)<br>81% (64%)
11	20 mol% additive loading	85% (70%)
12	4 h	73%
13	1 h	77% (68%)
14	DMF instead of DMSO	60%

[a] NMR yield determined through ^1H NMR analysis of the crude reaction mixture using trichloroethylene or nitromethane as an internal standard. Isolated yield given in parentheses.

Characterisation Data

Amino Acid Protection

N^{α},N^{τ}-Bis(*tert*-butoxycarbonyl)-*L*-histidine (2f')

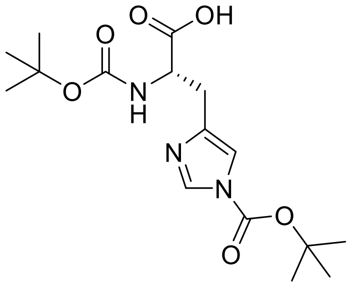

N^{α},N^{τ}-bis(*tert*-butoxycarbonyl)-*L*-histidine
Chemical Formula: $C_{16}H_{25}N_3O_6$
Molecular Weight: 355.39

Prepared according to General Procedure A with *L*-histidine (1.55 g, 10.0 mmol, 1.00 eq.), Na_2CO_3 (5.30 g, 50.0 mmol, 5.00 eq.) and Boc_2O (5.46 g, 25.0 mmol, 2.50 eq.) in THF:H_2O (20 mL). The desired product as a white solid (3.1 g, 87%) which was used without further purification.

^{1}H NMR (400 MHz, CDCl$_3$) δ 8.15 (s, 1H), 7.18 (s, 1H), 5.45 (br s, 1H), 4.49 (br s, 1H), 3.34 – 3.14 (m, 2H), 1.59 (d, *J* = 2.8 Hz, 9H), 1.45 (s, 9H).

Spectroscopic data in agreement with the literature.[7]

3-((*tert*-Butoxycarbonyl)amino)-3-(*p*-tolyl)propanoic acid (2g')

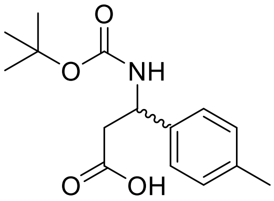

3-((*tert*-butoxycarbonyl)amino)-3-(*p*-tolyl)propanoic acid
Chemical Formula: $C_{15}H_{21}NO_4$
Molecular Weight: 279.34

Prepared according to General Procedure A with 3-amino-3-(*p*-tolyl)propionic acid (2.84 g, 10.0 mmol, 1.00 eq.), Na_2CO_3 (3.18 g, 30.0 mmol, 3.00 eq.) and Boc_2O (2.84 g, 13.0 mmol, 1.30 eq.) in THF:H_2O (20 mL) to afford the desired product as a white solid (2.8 g, >99%) which was used without further purification.

^{1}H NMR (500 MHz, CDCl$_3$) δ 7.21 – 7.16 (m, 2H), 7.16 – 7.12 (m, 2H), 5.33 (br s, 1H), 5.07 (br s, 1H), 3.05 – 2.67 (m, 2H), 2.32 (s, 3H), 1.53 (s, 9H).

^{13}C{^{1}H} NMR (126 MHz, CDCl$_3$) δ 163.1, 146.8, 137.4, 136.7, 129.4, 126.1, 85.2, 50.2, 40.4, 27.4, 21.1.

IR (solid): 3333, 2978, 1703, 1682, 1526, 1169, 1051 cm^{-1}.

HRMS (ESI): *m/z* calculated for $[M + Na]^+$ $(C_{15}H_{21}N_1O_4Na)^+$: 302.1363; found 302.1364.

(R)-3-(($tert$-Butoxycarbonyl)amino)butanoic acid (2i')

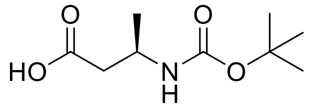

(R)-3-(($tert$-butoxycarbonyl)amino)butanoic acid
Chemical Formula: $C_9H_{17}NO_4$
Molecular Weight: 203.24

Prepared according to General Procedure A with 3-amino-3butanoic acid (1.03 g, 10.0 mmol, 1.00 eq.), Na_2CO_3 (3.18 g, 30 mmol, 3.00 eq.) and Boc_2O (2.84 g, 13.0 mmol, 1.30 eq.) in THF:H_2O (20 mL) to afford the desired product as a white solid (2.5 g, >99%) which was used without further purification.

^{1}H NMR (400 MHz, $CDCl_3$) δ 4.99 (s, 1H), 4.02 (s, 1H), 2.62 – 2.43 (m, 2H), 1.43 (s, 9H), 1.23 (d, J = 6.7 Hz, 3H).

Spectroscopic data in agreement with the literature.[8]

2-(($tert$-Butoxycarbonyl)amino)cyclopentane-1-carboxylic acid (2j')

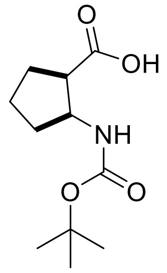

2-((*tert*-butoxycarbonyl)amino)cyclopentane-1-carboxylic acid
Chemical Formula: $C_{11}H_{19}NO_4$
Molecular Weight: 229.28

Prepared according to General Procedure A with 2-aminocyclopentane-1-carboxylic acid hydrochloride **M2** (0.55 g, 3.4 mmol, 1.0 eq.), Na_2CO_3 (1.5 g, 14 mmol, 4.0 eq.) and Boc_2O (0.99 g, 4.5 mmol, 1.3 eq.) in THF:H_2O (10 mL) to afford the desired product as a pale yellow oil (0.80 g, >99%) as a single diastereomer, which was used without further purification.

^{1}H NMR (500 MHz, $CDCl_3$) δ 6.78 (s, 0.6H), 5.14 (s, 0.4H), 4.30 – 3.98 (m, 1H), 3.14 – 2.99 (m, 1H), 2.12 – 1.54 (m, 6H), 1.47 (s, 9H).

Spectroscopic data in agreement with the literature.[9]

Acetyl glycine (2l')

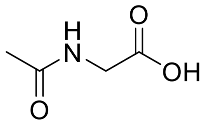

acetylglycine
Chemical Formula: $C_4H_7NO_3$
Molecular Weight: 117.10

In a round-bottom flask equipped with a Teflon-coated stir bar, glycine (3.00 g, 40.0 mmol, 1.00 eq.) was dissolved in H_2O (80 mL). Acetic anhydride (7.5 mL, 80 mmol, 2.0 eq.) was added and the mixture was stirred at room temperature for 1 h. The solvent was removed *in vacuo* to afford the desired product as a white solid (4.1 g, 88%).

^{1}H NMR (400 MHz, DMSO-d_6) δ 8.17 (br s, 1H), 3.71 (d, J = 4.80 Hz, 2H), 1.84 (s, 3H).

Spectroscopic data in agreement with the literature.[10]

tert-Butyl cinnamoylcarbamate (52a)

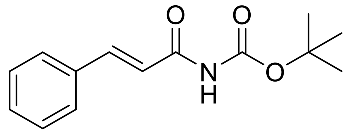

tert-butyl cinnamoylcarbamate
Chemical Formula: $C_{14}H_{17}NO_3$
Molecular Weight: 247.29

In a round-bottom flask equipped with a Teflon-coated stir bar, cinnamamide (1.47 g, 10.0 mmol, 1.00 eq.) was dissolved in CH_2Cl_2 (50 mL). NaH (60% in mineral oil, 0.440 g, 11.0 mmol, 1.10 eq.) was added at 0 °C and the mixture was stirred for 30 minutes before adding Boc_2O (2.40 g, 11.0 mmol, 1.10 eq.). The reaction mixture was stirred in air at room temperature overnight. After that saturated aqueous solution of NH_4Cl (20 mL) was added and the organics were extracted with EtOAc (2 × 25 mL), washed with brine (50 mL), dried over sodium sulfate and filtered. The solvent was removed *in vacuo* to afford white solid (1.3 g, 50%).

^{1}H NMR (500 MHz, $CDCl_3$) δ 7.83 (d, J = 15.8 Hz, 1H), 7.63 – 7.57 (m, 2H), 7.55 (d, J = 15.8 Hz, 1H), 7.41 – 7.37 (m, 3H), 7.35 (s, 1H), 1.53 (s, 9H).

^{13}C{^{1}H} NMR (126 MHz, $CDCl_3$) δ 166.5, 150.6, 145.8, 134.6, 130.5, 128.9, 128.5, 118.2, 82.7, 28.1.

IR (solid): 3289, 2989, 1736, 1624, 1499, 1250, 1128 cm^{-1}.

HRMS (ESI): *m/z* calculated for $[M + Na]^+$ $(C_{14}H_{17}N_1O_3Na_1)^+$: 270.1101; found 270.1098.

NHPI Ester Synthesis

1,3-Dioxoisoindolin-2-yl (_tert_-butoxycarbonyl)glycinate (2a)

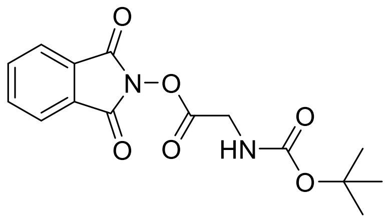

1,3-dioxoisoindolin-2-yl (*tert*-butoxycarbonyl)glycinate
Chemical Formula: $C_{15}H_{16}N_2O_6$
Molecular Weight: 320.30

Prepared according to General Procedure B with (*tert*-butoxycarbonyl)glycine (0.826 g, 5.00 mmol, 1.00 eq.), EDC hydrochloride (1.15 g, 6.00 mmol, 1.20 eq.), 4-dimethylaminopyridine (61 mg, 0.50 mmol, 10 mol%) and *N*-hydroxyphthalimide (0.816 g, 5.00 mmol, 1.00 eq.) in CH_2Cl_2 (50 mL) to afford the desired product as a white solid (1.4 g, 88%).

Rotamer ratio determined from crude ^{1}H NMR – 1.0:0.25.

Major rotamer:

^{1}H NMR (400 MHz, $CDCl_3$) δ 7.93 – 7.87 (m, 2H), 7.84 – 7.78 (m, 2H), 5.00 (br s, 1H), 4.36 (d, J = 5.73, 2H), 1.47 (s, 9H).

Minor rotamer:

^{1}H NMR (400 MHz, $CDCl_3$) δ 7.93 – 7.87 (m, 2H), 7.84 – 7.78 (m, 2H), 4.80 (br s, 1H), 4.21 (br s, 2H), 1.51 (s, 9H).

Spectroscopic data in agreement with the literature.[6]

1,3-Dioxoisoindolin-2-yl *N*-(*tert*-butoxycarbonyl)-*N*-methylglycinate (2b)

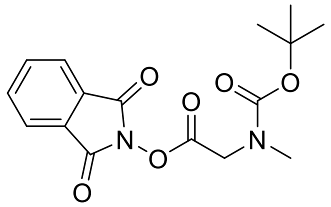

1,3-dioxoisoindolin-2-yl
N-(*tert*-butoxycarbonyl)-*N*-methylglycinate
Chemical Formula: $C_{16}H_{18}N_2O_6$
Molecular Weight: 334.33

Prepared according to General Procedure B with *N*-(*tert*-butoxycarbonyl)-*N*-methylglycine (0.378 g, 2.00 mmol, 1.00 eq.), EDC hydrochloride (0.460 g, 2.40 mmol, 1.20 eq.), 4-dimethylaminopyridine (24 mg, 0.20 mmol, 10 mol%) and *N*-hydroxyphthalimide (0.326 g, 2.00 mmol, 1.00 eq.) in CH_2Cl_2 (20 mL) to afford the desired product as a white solid (0.59 g, 88%).

Rotamer ratio determined from crude ^{1}H NMR – 1.0:0.45.

Major rotamer:

^{1}H NMR (500 MHz, $CDCl_3$) δ 7.89 – 7.87 (m, 2H), 7.81 – 7.77 (m, 2H), 4.29 (s, 2H), 3.01 (s, 3H), 1.49 (s, 9H).

Minor rotamer:

^{1}H NMR (500 MHz, $CDCl_3$) δ 7.89 – 7.87 (m, 2H), 7.81 – 7.77 (m, 2H), 4.42 (s, 2H), 2.98 (s, 3H), 1.47 (s, 9H).

Spectroscopic data in agreement with the literature.[11]

1,3-Dioxoisoindolin-2-yl *N*-(*tert*-butoxycarbonyl)-*N*-methyl-*L*-alaninate (2c)

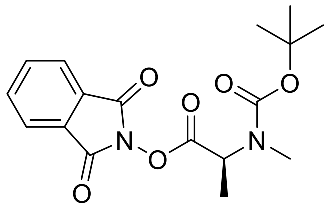

1,3-dioxoisoindolin-2-yl
N-(*tert*-butoxycarbonyl)-*N*-methyl-*L*-alaninate
Chemical Formula: $C_{17}H_{20}N_2O_6$
Molecular Weight: 348.36

Prepared according to General Procedure B with *N*-(*tert*-butoxycarbonyl)-*N*-methylalanine (0.406 g, 2.00 mmol, 1.00 eq.), EDC hydrochloride (0.460 g, 2.40 mmol, 1.20 eq.), 4-dimethylaminopyridine (0.024 g, 0.20 mmol, 10 mol%) and *N*-hydroxyphthalimide (0.326 g, 2.00 mmol, 1.00 eq.) in CH_2Cl_2 (20 mL) to afford the desired product as a white solid (0.38 g, 54%).

Major rotamer:

^{1}H NMR (500 MHz, $CDCl_3$) δ 7.91 – 7.85 (m, 2H), 7.82 – 7.76 (m, 2H), 4.90 (br s, 1H), 2.98 (s, 3H), 1.61 (d, J = 5.60 Hz, 3H), 1.51 (s, 9H).

Minor rotamer:

^{1}H NMR (500 MHz, $CDCl_3$) δ 7.91 – 7.85 (m, 2H), 7.82 – 7.76 (m, 2H), 5.33 (br s, 1H), 2.92 (s, 3H), 1.58 (d, J = 5.60 Hz, 3H), 1.51 (s, 9H).

Spectroscopic data in agreement with the literature.[12]

1,3-Dioxoisoindolin-2-yl (*tert*-butoxycarbonyl)-*L*-alaninate (2d)

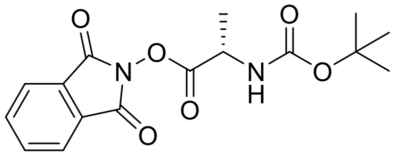

1,3-dioxoisoindolin-2-yl (*tert*-butoxycarbonyl)-*L*-alaninate
Chemical Formula: $C_{16}H_{18}N_2O_6$
Molecular Weight: 334.33

Prepared according to General Procedure B with Boc-alanine (0.946 g, 5.00 mmol, 1.00 eq.), EDC hydrochloride (1.15 g, 6.00 mmol, 1.20 eq.), 4-dimethylaminopyridine (60 mg, 0.50 mmol, 10 mol%) and *N*-hydroxyphthalimide (0.815 g, 5.00 mmol, 1.00 eq.) in CH_2Cl_2 (50 mL) to afford the desired product as a white solid (0.30 g, 18%).

Rotamer ratio determined from crude ^{1}H NMR – 1.0:0.34.

Major rotamer:

^{1}H NMR (400 MHz, $CDCl_3$) δ 7.96 – 7.89 (m, 2H), 7.88 – 7.79 (m, 2H), 5.06 (s, 1H), 4.80 (s, 1H), 1.66 (dd, J = 7.2, 2.2 Hz, 3H), 1.49 (s, 9H).

Minor rotamer:

^{1}H NMR (400 MHz, $CDCl_3$) δ 7.88 – 7.79 (m, 2H), 7.78 – 7.68 (m, 2H), 4.80 (s, 1H), 4.54 (s, 1H), 1.66 (dd, J = 7.2, 2.2 Hz, 3H), 1.47 (s, 9H).

Spectroscopic data in agreement with the literature.[13]

1,3-Dioxoisoindolin-2-yl acetyl-*D*-phenylalanine (2e)

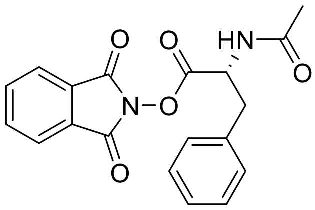

1,3-dioxoisoindolin-2-yl acetyl-*D*-phenylalaninate
Chemical Formula: $C_{19}H_{16}N_2O_5$
Molecular Weight: 352.35

Prepared according to General Procedure B with acetyl-*D*-phenylalanine (0.207 g, 1.00 mmol, 1.00 eq.), EDC hydrochloride (0.230 g, 1.20 mmol, 1.20 eq.), 4-dimethylaminopyridine (12 mg, 0.10 mmol, 10 mol%) and *N*-hydroxyphthalimide (0.163 g, 1.00 mmol, 1.00 eq.) in CH_2Cl_2 (10 mL) to afford the desired product as a white solid (0.23 g, 65%).

^{1}H NMR (400 MHz, CDCl$_3$) δ 7.98 – 7.86 (m, 2H), 7.86 – 7.78 (m, 2H), 7.39 – 7.28 (m, 5H), 5.81 (d, *J* = 8.2 Hz, 1H), 5.42 – 5.33 (m, 1H), 3.45 – 3.28 (m, 2H), 2.00 (s, 3H).

Spectroscopic data in agreement with the literature.[14]

tert-Butyl (*S*)-4-(2-((*tert*-butoxycarbonyl)amino)-3-((1,3-dioxoisoindolin-2-yl)oxy)-3-oxopropyl)-1*H*-imidazole-1-carboxylate (2f)

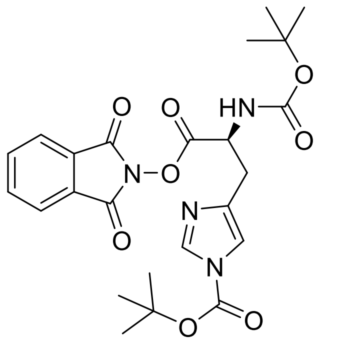

tert-butyl (*S*)-4-(2-((*tert*-butoxycarbonyl)amino)-3-((1,3-dioxoisoindolin-2-yl)oxy)-3-oxopropyl)-1*H*-imidazole-1-carboxylate
Chemical Formula: $C_{24}H_{28}N_4O_8$
Molecular Weight: 500.51

Prepared according to General Procedure B with N$^{\alpha}$,N$^{\tau}$-bis(*tert*-butoxycarbonyl)-*L*-histidine **2f'** (1.78 g, 5.00 mmol, 1.00 eq.), EDC hydrochloride (1.15 g, 6.00 mmol, 1.20 eq.), 4-dimethylaminopyridine (60 mg, 0.50 mmol, 10 mol%) and *N*-hydroxyphthalimide (0.815 g, 5.00 mmol, 1.00 eq.) in CH_2Cl_2 (50 mL) to afford the desired product as a white solid (1.8 g, 72%).

Rotamer ratio determined from crude ^{1}H NMR – 1.0:0.35.

Major rotamer:

^{1}H NMR (400 MHz, CDCl$_3$) δ 8.05 (br s, 1H), 7.92 – 7.83 (m, 2H), 7.82 – 7.74 (m, 2H), 7.45 (br s, 1H), 6.04 (d, *J* = 8.4 Hz, 1H), 5.04 – 4.95 (m, 1H), 3.34 – 3.17 (m, 2H), 1.62 (s, 9H), 1.44 (s, 9H).

^{13}C{^{1}H} NMR (126 MHz, CDCl$_3$) δ 168.5, 161.4, 155.1, 146.9, 137.1, 134.7, 134.0, 128.9, 124.0, 115.9, 85.6, 80.3, 52.2, 30.1, 28.3, 27.9.

Minor rotamer:

^{1}H NMR (400 MHz, CDCl$_3$) δ 8.03 (br s, 1H), 7.92 – 7.83 (m, 2H), 7.82 – 7.74 (m, 2H), 7.43 (br s, 1H), 5.77 – 5.71 (m, 1H), 4.81 – 4.73 (m, 1H), 3.34 – 3.17 (m, 2H), 1.62 (s, 9H), 1.44 (s, 9H).

^{13}C{^{1}H} NMR (126 MHz, CDCl$_3$) δ 168.5, 161.4, 155.1, 146.9, 137.1, 134.7, 134.0, 128.9, 124.0, 115.9, 85.6, 80.3, 53.5, 30.1, 28.4, 28.0.

IR (solid): 1744, 1717, 1389, 1250, 1152, 1011 cm^{-1}.

HRMS (ESI): *m/z* calculated for [M + H]$^+$ ($C_{24}H_{29}N_4O_8$)$^+$: 501.1980; found 501.1982.

1,3-Dioxoisoindolin-2-yl 3-((*tert*-butoxycarbonyl)amino)-3-(*p*-tolyl)propanoate (2g)

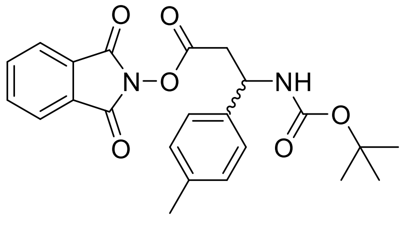

1,3-dioxoisoindolin-2-yl 3-((*tert*-butoxycarbonyl)amino)-3-(*p*-tolyl)propanoate
Chemical Formula: $C_{23}H_{24}N_2O_6$
Molecular Weight: 424.45

Prepared according to General Procedure B with 3-((*tert*-butoxycarbonyl)amino)-3-(*p*-tolyl)propanoic acid **2g'** (1.40 g, 5.00 mmol, 1.00 eq.), EDC hydrochloride (1.15 g, 6.00 mmol, 1.20 eq.), 4-dimethylaminopyridine (60 mg, 0.50 mmol, 10 mol%) and *N*-hydroxyphthalimide (0.815 g, 5.00 mmol, 1.00 eq.) in CH_2Cl_2 (50 mL) to afford the desired product as a white solid (1.3 g, 60 %).

^{1}H NMR (400 MHz, $CDCl_3$) δ 7.91 – 7.85 (m, 2H), 7.83 – 7.76 (m, 2H), 7.28 – 7.25 (m, 2H), 7.23 – 7.18 (m, 2H), 5.32 (br s, 1H), 5.24 (br s, 1H), 3.38 – 3.13 (m, 2H), 2.36 (s, 3H), 1.47 (s, 9H).

^{13}C{^{1}H} NMR (126 MHz, $CDCl_3$) δ 167.0, 161.7, 154.9, 137.7, 136.8, 134.8, 129.6, 128.9, 126.2, 14.0, 80.0, 50.7, 37.5, 28.4, 21.1.

IR (solid): 2976, 2918, 1740, 1684, 1522, 1364, 1074, 818 cm^{-1}.

HRMS (ESI): *m/z* calculated for $[M + Na]^+$ $(C_{23}H_{24}N_2O_6Na)^+$: 447.1527; found 447.1523.

1,3-Dioxoisoindolin-2-yl 3-((*tert*-butoxycarbonyl)amino)propanoate (2h)

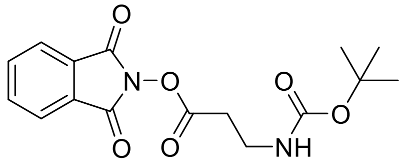

1,3-dioxoisoindolin-2-yl 3-((*tert*-butoxycarbonyl)amino)propanoate
Chemical Formula: $C_{16}H_{18}N_2O_6$
Molecular Weight: 334.33

Prepared according to General Procedure B with 3-((*tert*-butoxycarbonyl)amino)propanoic acid, (0.378 g, 2.00 mmol, 1.00 eq.), EDC hydrochloride (1.15 g, 2.40 mmol, 1.20 eq.), 4-dimethylaminopyridine (61 mg, 0.20 mmol, 10 mol%) and *N*-hydroxyphthalimide (0.816 g, 2.00 mmol, 1.00 eq.) in CH_2Cl_2 (20 mL) to afford the desired product as a white solid (0.53 g, 79%).

^{1}H NMR (400 MHz, $CDCl_3$) δ 7.94 – 7.86 (m, 2H), 7.84 – 7.77 (m, 2H), 5.14 (br s, 1H), 3.56 (q, J = 6.02 Hz, 2H), 2.91 (t, J = 6.00 Hz, 2H), 1.45 (s, 9H).

Spectroscopic data in agreement with the literature.[15]

1,3-Dioxoisoindolin-2-yl (*R*)-3-((*tert*-butoxycarbonyl)amino)butanoate (2i)

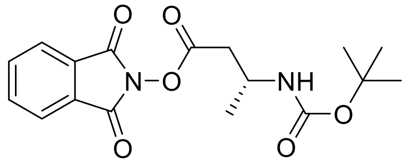

1,3-dioxoisoindolin-2-yl (*R*)-3-((*tert*-butoxycarbonyl)amino)butanoate
Chemical Formula: $C_{17}H_{20}N_2O_6$
Molecular Weight: 348.36

Prepared according to General Procedure B with (*R*)-3-((*tert*-butoxycarbonyl)amino)butanoic acid **4i'** (1.02 g, 5.00 mmol, 1.00 eq.), EDC hydrochloride (1.15 g, 6.00 mmol, 1.20 eq.), 4-dimethylaminopyridine (60 mg, 0.50 mmol, 10 mol%) and *N*-hydroxyphthalimide (0.815 g, 5.00 mmol, 1.00 eq.) in CH_2Cl_2 (50 mL) to afford the desired product as a white solid (1.5 g, 87%).

^{1}H NMR (400 MHz, CDCl$_3$) δ 7.91 – 7.85 (m, 2H), 7.82 – 7.75 (m, 2H), 4.93 (d, *J* = 8.5 Hz, 1H), 4.17 (br s, 1H), 2.90 (d, *J* = 5.0 Hz, 2H), 1.44 (s, 9H), 1.33 (d, *J* = 6.8 Hz, 3H).

Spectroscopic data in agreement with the literature.[16]

1,3-Dioxoisoindolin-2-yl 2-((*tert*-butoxycarbonyl)amino)cyclopentane-1-carboxylate (2j)

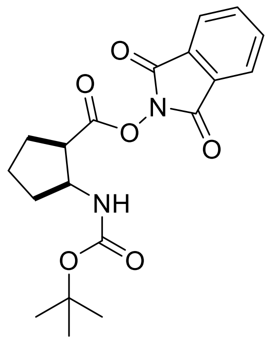

1,3-dioxoisoindolin-2-yl 2-((*tert*-butoxycarbonyl)amino)cyclopentane-1-carboxylate
Chemical Formula: $C_{19}H_{22}N_2O_6$
Molecular Weight: 374.39

Prepared according to General Procedure B with 2-((*tert*-butoxycarbonyl)amino)cyclopentane-1-carboxylic acid **4j'** (0.867 g, 3.78 mmol, 1.00 eq.), EDC hydrochloride (0.870 g, 4.54 mmol, 1.20 eq.), 4-dimethylaminopyridine (46 mg, 0.38 mmol, 10 mol%) and *N*-hydroxyphthalimide (0.617 g, 3.78 mmol, 1.00 eq.) in CH_2Cl_2 (40 mL) to afford the desired product as a white solid (0.95 g, 67%) as a single diastereomer.

^{1}H NMR (500 MHz, CDCl$_3$) δ 7.92 – 7.87 (m, 2H), 7.82 – 7.77 (m, 2H), 5.33 (d, *J* = 9.4 Hz, 1H), 4.42 – 4.31 (m, 1H), 3.43 (td, *J* = 7.9, 3.5 Hz, 1H), 2.23 – 2.15 (m, 1H), 2.10 – 2.00 (m, 2H), 1.97 – 1.86 (m, 1H), 1.75 – 1.61 (m, 2H), 1.45 (s, 9H).

^{13}C{^{1}H} NMR (126 MHz, CDCl$_3$) δ 170.7, 162.2, 155.8, 135.0, 129.1, 124.2, 79.7, 54.7, 44.4, 30.7, 28.5, 27.5, 21.6.

IR (solid): 3333, 1740, 1680, 1522, 1161, 1084, 878, 785 cm^{-1}.

HRMS (ESI): *m/z* calculated for $[M + H]^+$ $(C_{19}H_{23}N_2O_6)^+$: 375.1551; found 375.1554.

1,3-Dioxoisoindolin-2-yl (*tert*-butoxycarbonyl)-*L*-phenylalaninate (2k)

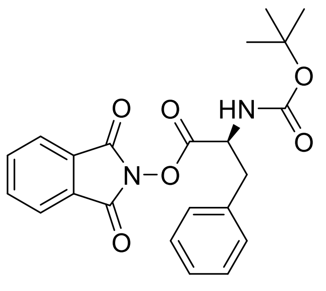

1,3-dioxoisoindolin-2-yl (*tert*-butoxycarbonyl)-*L*-phenylalaninate
Chemical Formula: $C_{22}H_{22}N_2O_6$
Molecular Weight: 410.43

Prepared according to General Procedure B with (*tert*-butoxycarbonyl)-*L*-phenylalanine (1.33 g, 5.00 mmol, 1.00 eq.), EDC hydrochloride (1.15 g, 6.00 mmol, 1.20 eq.), 4-dimethylaminopyridine (60 mg, 0.50 mmol, 10 mol%) and *N*-hydroxyphthalimide (0.815 g, 5.00 mmol, 1.00 eq.) in CH_2Cl_2 (50 mL) to afford the desired product as a white solid (0.23 g, 65%).

^{1}H NMR (500 MHz, CDCl$_3$) δ 7.94 – 7.88 (m, 2H), 7.85 – 7.77 (m, 2H), 7.39 – 7.16 (m, 5H), 5.08 – 4.51 (m, 2H), 3.40 – 2.70 (m, 2H), 1.44 (s, 9H).

Spectroscopic data in agreement with the literature.[17]

1,3-Dioxoisoindolin-2-yl acetylglycinate (2l)

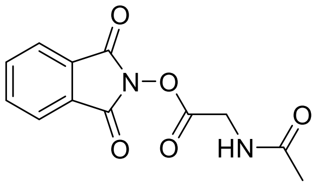

1,3-dioxoisoindolin-2-yl acetylglycinate
Chemical Formula: $C_{12}H_{10}N_2O_5$
Molecular Weight: 262.22

Prepared according to General Procedure B with acetylglycine **4l'**, (0.234 g, 2.00 mmol, 1.00 eq.), EDC hydrochloride (0.460 g, 2.40 mmol, 1.20 eq.), 4-dimethylaminopyridine (24 mg, 0.20 mmol, 10 mol%) and *N*-hydroxyphthalimide (0.326 g, 2.00 mmol, 1.00 eq.) in CH_2Cl_2 (20 mL) to afford the desired product as a white solid (0.36 g, 69%).

^{1}H NMR (500 MHz, CDCl$_3$) δ 7.94 – 7.86 (m, 2H), 7.84 – 7.77 (m, 2H), 6.01 (br s, 1H), 4.50 (d, *J* = 5.30 Hz, 2H), 2.09 (s, 3H).

^{13}C{^{1}H} NMR (126 MHz, CDCl$_3$) δ 170.4, 167.1, 161.6, 135.1, 128.9, 124.3, 39.3, 23.0.

IR (solid): 3291, 1796, 1734, 1647, 1377, 1196, 1136, 1082, 874 cm^{-1}.

HRMS (ESI): *m/z* calculated for [M + Na]$^+$ ($C_{12}H_{10}N_2O_5Na$)$^+$: 285.0482; found 285.0492.

Photocoupling

***tert*-Butyl cinnamylcarbamate (3)**

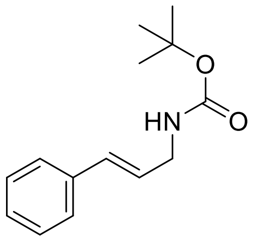

tert-butyl cinnamylcarbamate
Chemical Formula: $C_{14}H_{19}NO_2$
Molecular Weight: 233.31

Prepared according to General Procedure C with 1,3-dioxoisoindolin-2-yl (*tert*-butoxycarbonyl)glycinate **2a** (64.1 mg, 0.200 mmol, 1.00 eq.), (*E*)-2-phenylvinylboronic acid (59.2 mg, 0.400 mmol, 2.00 eq.), 4CzIPN (1.6 mg, 2.0 µmol, 1.0 mol%) and triphenylamine (49 mg, 0.020 mmol, 10 mol%) in DMSO (1 mL). The crude was purified by column chromatography (basified silica, 0 to 10% ethyl acetate in hexane) to afford the product as a white solid (32 mg, 69%).

^{1}H NMR (400 MHz, CDCl$_3$) δ 7.37 – 7.33 (m, 2H), 7.33 – 7.27 (m, 2H), 7.25 – 7.20 (m, 1H), 6.50 (dt, *J* = 16.0, *J* = 1.4, 1H), 6.24 – 6.15 (m, 1H), 4.68 (br s, 1H), 3.91 (t, *J* = 5.7 Hz, 2H), 1.47 (s, 9H).

^{13}C NMR (126 MHz, CDCl$_3$) δ 155.8, 136.7, 131.5, 128.6, 127.6, 126.4, 79.5, 42.8, 28.4.

Spectroscopic data in agreement with the literature.[18]

***tert*-Butyl (*E*)-(4-phenylbut-3-en-2-yl)carbamate (4)**

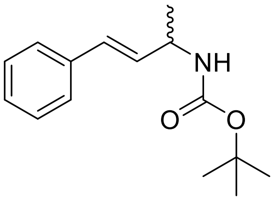

tert-butyl (*E*)-(4-phenylbut-3-en-2-yl)carbamate
Chemical Formula: $C_{15}H_{21}NO_2$
Molecular Weight: 247.34

*Reaction performed in three vials (0.20 mmol scale each) and worked up and purified together.

Prepared according to General Procedure C with 1,3 dioxoisoindolin-2-yl (*tert*-butoxycarbonyl)alaninate **2d** (67 mg, 0.20 mmol, 1.0 eq.), phenylvinylboronic acid (59 mg, 0.40 mmol, 2.0 eq.), triphenylamine (4.9 mg, 0.020 mmol, 10 mol%) and 4CzIPN (1.6 mg, 2.0 µmol, 1.0 mol%) in DMSO (1 mL). The crude was purified by column chromatography (basified silica, 0 to 15% , ethyl acetate in cyclohexane) to afford the desired product as a white solid (73 mg, 49%).

^{1}H NMR (400 MHz, CDCl$_3$) δ 7.41 – 7.36 (m, 2H), 7.36 – 7.29 (m, 2H), 7.28 – 7.21 (m, 1H), 6.52 (d, *J* = 16.0 Hz, 1H), 6.18 (dd, *J* = 15.9, 5.7 Hz, 1H), 4.59 (s, 1H), 4.43 (br s, 1H), 1.49 (s, 9H), 1.33 (d, *J* = 6.9 Hz, 3H).

Spectroscopic data in agreement with the literature.[18]

(*E*)-*N*-cinnamylacetamide (5)

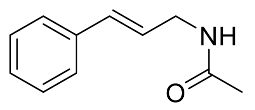

N-cinnamylacetamide
Chemical Formula: $C_{11}H_{13}NO$
Molecular Weight: 175.23

Prepared according to General Procedure C with 1,3-dioxoisoindolin-2-yl acetylglycinate **2l** (52.4 mg, 0.200 mmol, 1.00 eq.), phenylvinylboronic acid (59 mg, 0.40 mmol, 2.0 eq.), triphenylamine (4.9 mg, 0.020 mmol, 10 mol%) and 4CzIPN (1.6 mg, 2.0 μmol, 1.0 mol%) in DMSO (1 mL). The crude was purified by column chromatography (basified silica, 0 to 15% ethyl acetate in cyclohexane) to afford the desired product as a white solid (73 mg, 54%).

^{1}H NMR (500 MHz, $CDCl_3$) δ 7.40 – 7.30 (m, 4H), 7.28 – 7.23 (m, 1H), 6.55 (dt, *J* = 15.8, 1.6 Hz, 1H), 6.22 (dt, *J* = 15.8, 6.4 Hz, 1H), 5.63 (br s, 1H), 4.06 (td, *J* = 6.1, 1.5 Hz, 2H), 2.05 (s, 3H).

^{13}C NMR (126 MHz, $CDCl_3$) δ 169.9, 136.5, 132.3, 128.6, 127.8, 126.4, 125.5, 41.7, 23.4.

Spectroscopic data in agreement with the literature.[19]

***tert*-Butyl (*E*)-methyl(4-phenylbut-3-en-2-yl)carbamate (6)**

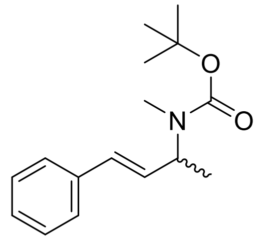

tert-butyl (*E*)-methyl(4-phenylbut-3-en-2-yl)carbamate
Chemical Formula: $C_{16}H_{23}NO_2$
Molecular Weight: 261.37

Prepared according to General Procedure C with 1,3-dioxoisoindolin-2-yl *N*-(*tert*-butoxycarbonyl)-*N*-methylalaninate **2c** (69.7 mg, 0.200 mmol, 1.00 eq.), *(E)*-2-phenylvinylboronic acid (59.2 mg, 0.400 mmol, 2.00 eq.), 4CzIPN (1.6 mg, 2.0 μmol, 1.0 mol%) and triphenylamine (4.9 mg, 20 μmol, 10 mol%) in DMSO (1 mL). The crude product was purified by column chromatography (basified silica, 0 to 5% ethyl acetate in hexane) to afford the product as a colourless oil (21 mg, 40%).

^{1}H NMR (400 MHz, $CDCl_3$) δ 7.39 – 7.35 (m, 2H), 7.34 – 7.28 (m, 2H), 7.26 – 7.21 (m, 1H), 6.43 (d, *J* = 16.1 Hz, 1H), 6.18 (dd, *J* = 16.1, 4.7 Hz, 1H), 4.96 (br s, 1H), 2.73 (s, 3H), 1.49 (s, 9H), 1.32 (d, *J* = 6.9 Hz, 3H).

Spectroscopic data in agreement with the literature.[20]

***tert*-Butyl (*E*)-(3-(4-chlorophenyl)allyl)carbamate (7)**

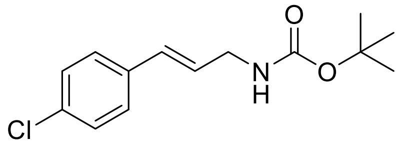

tert-butyl (*E*)-(3-(4-chlorophenyl)allyl)carbamate
Chemical Formula: $C_{14}H_{18}ClNO_2$
Molecular Weight: 267.75

Prepared according to General Procedure C with 1,3 dioxoisoindolin-2-yl (*tert*-butoxycarbonyl)glycinate **2a** (64 mg, 0.20 mmol, 1.0 eq.), *p*-chloro-phenylvinylboronic acid (73 mg, 0.40 mmol, 2.0 eq.), triphenylamine (4.9 mg, 0.020 mmol, 10 mol%) and 4CzIPN (1.6 mg, 2.0 µmol, 1.0 mol%) in DMSO (1 mL). The crude was purified by column chromatography (basified silica, 0 to 5% ethyl acetate in hexane) to afford the desired product as a white solid (28 mg, 52%).

^{1}H NMR (400 MHz, $CDCl_3$) δ 7.27 (s, 4H), 6.45 (dt, *J* = 15.8, 1.6 Hz, 1H), 6.17 (dt, *J* = 15.8, 6.0 Hz, 1H), 4.67 (s, 1H), 3.90 (d, *J* = 6.4 Hz, 2H), 1.46 (s, 9H).

Spectroscopic data in agreement with the literature.[21]

***tert*-Butyl (*E*)-(3-(4-methoxyphenyl)allyl)carbamate (8)**

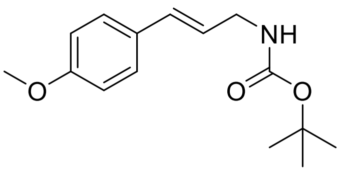

tert-butyl (*E*)-(3-(4-methoxyphenyl)allyl)carbamate
Chemical Formula: $C_{15}H_{21}NO_3$
Molecular Weight: 263.34

Prepared according to General Procedure C with 1,3 dioxoisoindolin-2-yl (*tert*-butoxycarbonyl)glycinate **2a** (64 mg, 0.20 mmol, 1.0 eq.), *p*-methoxyphenylvinylboronic acid (72 mg, 0.40 mmol, 2.0 eq.), triphenylamine (4.9 mg, 0.020 mmol, 10 mol%) and 4CzIPN (1.6 mg, 2.0 µmol, 1.0 mol%) in DMSO (1 mL). The crude was purified by column chromatography (basified silica, 0 to 15% ethyl acetate in cyclohexane) to afford the desired product as a white solid (42 mg, 26%).

^{1}H NMR (400 MHz, $CDCl_3$) δ 7.35 – 7.25 (m, 2H), 6.90 – 6.82 (m, 2H), 6.46 (d, *J* = 15.7 Hz, 1H), 6.07 (dt, *J* = 14.9, 6.4 Hz, 1H), 4.70 (s, 1H), 3.96 – 3.85 (m, 2H), 3.82 (s, 3H), 1.48 (s, 9H).

Spectroscopic data in agreement with the literature.[22]

tert-Butyl (_E_)-(1,4-diphenylbut-3-en-2-yl)carbamate (10)

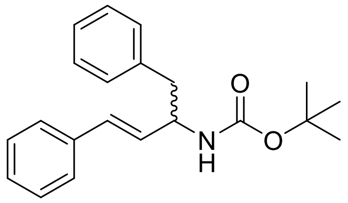

tert-butyl (*E*)-(1,4-diphenylbut-3-en-2-yl)carbamate
Chemical Formula: $C_{21}H_{25}NO_2$
Molecular Weight: 323.44

Prepared according to General Procedure C with 1,3-dioxoisoindolin-2-yl (*tert*-butoxycarbonyl)-*D*-phenylalaninate **2k** (82 mg, 0.20 mmol, 1.0 eq.), phenylvinylboronic acid (59 mg, 0.40 mmol, 2.0 eq.), triphenylamine (4.9 mg, 0.020 mmol, 10 mol%) and 4CzIPN (1.6 mg, 2.0 µmol, 1.0 mol%) in DMSO (1 mL). The crude was purified by column chromatography (basified silica, 0 to 5% ethyl acetate in cyclohexane, then again 0 to 2.5% ethyl acetate in hexane) to afford the desired product as a white solid (26 mg, 40%).

^{1}H NMR (500 MHz, $CDCl_3$) δ 7.34 – 7.27 (m, 6H), 7.25 – 7.19 (m, 4H), 6.45 (d, *J* = 15.9 Hz, 1H), 6.18 – 6.10 (m, 1H), 4.58 (s, 2H), 2.99 – 2.90 (m, 2H), 1.45 (s, 9H).

Spectroscopic data in agreement with the literature.[23]

(_E_)-_N_-(1,4-Diphenylbut-3-en-2-yl)acetamide (11)

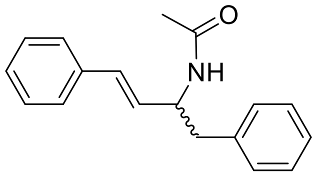

(*E*)-*N*-(1,4-diphenylbut-3-en-2-yl)acetamide
Chemical Formula: $C_{18}H_{19}NO$
Molecular Weight: 265.36

Prepared according to General Procedure C with 1,3-dioxoisoindolin-2-yl acetyl-*D*-phenylalanine **2e** (70 mg, 0.20 mmol, 1.0 eq.), phenylvinylboronic acid (59 mg, 0.40 mmol, 2.0 eq.), triphenylamine (4.9 mg, 0.020 mmol, 10 mol%) and 4CzIPN (1.6 mg, 2.0 µmol, 1.0 mol%) in DMSO (1 mL). The crude was purified by column chromatography (basified silica, 0 to 40% ethyl acetate in hexane) to afford the desired product as a white solid (21 mg, 38%).

^{1}H NMR (400 MHz, $CDCl_3$) δ 7.36 – 7.16 (m, 10H), 6.46 (dd, *J* = 15.9, 1.5 Hz, 1H), 6.14 (dd, *J* = 15.9, 6.3 Hz, 1H), 5.52 (d, *J* = 8.5 Hz, 1H), 5.00 – 4.90 (m, 1H), 2.97 (d, *J* = 6.3 Hz, 2H), 1.98 (s, 3H).

^{13}C{^{1}H} NMR (126 MHz, $CDCl_3$) δ 169.4, 137.2, 136.7, 130.9, 129.6, 129.1, 128.7, 128.6, 127.8, 126.8, 126.5, 51.8, 41.5, 23.6.

IR (solid): 3294, 3026, 2920, 1541, 1541, 1368. 964 cm^{-1}.

HRMS (ESI): *m/z* calculated for $[M + H]^+$ $(C_{18}H_{20}NO)^+$: 266.1539; found 266.1539.

tert-Butyl cinnamyl(methyl)carbamate (12)

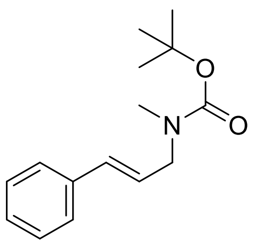

tert-butyl cinnamyl(methyl)carbamate
Chemical Formula: $C_{15}H_{21}NO_2$
Molecular Weight: 247.34

Prepared according to General Procedure C with 1,3-dioxoisoindolin-2-yl *N*-(*tert*-butoxycarbonyl)-*N*-methylglycinate **2b** (66.9 mg, 0.200 mmol, 1.00 eq.), (*E*)-2-phenylvinylboronic acid (59.2 mg, 0.400 mmol, 2.00 eq.), 4CzIPN (1.6 mg, 2.0 µmol, 1.0 mol%) and triphenylamine (4.9 mg, 0.020 mmol, 10 mol%) in DMSO (1 mL). The crude product was purified by column chromatography (basified silica, 0 to 10% ethyl acetate in hexane) to afford the product as a colourless oil (32 mg, 64%).

^{1}H NMR (500 MHz, $CDCl_3$) δ 7.42 – 7.37 (m, 2H), 7.36 – 7.31 (m, 2H), 7.29 – 7.23 (m, 1H), 6.49 (d, *J* = 15.9 Hz, 1H), 6.23 – 6.14 (m, 1H), 4.00 (br s, 2H), 2.89 (s, 3H), 1.51 (s, 9H).

^{13}C NMR (126 MHz, $CDCl_3$) δ 155.8, 136.8, 128.6, 127.6, 126.4, 79.6, 65.9, 51.1, 33.8, 28.5, 15.3.

Spectroscopic data in agreement with the literature.[24]

tert-Butyl (*E*)-(3-(4-fluorophenyl)allyl)(methyl)carbamate (13)

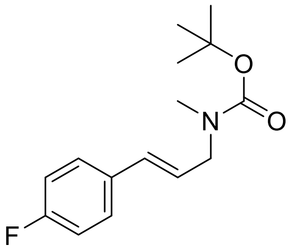

tert-butyl (*E*)-(3-(4-fluorophenyl)allyl)(methyl)carbamate
Chemical Formula: $C_{15}H_{20}FNO_2$
Molecular Weight: 265.33

Prepared according to General Procedure C with 1,3-dioxoisoindolin-2-yl *N*-(*tert*-butoxycarbonyl)-*N*-methylglycinate **2b** (66.9 mg, 0.200 mmol, 1.00 eq.), (*E*)-(4-fluorostyryl)boronic acid (66.4 mg, 0.400 mmol, 2.00 eq.), 4CzIPN (1.6 mg, 2.0 µmol, 1.0 mol%) and triphenylamine (4.9 mg, 0.020 mmol, 10 mol%) in DMSO (1 mL). The crude product was purified by column chromatography (basified silica, 0 to 10% ethyl acetate in hexane) to afford the product as a white solid (31 mg, 59%).

^{1}H NMR (500 MHz, $CDCl_3$) δ 7.36 – 7.29 (m, 2H), 7.04 – 6.95 (m, 2H), 6.41 (d, *J* = 15.6 Hz, 1H), 6.10 – 6.00 (m, 1H), 3.96 (br s, 2H), 2.86 (s, 3H), 1.47 (s, 9H).

^{13}C{^{1}H} NMR (126 MHz, $CDCl_3$) δ 161.4 (d, $^1J_{CF}$ = 246 Hz), 155.9, 133.1, 130.6, 127.9 (d, $^3J_{CF}$ = 7.43 Hz), 125.2, 115.7 (d, $^2J_{CF}$ = 20.7 Hz), 79.7, 51.1, 33.9, 28.6.

^{19}F NMR (471 MHz, $CDCl_3$) δ −114.51.

IR (solid): 2976, 2928, 1696, 1601, 1508, 1391, 1366, 1227, 1142, 843 cm^{-1}.

HRMS (ESI): *m/z* calculated for $[M + Na]^+$ $(C_{15}H_{20}FNO_2Na)^+$: 288.1370; found 288.1369.

tert-Butyl (_E_)-methyl(3-(pyridine-2-yl)allyl)carbamate (14)

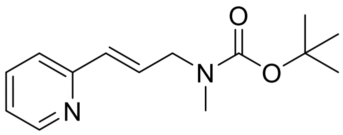

tert-butyl (*E*)-methyl(3-(pyridin-2-yl)allyl)carbamate
Chemical Formula: $C_{14}H_{20}N_2O_2$
Molecular Weight: 248.33

Prepared according to General Procedure C with 1,3-dioxoisoindolin-2-yl *N*-(*tert*-butoxycarbonyl) -*N*-methylglycinate **2b** (67 mg, 0.20 mmol, 1.0 eq.), (*E*)-(2-(pyridin-2-yl)vinyl)boronic acid (60 mg, 0.40 mmol, 2.0 eq.), triphenylamine (4.9 mg, 0.020 mmol, 10 mol%) and 4CzIPN (1.6 mg, 2.0 µmol, 1.0 mol%) in DMSO (1 mL). The crude was purified by column chromatography (basified silica, 0 to 50% ethyl acetate in hexane). The isolated mixture of product and phthalimide was treated with aq. NaOH (1 M, 3 mL) and extracted with diethyl ether (2 × 5 mL) to afford the desired product as a white solid (17 mg, 35%).

^{1}H NMR (500 MHz, CDCl$_3$) δ 8.55 (d, J = 4.8 Hz, 1H), 7.62 (td, J = 7.6, 1.8 Hz, 1H), 7.28 (br s, 1H), 7.13 (dd, J = 7.5, 4.8 Hz, 1H), 6.71 – 6.60 (m, 1H), 6.60 – 6.48 (m, 1H), 4.16 – 3.91 (m, 2H), 2.88 (s, 3H), 1.47 (s, 9H).

^{13}C{^{1}H} NMR (126 MHz, CDCl$_3$) δ 164.9, 155.1, 149.6, 136.5, 130.1, 122.2, 121.6, 79.6, 50.8, 34.0, 28.5.

IR (solid): 2972, 2928, 1684, 1391, 1366, 1142 cm^{-1}.

HRMS (ESI): *m/z* calculated for $[M + H]^+$ $(C_{14}H_{21}N_2O_2)^+$: 249.1598; found 249.1602.

tert-Butyl (_E_)-4-(2-((_tert_-butoxycarbonyl)amino)-4-phenylbut-3-en-1-yl)-1_H_-imidazole-1-carboxylate (15)

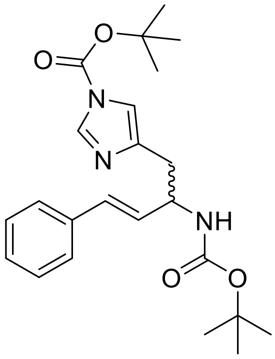

tert-butyl (*E*)-4-(2-((*tert*-butoxycarbonyl)amino)-4-phenylbut-3-en-1-yl)-1*H*-imidazole-1-carboxylate
Chemical Formula: $C_{23}H_{31}N_3O_4$
Molecular Weight: 413.52

Prepared according to General Procedure C with *tert*-butyl (*S*)-4-(2-((*tert*-butoxycarbonyl)amino)-3-((1,3-dioxoisoindolin-2-yl)oxy)-3 oxopropyl)-1*H*-imidazole-1-carboxylate **2f** (0.10 g, 0.20 mmol, 1.0 eq.), phenylvinylboronic acid (59 mg, 0.40 mmol, 2.0 eq.), *N*,*N*-dimethylaniline (2.5 µL, 0.040 mmol, 2 mol%) and $Ru(bpy)_3(PF_6)_2$ (1.7 mg, 2.0 µmol, 1.0 mol%) in DMSO (1 mL). The crude was purified by column chromatography (basified silica, 0 to 20% ethyl acetate in cyclohexane), washed with aq. NaOH (1 M, 3 mL), dried over Na_2SO_4 and filtered. The solvent was removed *in vacuo* to afford the desired product as a yellow oil (26 mg, 30%).

Major rotamer:

^{1}H NMR (500 MHz, CDCl$_3$) δ 8.01 (d, *J* = 1.3 Hz, 1H), 7.36 – 7.26 (m, 4H), 7.25 – 7.18 (m, 1H), 7.15 (s, 1H), 6.51 (d, *J* = 15.9 Hz, 1H), 6.14 (dd, *J* = 15.9, 6.1 Hz, 1H), 5.48 (br s), 4.60 (s, 1H), 2.89 – 2.76 (m, 2H), 1.60 (s, 9H), 1.43 (s, 9H).

^{13}C{^{1}H} NMR (126 MHz, CDCl$_3$) δ 155.4, 147.0, 139.8, 136.9, 136.7, 130.2, 129.9, 128.5, 127.4, 126.5, 114.4, 85.5, 79.4, 51.9, 33.6, 28.4, 27.9.

Minor rotamer:

^{1}H NMR (500 MHz, CDCl$_3$) δ 7.98 (d, *J* = 1.3 Hz, 1H), 7.36 – 7.26 (m, 4H), 7.25 – 7.18 (m, 1H), 7.07 (s, 1H), 6.47 (d, *J* = 11.8 Hz, 1H), 5.54 (dd, *J* = 11.7, 9.2 Hz, 1H), 5.29 (br s), 4.92 (s, 1H), 2.99 – 2.91 (m, 2H), 1.60 (s, 9H), 1.44 (s, 9H).

^{13}C{^{1}H} NMR (126 MHz, CDCl$_3$) δ 155.4, 147.0, 139.9, 139.8, 136.6, 132.1, 130.0, 128.7, 128.4, 127.1, 114.6, 85.4, 79.7, 48.2, 33.8, 28.4, 27.9.

IR (film): 3356, 2978, 2930, 1755, 1697, 1495, 1368, 1250, 1153, 1011 cm^{-1}.

HRMS (ESI): *m/z* calculated for [M + Na]$^+$ ($C_{23}H_{31}N_3O_4Na_1$)$^+$: 436.2207; found 436.2215.

Methyl (*E*)-3-(3-((*tert*-butoxycarbonyl)amino)prop-1-1en-1-yl)benzoate (16)

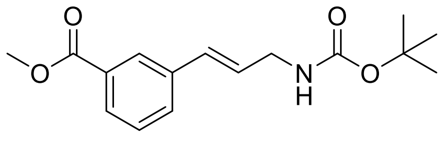

methyl (*E*)-3-(3-((*tert*-butoxycarbonyl)amino)prop-1-en-1-yl)benzoate
Chemical Formula: $C_{16}H_{21}NO_4$
Molecular Weight: 291.35

Prepared according to General Procedure C with 1,3-dioxoisoindolin-2-yl (*tert*-butoxycarbonyl)glycinate **2a** (64 mg, 0.20 mmol, 1.0 eq.), (*E*)-(3-(methoxycarbonyl)styryl)boronic acid (82 mg, 0.40 mmol, 2.0 eq.), triphenylamine (4.9 mg, 0.020 mmol, 10 mol%) and 4CzIPN (1.6 mg, 2.0 µmol, 1.0 mol%) in DMSO (1 mL). The crude was purified by column chromatography (basified silica, 0 to 5% ethyl acetate in hexane) to afford the desired product as a white solid (38 mg, 63%).

^{1}H NMR (500 MHz, CDCl$_3$) δ 8.03 (t, *J* = 1.8 Hz, 1H), 7.89 (dt, *J* = 7.8, 1.4 Hz, 1H), 7.52 (dt, *J* = 7.8, 1.6 Hz, 1H), 7.37 (t, *J* = 7.7 Hz, 1H), 6.53 (d, *J* = 15.9, 1H), 6.28 (dt, *J* = 15.9, 6.0 Hz, 1H), 4.71 (s, 1H), 3.96 – 3.88 (m, 5H), 1.46 (s, 9H).

^{13}C{^{1}H} NMR (126 MHz, CDCl$_3$) δ 167.0, 155.8, 137.1, 130.7, 130.5, 130.3, 128.6 (two carbons), 127.9, 127.5, 79.6, 52.2, 42.6, 28.4.

IR (solid): 3364, 2920, 2853, 1717, 1680, 1506, 1242, 1161 cm^{-1}.

HRMS (ESI): *m/z* calculated for [M + H]$^+$ ($C_{16}H_{22}N_1O_4$)$^+$: 292.1543; found 292.1549.

***tert*-Butyl (*E*)-(4-phenylbut-3-en-1-yl)carbamate (17)**

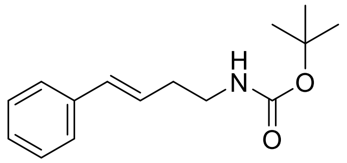

tert-butyl (*E*)-(4-phenylbut-3-en-1-yl)carbamate
Chemical Formula: $C_{15}H_{21}NO_2$
Molecular Weight: 247.34

Prepared according to General Procedure C with 1,3-dioxoisoindolin-2-yl *tert*-butyl (*E*)-(4-phenylbut-3-en-1-yl)carbamate **2h** (66.9 mg, 0.200 mmol, 1.00 eq.), (*E*)-2-phenylvinylboronic acid (59.2 mg, 0.400 mmol, 2.00 eq.), 4CzIPN (1.6 mg, 2.0 μmol, 1.0 mol%) and triphenylamine (4.9 mg, 20 μmol, 10 mol%) in DMSO (1 mL). The crude product was purified by column (basified silica, 0 to 10% ethyl acetate in hexane) to afford the product as a colourless oil (37 mg, 74%).

^{1}H NMR (400 MHz, $CDCl_3$) δ 7.40 – 7.35 (m, 2H), 7.35 – 7.29 (m, 2H), 7.27 – 7.21 (m, 1H), 6.48 (d, *J* = 15.8 Hz, 1H), 6.23 – 6.12 (m, 1H), 4.64 (br s, 1H), 3.33 – 3.25 (m, 2H), 2.46 – 2.39 (m, 2H), 1.46 (s, 9H).

^{13}C NMR (126 MHz, $CDCl_3$) δ 156.0, 137.3, 132.3, 128.6, 127.3, 127.0, 126.1, 79.2, 77.3, 77.1, 76.8, 40.1, 33.6, 28.4.

Spectroscopic data in agreement with the literature.[25]

***tert*-Butyl (*R*,*E*)-(5-(4-methoxyphenyl)pent-4-en-2-yl)carbamate (19)**

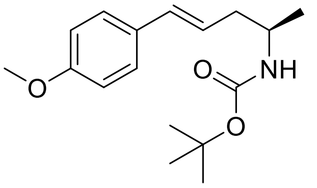

tert-butyl (*R*,*E*)-(5-(4-methoxyphenyl)pent-4-en-2-yl)carbamate
Chemical Formula: $C_{17}H_{25}NO_3$
Molecular Weight: 291.39

Prepared according to General Procedure C with 1,3-dioxoisoindolin-2-yl *N*-(*tert*-butoxycarbonyl) -*N*-methylglycinate **2i** (69.7 mg, 0.200 mmol, 1.00 eq.), *trans*-2-(4-methoxyphenyl)vinylboronic acid (71.2 mg, 0.400 mmol, 2.00 eq.), triphenylamine (4.9 mg, 0.020 mmol, 10 mol%) and 4CzIPN (1.6 mg, 2.0 μmol, 1.0 mol%) in DMSO (1 mL). The crude was purified by column chromatography (basified silica, 0 to 5% ethyl acetate in hexane) to afford the desired product as a colourless oil (24 mg, 40%).

^{1}H NMR (500 MHz, $CDCl_3$) δ 7.30 – 7.27 (m, 2H), 6.87 – 6.81 (m, 2H), 6.37 (d, *J* = 15.7 Hz, 1H), 6.03 (dt, *J* = 15.3, 7.3 Hz, 1H), 4.41 (br s, 1H), 3.83 – 3.75 (m, 4H), 2.38 – 2.27 (m, 2H), 1.42 (s, 9H), 1.16 (d, *J* = 6.6 Hz, 3H).

^{13}C{^{1}H} NMR (126 MHz, $CDCl_3$) δ 158.9, 155.3, 132.1, 130.3, 127.2, 123.9, 113.9, 79.1, 55.3, 46.4, 40.5, 28.4, 20.6.

IR (solid): 3377, 2967, 2928, 1682, 1508, 1244, 1059, 968 cm^{-1}.

HRMS (ESI): *m/z* calculated for $[M + H]^+$ $(C_{17}H_{26}N_1O_3)^+$: 292.1907; found 292.1908.

tert-Butyl (_E_)-(5-(phenyl)pent-4-en-2-yl)carbamate (20)

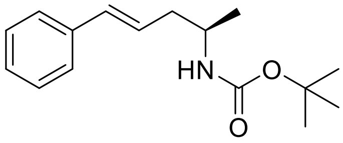

tert-butyl (*R*,*E*)-(5-phenylpent-4-en-2-yl)carbamate
Chemical Formula: $C_{16}H_{23}NO_2$
Molecular Weight: 261.37

Prepared according to General Procedure C with 1,3-dioxoisoindolin-2-yl *N*-(*tert*-butoxycarbonyl)-*N*-methylglycinate **2i** (69.7 mg, 0.200 mmol, 1.00 eq.), phenylvinylboronic acid (59 mg, 0.40 mmol, 2.0 eq.), triphenylamine (4.9 mg, 0.020 mmol, 10 mol%) and 4CzIPN (1.6 mg, 2.0 µmol, 1.0 mol%) in DMSO (1 mL). The crude was purified by column chromatography (basified silica, 0 to 5% ethyl acetate in hexane) to afford the desired product as a colourless oil (32 mg, 61%).

^{1}H NMR (400 MHz, $CDCl_3$) δ 7.41 – 7.27 (m, 4H), 7.27 – 7.19 (m, 1H), 6.45 (d, *J* = 15.8 Hz, 1H), 6.27 – 6.15 (m, 1H), 4.46 (s, 1H), 3.84 (s, 1H), 2.45 – 2.32 (m, 2H), 1.45 (s, 9H), 1.22 – 1.17 (m, 3H).

Spectroscopic data in agreement with the literature.[26]

tert-Butyl (_R_,_E_)-(5-(2-bromophenyl)pent-4-en-2-yl)carbamate (21)

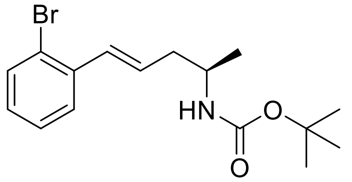

tert-butyl (*R*,*E*)-(5-(2-bromophenyl)pent-4-en-2-yl)carbamate
Chemical Formula: $C_{16}H_{22}BrNO_2$
Molecular Weight: 340.26

Prepared according to General Procedure C with 1,3-dioxoisoindolin-2-yl (*R*)-3-((*tert*-butoxycarbonyl)amino)butanoate **2i** (45 mg, 0.10 mmol, 1.0 eq.), *o*-bromo-phenylvinylboronic acid (35 mg, 0.20 mmol, 2.0 eq.), triphenylamine (2.5 mg, 0.010 mmol, 10 mol%) and 4CzIPN (0.8 mg, 1.0 µmol, 1.0 mol%) in DMSO (0.5 mL). The crude was purified by column chromatography (basified silica, 0 to 5% ethyl acetate in hexane) to afford the desired product as a white solid (19 mg, 55%).

^{1}H NMR (500 MHz, $CDCl_3$) δ 7.52 (dd, *J* = 8.0, 1.3 Hz, 1H), 7.48 (dd, *J* = 7.8, 1.7 Hz, 1H), 7.24 (td, *J* = 7.5, 1.5 Hz, 1H), 7.07 (td, *J* = 7.7, 1.7 Hz, 1H), 6.74 (d, *J* = 15.7 Hz, 1H), 6.12 (dt, *J* = 15.3, 7.4 Hz, 1H), 4.43 (br s, 1H), 3.84 (br s, 1H), 2.43 – 2.37 (m, 2H), 1.42 (s, 9H), 1.18 (d, *J* = 6.7 Hz, 3H).

^{13}C{^{1}H} NMR (126 MHz, $CDCl_3$) δ 155.4, 137.5, 132.9, 131.7, 129.7, 128.6, 127.6, 127.2, 123.3, 79.3, 46.3, 40.9, 28.6, 20.9.

IR (solid): 3366, 1678, 1520, 1250, 1169, 1059, 961, 760 cm^{-1}.

HRMS (ESI): *m/z* calculated for $[M + H]^+$ $(C_{16}H_{23}Br_1N_1O_2)^+$: 340.0907; found 340.0911.

***tert*-Butyl (*E*)-(4-phenyl-1-(*p*-tolyl)but-3-en-1-yl)carbamate (22)**

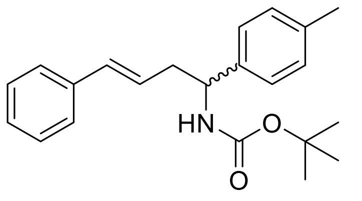

tert-butyl (*E*)-(4-phenyl-1-(*p*-tolyl)but-3-en-1-yl)carbamate
Chemical Formula: $C_{22}H_{27}NO_2$
Molecular Weight: 337.46

Prepared according to General Procedure C with 1,3-dioxoisoindolin-2-yl 3-((*tert*-butoxycarbonyl)amino)-3-(*p*-tolyl)propanoate **2g** (85 mg, 0.20 mmol, 1.0 eq.), phenylvinylboronic acid (59 mg, 0.40 mmol, 2.0 eq.), triphenylamine (4.9 mg, 0.020 mmol, 10 mol%) and 4CzIPN (1.6 mg, 2.0 µmol, 1.0 mol%) in DMSO (1 mL). The crude was purified by column chromatography (basified silica, 0 to 5% ethyl acetate in hexane) to afford the desired product as a white solid (55 mg, 82%).

^{1}H NMR (500 MHz, CDCl$_3$) δ 7.33 – 7.27 (m, 3H), 7.23 – 7.14 (m, 5H), 6.49 – 6.43 (m, 1H), 6.08 (dt, *J* = 15.8, 7.2 Hz, 1H), 4.91 (br s, 1H), 4.81 (br s, 1H), 2.68 (t, *J* = 7.0 Hz, 2H), 2.35 (s, 3H), 1.41 (s, 9H).

^{13}C{^{1}H} NMR (126 MHz, CDCl$_3$) δ 155.2, 139.3, 137.3, 136.8, 133.0, 129.3, 128.5, 127.3, 126.2 (two carbons), 125.8, 79.5, 54.2, 40.5, 28.4, 21.1.

IR (solid): 3389, 2922, 2855, 1682, 1512, 1167, 1016, 816 cm^{-1}.

HRMS (ESI): *m/z* calculated for $[M + Na]^+$ $(C_{22}H_{27}N_1O_2Na_1)^+$: 360.1934; found 360.1930.

***tert*-Butyl (*E*)-(4-(4-chlorophenyl)-1-(*p*-tolyl)but-3-en-1-yl)carbamate (23)**

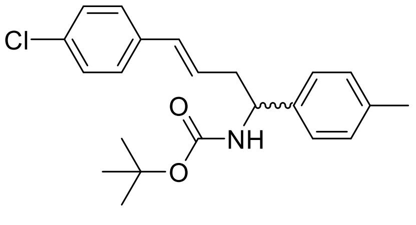

tert-butyl (*E*)-(4-(4-chlorophenyl)-1-(*p*-tolyl)but-3-en-1-yl)carbamate
Chemical Formula: $C_{22}H_{26}ClNO_2$
Molecular Weight: 371.91

Prepared according to General Procedure C with 1,3-dioxoisoindolin-2-yl 3-((*tert*-butoxycarbonyl)amino)-3-(*p*-tolyl)propanoate **2g** (85 mg, 0.20 mmol, 1.0 eq.), *p*-chloro-phenylvinylboronic acid (73 mg, 0.40 mmol, 2.0 eq.), triphenylamine (4.9 mg, 0.020 mmol, 10 mol%) and 4CzIPN (1.6 mg, 2.0 µmol, 1.0 mol%) in DMSO (1 mL). The crude was purified by column chromatography (basified silica, 0 to 5% ethyl acetate in hexane) to afford the desired product as a white solid (47 mg, 63%).

^{1}H NMR (400 MHz, CDCl$_3$) δ 7.27 – 7.12 (m, 8H), 6.39 (dt, *J* = 15.8, 1.5 Hz, 1H), 6.05 (dt, *J* = 15.8, 7.2 Hz, 1H), 4.87 (d, *J* = 8.0 Hz, 1H), 4.78 (br s, 1H), 2.70 – 2.62 (m, 2H), 2.34 (s, 3H), 1.40 (s, 9H).

^{13}C{^{1}H} NMR (126 MHz, CDCl$_3$) δ 155.2, 139.1, 136.9, 135.8, 132.8, 131.7, 129.3, 128.6, 127.4, 126.7, 126.2, 79.6, 54.3, 40.5, 28.4, 21.1.

IR (solid): 3381, 2978, 2918, 1682, 1514, 1167, 797 cm^{-1}.

HRMS (ESI): *m/z* calculated for $[M + H]^+$ $(C_{22}H_{27}N_1O_2Cl)^+$: 372.1725; found 372.1718.

tert-Butyl (*E*)-(2-styrylcyclopentyl)carbamate (25)

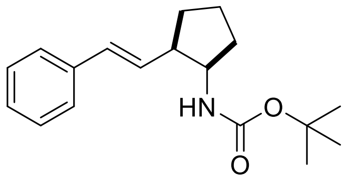

tert-butyl ((1*R*,2*R*)-2-((*E*)-styryl)cyclopentyl)carbamate
Chemical Formula: $C_{18}H_{25}NO_2$
Molecular Weight: 287.40

Prepared according to General Procedure C with 1,3-dioxoisoindolin-2-yl 2-((*tert*-butoxycarbonyl)amino)cyclopentane-1-carboxylate **2j** (74.9 mg, 0.200 mmol, 1.00 eq.), phenylvinylboronic acid (59.2 mg, 0.200 mmol, 2.00 eq.), triphenylamine (4.9 mg, 0.020 mmol, 10 mol%) and 4CzIPN (1.6 mg, 2.0 µmol, 1.0 mol%) in DMSO (1 mL). The crude was purified by column chromatography (basified silica, 0 to 5% ethyl acetate in hexane) to afford the desired product as a colourless oil (39 mg, 68%) as a single diastereomer.

^{1}H NMR (500 MHz, CDCl$_3$) δ 7.37 – 7.33 (m, 2H), 7.31 – 7.26 (m, 2H), 7.21 – 7.17 m, 1H), 6.41 (d, *J* = 15.8 Hz, 1H), 6.15 (dd, *J* = 15.8, 8.1 Hz, 1H), 4.50 (br s, 1H), 3.71 (br s, 1H), 2.39 – 2.29 (m, 1H), 2.22 – 2.13 (m, 1H), 1.98 – 1.90 (m, 1H), 1.76 – 1.68 (m, 2H), 1.60 – 1.50 (m, 1H), 1.48 – 1.41 (m, 1H), 1.40 (s, 9H).

^{13}C{^{1}H} NMR (126 MHz, CDCl$_3$) δ 155.9, 137.6, 132.3, 130.3, 128.6, 127.1, 126.2, 79.2, 57.6, 50.8, 32.5, 30.7, 28.5, 21.8.

IR (solid): 3350, 1736, 1678, 1522, 1364, 1082, 876, 785 cm^{-1}.

HRMS (ESI): *m/z* calculated for $[M + H]^+$ $(C_{18}H_{26}N_1O_2)^+$: 288.1958; found 288.1959.

tert-Butyl (*R*)-(1-(3,4-dihydronaphthalen-2-yl)propan-2-yl)carbamate (26)

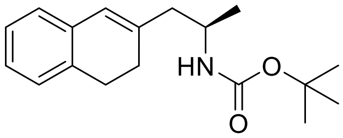

tert-butyl (*R*)-(1-(3,4-dihydronaphthalen-2-yl)propan-2-yl)carbamate
Chemical Formula: $C_{18}H_{25}NO_2$
Molecular Weight: 287.40

Prepared according to General Procedure C with 1,3-dioxoisoindolin-2-yl (*R*)-3-((*tert*-butoxycarbonyl)amino)butanoate **2d** (70 mg, 0.20 mmol, 1.0 eq.), (3,4-dihydronaphthalen-2-yl)boronic acid (70 mg, 0.40 mmol, 2.0 eq.), triphenylamine (4.9 mg, 0.020 mmol, 10 mol%) and 4CzIPN (1.6 mg, 2.0 µmol, 1.0 mol%) in DMSO (1 mL). The crude was purified by column chromatography (basified silica, 0 to 5% ethyl acetate in hexane) to afford the desired product as a white solid (14 mg, 25%).

^{1}H NMR (500 MHz, CDCl$_3$) δ 7.15 – 7.10 (m, 1H), 7.10 – 7.07 (m, 2H), 6.99 – 6.96 (m, 1H), 6.24 (s, 1H), 4.38 (s, 1H), 3.94 – 3.84 (m, 1H), 2.88 – 2.73 (m, 2H), 2.41 – 2.20 (m, 4H), 1.41 (s, 9H), 1.16 (d, *J* = 6.5 Hz, 3H).

$^{13}C\{^1H\}$ NMR (126 MHz, $CDCl_3$) δ 155.3, 138.5, 134.6, 127.2, 126.4, 125.5, 125.0, 80.6, 45.4, 45.0, 28.4, 28.1, 27.1, 21.1.

IR (solid): 3194, 2924, 2951, 1717, 1695, 1306, 1169, 1051 cm^{-1}.

HRMS (ESI): *m/z* calculated for $[M + Na]^+$ $(C_{18}H_{25}N_1O_2Na_1)^+$: 310.17775; found 310.17765.

Reprotection

(*E*)-*N*-(3-(4-chlorophenyl)allyl)acetamide (9)

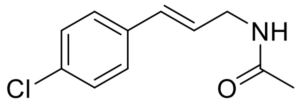

(*E*)-*N*-(3-(4-chlorophenyl)allyl)acetamide
Chemical Formula: $C_{11}H_{12}ClNO$
Molecular Weight: 209.67

Prepared according to General Procedure D with *tert*-butyl (*E*)-(3-(4-chlorophenyl)allyl)carbamate **7** (72 mg, 0.27 mmol, 1.0 eq.), TFA:CH_2Cl_2 (1:1, 5 mL) and then Ac_2O (33 µL, 0.35 mmol, 1.3 eq.) and Na_2CO_3 (86 mg, 0.81 mmol, 3.0 eq.) in THF:H_2O (10:1, 3 mL). The crude was purified by column chromatography (basified silica, 0 to 15% acetone in cyclohexane) to afford the desired product as a white solid (44 mg, 76%).

^1H NMR (400 MHz, $CDCl_3$) δ 7.29 – 7.27 (m, 4H), 6.47 (d, J = 15.9 Hz, 1H), 6.17 (dt, J = 15.2, 6.4 Hz, 1H), 5.57 (s, 1H), 4.05 – 4.01 (m, 2H), 2.03 (s, 3H).

Spectroscopic data in agreement with the literature.[27]

(*E*)-*N*-(4-phenylbut-3-en-1-yl)acetamide (18)

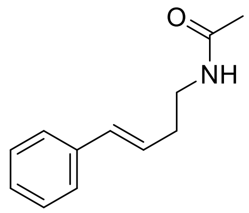

(*E*)-*N*-(4-phenylbut-3-en-1-yl)acetamide
Chemical Formula: $C_{12}H_{15}NO$
Molecular Weight: 189.26

Prepared according to General Procedure D with *tert*-butyl (*E*)-(4-phenylbut-3-en-1-yl)carbamate **17** (73 mg, 0.29 mmol, 1.0 eq.) in TFA:CH_2Cl_2 (1:1, 3 mL) and then Na_2CO_3 (70 mg, 0.66 mmol, 2.3 eq.) and Ac_2O (27 µL, 0.30 mmol, 1.0 eq.) in THF:H_2O (10:1, 0.5 mL). The crude was purified by column chromatography (basified silica, 0 to 30% acetone in hexane) to afford the desired product as a white solid (44 mg, 79%).

^1H NMR (400 MHz, $CDCl_3$) δ 7.40 – 7.19 (m, 5H), 6.47 (d, J = 15.8 Hz, 1H), 6.23 – 6.10 (m, 1H), 5.83 (s, 1H), 3.45 – 3.35 (m, 2H), 2.49 – 2.39 (m, 2H), 1.98 (s, 3H).

Spectroscopic data in agreement with the literature.[28]

(*E*)-*N*-(4-(4-Chlorophenyl)-1-(*p*-tolyl)but-3-en-1-yl)acetamide (24)

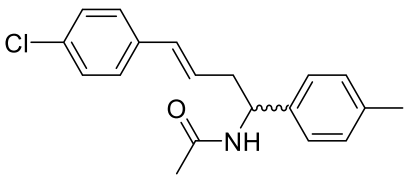

(*E*)-*N*-(4-(4-chlorophenyl)-1-(*p*-tolyl)but-3-en-1-yl)acetamide
Chemical Formula: $C_{19}H_{20}ClNO$
Molecular Weight: 313.83

Prepared according to General Procedure D with *tert*-butyl (*E*)-(4-(4-chlorophenyl)-2-(*p*-tolyl)but-3-en-1-yl)carbamate **23** (0.15 g, 0.40 mmol, 1.0 eq.) in TFA:CH_2Cl_2 (1:1, 8 mL) and then Na_2CO_3 (0.097 g, 0.92 mmol, 2.3 eq.) and Ac_2O (37 µL, 0.52 mmol, 1.3 eq.) in THF:H_2O (10:1, 1 mL). The crude was purified by column chromatography (basified silica, 0 to 30% acetone in hexane) to afford the desired product as a white solid (79 mg, 63%).

^{1}H NMR (500 MHz, $CDCl_3$) δ 7.28 – 7.13 (m, 8H), 6.39 (d, J = 15.8 Hz, 1H), 6.03 (dt, J = 15.8, 7.1 Hz, 1H), 5.74 – 5.67 (m, 1H), 5.15 – 5.08 (m, 1H), 2.79 – 2.65 (m, 2H), 2.34 (s, 3H), 1.98 (s, 3H).

^{13}C{^{1}H} NMR (126 MHz, $CDCl_3$) δ 169.2, 138.3, 137.3, 135.7, 132.9, 131.8, 129.5, 128.7, 127.4, 126.5 (two carbons), 52.7, 39.6, 23.5, 21.1.

IR (solid): 3273, 1645, 1557, 1373, 1098, 959, 795 cm^{-1}.

HRMS (ESI): *m/z* calculated for $[M + H]^+$ $(C_{19}H_{21}Cl_1N_1O_1)^+$: 314.1306; found 314.1306.

NXS Cyclisation

5-Iodo-6-phenyl-1,3-oxazinan-2-one (27)

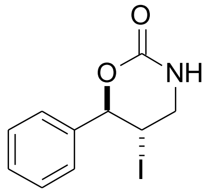

5-iodo-6-phenyl-1,3-oxazinan-2-one
Chemical Formula: $C_{10}H_{10}INO_2$
Molecular Weight: 303.10

Prepared according to General Procedure E with *tert*-butyl cinnamylcarbamate **3** (46.7 mg, 0.200 mmol, 1.00 eq.), and *N*-iodosuccinimide (0.135 g, 0.600 mmol, 3.00 eq.) in MeCN (1 mL) for 20 h. The crude was purified by column chromatography (silica, 0 to 20% ethyl acetate in hexane) to afford the desired product as a white solid (47 mg, 77%) as a single diastereomer.

^{1}H NMR (400 MHz, $CDCl_3$) δ 7.43 – 7.38 (m, 3H), 7.38 – 7.33 (m, 2H), 6.31 (br s, 1H), 5.41 (d, J = 8.0 Hz, 1H), 4.37 (dt, J = 7.9, 6.5 Hz, 1H), 3.69 (dd, J = 6.5, 2.5 Hz, 2H).

^{13}C{^{1}H} NMR (126 MHz, $CDCl_3$) δ 153.1, 137.1, 129.5, 128.9, 126.9, 84.6, 48.5, 19.2.

IR (solid): 3221, 3109, 2922, 2853, 1734, 1684, 1474, 1456, 1396, 1265, 1132, 982, 756 cm^{-1}.

HRMS (ESI): *m/z* calculated for $[M + H]^+$ $(C_{10}H_{11}INO_2)^+$: 303.9829; found 303.9830.

5-Bromo-6-phenyl-1,3-oxazinan-2-one (28)

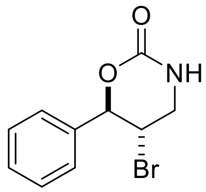

5-bromo-6-phenyl-1,3-oxazinan-2-one
Chemical Formula: $C_{10}H_{10}BrNO_2$
Molecular Weight: 256.10

Prepared according to General Procedure E with t*ert*-butyl cinnamylcarbamate **3** (47 mg, 0.20 mmol, 1.0 eq.) and *N*-bromosuccinimide (0.11 g, 0.60 mmol, 3.0 eq.) in MeCN (2.0 mL) for 20 h. The crude was purified by column chromatography (silica, 0 to 20% ethyl acetate in hexane) to afford the desired product as a white solid (5.0 mg, 10%) as a single diastereomer.

^{1}H NMR (500 MHz, CDCl$_3$) δ 7.48 – 7.32 (m, 5H), 5.45 (d, *J* = 6.4 Hz, 1H), 4.34 (td, *J* = 6.5, 4.4 Hz, 1H), 3.71 (dd, *J* = 12.6, 4.5 Hz, 1H), 3.59 (dd, *J* = 12.9, 6.9 Hz, 1H).

^{13}C{^{1}H} NMR (126 MHz, CDCl$_3$) δ 136.4, 129.3, 128.9, 126.3, 83.1, 46.0, 42.1.

IR (solid): 3227, 3111, 2922, 1684, 1458, 1275, 1275, 1148, 1003 cm^{-1}.

HRMS (ESI): *m/z* calculated for $[M + H]^+$ $(C_{10}H_{11}Br_1N_1O_2)^+$: 255.9968; found 255.9971.

6-(4-chlorophenyl)-5-iodo-1,3-oxazinan-2-one (29)

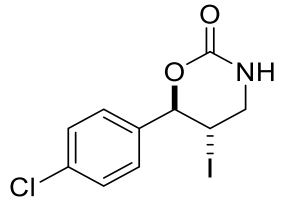

6-(4-chlorophenyl)-5-iodo-1,3-oxazinan-2-one
Chemical Formula: $C_{10}H_9ClINO_2$
Molecular Weight: 337.54

Prepared according to General Procedure E with *tert*-butyl (*E*)-(3-(4-chlorophenyl)allyl)carbamate **7** (26 mg, 0.10 mmol, 1.0 eq.) and *N*-iodosuccinimide (68 mg, 0.30 mmol, 3.0 eq.) in MeCN (1 mL) for 20 h. The crude was dissolved in minimal amount of acetone, treated with hexane and left in the freezer for 10 minutes. The resulting solid was filtered off to afford the desired product (20 mg, 58%) as a single diastereomer.

^{1}H NMR (500 MHz, CDCl$_3$) δ 7.42 – 7.36 (m, 2H), 7.33 – 7.28 (m, 2H), 5.85 (s, 1H), 5.36 (d, *J* = 8.6 Hz, 1H), 4.29 (td, *J* = 8.4, 5.7 Hz, 1H), 3.79 – 3.66 (m, 2H).

^{13}C{^{1}H} NMR (126 MHz, CDCl$_3$) δ 152.4, 135.4, 129.0, 128.4, 83.8, 48.7, 18.6.

IR (solid): 3219, 3111, 2924. 2376, 2313, 1684, 1269, 1134, 1082, 984, 818, 764 cm^{-1}.

HRMS (ESI): *m/z* calculated for $[M + H]^+$ $(C_{10}H_{10}Cl_1I_1N_1O_2)^+$: 337.9439; found 337.9450.

5-Iodo-3-methyl-6-phenyl-1,3-oxazinan-2-one (30)

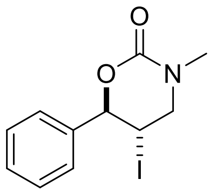

5-iodo-3-methyl-6-phenyl-1,3-oxazinan-2-one
Chemical Formula: $C_{11}H_{12}INO_2$
Molecular Weight: 317.13

Prepared according to General Procedure E with *tert*-butyl cinnamyl(methyl)carbamate **12** (57 mg, 0.23 mmol, 1.0 eq.) and *N*-iodosuccinimide (0.16 g, 0.69 mmol, 3.0 eq.) in MeCN (1.2 mL) for 20 h. The crude was purified by column chromatography (silica, 0 to 20% acetone in hexane) to afford the desired product as a white crystalline solid (72 mg, 98%) as a single diastereomer.

^{1}H NMR (500 MHz, CDCl$_3$) δ 7.39 – 7.35 (m, 3H), 7.33 – 7.29 (m, 2H), 5.31 (d, *J* = 8.7 Hz, 1H), 4.38 (td, *J* = 8.8, 5.2 Hz, 1H), 3.71 (dd, *J* = 12.5, 8.9 Hz, 1H), 3.62 (dd, *J* = 12.5, 5.2 Hz, 1H), 3.02 (s, 3H).

^{13}C{^{1}H} NMR (126 MHz, CDCl$_3$) δ 152.6, 137.0, 129.4, 128.7, 126.9, 83.6, 55.6, 37.0, 19.5.

IR (solid): 3219, 3111, 2924. 2376, 2313, 1684, 1269, 1134, 1082, 984, 818, 764 cm^{-1}.

HRMS (ESI): *m/z* calculated for $[M + Na]^+$ $(C_{11}H_{12}I_1N_1O_2Na_1)^+$: 339.98049; found 339.97957.

mp 98 – 99 °C

1-(2-(Bromo(phenyl)methyl)aziridin-1-yl)ethan-1-one (32)

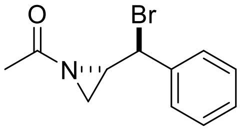

1-(2-(bromo(phenyl)methyl)aziridin-1-yl)ethan-1-one
Chemical Formula: $C_{11}H_{12}BrNO$
Molecular Weight: 254.12700

Prepared according to General Procedure E with *N*-cinnamylacetamide **5** (35 mg, 0.20 mmol, 1.0 eq.) and *N*-bromosuccinimide (43 mg, 0.24 mmol, 1.2 eq.) for 4 h. The crude was purified by column chromatography (basified silica, 0 to 20% ethyl acetate in hexane) to afford the desired product as a colourless oil (42 mg, 82%) as a single diastereomer.

^{1}H NMR (400 MHz, CDCl$_3$) δ 7.44 — 7.30 (m, 5H), 5.12 (d, *J* = 8.2 Hz, 1H), 4.20 (td, *J* = 8.3, 4.9 Hz, 1H), 3.79 (ddq, *J* = 16.6, 4.9, 1.2 Hz, 1H), 3.70 – 3.62 (m, 1H), 2.01 (s, 3H).

^{13}C{^{1}H} NMR (126 MHz, CDCl$_3$) δ 157.9, 137.6, 129.2, 128.8, 127.0, 80.8, 50.3, 45.4, 21.4.

IR (film): 2970, 2924, 1676, 1383, 1229, 1090, 1047, 1015, 880, 787 cm^{-1}.

HRMS (ESI): *m/z* calculated for $[M + H]^+$ $(C_{11}H_{13}N_1O_1Br_1)^+$: 254.0175; found 254.0177.

1-(2-(Iodo(phenyl)methyl)aziridin-1-yl)etha-1-one (33)

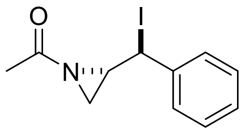

1-(2-(iodo(phenyl)methyl)aziridin-1-yl)ethan-1-one
Chemical Formula: $C_{11}H_{12}INO$
Molecular Weight: 301.13

Prepared according to General Procedure E with *N*-cinnamylacetamide **5** (15 mg, 0.085 mmol, 1.0 eq.) and *N*-iodosuccinimide (58 mg, 0.26 mmol, 3.0 eq.) in MeCN (0.9 mL) for 4 h. The crude was purified by column chromatography (basified silica, 0 to 20% ethyl acetate in hexane) to afford the desired product as a colourless oil (6.6 mg, 27%) as a single diastereomer.

^{1}H NMR (400 MHz, $CDCl_3$) δ 7.43 – 7.38 (m, 3H), 7.35 – 7.31 (m, 2H), 5.18 (d, *J* = 9.4 Hz, 1H), 4.31 (q, *J* = 8.2 Hz, 1H), 3.86 – 3.81 (m, 2H), 1.98 (s, 3H).

^{13}C{^{1}H} NMR (126 MHz, $CDCl_3$) δ 171.2, 138.1, 129.2, 128.6, 127.1, 81.9, 52.8, 24.2, 21.5.

IR (solid): 2926, 1674, 1379, 1213, 1009, 770, 696 cm^{-1}.

HRMS (ESI): *m/z* calculated for $[M + H]^+$ $(C_{11}H_{13}N_1O_1I_1)^+$: 302.0036; found 302.0033.

1-(2-Benzyl-3-(bromo(phenyl)methyl)aziridine-1-yl)ethan-1-one (34)

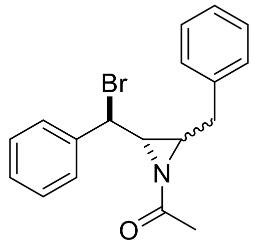

1-(2-benzyl-3-(bromo(phenyl)methyl)aziridin-1-yl)ethan-1-one
Chemical Formula: $C_{18}H_{18}BrNO$
Molecular Weight: 344.25

Prepared according to General Procedure E with (*E*)-*N*-(1,4-diphenylbut-3-3n-2-yl)acetamide **11** (24 mg, 0.090 mmol, 1.0 eq.) and *N*-bromosuccinimide (19 mg, 0.11 mmol, 1.2 eq.) in MeCN (1 mL) for 4 h. The crude was purified by column chromatography (basified silica, 0 to 15% ethyl acetate in cyclohexane) to afford the desired product as a colourless oil (23 mg, 73%).

Diastereomer ratio determined from crude ^{1}H NMR – 1.0:0.71.

Major diastereomer:

^{1}H NMR (500 MHz, $CDCl_3$) δ 7.41 – 7.19 (m, 10H), 5.07 (d, *J* = 10.4 Hz, 1H), 4.06 – 3.99 (m, 1H), 3.79 (t, *J* = 10.1 Hz, 1H), 3.36 – 3.28 (m, 1H), 3.05 (dd, *J* = 13.6, 6.3 Hz, 1H), 1.97 (d, *J* = 1.7 Hz, 3H).

^{13}C{^{1}H} NMR (126 MHz, $CDCl_3$) δ 157.4, 137.3, 137.1, 130.1, 129.2, 128.5, 128.1, 127.7, 126.5, 80.2, 61.3, 51.2, 39.2, 21.0.

Minor diastereomer:

^{1}H NMR (500 MHz, $CDCl_3$) δ 7.41 – 7.19 (m, 6H), 7.19 – 7.15 (m, 2H), 7.14 – 7.10 (m, 2H), 5.52 (d, *J* = 2.3 Hz, 1H), 4.17 – 4.15 (m, 1H), 3.36 – 3.28 (m, 1H), 3.10 (dd, *J* = 13.7, 5.6 Hz, 1H), 2.88 (dd, *J* = 13.7, 9.8 Hz, 1H), 1.97 (d, *J* = 1.7 Hz, 3H).

^{13}C{^{1}H} NMR (126 MHz, $CDCl_3$) δ 156.0, 139.0, 137.6, 129.2, 129.0 (two carbons), 128.5, 126.6, 124.8, 80.9, 51.5, 50.7, 41.4, 21.4.

IR (solid): 3028, 2924, 2851, 1674, 1454, 1229, 1061 cm^{-1}.

HRMS (ESI): *m/z* calculated for $[M + Na]^+$ $(C_{18}H_{18}Br_1N_1O_1Na_1)^+$: 366.0464; found 366.0465.

1-(2-(Bromo(4-chlorophenyl)methyl)aziridine-1-yl)ethan-1-one (35)

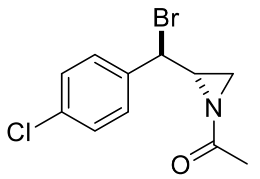

1-(2-(bromo(4-chlorophenyl)methyl)aziridin-1-yl)ethan-1-one
Chemical Formula: $C_{11}H_{11}BrClNO$
Molecular Weight: 288.57

Prepared according to General Procedure E with (*E*)-*N*-(3-(4-chlorophenyl)allyl)acetamide **9** (42 mg, 0.20 mmol, 1.0 eq.) and *N*-bromosuccinimide (42 mg, 0.24 mmol, 1.2 eq.) in MeCN (2 mL) for 4 h. The crude was purified by column chromatography (basified silica, 0 to 15% ethyl acetate in cyclohexane) to afford the desired product as white crystalline solid (29 mg, 51%) as a single diastereomer.

^{1}H NMR (500 MHz, $CDCl_3$) δ 7.40 – 7.36 (m, 2H), 7.30 – 7.26 (m, 2H), 5.07 (d, *J* = 8.8 Hz, 1H), 4.12 (td, *J* = 9.0, 5.0 Hz, 1H), 3.81 (ddq, *J* = 16.6, 5.1, 1.1 Hz, 1H), 3.67 (ddq, *J* = 16.5, 9.0, 1.7 Hz, 1H), 2.00 (t, *J* = 1.4 Hz, 3H).

^{13}C{^{1}H} NMR (126 MHz, $CDCl_3$) δ 157.6, 135.8, 135.1, 128.9, 128.4, 80.0, 50.6, 45.1, 21.2.

IR (film): 2968, 2924, 1680, 1383, 1229, 1092, 1049, 1013, 820 cm^{-1}.

HRMS (ESI): *m/z* calculated for $[M + H]^+$ $(C_{11}H_{12}N_1O_1Br_1Cl_1)^+$: 287.9785; found 287.9784.

tert-Butyl 3-bromo-2-phenylpyrrolidine-1-carboxylate (36)

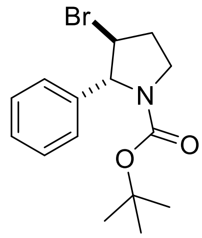

tert-butyl bromo-2-phenylpyrrolidine-1-carboxylate
Chemical Formula: $C_{15}H_{20}BrNO_2$
Molecular Weight: 326.23

Prepared according to General Procedure E with *tert*-butyl (*E*)-(4-phenylbut-3-en-1-yl)carbamate **17** (49 mg, 0.20 mmol, 1.0 eq.) and *N*-bromosuccinimide (43 mg, 0.24 mmol, 1.2 eq.) for 4 h. The crude was purified by column chromatography (basified silica, 0 to 50% ethyl acetate in hexane) to afford the desired product as a colourless oil (28 mg, 42%) as a single diastereomer.

Rotamer ratio determined from crude ^{1}H NMR – 1.0:0.51.

Major rotamer:

^{1}H NMR (500 MHz, CDCl$_3$) δ 7.36 – 7.30 (m, 2H), 7.30 – 7.26 (m 1H), 7.22 – 7.16 (m, 2H), 5.11 (s, 1H), 4.30 (br s, 1H), 3.93 – 3.82 (m, 1H), 2.23 – 2.10 (m, 1H), 1.22 (s, 9H).

^{13}C NMR (126 MHz, CDCl$_3$) δ 154.4, 141.4, 128.6, 127.6, 125.5, 79.9, 71.0, 54.7, 44.9, 32.7, 28.1.

Major rotamer:

^{1}H NMR (500 MHz, CDCl$_3$) δ 7.36 – 7.30 (m, 2H), 7.30 – 7.26 (m 1H), 7.22 – 7.16 (m, 2H), 5.32 (s, 1H), 4.30 (br s, 1H), 3.82 – 3.68 (m, 1H), 2.47 – 2.34 (m, 1H), 1.49 (s, 9H).

^{13}C NMR (126 MHz, CDCl$_3$) δ 154.4, 140.6, 128.8, 127.6, 125.5, 79.9, 70.6, 54.3, 45.2, 33.1, 28.5.

Spectroscopic data in agreement with the literature.[29]

1-(3-Bromo-2-phenylpyrrolidin-1-yl)ethan-1-one (37)

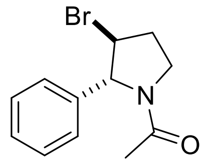

1-(3-bromo-2-phenylpyrrolidin-1-yl)ethan-1-one
Chemical Formula: $C_{12}H_{14}BrNO$
Molecular Weight: 268.15

Prepared according to General Procedure E with the (*E*)-*N*-(4-phenylbut-3-en-1-yl)acetamide **18** (44 mg, 0.23 mmol, 1.0 eq.) and *N*-bromosuccinimide (49 mg, 0.28 mmol, 1.2 eq.) for 4 h. The crude was purified by column chromatography (basified silica, 0 to 50% ethyl acetate in hexane) to afford the desired product as a colourless oil (22 mg, 36%) as a single diastereomer.

Rotamer ratio determined from crude ^{1}H NMR – 1.0:0.40.

Major rotamer:

^{1}H NMR (500 MHz, CDCl$_3$) δ 7.41 – 7.36 (m, 2H), 7.35 – 7.30 (m 1H), 7.22 – 7.19 (m, 2H), 5.19 (s, 1H), 4.40 – 4.38 (m, 1H), 4.05 – 3.89 (m, 1H), 3.93 – 3.87 (m, 1H), 2.42 – 2.33 (m, 1H), 2.22 – 2.16 (m, 1H), 1.87 (s, 3H).

^{13}C{^{1}H} NMR (126 MHz, CDCl$_3$) δ 170.3, 139.7, 129.3, 128.5, 125.6, 72.2, 54.9, 44.9, 31.6, 22.5.

Minor rotamer:

^{1}H NMR (500 MHz, CDCl$_3$) δ 7.41 – 7.36 (m, 2H), 7.27 – 7.24 (m 1H), 7.17 – 7.14 (m, 2H), 5.56 (s, 1H), 4.40 – 4.38 (m, 1H), 4.05 – 3.89 (m, 1H), 3.86 – 3.81 (m, 1H), 2.53 – 2.44 (m, 1H), 2.28 – 2.22 (m, 1H), 2.22 (s, 3H).

^{13}C{^{1}H} NMR (126 MHz, CDCl$_3$) δ 169.7, 139.6, 128.8, 127.8, 125.5, 70.2, 53.6, 46.2, 33.3, 22.5.

IR (solid): 2970, 2889, 1628, 1416, 1047, 752 cm^{-1}.

HRMS (ESI): *m/z* calculated for $[M + H]^+$ $(C_{12}H_{15}N_1O_1Br)^+$: 268.0332; found 268.0333.

***tert*-Butyl 3-bromo-5-methyl-2-phenylpyrrolidine-1-carboxylate (38)**

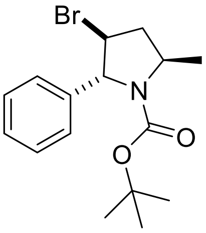

tert-butyl 3-bromo-5-methyl-2-phenylpyrrolidine-1-carboxylate
Chemical Formula: $C_{16}H_{22}BrNO_2$
Molecular Weight: 340.26

Prepared according to General Procedure E with the *tert*-butyl (*E*)-(5-phenylpent-4-en-2-yl)carbamate **20** (52 mg, 0.20 mmol, 1.0 eq.) and *N*-bromosuccinimide (0.043 g, 0.24 mmol, 1.2 eq.) for 4 h. The crude was purified by column chromatography (basified silica, 0 to 1% ethyl acetate in hexane) to afford the desired product as a colourless oil (44 mg, 65%) as a single diastereomer.

Rotamer ratio determined from crude ^{1}H NMR – 1.0:0.37.

Major rotamer:

^{1}H NMR (500 MHz, CDCl$_3$) δ 7.39 – 7.31 (m, 2H), 7.32 – 7.23 (m, 1H), 7.19 – 7.13 (m, 2H), 5.20 (s, 1H), 4.49 – 4.35 (m, 1H), 4.28 – 4.21 (m, 1H), 2.88 – 2.76 (m, 1H), 2.15 – 2.08 (m, 1H), 1.69 (d, *J* = 6.5 Hz, 3H), 1.14 (s, 9H).

^{13}C{^{1}H} NMR (126 MHz, CDCl$_3$) δ 154.0, 142.5, 128.7, 127.6, 125.3, 79.7, 73.1, 54.7, 52.6, 39.7, 28.0, 21.1.

Minor rotamer:

^{1}H NMR (500 MHz, CDCl$_3$) δ 7.39 – 7.31 (m, 2H), 7.32 – 7.23 (m, 1H), 7.19 – 7.13 (m, 2H), 5.40 (s, 1H), 4.31 (br s, 1H), 4.28 – 4.21 (m, 1H), 2.88 – 2.76 (m, 1H), 2.15 – 2.08 (m, 1H), 1.69 (d, *J* = 6.5 Hz, 3H), 1.49 (s, 9H).

^{13}C{^{1}H} NMR (126 MHz, CDCl$_3$) δ 154.0, 141.2, 128.9, 127.6, 125.1, 80.0, 72.6, 54.5, 51.9, 40.0, 28.5, 22.2.

IR (film): 2976, 2930, 1694, 1379, 1366, 1169, 874 cm^{-1}.

HRMS (ESI): *m/z* calculated for $[M + H]^+$ $(C_{16}H_{23}N_1O_2Br_1)^+$: 340.0907; found 340.0903.

tert-Butyl 3-iodo-5-methyl-2-phenylpyrrolidine-1-carboxylate (39)

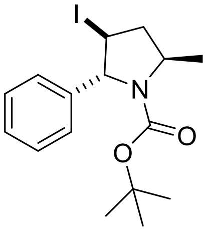

tert-butyl (5*R*)-3-iodo-5-methyl-2-phenylpyrrolidine-1-carboxylate
Chemical Formula: $C_{16}H_{22}INO_2$
Molecular Weight: 387.26

Prepared according to General Procedure E in a 4 mL vial with the *tert*-butyl (*E*)-(5-phenylpent-4-en-2-yl)carbamate **20** (52 mg, 0.20 mmol, 1.0 eq.) and *N*-iodosuccinimide (54 mg, 0.24 mmol, 1.2 eq.) for 4 h. The crude was purified by column chromatography (basified silica, 0 to 1% ethyl acetate in hexane) to afford the desired product as a colourless oil (59 mg, 76%) as a single diastereomer.

Rotamer ratio determined from crude ^{1}H NMR – 1.0:0.32.

Major rotamer:

^{1}H NMR (500 MHz, CDCl$_3$) δ 7.35 – 7.22 (m, 3H), 7.18 – 7.12 (m, 2H), 5.13 (s, 1H), 4.40 – 4.30 (m, 1H), 4.16 – 4.09 (m, 1H), 2.83 – 2.75 (m, 1H), 2.17 – 2.09 (m, 1H), 1.72 – 1.65 (m, 3H), 1.08 (s, 9H).

^{13}C{^{1}H} NMR (126 MHz, CDCl$_3$) δ 153.7, 143.0, 128.6, 128.2, 125.4, 79.6, 74.4, 55.3, 42.3, 28.0, 25.7, 20.8.

Minor rotamer:

^{1}H NMR (500 MHz, CDCl$_3$) δ 7.35 – 7.22 (m, 3H), 7.18 – 7.12 (m, 2H), 5.35 (s, 1H), 4.40 – 4.30 (br s, 1H), 4.16 – 4.09 (m, 1H), 2.83 – 2.75 (m, 1H), 2.17 – 2.09 (m, 1H), 1.72 – 1.65 (m, 3H), 1.46 (s, 9H).

^{13}C{^{1}H} NMR (126 MHz, CDCl$_3$) δ 153.7, 143.0, 128.9, 127.5, 125.0, 80.1, 74.2, 55.0, 41.9, 28.5, 24.5, 22.1.

IR (film): 2974, 2928, 1692, 1364, 1165, 1126 cm^{-1}.

HRMS (ESI): *m/z* calculated for $[M + H]^+$ $(C_{16}H_{23}N_1O_2I_1)^+$: 388.0768; found 388.0765.

tert-Butyl (5_R_)-3-bromo-2-(4-methoxyphenyl)-5-methylpyrrolidine-1-carboxylate (40)

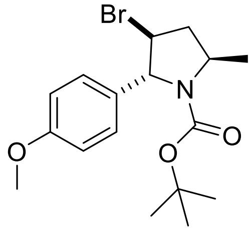

tert-butyl (5*R*)-3-bromo-2-(4-methoxyphenyl)-5-methylpyrrolidine-1-carboxylate
Chemical Formula: $C_{17}H_{24}BrNO_3$
Molecular Weight: 370.29

Prepared according to General Procedure E with the *tert*-butyl (*R*,*E*)-(5-(4-methoxyphenyl)pent-4-en-2-yl)carbamate **19** (23 mg, 0.080 mmol, 1.0 eq.) and *N*-bromosuccinimide (0.017 g, 0.096 mmol, 1.2 eq.) for 4 h. The crude was purified by column chromatography (basified silica, 0 to 10% ethyl acetate in hexane) to afford the desired product as pale yellow oil (10 mg, 32%) as a single diastereomer.

Rotamer ratio determined from crude ^{1}H NMR – 1.0:0.38.

Major rotamer:

^{1}H NMR (500 MHz, $CDCl_3$) δ 7.05 (d, *J* = 8.3 Hz, 2H), 6.90 – 6.81 (m, 2H), 5.14 (s, 1H), 4.42 – 4.32 (m, 1H), 4.20 – 4.17 (m, 1H), 3.79 (s, 3H), 2.84 – 2.75 (m, 1H), 2.11 – 2.02 (m, 1H), 1.65 (d, *J* = 6.4 Hz, 3H), 1.15 (s, 9H).

^{13}C{^{1}H} NMR (126 MHz, $CDCl_3$) δ 159.0, 154.0, 134.6, 126.5, 113.9, 79.6, 72.5, 55.3, 54.6, 53.0, 39.5, 28.1, 21.1.

Minor rotamer:

^{1}H NMR (500 MHz, $CDCl_3$) δ 7.23 – 7.18 (m, 2H), 6.90 – 6.81 (m, 2H), 5.32 (s, 1H), 4.32 – 4.23 (m, 2H), 3.79 (s, 3H), 2.84 – 2.75 (m, 1H), 2.11 – 2.02 (m, 1H), 1.51 – 1.41 (m, 9H).

^{13}C{^{1}H} NMR (126 MHz, $CDCl_3$) δ 159.0, 154.0, 134.6, 126.9, 114.3, 80.0, 72.1, 54.4, 53.2, 52.2, 34.0, 28.5, 22.2.

IR (solid): 2976, 2936, 1692, 1508, 1383, 1246, 1167, 1028, 874 cm^{-1}.

HRMS (ESI): *m/z* calculated for $[M + Na]^+$ $(C_{17}H_{24}Br_1N_1O_3Na_1)^+$: 392.0832; found 392.0821.

tert-Butyl (5_R_)-3-bromo-2-(2-bromophenyl)-5-methylpyrrolidine-1-carboxylate (41)

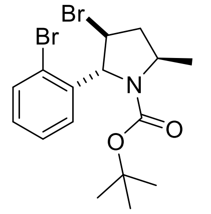

tert-butyl (5*R*)-3-bromo-2-(2-bromophenyl)-5-methylpyrrolidine-1-carboxylate
Chemical Formula: $C_{16}H_{21}Br_2NO_2$
Molecular Weight: 419.16

Prepared according to General Procedure E with the *tert*-butyl (*R*,*E*)-(5-(2-bromophenyl)pent-4-en-2-yl)carbamate **21** (17 mg, 0.050 mmol, 1.0 eq.) and *N*-bromosuccinimide (11 mg, 0.060 mmol, 1.2 eq.) for 4 h. The crude was purified by column chromatography (basified silica, 0 to 20% ethyl acetate in hexane) to afford the desired product as off white solid (22 mg, 62%) as a single diastereomer.

Rotamer ratio determined from crude ^{1}H NMR – 1.0:0.47.

Major rotamer:

^{1}H NMR (400 MHz, CDCl$_3$) δ 7.66 – 7.56 (m, 1H), 7.32 – 7.25 (m, 1H), 7.20 – 7.11 (m, 1H), 7.05 – 6.99 (m, 1H), 5.56 – 5.51 (m, 1H), 4.52 – 4.42 (m, 1H), 4.29 (br s, 1H), 2.82 – 2.66 (m, 1H), 2.18 – 2.14 (m, 1H), 1.73 (s, 3H), 1.18 (s, 9H).

^{13}C{^{1}H} NMR (126 MHz, CDCl$_3$) δ 153.7, 141.2, 133.1, 129.1, 127.5, 126.0, 122.5, 79.8, 72.4, 55.0, 50.8, 39.0, 28.0, 21.2.

Minor rotamer:

^{1}H NMR (400 MHz, CDCl$_3$) δ 7.66 – 7.56 (m, 1H), 7.32 – 7.25 (m, 1H), 7.20 – 7.11 (m, 1H), 6.98 – 6.93 (m, 1H), 5.63 – 5.60 (m, 1H), 4.41 – 4.32 (m, 1H), 4.28 (br s, 1H), 2.82 – 2.66 (m, 1H), 2.14 – 2.10 (m, 1H), 1.72 (s, 3H), 1.51 (s, 9H).

^{13}C{^{1}H} NMR (126 MHz, CDCl$_3$) δ 153.7, 139.9, 133.6, 129.1, 127.5, 125.4, 122.9, 80.3, 72.3, 54.8, 50.1, 39.6, 28.5, 22.3.

IR (solid): 2924, 1688, 1364, 1165, 762 cm^{-1}.

HRMS (ESI): *m/z* calculated for $[M + H]^+$ $(C_{16}H_{22}Br_2N_1O_2)^+$: 418.0012; found 418.0011.

***tert*-Butyl 3-bromo-2-(4-chlorophenyl)-5-(*p*-tolyl)pyrrolidine-1-carboxylate (42)**

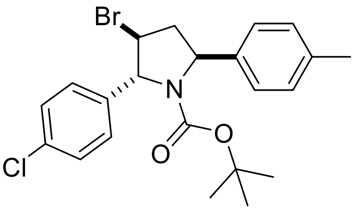

tert-butyl 3-bromo-2-(4-chlorophenyl)-5-(*p*-tolyl)pyrrolidine-1-carboxylate
Chemical Formula: $C_{22}H_{25}BrClNO_2$
Molecular Weight: 450.80

Prepared according to General Procedure E with the *tert*-butyl (*E*)-(4-(4-chlorophenyl)-2-(p-tolyl)but-3-en-1-yl)carbamate **23** (74 mg, 0.20 mmol, 1.0 eq.) and *N*-bromosuccinimide (43 mg, 0.24 mmol, 1.2 eq.) in MeCN (2 mL) for 4 h. The crude was purified by column chromatography (basified silica, 0 to 5% ethyl acetate in hexane) to afford the desired product as a white solid (75 mg, 82%) as a single diastereomer.

Unable to determine the rotamer ratio from crude ^{1}H NMR due to signal overlap.

Major rotamer:

^{1}H NMR (400 MHz, CDCl$_3$) δ 7.43 – 7.32 (m, 2H), 7.32 – 7.22 (m, 4H), 7.22 – 7.13 (m, 2H), 5.43 – 5.06 (m, 2H), 4.22 – 4.03 (m, 1H), 3.15 – 2.98 (m, 1H), 2.44 – 2.31 (m, 4H), 1.12 (s, 9H).

^{13}C{^{1}H} NMR (126 MHz, CDCl$_3$) δ 153.3, 140.9, 139.3, 136.6, 133.6, 129.2, 129.0, 127.2, 125.7, 80.4, 72.5, 62.0, 51.7, 43.2, 28.0, 21.2.

Minor rotamer:

^{1}H NMR (400 MHz, CDCl$_3$) δ 7.43 – 7.32 (m, 2H), 7.32 – 7.22 (m, 4H), 7.22 – 7.13 (m, 2H), 5.43 – 5.06 (m, 2H), 4.22 – 4.03 (m, 1H), 3.15 – 2.98 (m, 1H), 2.44 – 2.31 (m, 4H), 1.10 (s, 9H).

^{13}C{^{1}H} NMR (126 MHz, CDCl$_3$) δ 153.3, 140.7, 139.4, 136.6, 133.6, 129.2, 128.9, 127.0, 126.2, 80.4, 71.8, 62.4, 51.1, 44.0, 28.0, 21.2.

IR (solid): 2947, 2920, 2853, 1694, 1375, 1364, 1163, 814 cm^{-1}.

HRMS (ESI): *m/z* calculated for [M + H]$^+$ ($C_{22}H_{26}Br_1Cl_1N_1O_2$)$^+$: 450.0830; found 450.0826.

mp 117 – 120 °C

***tert*-Butyl 3-bromo-2-phenylhexahydrocyclopenta[*b*]pyrrole-1(2*H*)-carboxylate (43)**

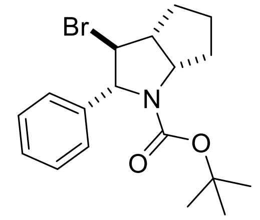

tert-butyl 3-bromo-2-phenylhexahydrocyclopenta[*b*]pyrrole-1(2*H*)-carboxylate
Chemical Formula: $C_{18}H_{24}BrNO_2$
Molecular Weight: 366.30

Prepared according to General Procedure E with the *tert*-butyl (*E*)-(2styrylcyclopentyl)carbamate **25** (40 mg, 0.14 mmol, 1.0 eq.) and *N*-bromosuccinimide (30 mg, 0.17 mmol, 1.2 eq.) for 16 h. The crude was purified by column chromatography (basified silica, 0 to 5% ethyl acetate in hexane) to afford the desired product as a colourless oil (36 mg, 70%) as a single diastereomer.

Rotamer ratio determined from crude ^{1}H NMR – 1.0:0.46.

Major rotamer:

^{1}H NMR (500 MHz, CDCl$_3$) δ 7.37 – 7.27 (m, 5H), 5.11 (d, *J* = 7.7 Hz, 1H), 3.79 (dd, *J* = 11.5, 7.7 Hz, 1H), 3.76 – 3.57 (m, 1H), 2.53 – 2.43 (m, 1H), 2.42 – 2.34 (m, 1H), 2.27 – 2.06 (m, 2H), 1.86 – 1.76 (m, 1H), 1.75 – 1.62 (m, 1H), 1.47 – 1.16 (m, 1H), 1.05 (s, 9H).

^{13}C{^{1}H} NMR (126 MHz, CDCl$_3$) δ 153.3, 141.7, 128.5, 127.7, 126.3, 79.7, 78.7, 67.9, 59.6, 53.6, 28.4, 28.0, 26.3, 21.4.

Minor rotamer:

^{1}H NMR (500 MHz, CDCl$_3$) δ 7.37 – 7.27 (m, 5H), 5.26 (d, *J* = 7.7 Hz, 1H), 3.76 – 3.57 (m, 2H), 2.53 – 2.43 (m, 1H), 2.42 – 2.34 (m, 1H), 2.27 – 2.06 (m, 2H), 1.86 – 1.76 (m, 1H), 1.75 – 1.62 (m, 1H), 1.47 – 1.16 (m, 1H), 1.39 (s, 9H).

^{13}C{^{1}H} NMR (126 MHz, CDCl$_3$) δ 152.6, 141.7, 128.7, 127.7, 125.7, 79.7, 77.8, 67.9, 60.1, 53.5, 28.6, 27.5, 26.0, 21.4.

IR (film): 2974, 2876, 1697, 1418, 1364, 1171, 1140 cm^{-1}.

HRMS (ESI): *m/z* calculated for [M + Na]$^+$ ($C_{18}H_{24}Br_1N_1O_2Na_1$)$^+$: 388.08826; found 388.08726.

***tert*-Butyl 3a-bromo-2-methyl-2,3,3a,4,5,9b-hexahydro-1H-benzo[g]indole-1-carboxylate (44)**

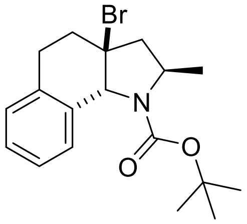

tert-butyl 3a-bromo-2-methyl-2,3,3a,4,5,9b-hexahydro-1*H*-benzo[*g*]indole-1-carboxylate
Chemical Formula: $C_{18}H_{24}BrNO_2$
Molecular Weight: 366.30

Prepared according to General Procedure E with *tert*-butyl (*R*)-(1-(3,4-dihydronaphthalen-2-yl)propan-2-yl)carbamate **26** (19 mg, 0.050 mmol, 1.0 eq.), *N*-bromosuccinimide (11 mg, 0.060 mmol, 1.2 eq.). and MeCN (0.5 mL) for 4 h. The crude was purified by column chromatography (silica, 0 to 20% ethyl acetate in hexane) to afford the desired product as a colourless oil (6.7 mg, 36%) as a single diastereomer.

^{1}H NMR (500 MHz, $CDCl_3$) δ 7.39 (br s, 1H), 7.21 – 7.14 (m, 2H), 7.14 – 7.06 (m, 1H), 5.32 (br s, 1H), 4.04 – 3.90 (m, 1H), 2.90 – 2.73 (m, 3H), 2.50 – 2.39 (m, 1H), 2.32 – 2.26 (m, 1H), 2.25 – 2.19 (m, 1H), 1.71 (d, *J* = 6.5 Hz, 3H), 1.50 (s, 9H).

^{13}C{^{1}H} NMR (126 MHz, $CDCl_3$) δ 136.3, 127.7, 127.2, 126.5, 80.0, 69.8, 68.2, 52.8, 47.2, 39.2, 28.5, 27.8, 21.0.

IR (film): 2972, 2928, 1684, 1389, 1169, 1134, 1047 cm^{-1}.

HRMS (ESI): *m/z* calculated for $[M + Na]^+$ $(C_{18}H_{24}Br_1N_1O_2Na_1)^+$: 388.08826; found 388.08718.

Epoxidation

***tert*-Butyl (2-(3-phenyloxiran-2-yl)ethyl) carbamate (45)**

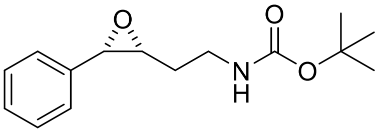

tert-butyl (2-(3-phenyloxiran-2-yl)ethyl)carbamate
Chemical Formula: $C_{15}H_{21}NO_3$
Molecular Weight: 263.34

Prepared according to General Procedure F with *tert*-butyl (*E*)-(4-phenylbut-3-en-1-yl)carbamate **17** (31 mg, 0.13 mmol, 1.0 eq.) and *m*CPBA (69 mg, 0.40 mmol, 3.0 eq.), in THF (1.3 mL). The crude was purified by column chromatography (basified silica, 0 to 10% ethyl acetate in cyclohexane) to afford the desired product as a colourless oil (21 mg, 61%) as a single diastereomer.

^{1}H NMR (500 MHz, $CDCl_3$) δ 7.36 – 7.27 (m, 3H), 7.27 – 7.24 (m, 2H), 4.82 (s, 1H), 3.71 – 3.63 (m, 1H), 3.43 – 3.27 (m, 2H), 3.05 – 2.99 (m, 1H), 2.10 – 1.99 (m, 1H), 1.77 (dq, *J* = 14.3, 6.5 Hz, 1H), 1.44 (s, 9H).

^{13}C{^{1}H} NMR (126 MHz, $CDCl_3$) δ 155.9, 137.2, 128.5, 128.2, 125.6, 79.4, 61.2, 58.1, 37.8, 32.3, 28.4.

IR (film): 3410, 2974, 2922, 2853, 1692, 1514, 1366, 1250, 1167 cm^{-1}.

HRMS (ESI): *m/z* calculated for $[M + H]^+$ $(C_{15}H_{22}N_1O_3)^+$: 264.1594; found 264.1600.

tert-Butyl ((2*R*)-1-(3-phenyloxiran-2-yl)propan-2-yl) carbamate (46)

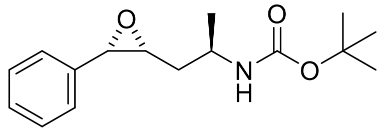

tert-butyl ((2*R*)-1-(3-phenyloxiran-2-yl)propan-2-yl)carbamate
Chemical Formula: $C_{16}H_{23}NO_3$
Molecular Weight: 277.36

Prepared to General Procedure F with *tert*-butyl (*E*)-(5-phenylpent-4-en-2-yl)carbamate **20** (78.4 mg, 0.300 mmol, 1.00 eq.) and *m*CPBA (0.155 g, 0.900 mmol, 3.00 eq.) in THF (3 mL). The crude was purified by column chromatography (basified silica, 0 to 20% ethyl acetate in cyclohexane) to afford the desired product as a white solid (42 mg, 50%).

Unable to determine the diastereomer ratio from crude ^{1}H NMR due to signal overlap.

Major diastereomer:

^{1}H NMR (400 MHz, $CDCl_3$) δ 7.40 – 7.25 (m, 5H), 4.62 (br s, 1H), 4.02 – 3.84 (m, 1H), 3.67 – 3.60 (m, 1H), 3.11 – 3.00 (m, 1H), 2.01 – 1.87 (m, 1H), 1.87 – 1.71 (m, 1H), 1.46 (s, 9H), 1.25 (d, *J* = 6.7 Hz, 3H).

$^{13}C\{^1H\}$ NMR (126 MHz, $CDCl_3$) δ 155.3, 137.4, 128.5, 128.2, 125.6, 79.3, 60.5, 58.5, 45.0, 39.5, 28.4, 21.3.

Minor diastereomer:

^{1}H NMR (400 MHz, $CDCl_3$) δ 7.40 – 7.25 (m, 5H), 4.67 (br s, 1H), 4.02 – 3.84 (m, 1H), 3.67 – 3.60 (m, 1H), 3.11 – 3.00 (m, 1H), 2.01 – 1.87 (m, 1H), 1.87 – 1.71 (m, 1H), 1.42 (s, 9H), 1.28 (d, *J* = 6.8 Hz, 3H).

$^{13}C\{^1H\}$ NMR (126 MHz, $CDCl_3$) δ 155.3, 137.4, 128.1, 126.5, 125.6, 79.3, 60.3, 57.9, 45.0, 39.5, 28.4, 21.1.

IR (film): 3364, 2959, 2924, 2851, 1715, 1506, 1366, 1271, 1171 cm^{-1}.

HRMS (ESI): *m/z* calculated for $[M + Na]^+$ $(C_{16}H_{23}N_1O_3Na_1)^+$: 300.1570; found 300.1568.

tert-Butyl (2-(3-phenyloxiran-2-yl)-1-(p-tolyl)ethyl) carbamate (47)

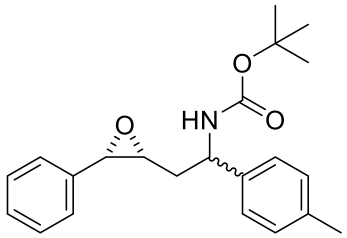

tert-butyl (2-(3-phenyloxiran-2-yl)-1-(*p*-tolyl)ethyl)carbamate
Chemical Formula: $C_{22}H_{27}NO_3$
Molecular Weight: 353.46

Prepared to General Procedure F with *tert*-butyl (*E*)-(4-phenyl-1-(*p*-tolyl)but-3-en-1-yl)carbamate **22** (67.5 mg, 0.200 mmol, 1.00 eq.) and *m*CPBA (0.104 g, 0.600 mmol, 3.00 eq.) in THF (2 mL). The crude was purified by column chromatography (silica, 0 to 10% ethyl acetate in cyclohexane) to afford the desired product as a white solid (55 mg, 77%).

Diastereomer ratio determined from crude ^{1}H NMR – 1.0:0.70.

Major diastereomer:

^{1}H NMR (500 MHz, CDCl$_3$) δ 7.33 – 7.26 (m, 5H), 7.23 – 7.09 (m, 4H), 5.29 (br s, 1H), 4.96 (br s, 1H), 3.55 (d, *J* = 2.0 Hz, 1H), 2.95 (br s, 1H), 2.33 (s, 3H), 2.25 – 2.07 (m, 2H), 1.40 (s, 9H).

^{13}C{^{1}H} NMR (126 MHz, CDCl$_3$) δ 155.1, 138.6, 137.2, 137.1, 129.4, 128.4, 128.2, 126.1, 125.6, 79.4, 59.9, 58.5, 53.1, 40.0, 28.4, 21.1.

Minor diastereomer:

^{1}H NMR (500 MHz, CDCl$_3$) δ 7.23 – 7.09 (m, 9H), 5.08 (br s, 1H), 4.86 (br s, 1H), 3.55 (d, *J* = 2.0 Hz, 1H), 2.95 (br s, 1H), 2.35 (s, 3H), 2.25 – 2.07 (m, 2H), 1.42 (s, 9H).

^{13}C{^{1}H} NMR (126 MHz, CDCl$_3$) δ 155.1, 138.7, 137.1, 137.0, 129.5, 128.3, 128.1, 126.0, 125.5, 79.4, 60.5, 58.1, 52.7, 39.1, 28.4, 21.1.

IR (solid): 3362, 2920, 2853, 1682, 1520, 1250, 1169, 818 cm^{-1}.

HRMS (ESI): *m/z* calculated for [M + Na]$^+$ ($C_{22}H_{27}N_1O_3Na_1$)$^+$: 376.1883; found 376.1884.

tert-Butyl (2-(3-(4-chlorophenyl)oxiran-2-yl)-1-(p-tolyl)ethyl)carbamate (48)

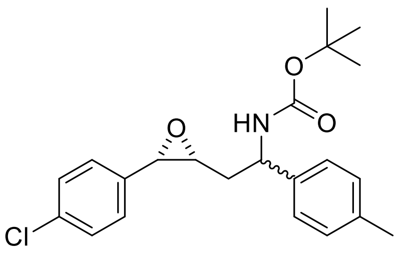

tert-butyl (2-(3-(4-chlorophenyl)oxiran-2-yl)-1-(*p*-tolyl)ethyl)carbamate
Chemical Formula: $C_{22}H_{26}ClNO_3$
Molecular Weight: 387.90

Prepared to General Procedure F with *tert*-butyl (*E*)-(4-(4-chlorophenyl)-2-(*p*-tolyl)but-3-en-1-yl)carbamate **23** (74.4 mg, 0.200 mmol, 1.00 eq.) and *m*CPBA (0.104 g, 0.600 mmol, 3.00 eq.) in THF (2 mL). The crude was purified by column chromatography (silica, 0 to 10%, ethyl acetate in hexane) to afford the desired product as a white solid (42 mg, 60%).

Diastereomer ratio determined from crude ^{1}H NMR – 1.0:0.70.

Major diastereomer:

^{1}H NMR (400 MHz, CDCl$_3$) δ 7.33 – 7.24 (m, 2H), 7.24 – 7.11 (m, 5H), 7.06 – 6.99 (m, 1H), 5.26 (br s, 1H), 4.96 (br s, 1H), 3.65 – 3.61 (m, 1H), 2.91 (br s, 1H), 2.36 (s, 3H), 2.24 – 2.09 (m, 3H), 1.42 (s, 9H).

^{13}C{^{1}H} NMR (126 MHz, CDCl$_3$) δ 155.1, 138.6, 137.1, 135.7, 133.9, 129.5, 128.6, 127.0, 126.2, 79.6, 60.1, 58.0, 52.7, 39.8, 28.4, 21.1.

Minor diastereomer:

^{1}H NMR (400 MHz, CDCl$_3$) δ 7.33 – 7.24 (m, 2H), 7.24 – 7.11 (m, 5H), 7.06 – 6.99 (m, 1H), 5.08 (br s, 1H), 4.86 (br s, 1H), 3.43 – 3.39 (m, 1H), 2.91 (br s, 1H), 2.36 (s, 3H), 2.24 – 2.09 (m, 3H), 1.42 (s, 9H).

^{13}C{^{1}H} NMR (126 MHz, CDCl$_3$) δ 155.1, 139.0, 137.3, 135.8, 133.8, 129.5, 128.5, 126.9, 126.1, 79.6, 60.6, 57.6, 53.0, 39.1, 28.4, 21.1.

IR (solid): 1674, 1541, 1493, 1290, 1169, 1013, 812, 797 cm^{-1}.

HRMS (ESI): *m/z* calculated for $[M + H]^+$ $(C_{22}H_{27}Cl_1N_1O_3)^+$: 388.1674; found 388.1667.

***tert*-Butyl (2-(3-phenyloxiran-2-yl)cyclopentyl) carbamate (49)**

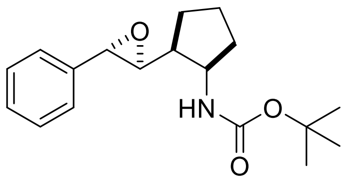

tert-butyl (2-(3-phenyloxiran-2-yl)cyclopentyl)carbamate
Chemical Formula: $C_{18}H_{25}NO_3$
Molecular Weight: 303.40

Prepared to General Procedure F with *tert*-butyl (*E*)-(2-styrylcyclopentyl)carbamate **25** (57.5 mg, 0.200 mmol, 1.00 eq.) and *m*CPBA (0.104 g, 0.600 mmol, 3.00 eq.) in THF (2 mL). The crude was purified by column chromatography (silica, 0 to 10% ethyl acetate in cyclohexane) to afford the desired product as a white solid (46 mg, 75%).

Diastereomer ratio determined from crude ^{1}H NMR – 1.0:0.40.

Major diastereomer:

^{1}H NMR (500 MHz, CDCl$_3$) δ 7.38 – 7.26 (m, 5H), 4.57 (br s, 1H), 3.96 – 3.88 (m, 1H), 3.71 (br s, 1H), 3.02 (dd, *J* = 6.1, 2.1 Hz, 1H), 2.15 – 2.05 (m, 1H), 2.00 – 1.87 (m, 1H), 1.86 – 1.77 (m, 1H), 1.77 – 1.66 m, 2H), 1.66 – 1.53 (m, 1H), 1.52 – 1.40 (m, 1H), 1.44 (s, 9H).

^{13}C{^{1}H} NMR (126 MHz, CDCl$_3$) δ 155.6, 137.5, 128.5, 128.0, 125.7, 79.0, 64.5, 57.8, 54.9, 48.9, 33.0, 28.4, 26.7, 22.4.

Minor diastereomer:

^{1}H NMR (500 MHz, CDCl$_3$) δ 7.38 – 7.32 (m, 2H), 7.32 – 7.26 (m, 3H), 4.74 (br s, 1H), 3.88 – 3.81 (m, 1H), 3.70 (br s, 1H), 3.01 – 2.98 (m, 1H), 2.15 – 2.05 (m, 1H), 2.00 – 1.87 (m, 1H), 1.86 – 1.77 (m, 1H), 1.77 – 1.66 m, 2H), 1.66 – 1.53 (m, 1H), 1.52 – 1.40 (m, 1H), 1.49 (s, 9H).

^{13}C{^{1}H} NMR (126 MHz, $CDCl_3$) δ 155.6, 137.5, 128.4, 128.1, 125.6, 79.0, 64.5, 56.8, 55.3, 48.2, 33.3, 28.5, 26.9, 22.4.

IR (solid): 3345, 2924, 2864, 1674, 1530, 1300, 1167, 870 cm^{-1}.

HRMS (ESI): *m/z* calculated for $[M + H]^+$ $(C_{18}H_{26}N_1O_3)^+$: 304.1907; found 304.1914.

tert-Butyl ((3-(4-chlorophenyl)oxiran-2-yl)methyl)carbamate (50)

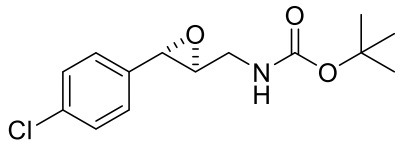

tert-butyl ((3-(4-chlorophenyl)oxiran-2-yl)methyl)carbamate
Chemical Formula: $C_{14}H_{18}ClNO_3$
Molecular Weight: 283.75

Prepared to General Procedure F with *tert*-butyl (*E*)-(3-(4-chlorophenyl)allyl)carbamate **7** (0.107 g, 0.400 mmol, 1.00 eq.) and *m*CPBA (0.210 g, 1.20 mmol, 3.00 eq.) in THF (4 mL). The crude was purified by column chromatography (basified silica, 0 to 20% ethyl acetate in cyclohexane) to afford the desired product as a colourless oil (60 mg, 53%) as a single diastereomer.

^{1}H NMR (500 MHz, $CDCl_3$) δ 7.31 – 7.27 (m, 2H), 7.18 – 7.15 (m, 2H), 4.89 (t, *J* = 6.3 Hz, 1H), 3.70 (d, *J* = 2.0 Hz, 1H), 3.61 – 3.53 (m, 1H), 3.42 – 3.34 (m, 1H), 3.11 – 3.06 (m, 1H), 1.45 (s, 9H).

^{13}C{^{1}H} NMR (126 MHz, $CDCl_3$) δ 155.9, 135.2, 134.1, 128.7, 127.0, 79.8, 61.1, 56.1, 41.3, 28.4.

IR (film): 3354, 2976, 2931, 1697, 1495, 1366, 1163, 1090, 818 cm^{-1}.

HRMS (ESI): *m/z* calculated for $[M + Na]^+$ $(C_{14}H_{18}Cl_1N_1O_3Na_1)^+$: 306.0867; found 306.0864.

tert-Butyl ((3-phenyloxiran-2-yl)methyl)carbamate (51)

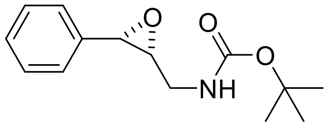

tert-butyl ((3-phenyloxiran-2-yl)methyl)carbamate
Chemical Formula: $C_{14}H_{19}NO_3$
Molecular Weight: 249.31000

Prepared to General Procedure F with *tert*-butyl cinnamylcarbamate **3** (46.7 mg, 0.200 mmol, 1.00 eq.) and *m*CPBA (0.104 g, 0.600 mmol, 3.00 eq.) in THF (2 mL). The crude was purified by column chromatography (silica, 0 to 20% ethyl acetate in hexane) to afford the desired product as a white solid (0.038 g, 75%) as a single diastereomer.

^{1}H NMR (500 MHz, $CDCl_3$) δ 7.39 – 7.23 (m, 5H), 3.74 (d, *J* = 2.0 Hz, 1H), 3.64 – 3.55 (m, 1H), 3.44 – 3.35 (m, 1H), 3.17 – 3.12 (m, 1H), 1.46 (s, 9H).

^{13}C{^{1}H} NMR (126 MHz, $CDCl_3$) δ 155.9, 136.6, 128.5, 128.3, 125.7, 61.0, 56.7, 41.5, 28.4.

IR (solid): 2978, 1690, 1508, 1366, 1248, 1163, 696 cm^{-1}.

HRMS (ESI): *m/z* calculated for $[M + H]^+$ $(C_{14}H_{20}N_1O_3)^+$: 250.1438; found 250.1437.

tert-Butyl (3-phenyloxirane-2-carbonyl)carbamate (52)

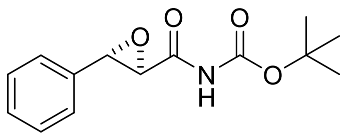

tert-butyl (3-phenyloxirane-2-carbonyl)carbamate
Chemical Formula: $C_{14}H_{17}NO_4$
Molecular Weight: 263.29

Prepared according to General Procedure F with *tert*-butyl cinnamoylcarbamate **52a** (248 mg, 1.00 mmol, 1.00 eq.) and *m*CPBA (0.520 g, 3.00 mmol, 3.00 eq.) in THF (10 mL). The crude was purified by column chromatography (basified silica, 0 to 20% ethyl acetate in cyclohexane) to afford the desired product as a white solid (213 mg, 80%) as a single diastereomer.

^{1}H NMR (500 MHz, $CDCl_3$) δ 7.83 (d, *J* = 15.8 Hz, 1H), 7.80 (br s, 1H), 7.61 – 7.56 (m, 2H), 7.52 (d, *J* = 15.7 Hz, 1H), 7.39 – 7.35 (m, 3H), 1.52 (s, 9H).

^{13}C{^{1}H} NMR (126 MHz, $CDCl_3$) δ 166.7, 150.8, 145.7, 134.6, 130.5, 128.9, 128.5, 118.4, 82.6, 28.1.

IR (solid): 3289, 2924, 1737, 1624, 1495, 1248, 1128, 980, 837 cm^{-1}.

HRMS (ESI): *m/z* calculated for $[M]^-$ $(C_{14}H_{17}N_1O_4)^-$: 263.1163; found 263.1173.

Epoxide Opening

5-Hydroxy-6-phenyl-1,3-oxazinan-2-one (53)

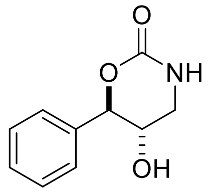

5-hydroxy-6-phenyl-1,3-oxazinan-2-one
Chemical Formula: $C_{10}H_{11}NO_3$
Molecular Weight: 193.20

Prepared according to General Procedure G with *tert*-butyl ((3-phenyloxiran-2-yl)methyl)carbamate **51** (50 mg, 0.20 mmol, 1.0 eq.) and boron trifluoride diethyl etherate (25 µL, 0.20 mmol, 1.0 eq.) in Et_2O (2 mL) for 4 h. The precipitate was filtered to afford the desired product as a white solid (13 mg, 33%) as a single diastereomer.

^{1}H NMR (400 MHz, acetone-d_6) δ 7.44 – 7.31 (m, 5H), 6.41 (br s, 1H), 5.21 (dd, *J* = 5.9, 1.0 Hz, 1H), 4.73 (d, *J* = 4.9 Hz, 1H), 4.15 – 4.05 (m, 1H), 3.34 (ddd, *J* = 11.8, 4.5, 2.5 Hz, 1H), 3.25 (dddd, *J* = 11.8, 6.0, 2.6, 1.0 Hz, 1H).

^{13}C{^{1}H} NMR (126 MHz, acetone-d_6) δ 153.2, 139.4, 129.3, 128.9, 127.2, 83.4, 66.0, 45.2.

IR (solid): 3281, 3198, 2916, 2851, 1684, 1481, 1092 cm^{-1}.

HRMS (ESI): *m/z* calculated for $[M + H]^+$ $(C_{10}H_{12}N_1O_3)^+$: 194.0812; found 194.0815.

6-(4-Chlorophenyl)-5-hydroxy-1,3-oxazinan-2,4-dione (54)

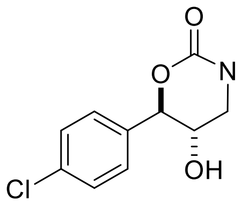

6-(4-chlorophenyl)-5-hydroxy-1,3-oxazinan-2-one
Chemical Formula: $C_{10}H_{10}ClNO_3$
Molecular Weight: 227.64

Prepared according to General Procedure G with t*ert*-butyl (*E*)-(3-(4-chlorophenyl)allyl)carbamate **50** (60 mg, 0.20 mmol, 1.0 eq.) boron trifluoride diethyl etherate (24 µL, 0.30 mmol, 1.0 eq.) in diethyl ether (2 mL). The crude was filtered to afford the desired product as a white solid (34 mg, 74%) as a single diastereomer.

^{1}H NMR (500 MHz, Acetone-*d*$_6$) δ 7.53 – 7.32 (m, 4H), 6.49 (s, 1H), 5.16 (d, *J* = 6.5 Hz, 1H), 4.08 – 4.03 (m, 1H), 3.38 – 3.32 (m, 1H), 3.26 – 3.20 (m, 1H).

^{13}C{^{1}H} NMR (126 MHz, acetone-*d*$_6$) δ 152.4, 137.3, 133.4, 128.5, 128.3, 81.8, 65.0, 44.5.

IR (solid): 3327, 3269, 1678, 1481, 1416, 1273, 1136, 1088, 1011, 829 cm^{-1}.

HRMS (LDI): *m/z* calculated for $[M + H]^+$ $(C_{10}H_{11}Cl_1N_1O_3)^+$: 228.0422; found 228.0415.

5-Hydroxy-6-phenyl-1,3-oxazinane-2,4-dione (55)

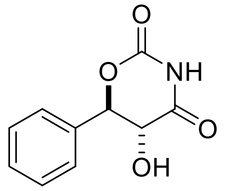

5-hydroxy-6-phenyl-1,3-oxazinane-2,4-dione
Chemical Formula: $C_{10}H_9NO_4$
Molecular Weight: 207.19

Prepared according to General Procedure G with *tert*-butyl (3-phenyloxirane-2-carbonyl)carbamate **52** (79 mg, 0.30 mmol, 1.0 eq.) boron trifluoride diethyl etherate (72 µL, 0.60 mmol, 2.0 eq.) in diethyl ether (3 mL). The crude was filtered to afford the desired product as a white solid (36 mg, 58%).

Diastereomer ratio determined from crude ^{1}H NMR – 1.0:0.10.

Major diastereomer:

^{1}H NMR (500 MHz, acetone-*d*$_6$) δ 9.59 (s, 1H), 8.41 (s, 1H), 8.00 – 7.92 (m, 1H), 7.79 – 7.71 (m, 2H), 7.55 – 7.47 (m, 3H), 6.93 (d, *J* = 15.7 Hz, 1H).

^{13}C{^{1}H} NMR (126 MHz, acetone-*d*$_6$) δ 170.1, 147.6, 142.2, 133.5, 131.6, 129.2, 128.9, 115.6.

IR (solid): 3368, 3200 (br), 1676, 1454, 1028, 864 cm^{-1}.

HRMS (ESI): *m/z* calculated for $[M + H]^+$ $(C_{10}H_{10}N_1O_4)^+$: 208.0604; found 208.0598.

***tert*-Butyl 3-hydroxy-2-phenyl-5-(*p*-tolyl)pyrrolidine-1-carboxylate (56)**

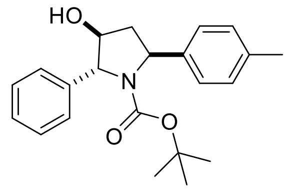

tert-butyl 3-hydroxy-2-phenyl-5-(*p*-tolyl)pyrrolidine-1-carboxylate
Chemical Formula: $C_{22}H_{27}NO_3$
Molecular Weight: 353.46

Prepared according to General Procedure G with *tert*-butyl (2-(3-phenyloxiran-2-yl)-1-(*p*-tolyl)ethyl) carbamate **47** (42 mg, 0.12 mmol, 1.0 eq.), boron trifluoride diethyl etherate (15 μL, 0.12 mmol, 1.0 eq.) in diethyl ether (1.2 mL). The crude was purified by column chromatography (basified silica, 0 to 15% ethyl acetate in hexane) to afford the desired product as a white solid (26 mg, 61%) as a single diastereomer.

Rotamer ratio determined from crude ^{1}H NMR – 1.0:0.73.

Major rotamer:

^{1}H NMR (500 MHz, $CDCl_3$) δ 7.37 – 7.32 (m, 2H), 7.29 – 7.21 (m, 5H), 7.19 – 7.12 (m, 2H), 5.39 – 5.35 (m, 1H), 5.08 – 5.06 (m, 1H), 4.20 – 4.13 (m, 1H), 2.75 – 2.62 (m, 1H), 2.32 (s, 3H), 1.98 – 1.92 (m, 1H), 1.14 (s, 9H).

^{13}C{^{1}H} NMR (126 MHz, $CDCl_3$) δ 154.2, 142.0, 140.9, 136.4, 129.5, 128.5, 127.1, 125.5, 125.1, 79.9, 79.7, 72.4, 60.7, 40.3, 28.0, 21.1.

Minor rotamer:

^{1}H NMR (500 MHz, $CDCl_3$) δ 7.37 – 7.32 (m, 2H), 7.29 – 7.21 (m, 5H), 7.19 – 7.12 (m, 2H), 5.23 – 5.21 (m, 1H), 5.21 – 5.18 (m, 1H), 4.20 – 4.13 (m, 1H), 2.75 – 2.62 (m, 1H), 2.34 (s, 3H), 1.98 – 1.92 (m, 1H), 1.16 (s, 9H).

^{13}C{^{1}H} NMR (126 MHz, $CDCl_3$) δ 154.1, 142.1, 140.8, 136.2, 129.1, 128.7, 127.2, 125.6, 125.3, 79.8, 79.2, 71.7, 61.5, 40.8, 28.1, 21.1.

IR (solid): 3439, 2920, 2853, 1670, 1395, 1152, 1123, 903 cm^{-1}.

HRMS (ESI): *m/z* calculated for $[M + Na]^+$ $(C_{22}H_{27}N_1O_3Na_1)^+$: 376.1883; found 376.1881.

mp 152 – 156 °C

tert-Butyl 3-hydroxy-2-phenylpyrrolidine-1-carboxylate (57)

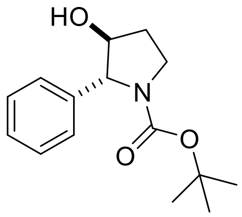

tert-butyl 3-hydroxy-2-phenylpyrrolidine-1-carboxylate
Chemical Formula: $C_{15}H_{21}NO_3$
Molecular Weight: 263.34

Prepared according to General Procedure G with *tert*-butyl (2-(3-phenyloxiran-2-yl)ethyl) carbamate **45** (40 mg, 0.15 mmol, 1.0 eq.), boron trifluoride diethyl etherate (18 μL, 0.150 mmol, 1.0 eq.) in diethyl ether (1.5 mL). The crude was purified by column chromatography (basified silica, 0 to 15% ethyl acetate in hexane) to afford the desired product as a white solid (11 mg, 27%) as a single diastereomer.

Rotamer ratio determined from crude ^{1}H NMR – 1.0:0.48.

Major rotamer:

^{1}H NMR (500 MHz, CDCl$_3$) δ 7.34 – 7.29 (m, 2H), 7.26 – 7.20 (m, 1H), 7.20 – 7.15 (m, 2H), 4.68 (s, 1H), 4.22 (s, 1H), 3.80 – 3.73 (m, 2H), 2.17 – 2.06 (m, 1H), 1.90 – 1.81 (m, 1H), 1.59 (br s), 1.20 (s, 9H).

^{13}C{^{1}H} NMR (126 MHz, CDCl$_3$) δ 154.7, 141.5, 128.4, 127.1, 125.6, 79.2, 78.3, 70.0, 44.5, 30.8, 28.1.

Minor rotamer:

^{1}H NMR (500 MHz, CDCl$_3$) δ 7.34 – 7.29 (m, 2H), 7.26 – 7.20 (m, 1H), 7.20 – 7.15 (m, 2H), 4.88 (s, 1H), 4.22 (s, 1H), 3.73 – 3.64 (m, 2H), 2.17 – 2.06 (m, 1H), 2.00 – 1.90 (m, 1H), 1.59 (br s), 1.47 (s, 9H).

^{13}C{^{1}H} NMR (126 MHz, CDCl$_3$) δ 154.7, 141.5, 128.6, 127.1, 125.5, 79.2, 78.3, 69.9, 45.0, 30.9, 28.5.

IR (solid): 2922, 2909, 1730, 1651, 1408, 1362, 1163, 1098, 972 cm^{-1}.

HRMS (ESI): *m/z* calculated for $[M + H]^+$ $(C_{15}H_{22}N_1O_3)^+$: 264.1594; found 264.1594.

tert-Butyl 3-hydroxy-2-phenylhexahydrocyclopenta[*b*]pyrrole-1(2*H*)-carboxylate (58)

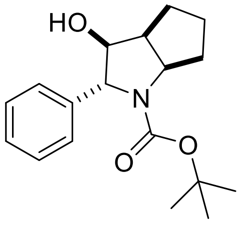

tert-butyl 3-hydroxy-2-phenylhexahydrocyclopenta[*b*]pyrrole-1(2*H*)-carboxylate
Chemical Formula: $C_{18}H_{25}NO_3$
Molecular Weight: 303.40

Prepared according to General Procedure G with *tert*-butyl (2-(3-phenyloxiran-2-yl)cyclopentyl) carbamate **49** (44 mg, 0.14 mmol, 1.0 eq.) boron trifluoride diethyl etherate (17 μL, 0.14 mmol, 1.0 eq.) in diethyl ether

(1.4 mL). The crude was purified by column chromatography (basified silica, 0 to 20% ethyl acetate in hexane) to afford the desired product as a colourless oil (3 mg, 8%) as a single diastereomer.

Rotamer ratio determined from crude ^{1}H NMR – 1.0:0.45.

Major rotamer:

^{1}H NMR (500 MHz, CDCl$_3$) δ 7.32 (m, 2H), 7.29 – 7.27 (m, 1H), 7.25 – 7.19 (m, 2H), 4.69 (d, J = 6.7 Hz, 1H), 3.96 – 3.57 (m, 2H), 2.31 – 2.21 (m, 1H), 2.20 – 2.03 (m, 3H), 1.86 – 1.76 (m, 1H), 1.71 – 1.55 (m, 1H), 1.37 – 1.26 (m, 1H), 1.04 (s, 9H).

^{13}C{^{1}H} NMR (126 MHz, CDCl$_3$) δ 153.8, 143.2, 128.5, 127.2, 126.1, 80.2, 79.3, 77.2, 66.7, 56.7, 28.0, 27.2, 26.8, 21.3.

Minor rotamer:

^{1}H NMR (500 MHz, CDCl$_3$) 7.32 (m, 2H), 7.29 – 7.27 (m, 1H), 7.25 – 7.19 (m, 2H), 4.82 (d, J = 6.7 Hz, 1H), 3.96 – 3.57 (m, 2H), 2.39 – 2.31 (m, 1H), 2.31 – 2.21 (m, 1H), 2.20 – 2.03 (m, 2H), 1.86 – 1.76 (m, 1H), 1.71 – 1.55 (m, 1H), 1.39 (s, 9H), 1.37 – 1.26 (m, 1H).

^{13}C{^{1}H} NMR (126 MHz, CDCl$_3$) δ 153.2, 142.0, 128.8, 125.8, 125.5, 80.2, 79.3, 77.2, 66.7, 57.0, 28.5, 28.4, 26.5, 21.4.

IR (film): 2974, 2928, 1697, 1670, 1427, 1364, 1148 cm^{-1}.

HRMS (ESI): *m/z* calculated for $[M + H]^+$ $(C_{18}H_{26}N_1O_3)^+$: 304.1907; found 304.1909.

tert-Butyl (2*R*,3*S*)-2-(4-chlorophenyl)-3-hydroxy-5-(*p*-tolyl)pyrrolidine-1-carboxylate (59)

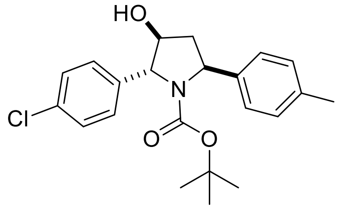

tert-butyl 2-(4-chlorophenyl)-3-hydroxy-5-(*p*-tolyl)pyrrolidine-1-carboxylate
Chemical Formula: $C_{22}H_{26}ClNO_3$
Molecular Weight: 387.90

Prepared according to General Procedure G with *tert*-butyl (2-(3-(4-chlorophenyl)oxiran-2-yl)-1-(*p*-tolyl)ethyl)carbamate **48** (56 mg, 0.15 mmol, 1.0 eq.) boron trifluoride diethyl etherate (18 µL, 0.14 mmol, 1.0 eq.) in diethyl ether (1.4 mL). The crude was purified by column chromatography (basified silica, 0 to 15% ethyl acetate in hexane) to afford the desired product as a cream solid (15 mg, 27%) as a single diastereomer.

Unable to determine the ratio from crude ^{1}H NMR due to signal overlap (all signals in sets of three).

Major rotamer:

^{1}H NMR (500 MHz, CDCl$_3$) δ 7.42 – 7.36 (m, 1H), 7.35 – 7.30 (m, 3H), 7.25 – 7.20 (m, 1H), 7.20 – 7.11 (m, 3H), 5.49 – 4.01 (m, 3H), 2.70 – 2.55 (m, 1H), 2.35 (s, 3H), 2.01 – 1.92 (m, 1H), 1.45 (s, 9H).

^{13}C{^{1}H} NMR (126 MHz, CDCl$_3$) δ 154.0, 141.8, 140.6, 136.5, 132.8, 129.6, 128.7, 126.9, 125.1, 80.0, 79.7, 71.7, 60.7, 40.2, 28.1, 21.1.

IR (film): 2976, 2926, 1697, 1674, 1491, 1395, 1366, 1171, 1157, 1015, 816 cm^{-1}.

HRMS (ESI): *m/z* calculated for [M + Na]$^+$ ($C_{22}H_{26}Cl_1N_1O_3Na_1$)$^+$: 410.1493; found 410.1496.

tert-Butyl (5*R*)-3-hydroxy-5-methyl-2-phenylpyrrolidine-1-carboxylate (60)

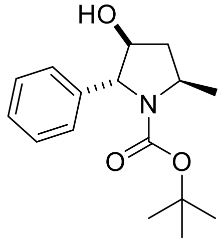

tert-butyl (5*R*)-3-hydroxy-5-methyl-2-phenylpyrrolidine-1-carboxylate
Chemical Formula: $C_{16}H_{23}NO_3$
Molecular Weight: 277.36

Prepared according to General Procedure G with *tert*-butyl (1-(3-phenyloxiran-2-yl)propan-2-yl) carbamate **46** (42 mg, 0.15 mmol, 1.0 eq.) boron trifluoride diethyl etherate (18 µL, 0.15 mmol, 1.0 eq.) in diethyl ether (1.5 mL). The crude was purified by column chromatography (basified silica, 0 to 15% ethyl acetate in cyclohexane) to afford the desired product as a white solid (7 mg, 17%) as a single diastereomer.

Rotamer ratio determined from crude ^{1}H NMR – 1.0:0.50.

Major rotamer:

^{1}H NMR (500 MHz, CDCl$_3$) δ 7.39 – 7.27 (m, 2H), 7.26 – 7.17 (m, 1H), 7.16 – 7.09 (m, 2H), 4.82 (s, 1H), 4.36 – 4.27 (m, 1H), 4.23 – 4.12 (m, 1H), 2.41 – 2.26 (m, 1H), 2.05 (s, 1H), 1.69 – 1.62 (m, 1H), 1.52 (d, J = 6.4 Hz, 3H), 1.13 (s, 9H).

^{13}C{^{1}H} NMR (126 MHz, CDCl$_3$) δ 154.3, 142.2, 128.4, 127.0, 125.5, 79.2, 79.2, 71.8, 54.0, 37.8, 28.1, 21.6.

Minor rotamer:

^{1}H NMR (500 MHz, CDCl$_3$) δ 7.39 – 7.27 (m, 2H), 7.26 – 7.17 (m, 1H), 7.16 – 7.09 (m, 2H), 4.98 (s, 1H), 4.36 – 4.27 (m, 1H), 4.23 – 4.12 (m, 1H), 2.41 – 2.26 (m, 1H), 2.05 (s, 1H), 1.69 – 1.62 (m, 1H), 1.51 (d, J = 6.2 Hz, 3H), 1.46 (s, 9H).

^{13}C{^{1}H} NMR (126 MHz, CDCl$_3$) δ 154.3, 140.8, 128.6, 127.0, 125.2, 79.6, 78.6, 71.5, 54.0, 38.1, 28.5, 22.5.

IR (solid): 3397, 2978, 2940, 1645, 1400, 1369, 1167, 1032, 851 cm^{-1}.

HRMS (ESI): *m/z* calculated for [M + Na]$^+$ ($C_{16}H_{23}N_1O_3Na_1$)$^+$: 300.1570; found 300.1567.

Miscellaneous

6-azabicyclo[3.2.0]heptan-7-one (M1)

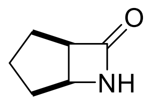

6-azabicyclo[3.2.0]heptan-7-one
Chemical Formula: C_6H_9NO
Molecular Weight: 111.14

According to the published procedure by Dener *et al.*[30]

In a flame-dried two-necked flask equipped with a Teflon-coated stirrer bar cyclopentene (4.5 mL, 51 mmol, 1.0 eq.) was dissolved in 23 mL anhydrous dichloromethane and cooled to 0 °C under nitrogen. Chlorosulfonyl isocyanate (4.3 mL, 49 mmol, 1.0 eq.) was dissolved in 7 mL anhydrous dichloromethane and was added dropwise to the cyclopentene solution with stirring over 30 minutes. The reaction was then heated to 40 °C and stirred for 20 h. The resulting solution was cooled to 0 °C and quenched with dropwise addition of water until bubbling ceased. Anhydrous sodium sulfite (15.8 g, 125 mmol, 2.50 eq.) and potassium phosphate (26.5 g, 125 mmol, 2.50 eq.) were dissolved in 240 mL water. This solution along with 120 mL dichloromethane was combined with the reaction solution and stirred for 24 h. The organics were collected and the aqueous phase was extracted with ethyl acetate (200 mL). The combined organic phases were dried with sodium sulfate and the solvents were removed *in vacuo*. The resulting solid was dissolved in ethyl acetate and recrystallized from hexane to afford white solid (2.1 g, 38%) as a single diastereomer.

^{1}H NMR (400 MHz, DMSO-*d*$_6$) δ 7.56 (br s, 1H), 3.92 – 3.87 (m, 1H), 2.38 – 2.33 (m, 1H), 1.81 – 1.22 (m, 6H).

Spectroscopic data in agreement with the literature.[30]

2-aminocyclopentane-1-carboxylic acid hydrochloride (M2)

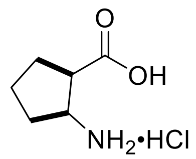

Chemical Formula: $C_6H_9O_2$
Molecular Weight: 113.14

According to the published procedure by Dener *et al.*[30]

6-azabicyclo[3.2.0]heptan-7-one **M1** (0.88 g, 7.9 mmol, 1.0 eq.) was dissolved in water (0.9 mL) and then the mixture was treated with concentrated HCl (0.9 mL). The reaction mixture was stirred for 2 h after which it was allowed to stand in the fridge for 2 h. The resulting precipitate was isolated by filtration to afford the desired product as a white solid (0.55 g, 3.4 mmol, 33%) as a single diastereomer, which was used without further purification.

^{1}H NMR (400 MHz, DMSO-*d*$_6$) δ 8.10 (s, 2H), 3.64 – 3.52 (m, 1H), 3.00 – 2.92 (m, 1H), 2.06 – 1.46 (m, 6H).

Spectroscopic data in agreement with the literature.[30]

Compounds **1** and **5** were also prepared according to the procedure by Zhang *et al.*[21]

To an oven-dried microwave vial phenylboronic acid (0.488 g, 4.00 mmol, 1.00 eq.), palladium acetate (45.0 mg, 0.2 mmol, 0.05 eq.), silver acetate (1.34 g, 8.00 mmol, 2.00 eq.) and potassium hydrogen difluoride (0.313 g, 4.00 mmol, 1.00 eq.) were added. The vial was capped, evacuated and refilled with Ar (3 times). Acetone (12 mL) and *N*-allylacetamide (0.79 g, 8.00 mmol, 2.00 eq.) or *tert*-butyl allylcarbamate (1.26 g, 8.00 mmol, 2.00 eq.) were added and the reaction mixture was stirred at 60 °C for 16 h. The reaction mixture was filtered through celite and the solvent was removed *in vacuo*. The crude was purified by flash column chromatography (basified silica, ethyl acetate in hexane) to afford the desired product as a white solid.

Product	Column solvent ratio	Mass	% yield
**1**	0 to 10%	0.53 g	57%
**5**	0 to 100%	0.30 g	43%

N-allylacetamide (M3)

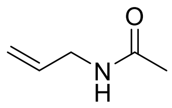

N-allylacetamide
Chemical Formula: C_5H_9NO
Molecular Weight: 99.13

According to the published procedure by Prediger *et al.*[31]

To a flame-dried round-bottom flask equipped with a Teflon-coated stir bar under argon atmosphere allylamine (0.58 g, 0.74 mL, 10 mmol, 1.0 eq.) and Et_3N (2.2 mL, 16 mmol, 1.6 eq.) were added followed by dissolved in CH_2Cl_2 (100 mL) and acetic anhydride (1.5 mL, 16 mmol, 1.6 eq.). The reaction mixture was stirred for 24 h at room temperature. After this time, saturated NH_4Cl (40 mL) was added and the mixture was extracted with CH_2Cl_2 (3 × 20 mL). The organic extracts were combined and dried over Na_2SO_4 and the solvent was removed *in vacuo* to afford a yellow oil which was purified by flash chromatography (basified silica, 10 to 40% ethyl acetate in hexane) to afford the desired product as a colourless oil (0.80 g, 80%).

^{1}H NMR (400 MHz, $CDCl_3$) δ 6.58 (s, 1H), 5.73 (ddt, J = 15.9, 10.2, 5.1 Hz, 1H), 5.05 (dd, J = 26.7, 13.7 Hz, 2H), 3.78 – 3.73 (m, 2H), 1.91 (s, 3H).

Spectroscopic data in agreement with the literatures.[32]

DFT Calculations

58 (*anti* and *syn* diastereomers)

NMR spectrum parameters DFT calculation using Spartan 24.2.0
NMR: ωB97X-D/6-31G*
Geometry: ωB97X-D/6-31G*
Energy & Weights: ωB97X-V/6-311+G(2df,2p)[6-311G*]
Multistep conformational run: MMFF94 (9 and 4 conformers)

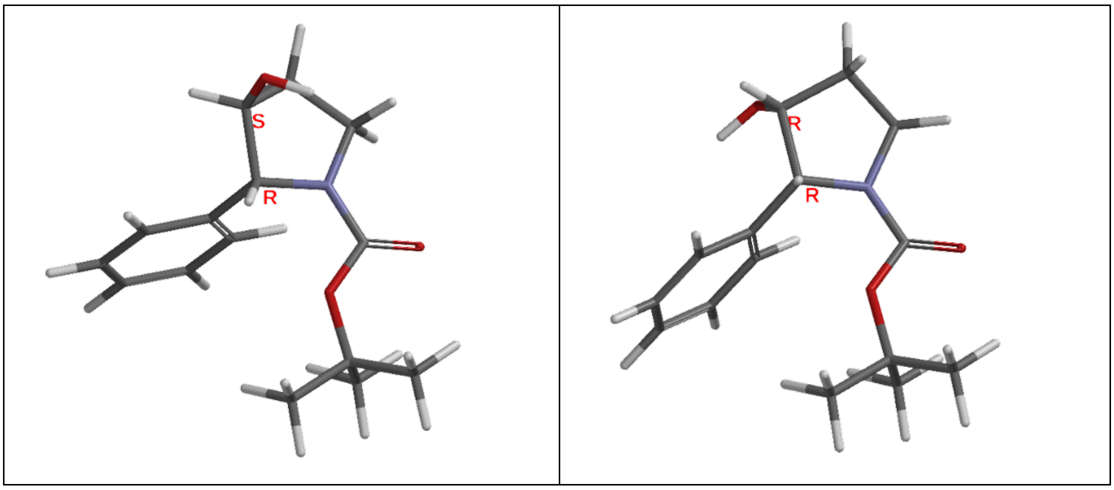

	Dihedral (H2-C2-C3-H3)	^{3}J(H2,H3) [Hz]
Experimental	-	0.0
anti – RS	−85.91	2.9
syn – RR	−34.67	5.3

Comparison of experimental and calculated ^{13}C chemical shifts [ppm]

	C2	C3	C4	C5	C10	RMS	DP4 score[33]
Experimental	70.0	79.2	30.8	44.5	141.3	-	-
anti – RS	71.2	80.3	30.4	45.1	142.7	1.02	99.4
syn – RR	67.9	74.0	31.8	45.6	139.7	2.7	0.6

anti

Cartesian coordinates

N	-1.485751	0.734678	-0.055402
C	-0.325520	1.614934	-0.169210
C	-0.937328	2.969027	0.253094
C	-1.917078	2.559035	1.359679
C	-2.522254	1.246870	0.842446
H	-0.001903	1.678820	-1.211798
H	-0.174497	3.668160	0.600028
H	-2.663698	3.334775	1.539962
H	-1.365854	2.381039	2.288479
H	-3.458514	1.405258	0.294324
H	-2.734594	0.526261	1.637128
C	-1.506125	-0.568433	-0.459291
O	-2.410180	-1.338878	-0.192537
O	-0.401735	-0.842347	-1.173862

C	-0.027249	-2.211589	-1.490318
C	0.087237	-3.035118	-0.207643
H	-0.889140	-3.178938	0.258555
H	0.753090	-2.531001	0.501705
H	0.513053	-4.016089	-0.442739
C	1.346250	-2.036389	-2.133266
H	2.037171	-1.564655	-1.427854
H	1.275437	-1.405577	-3.024743
H	1.750951	-3.009942	-2.426477
C	-1.024897	-2.812087	-2.478458
H	-2.009096	-2.917440	-2.019165
H	-0.675104	-3.798265	-2.801617
H	-1.110735	-2.171969	-3.362405
C	0.857716	1.199572	0.689014
C	3.079560	0.506721	2.250963
C	2.108056	1.767715	0.439650
C	0.733457	0.281484	1.730499
C	1.837274	-0.064903	2.505741
C	3.211833	1.427391	1.214457
H	2.218117	2.479577	-0.375269
H	-0.229049	-0.181099	1.930795
H	1.723913	-0.786670	3.309130
H	4.177772	1.876534	1.004716
H	3.940969	0.234652	2.853350
O	-1.581752	3.605777	-0.831621
H	-2.199800	2.973110	-1.220044

**Avg. Energy:**	-864.743220 hartrees (from 9 conformers)
**Post Energy:**	-864.743591 hartrees<br>ωB97X-V/6-311+G(2df,2p)

syn

Cartesian coordinates

N	0.582360	1.653861	0.172610
C	1.618072	0.628480	0.079801
C	2.775842	1.246975	0.911211
C	2.571035	2.745506	0.711546
C	1.050562	2.897726	0.776379
H	1.957548	0.529601	-0.960707
H	3.744193	0.889694	0.533928
H	2.942549	3.041465	-0.275503
H	3.095504	3.323080	1.475441

H	0.669382	3.753805	0.215808
H	0.702279	2.981134	1.811589
C	-0.650220	1.570704	-0.400024
O	-1.500466	2.437360	-0.303668
O	-0.766499	0.408935	-1.069197
C	-2.066160	-0.128064	-1.433577
C	-2.970678	-0.202748	-0.203915
H	-3.222318	0.794241	0.161550
H	-2.471260	-0.763283	0.593612
H	-3.896679	-0.725962	-0.464443
C	-1.717296	-1.531825	-1.921906
H	-1.238136	-2.104140	-1.120992
H	-1.028206	-1.481507	-2.770803
H	-2.624046	-2.056433	-2.239512
C	-2.681620	0.711904	-2.550282
H	-2.902239	1.719766	-2.194905
H	-3.608738	0.243771	-2.898484
H	-1.990348	0.777587	-3.396975
C	1.194658	-0.738883	0.570086
C	0.457151	-3.285699	1.472956
C	1.685813	-1.883298	-0.056498
C	0.338801	-0.881713	1.665224
C	-0.031338	-2.147117	2.110173
C	1.321765	-3.150387	0.390523
H	2.343431	-1.782282	-0.916631
H	-0.053984	0.007424	2.149870
H	-0.707007	-2.243594	2.955032
H	1.705755	-4.031875	-0.114450
H	0.163577	-4.272711	1.817452
O	2.652519	1.000644	2.293722
H	2.554444	0.047855	2.423960

**Avg. Energy:**	-864.744876 hartrees (from 4 conformers)
**Post Energy:**	-864.745326 hartrees<br>ωB97X-V/6-311+G(2df,2p)

60 (RRS and RRR diastereomers)

NMR spectrum parameters DFT calculation using Spartan 24.2.0

NMR: ωB97X-D/6-31G*

Geometry: ωB97X-D/6-31G*

Energy & Weights: ωB97X-V/6-311+G(2df,2p)[6-311G*]

Multistep conformational run: MMFF94 (7 and 3 conformers)

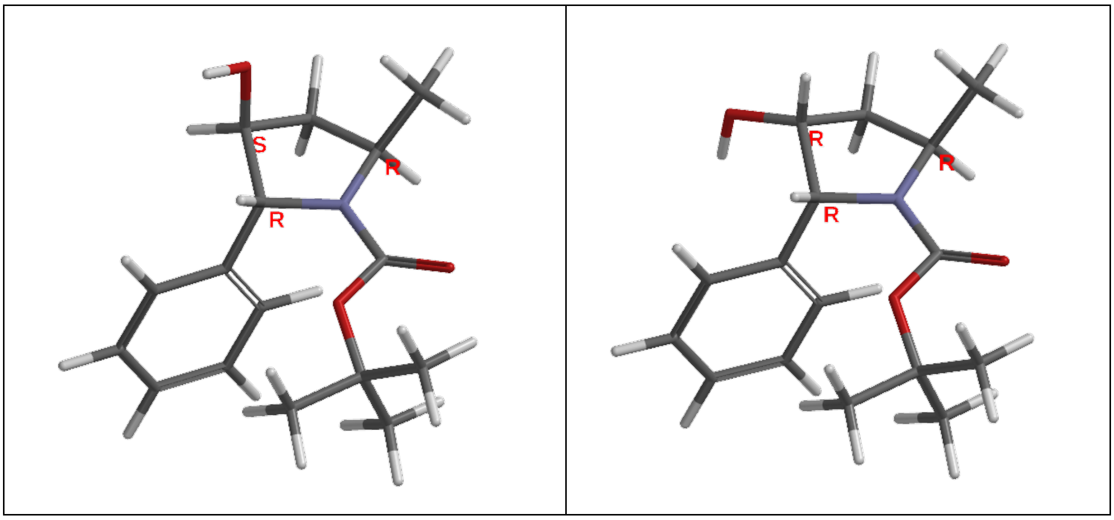

	Dihedral (H2-C2-C3-H3)	$^{3}J(H2,H3)$ [Hz]
Experimental	-	0.0
RRS	−92.5	1.3
RRR	34.6	6.7

Comparison of experimental and calculated ^{13}C chemical shifts [ppm]

	C2	C3	C4	C5	C10	C16	RMS	DP4 score[33]
Experimental	71.7	79.2	37.8	54.0	142.2	21.5	-	-
RRS	72.9	80.5	36.5	55.0	143.0	21.5	1.0	99.9
RRR	65.8	71.3	37.6	52.2	139.0	20.6	4.32	0.1

RRS

Cartesian coordinates

N	0.608987	1.261188	-0.019292
C	1.499552	0.113989	-0.116016
C	2.840518	0.684194	0.406663
C	2.397083	1.754450	1.400617
C	1.132709	2.374457	0.780102
H	1.610855	-0.191157	-1.163860
H	3.438335	-0.106082	0.881884
H	3.190282	2.487384	1.567670
H	2.159458	1.274933	2.356749
H	0.395559	2.622129	1.552337
C	-0.685867	1.248440	-0.440644
O	-1.484810	2.140242	-0.213429
O	-0.927811	0.117783	-1.130799
C	-2.283861	-0.314324	-1.415928

C	-3.088682	-0.413928	-0.121114
H	-3.261786	0.572478	0.309936
H	-2.553674	-1.033935	0.605515
H	-4.056490	-0.882186	-0.327454
C	-2.067126	-1.702426	-2.013047
H	-1.559045	-2.350353	-1.290872
H	-1.451726	-1.640099	-2.916470
H	-3.029117	-2.153545	-2.277274
C	-2.927772	0.627918	-2.429731
H	-3.052226	1.624787	-2.002971
H	-3.908416	0.238919	-2.725984
H	-2.302176	0.701218	-3.326107
C	1.032153	-1.091305	0.683833
C	0.237958	-3.345537	2.147489
C	1.428029	-2.369645	0.291565
C	0.232311	-0.955124	1.817986
C	-0.163312	-2.073895	2.545170
C	1.036265	-3.490606	1.016186
H	2.039344	-2.491443	-0.600724
H	-0.104446	0.032343	2.122117
H	-0.791223	-1.950500	3.422947
H	1.348561	-4.479185	0.692282
H	-0.073222	-4.219031	2.713220
O	3.581457	1.323495	-0.614251
H	3.799262	0.664847	-1.285009
C	1.394162	3.616093	-0.068316
H	2.143139	3.400584	-0.835816
H	1.762544	4.431771	0.564452
H	0.464265	3.940663	-0.543614

**Avg. Energy:**	-904.063448 hartrees (from 7 conformers)
**Post Energy:**	-904.063861 hartrees<br>ωB97X-V/6-311+G(2df,2p)

RRR

Cartesian coordinates

N	0.556714	1.318273	-0.145378
C	1.477160	0.208558	-0.332050
C	2.828244	0.854356	0.091491
C	2.408154	1.835484	1.191895
C	1.084135	2.416682	0.679638
H	1.512523	-0.086668	-1.384510

H	3.219802	1.410745	-0.765670
H	3.165172	2.601607	1.376349
H	2.251404	1.279568	2.125054
H	0.381029	2.604920	1.498051
C	-0.769949	1.264070	-0.454289
O	-1.575523	2.117516	-0.125816
O	-1.032299	0.148441	-1.156719
C	-2.393487	-0.333088	-1.325188
C	-3.058275	-0.512256	0.039865
H	-3.208605	0.449498	0.533609
H	-2.437399	-1.150283	0.678141
H	-4.031526	-0.996715	-0.090201
C	-2.181635	-1.688815	-1.994104
H	-1.569903	-2.333975	-1.355470
H	-1.671923	-1.567343	-2.955160
H	-3.145597	-2.176478	-2.170020
C	-3.179249	0.612465	-2.230813
H	-3.307111	1.585423	-1.754354
H	-4.165073	0.184808	-2.441964
H	-2.653518	0.748914	-3.181268
C	1.139345	-1.017761	0.499400
C	0.598120	-3.314145	2.015688
C	1.657513	-2.258814	0.122270
C	0.354880	-0.940268	1.651860
C	0.082456	-2.080285	2.403307
C	1.389492	-3.399383	0.873327
H	2.272074	-2.328832	-0.771327
H	-0.068221	0.015004	1.951236
H	-0.538158	-2.003470	3.291221
H	1.794817	-4.357130	0.561272
H	0.382297	-4.204301	2.598986
O	3.838149	-0.052049	0.435632
H	3.490522	-0.632662	1.127199
C	1.247240	3.702295	-0.128719
H	1.608038	4.511395	0.515737
H	0.284463	3.999428	-0.551610
H	1.963705	3.565273	-0.946596

**Avg. Energy:**	-904.064550 hartrees (from 3 conformers)
**Post Energy:**	-904.064917 hartrees<br>ωB97X-V/6-311+G(2df,2p)

References

(1) Hu, D. X.; Grice, P.; Ley, S. V. Rotamers or Diastereomers? An Overlooked NMR Solution. *J. Org. Chem.* **2012**, *77* (11), 5198-5202.
(2) *CrysAlisPro*. v1.171.42.94a, 43.109a & 44.115a. *Rigaku Oxford Diffraction, Rigaku Corporation, Oxford, U.K.* **2025**.
(3) Sheldrick, G. SHELXT - Integrated space-group and crystal-structure determination. *Acta Crystallogr., Sect. A: Found. Adv.* **2015**, *71* (1), 3-8.
(4) Sheldrick, G. Crystal structure refinement with SHELXL. *Acta Crystallogr., Sect. C: Struct. Chem.* **2015**, *71* (1), 3-8.
(5) Dolomanov, O. V.; Bourhis, L. J.; Gildea, R. J.; Howard, J. A. K.; Puschmann, H. OLEX2: a complete structure solution, refinement and analysis program. *J. Appl. Crystallogr.* **2009**, *42* (2), 339-341.
(6) Brals, J.; McGuire, T. M.; Watson, A. J. B. A Chemoselective Polarity-Mismatched Photocatalytic C(sp3)−C(sp2) Cross-Coupling Enabled by Synergistic Boron Activation. *Angew. Chem. Int. Ed.* **2023**, *62* (42), e202310462.
(7) Karmakar, A.; Basha, M.; Venkatesh Babu, G. T.; Botlagunta, M.; Malik, N. A.; Rampulla, R.; Mathur, A.; Gupta, A. K. Tertiary-butoxycarbonyl (Boc) – A strategic group for N-protection/deprotection in the synthesis of various natural/unnatural N-unprotected aminoacid cyanomethyl esters. *Tetrahedron Lett.* **2018**, *59* (48), 4267-4271.
(8) Sun, C. E., William R.; Bolton, Scott A.; Gu, Zhengxiang; Huang, Yanting; Murgesan, Natesan; Zhu, Yeheng. U.S. Patent WO2012125622A1, 2012.
(9) Wang, S.; Sun, J.; Zhang, Q.; Cao, X.; Zhao, Y.; Tang, G.; Yu, B. Amipurimycin: Total Synthesis of the Proposed Structures and Diastereoisomers. *Angew. Chem. Int. Ed.* **2018**, *57* (11), 2884-2888.
(10) Naturale, G.; Lamblin, M.; Commandeur, C.; Felpin, F.-X.; Dessolin, J. Direct C–H Alkylation of Naphthoquinones with Amino Acids Through a Revisited Kochi–Anderson Radical Decarboxylation: Trends in Reactivity and Applications. *Eur. J. Org. Chem.* **2012**, *2012* (29), 5774-5788.
(11) Ma, J.; Lin, J.; Zhao, L.; Harms, K.; Marsch, M.; Xie, X.; Meggers, E. Synthesis of β-Substituted γ-Aminobutyric Acid Derivatives through Enantioselective Photoredox Catalysis. *Angew. Chem. Int. Ed.* **2018**, *57* (35), 11193-11197.
(12) Reich, D.; Noble, A.; Aggarwal, V. K. Facile Conversion of α-Amino Acids into α-Amino Phosphonates by Decarboxylative Phosphorylation using Visible-Light Photocatalysis. *Angew. Chem. Int. Ed.* **2022**, *61* (37), e202207063.
(13) Shen, M.-L.; Shen, Y.; Wang, P.-S. Merging Visible-Light Photoredox and Chiral Phosphate Catalysis for Asymmetric Friedel–Crafts Reaction with in Situ Generation of N-Acyl Imines. *Org. Lett.* **2019**, *21* (9), 2993-2997.
(14) Luo, M.; Shen, Q.; Su, H.; Li, J. M.; Chan, C.-M.; Yu, W.-Y. A Metal-Free Cycloaddition of α-Diazoacetates with Amino Acid-Derived NHPI Esters for the Facile Synthesis of 1,2,4-Triazoles. *Org. Lett.* **2024**, *26* (26), 5511-5516.
(15) Tripathi, K. N.; Belal, M.; Singh, R. P. Organo Photoinduced Decarboxylative Alkylation of Coumarins with N-(Acyloxy)phthalimide. *J. Org. Chem.* **2020**, *85* (2), 1193-1201.
(16) Zhang, M.; Liu, L.; Tan, Y.; Jing, Y.; Liu, Y.; Wang, Z.; Wang, Q. Decarboxylative Radical Sulfilimination via Photoredox, Copper, and Brønsted Base Catalysis. *Angew. Chem. Int. Ed.* **2024**, *63* (6), e202318344.
(17) Yang, Z.-P.; Freas, D. J.; Fu, G. C. The Asymmetric Synthesis of Amines via Nickel-Catalyzed Enantioconvergent Substitution Reactions. *J. Am. Chem. Soc.* **2021**, *143* (7), 2930-2937.

(18) Das, K.; Shibuya, R.; Nakahara, Y.; Germain, N.; Ohshima, T.; Mashima, K. Platinum-Catalyzed Direct Amination of Allylic Alcohols with Aqueous Ammonia: Selective Synthesis of Primary Allylamines. *Angew. Chem. Int. Ed.* **2012**, *51* (1), 150-154.
(19) Hari Babu, M.; Ranjith Kumar, G.; Kant, R.; Sridhar Reddy, M. Ni-Catalyzed regio- and stereoselective addition of arylboronic acids to terminal alkynes with a directing group tether. *Chem. Commun.* **2017**, *53* (27), 3894-3897.
(20) Akkarasamiyo, S.; Sawadjoon, S.; Orthaber, A.; Samec, J. S. M. Tsuji–Trost Reaction of Non-Derivatized Allylic Alcohols. *Eur. J. Chem.* **2018**, *24* (14), 3488-3498.
(21) Zhang, L.; Dong, C.; Ding, C.; Chen, J.; Tang, W.; Li, H.; Xu, L.; Xiao, J. Palladium-Catalyzed Regioselective and Stereoselective Oxidative Heck Arylation of Allylamines with Arylboronic Acids. *Adv. Synth. Catal.* **2013**, *355* (8), 1570-1578.
(22) Lu, X.-Y.; Su, M.-X.; Huang, H.-Y.; Qian, Y.-J.; Sun, H.-L.; Li, X.; Hu, X.-R.; Dong, H.-Y. Iron-Catalyzed Decarboxylative and Deconstructive Cross-Coupling of Acrylic Acids with Ketone-Derived Dihydroquinazolinones. *Adv. Synth. Catal.* **2025**, *367* (8), e202401503.
(23) Burlingham, S.-J.; Guijarro, D.; Bosque, I.; Chinchilla, R.; Gonzalez-Gomez, J. C. Visible-light-mediated decarboxylative (E)-alkenylation of aliphatic carboxylic acids with aryl styryl sulfones under metal-free conditions. *Org. Biomol. Chem.* **2022**, *20* (40), 7923-7928.
(24) Lei, Y.; Qiu, R.; Zhang, L.; Xu, C.; Pan, Y.; Qin, X.; Li, H.; Xu, L.; Deng, Y. Palladium-Catalyzed Direct Arylation of Allylamines with Simple Arenes. *ChemCatChem* **2015**, *7* (8), 1275-1279.
(25) Alamillo-Ferrer, C.; Curle, J. M.; Davidson, S. C.; Lucas, S. C. C.; Atkinson, S. J.; Campbell, M.; Kennedy, A. R.; Tomkinson, N. C. O. Alkene Oxyamination Using Malonoyl Peroxides: Preparation of Pyrrolidines and Isoxazolidines. *J. Org. Chem.* **2018**, *83* (12), 6728-6740.
(26) Liu, J.; Cao, C.-G.; Sun, H.-B.; Zhang, X.; Niu, D. Catalytic Asymmetric Umpolung Allylation of Imines. *J. Am. Chem. Soc.* **2016**, *138* (40), 13103-13106.
(27) Leikoski, T.; Wrigstedt, P.; Helminen, J.; Matikainen, J.; Sipilä, J.; Yli-Kauhaluoma, J. The Heck reaction of polymer-supported allylamine with aryl iodides. *Tetrahedron* **2013**, *69* (2), 839-843.
(28) Chen, W.; Huang, X.; Zhou, H. A Novel One-Pot Stereoselective Synthesis of N-[(E)-Homocinnamyl] Amides. *Synthesis* **2004**, *2004* (10), 1573-1576.
(29) Qi, C.; Force, G.; Gandon, V.; Lebœuf, D. Hexafluoroisopropanol-Promoted Haloamidation and Halolactonization of Unactivated Alkenes. *Angew. Chem. Int. Ed.* **2021**, *60* (2), 946-953.
(30) Dener, J. M.; Fantauzzi, P. P.; Kshirsagar, T. A.; Kelly, D. E.; Wolfe, A. B. Large-Scale Syntheses of FMOC-Protected Non-Proteogenic Amino Acids: Useful Building Blocks for Combinatorial Libraries. *Org. Process Res. Dev.* **2001**, *5* (4), 445-449.
(31) Prediger, P.; Barbosa, L. F.; Génisson, Y.; Correia, C. R. D. Substrate-Directable Heck Reactions with Arenediazonium Salts. The Regio- and Stereoselective Arylation of Allylamine Derivatives and Applications in the Synthesis of Naftifine and Abamines. *J. Org. Chem.* **2011**, *76* (19), 7737-7749.
(32) Griffiths, R. C.; Smith, F. R.; Long, J. E.; Scott, D.; Williams, H. E. L.; Oldham, N. J.; Layfield, R.; Mitchell, N. J. Site-Selective Installation of N-Modified Sidechains into Peptide and Protein Scaffolds via Visible-Light-Mediated Desulfurative C–C Bond Formation. *Angew. Chem. Int. Ed.* **2022**, *61* (2), e202110223.
(33) Smith, S. G.; Goodman, J. M. Assigning Stereochemistry to Single Diastereoisomers by GIAO NMR Calculation: The DP4 Probability. *J. Am. Chem. Soc.* **2010**, *132* (37), 12946-12959.

Spectra

3-((*tert*-Butoxycarbonyl)amino)-3-(*p*-tolyl)propanoic acid (2g’)

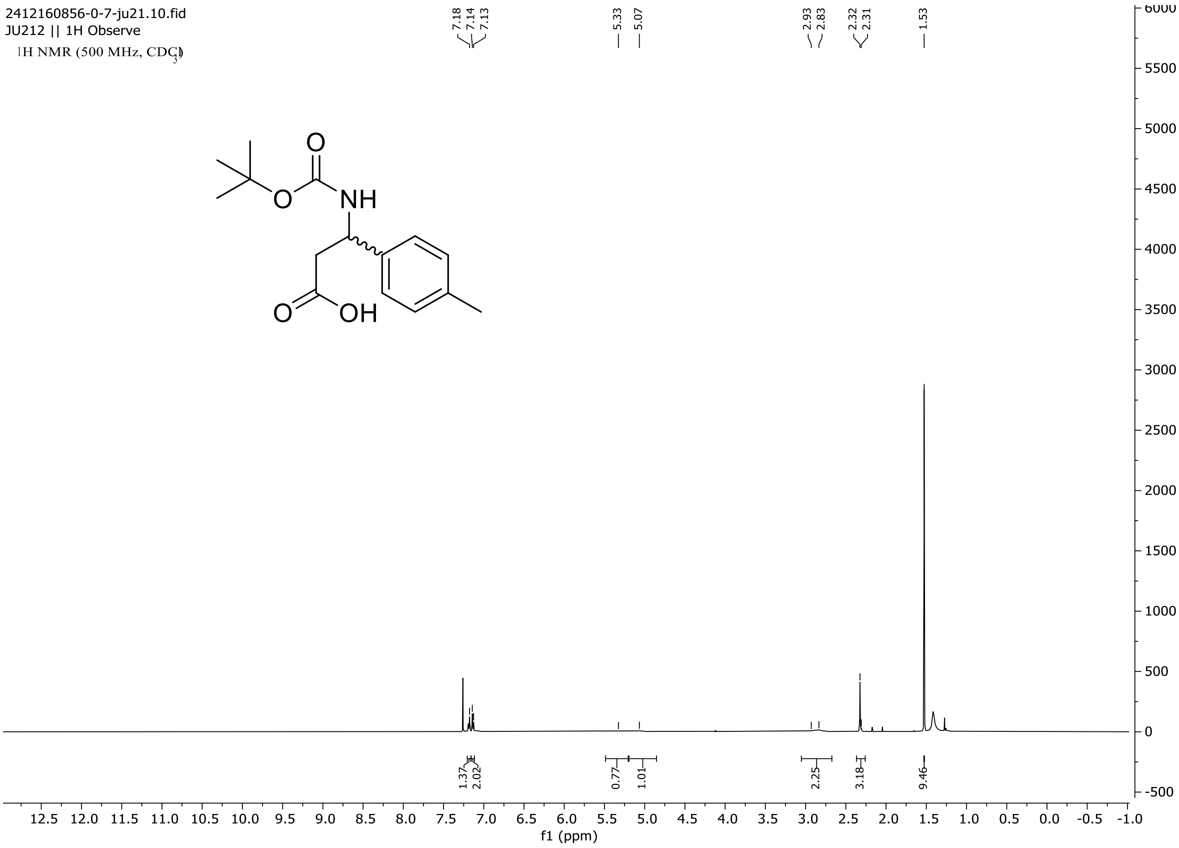

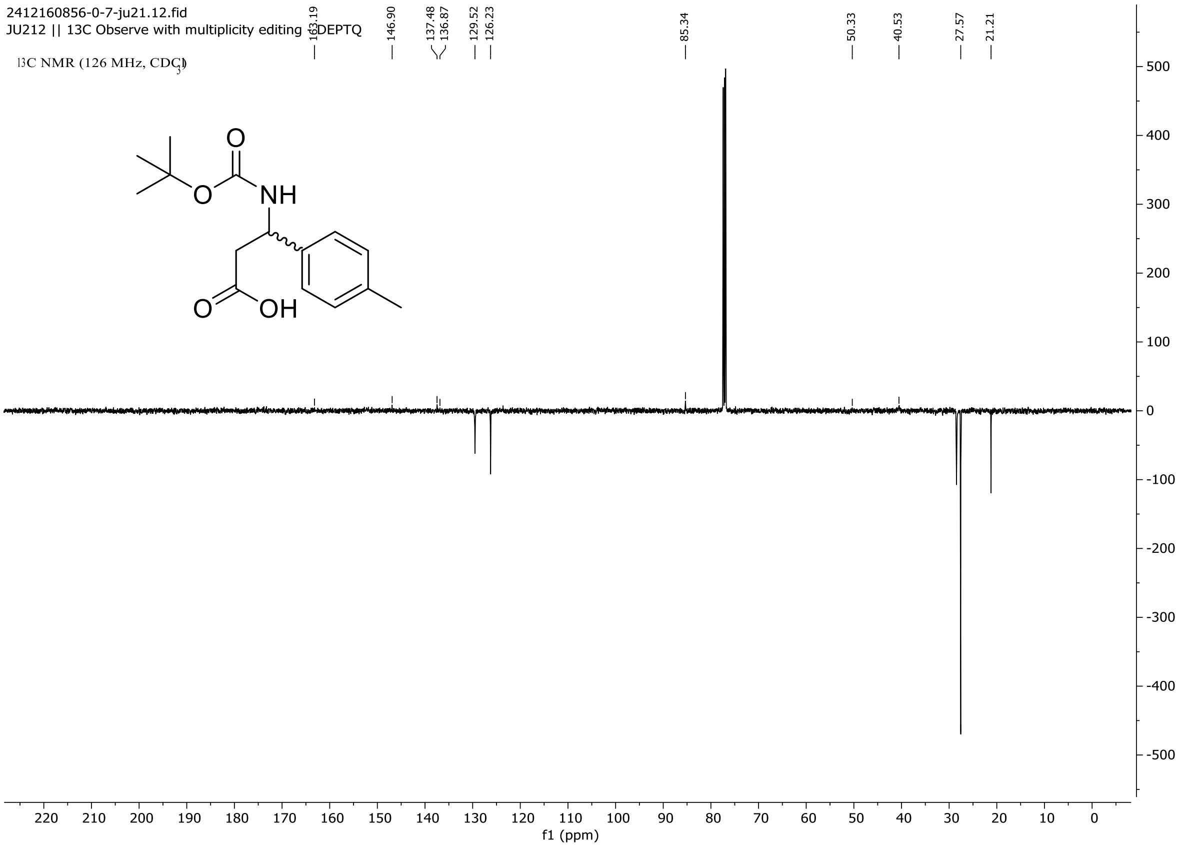

tert-Butyl cinnamoylcarbamate (52a)

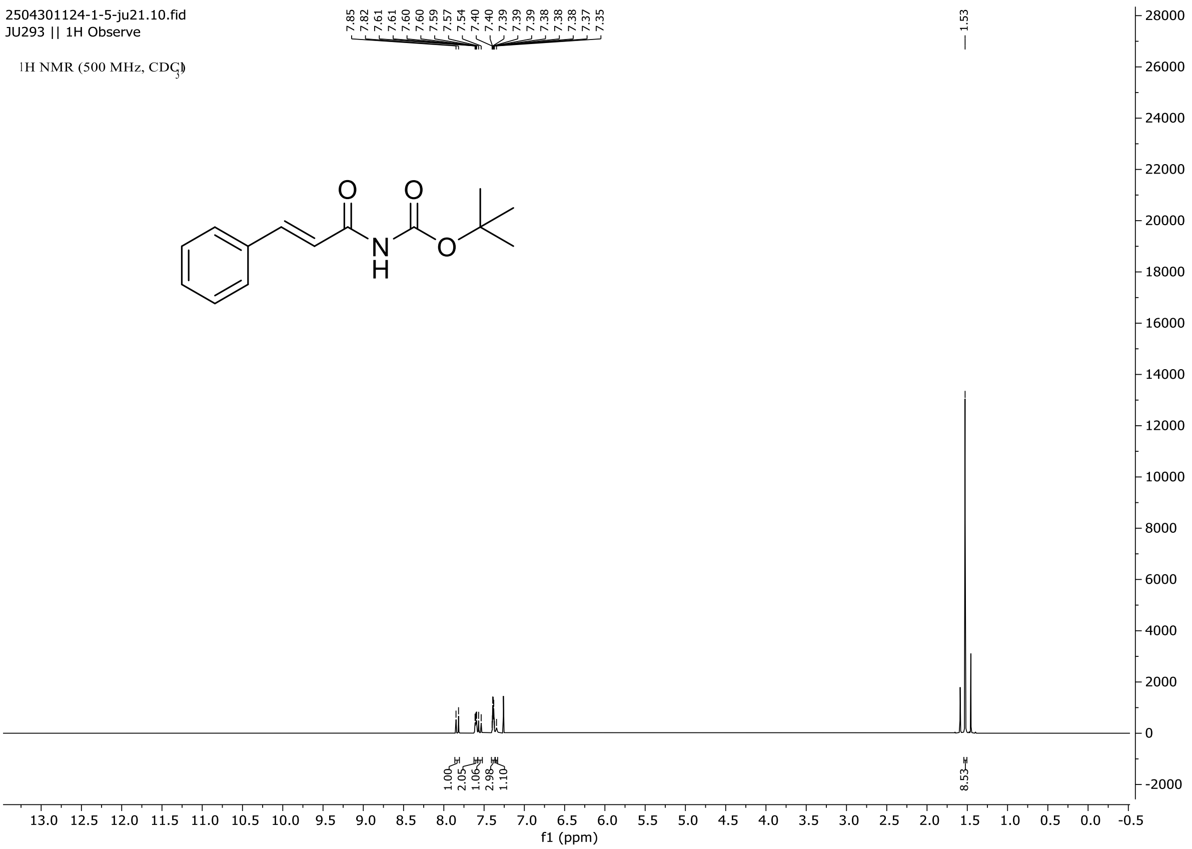

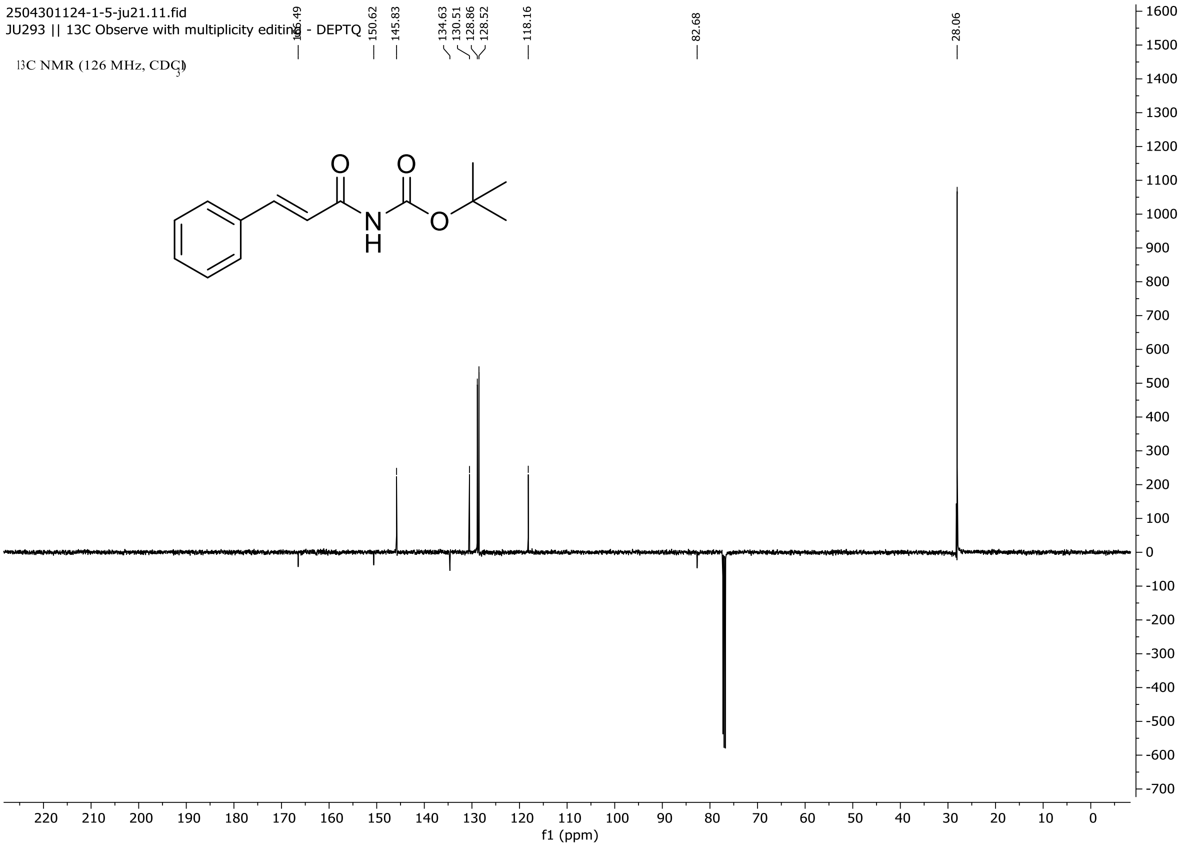

***tert*-Butyl (*S*)-4-(2-((*tert*-butoxycarbonyl)amino)-3-((1,3-dioxoisoindolin-2-yl)oxy)-3-oxopropyl)-1*H*-imidazole-1-carboxylate (2f)**

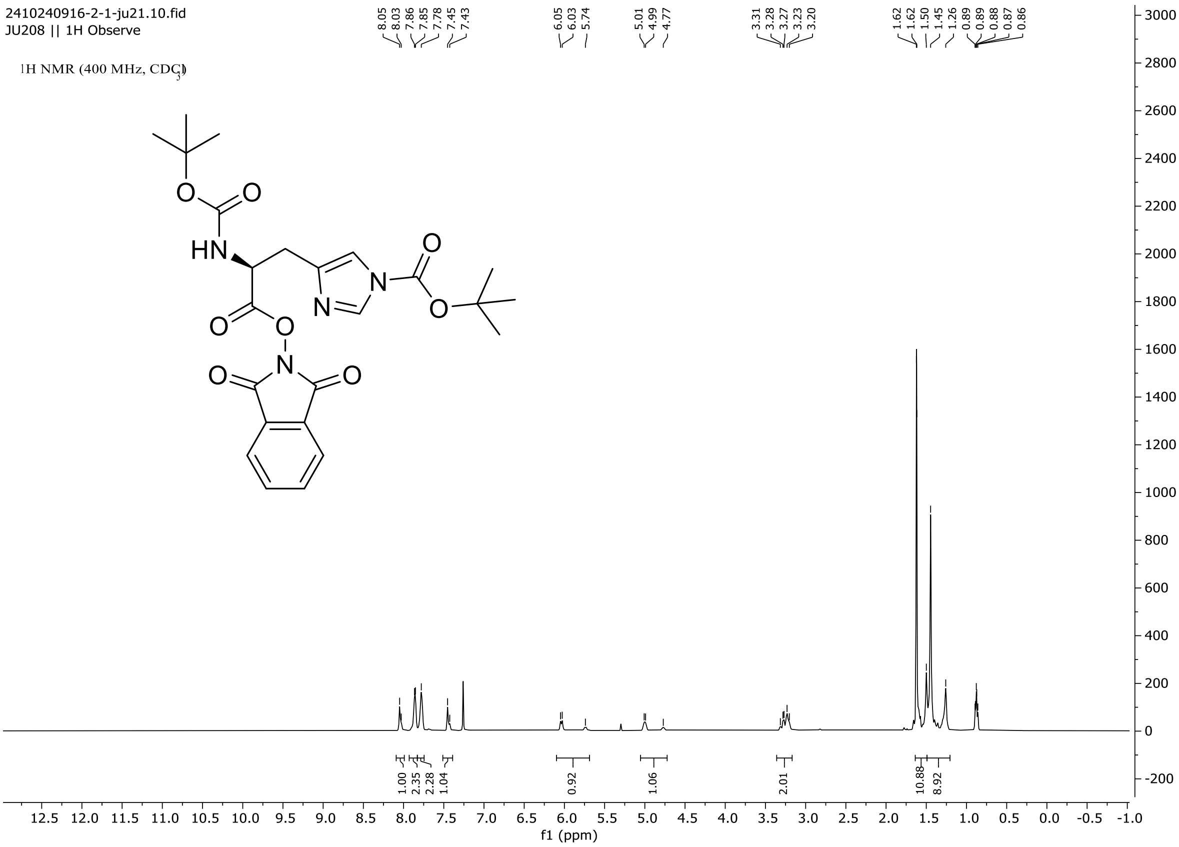

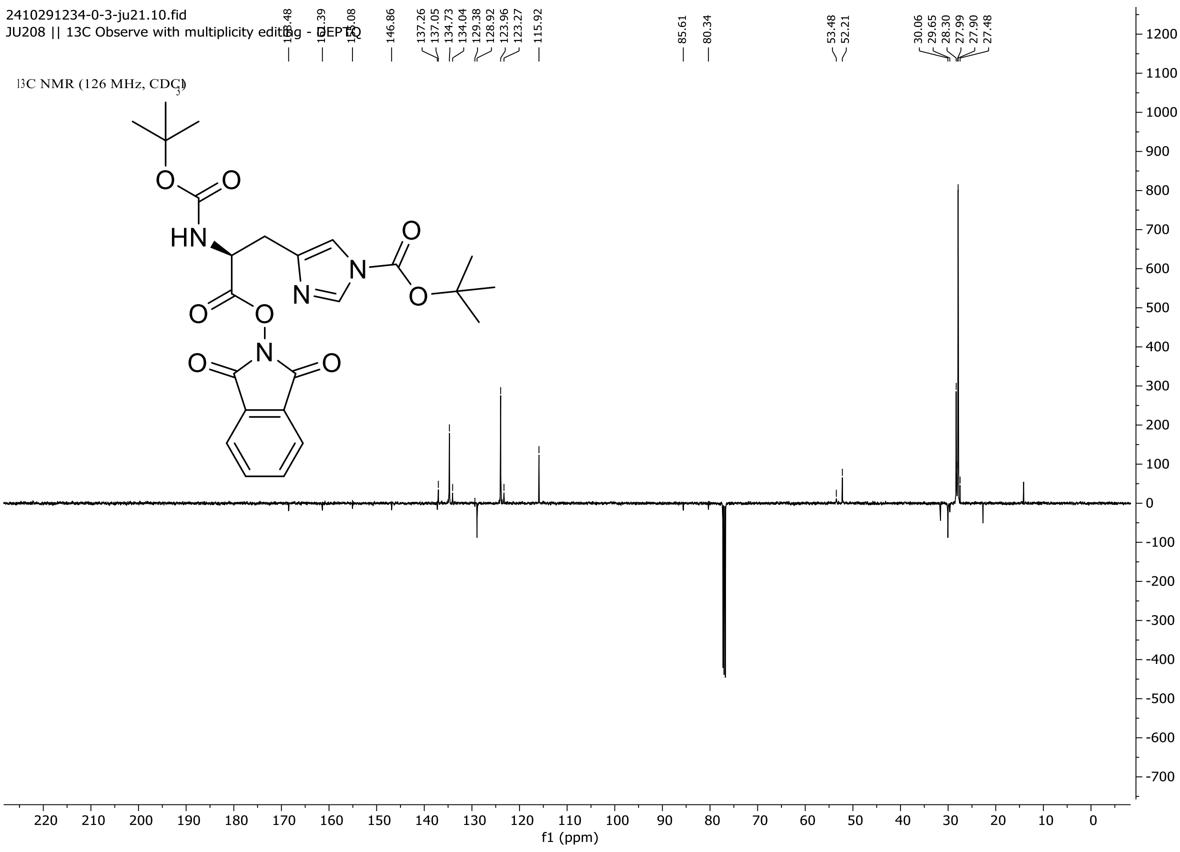

1,3-Dioxoisoindolin-2-yl 3-((*tert*-butoxycarbonyl)amino)-3-(*p*-tolyl)propanoate (2g)

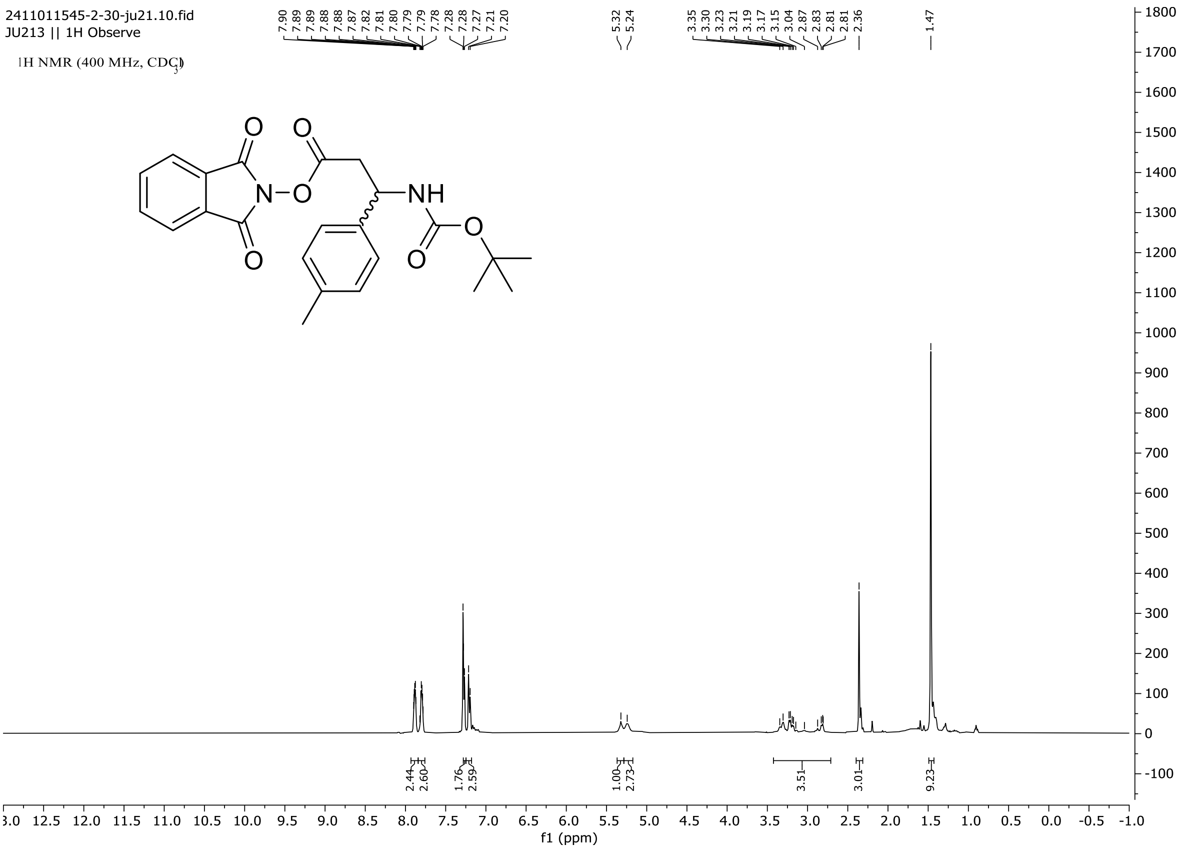

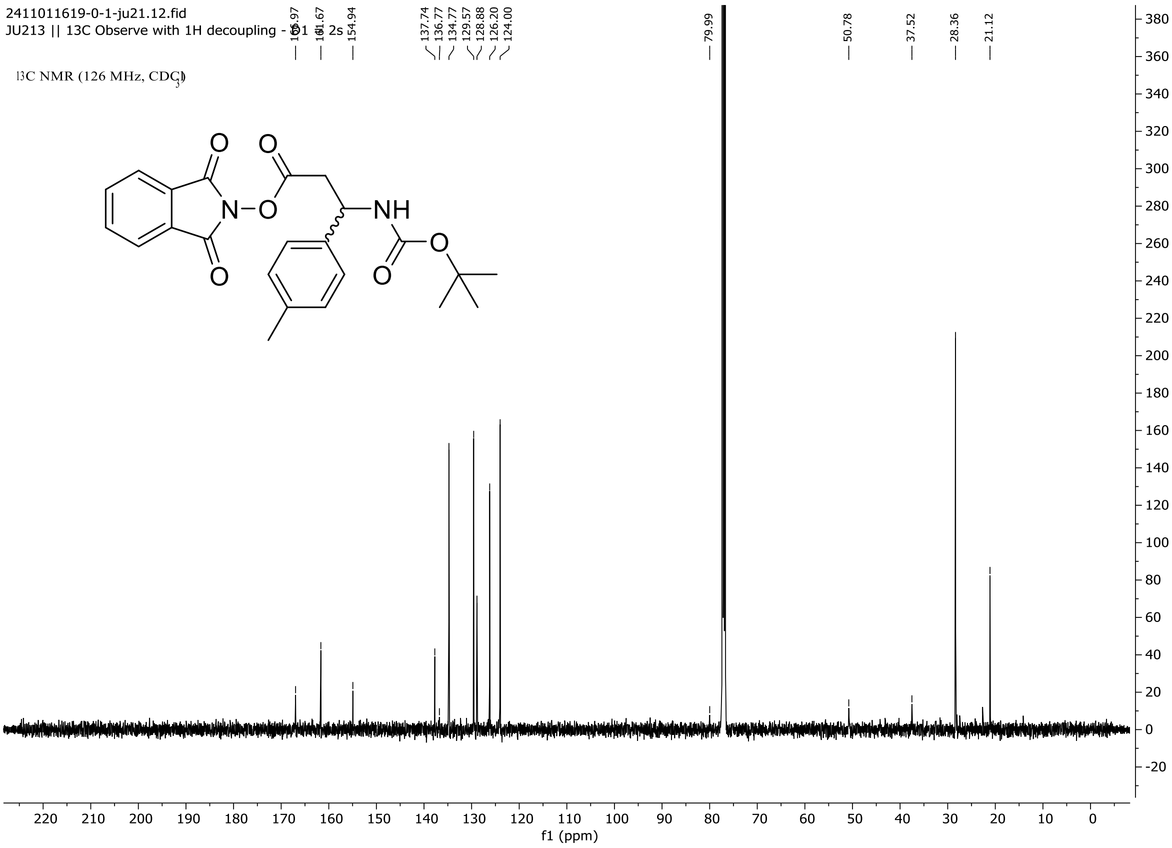

1,3-Dioxoisoindolin-2-yl 2-((*tert*-butoxycarbonyl)amino)cyclopentane-1-carboxylate (2j)

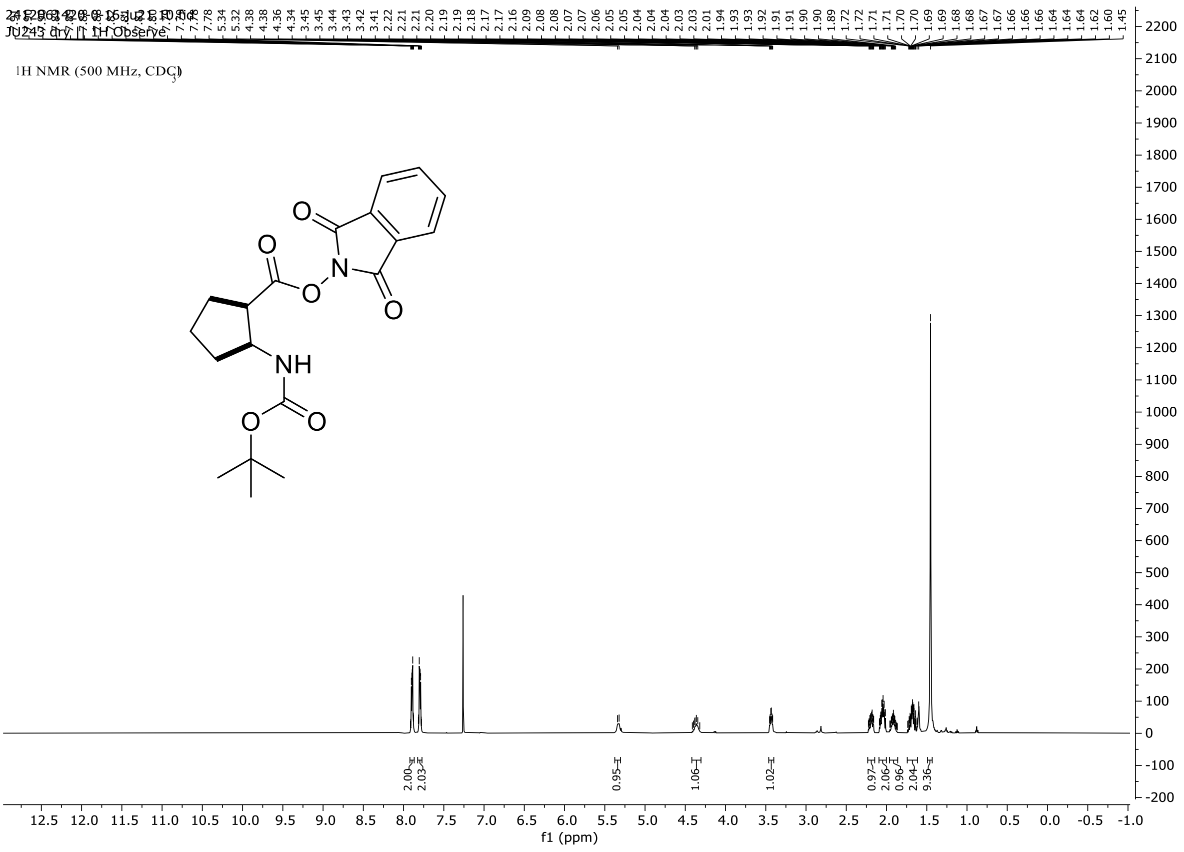

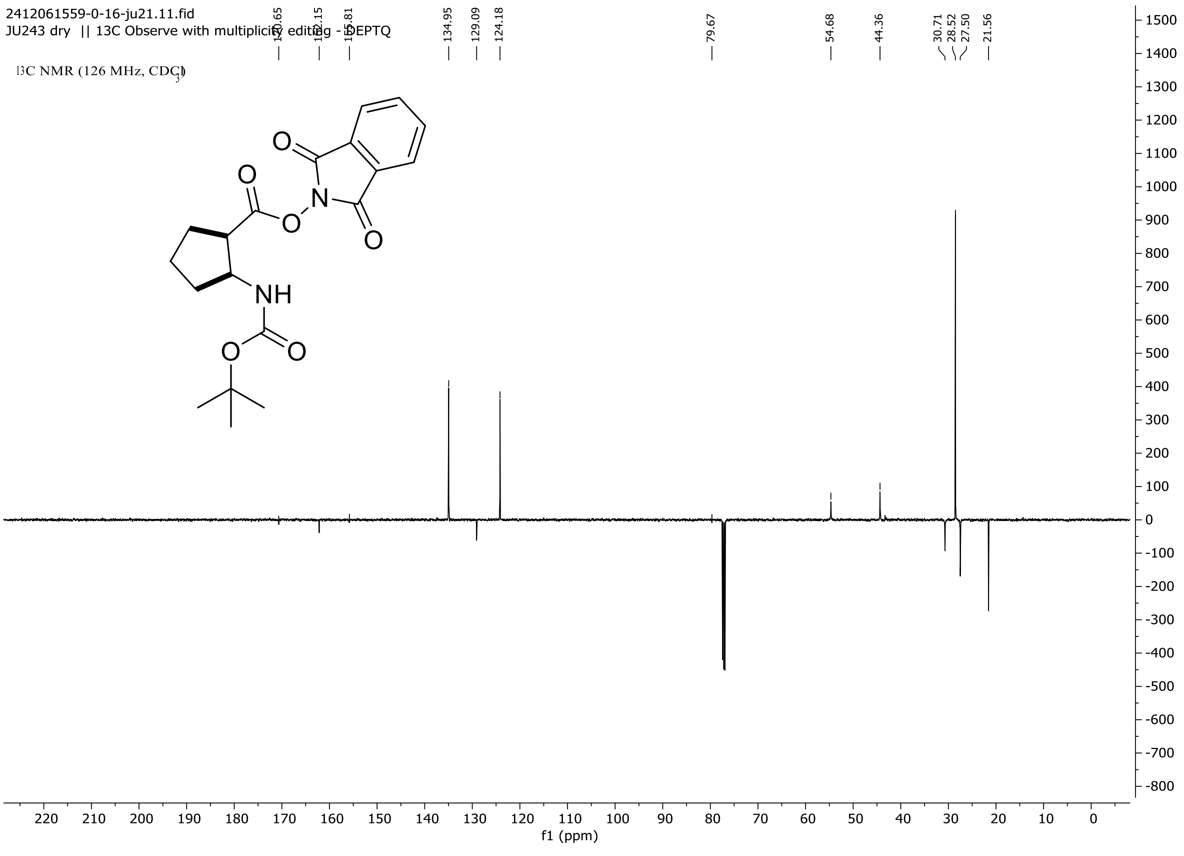

1,3-Dioxoisoindolin-2-yl acetylglycinate (2l)

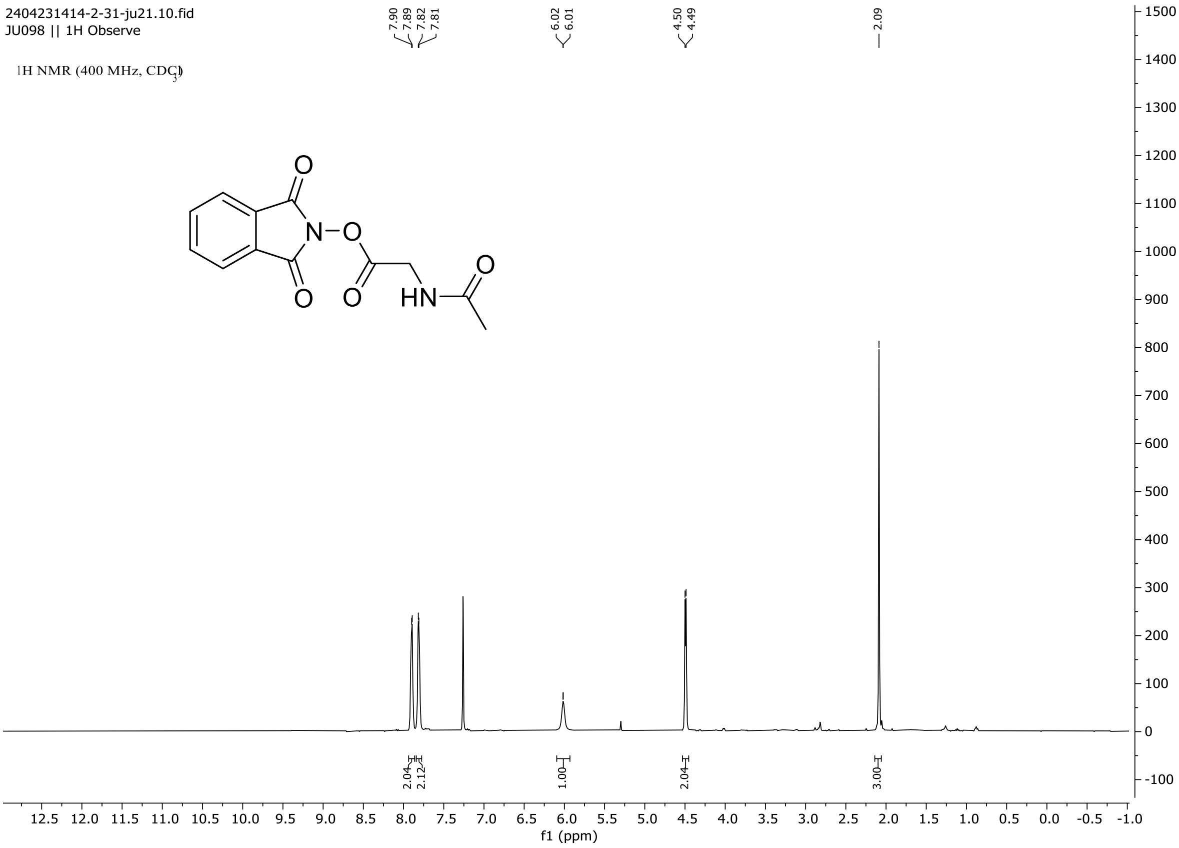

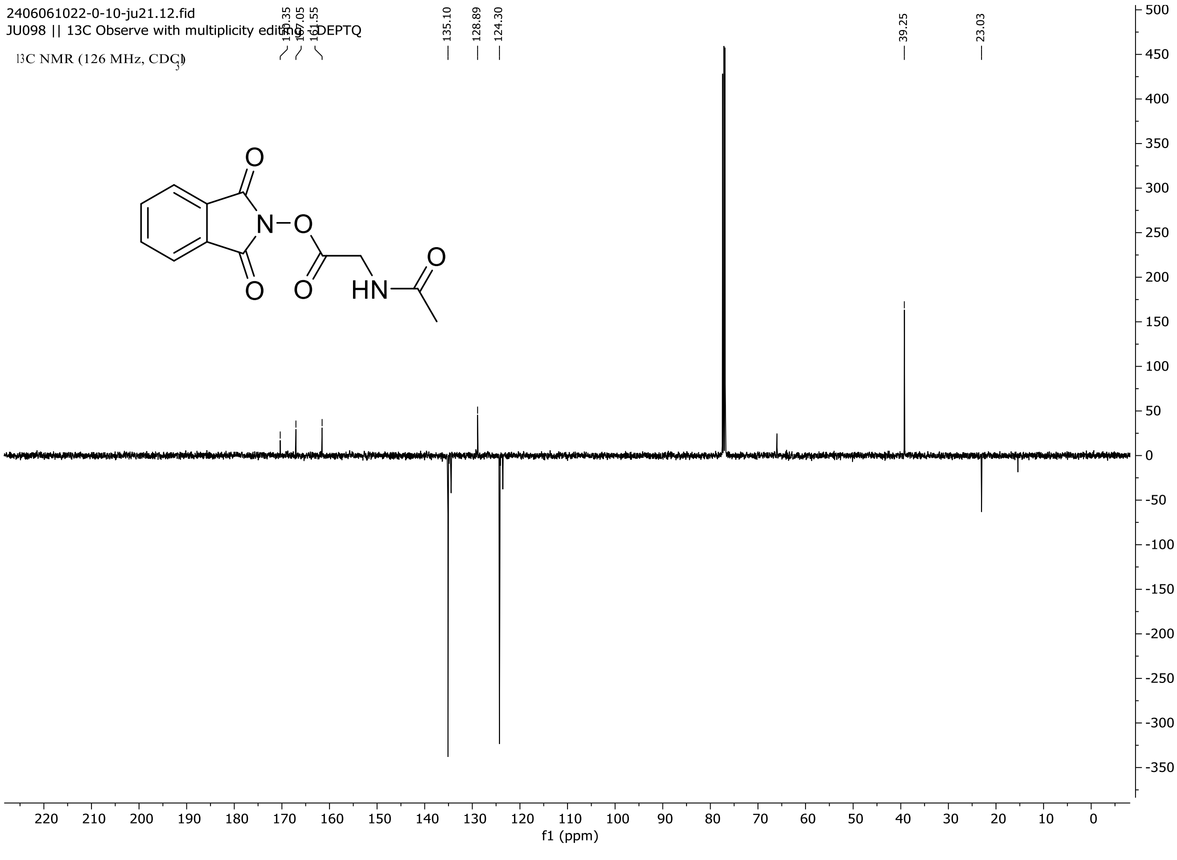

tert-Butyl cinnamylcarbamate (3)

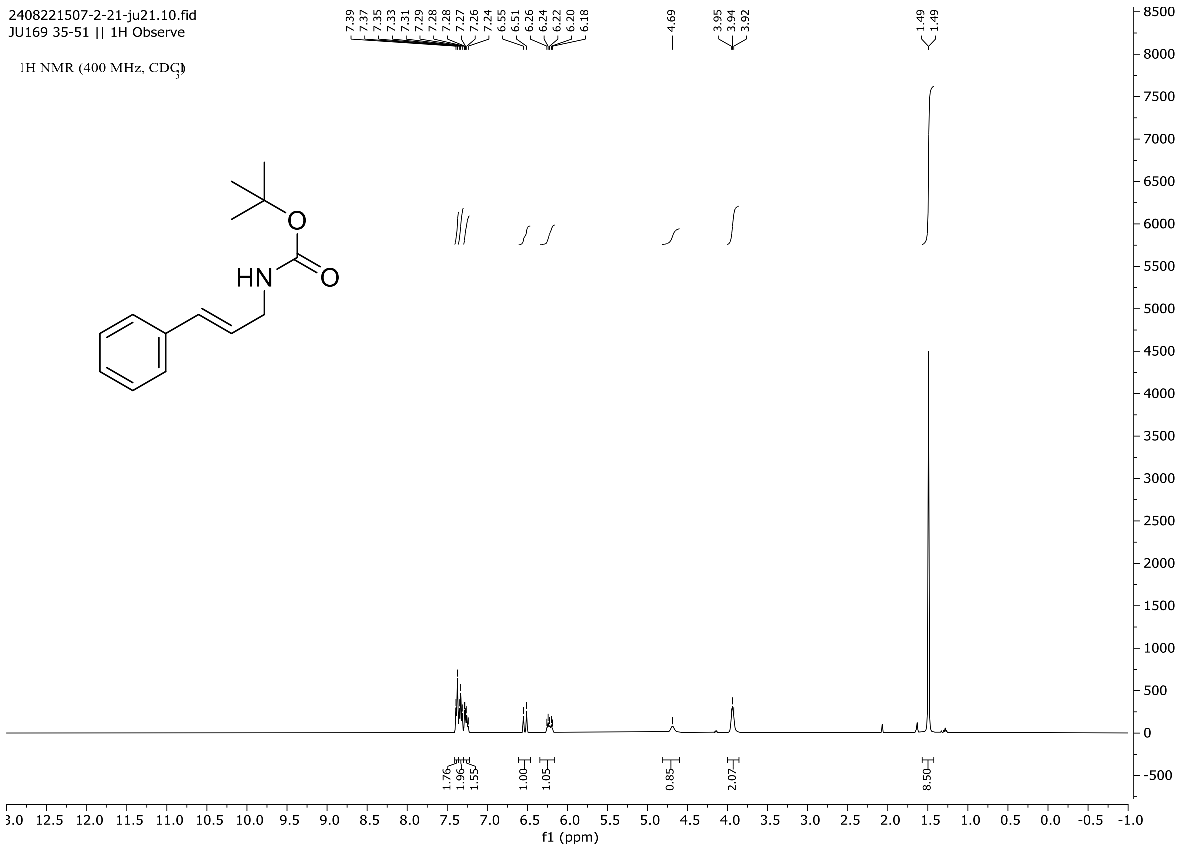

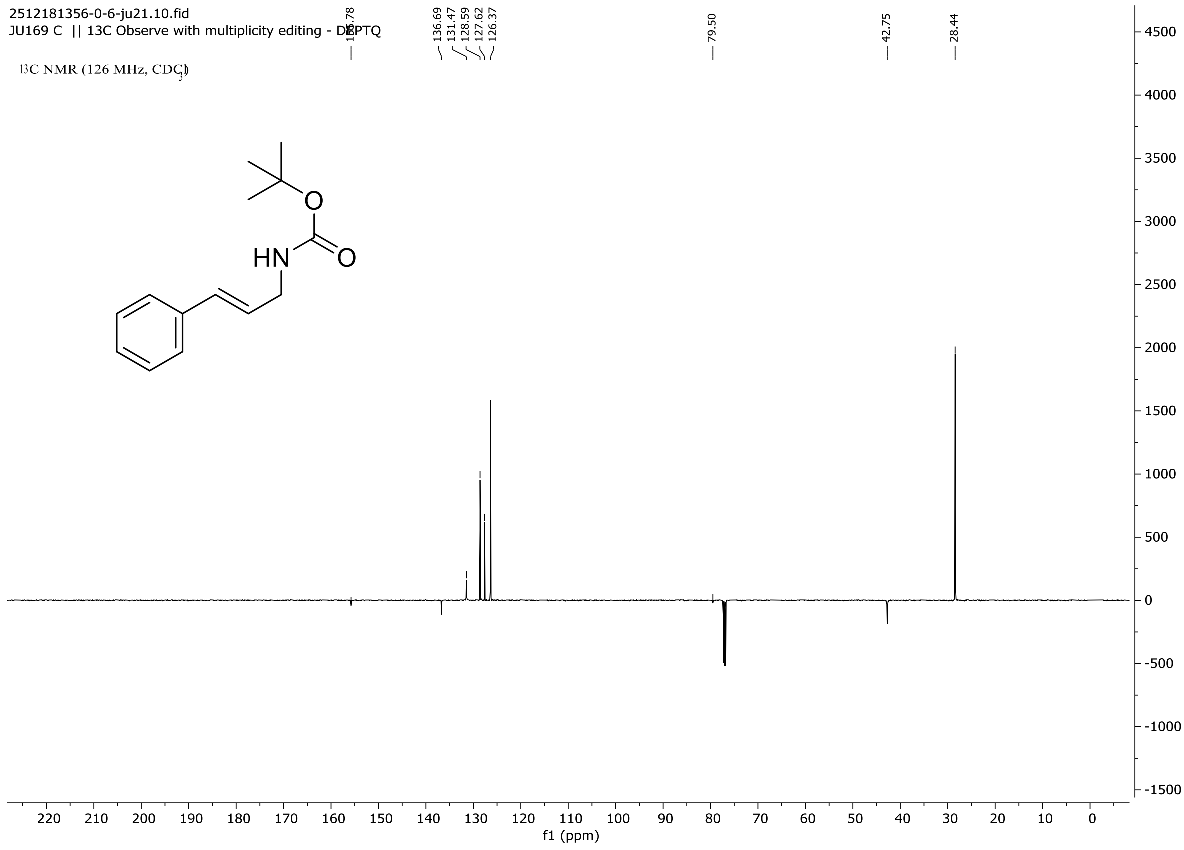

tert-Butyl (*E*)-(4-phenylbut-3-en-2-yl)carbamate (4)

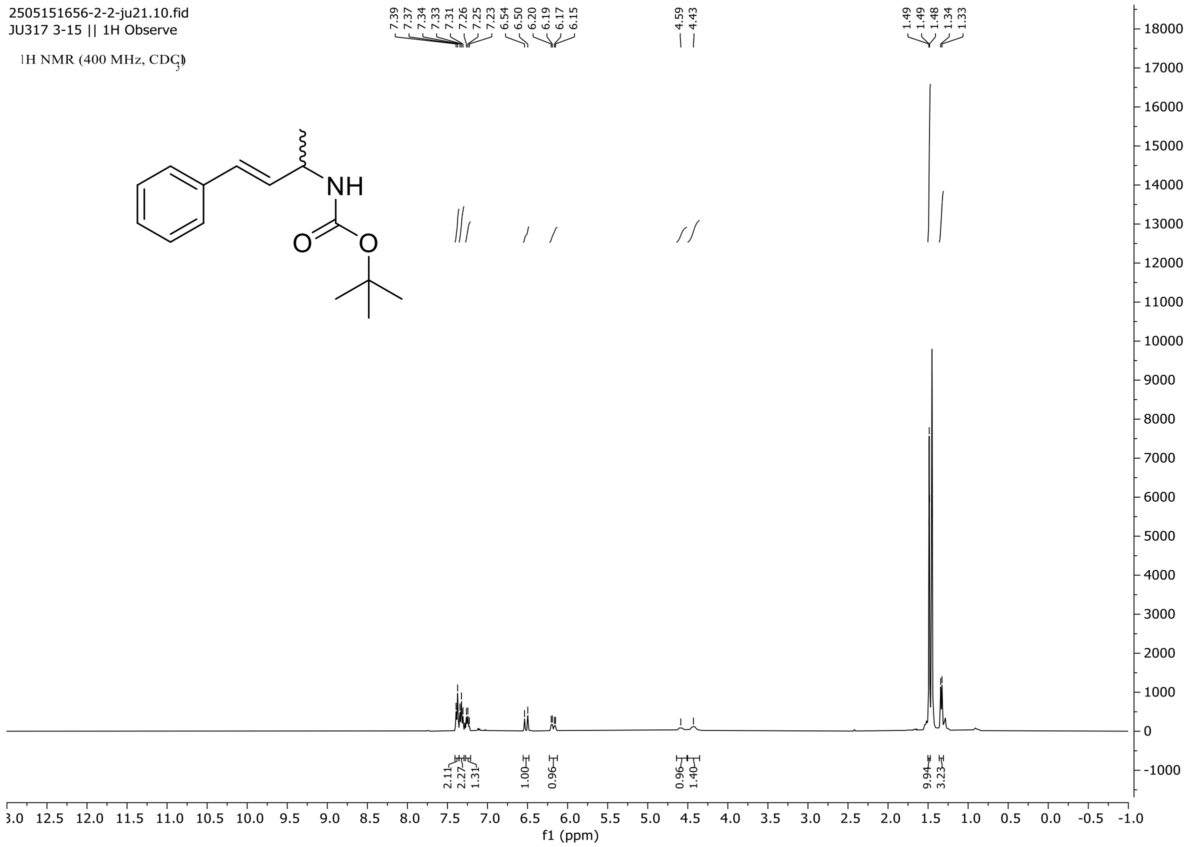

(*E*)-*N*-cinnamylacetamide (5)

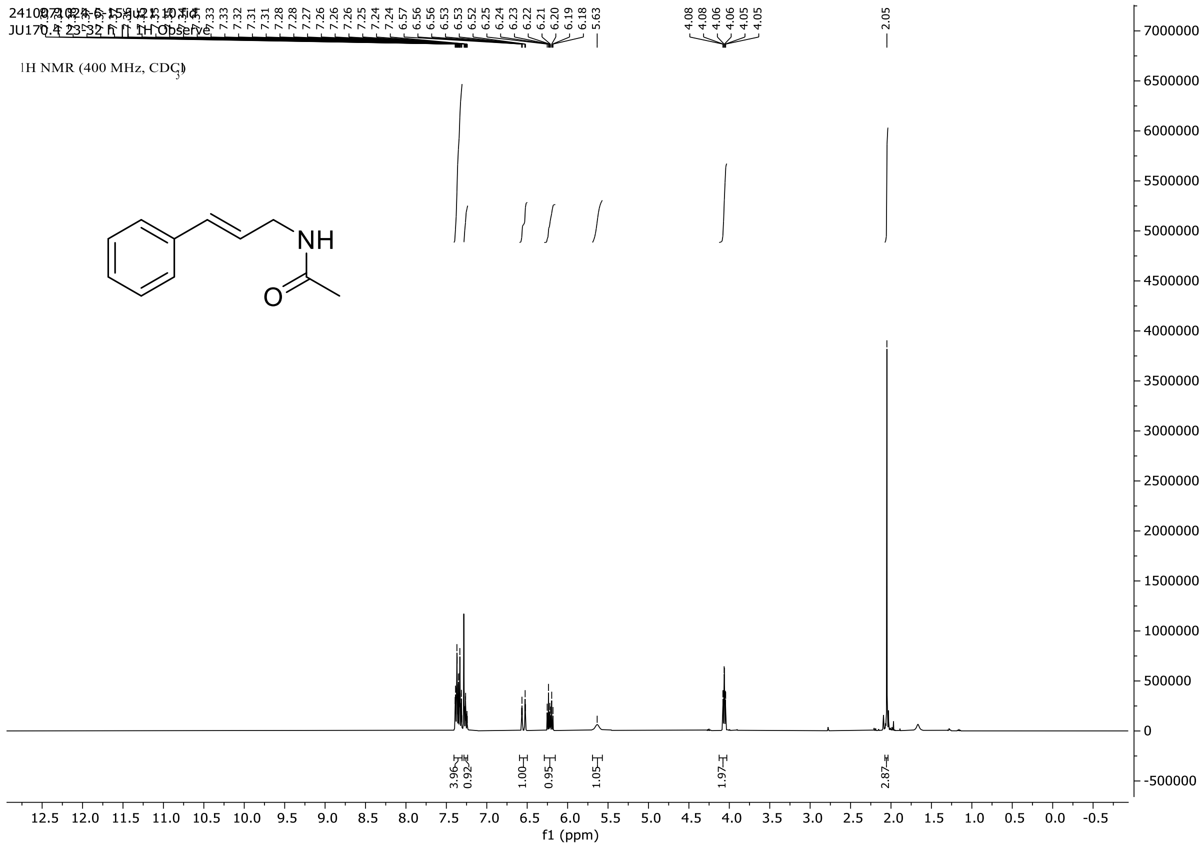

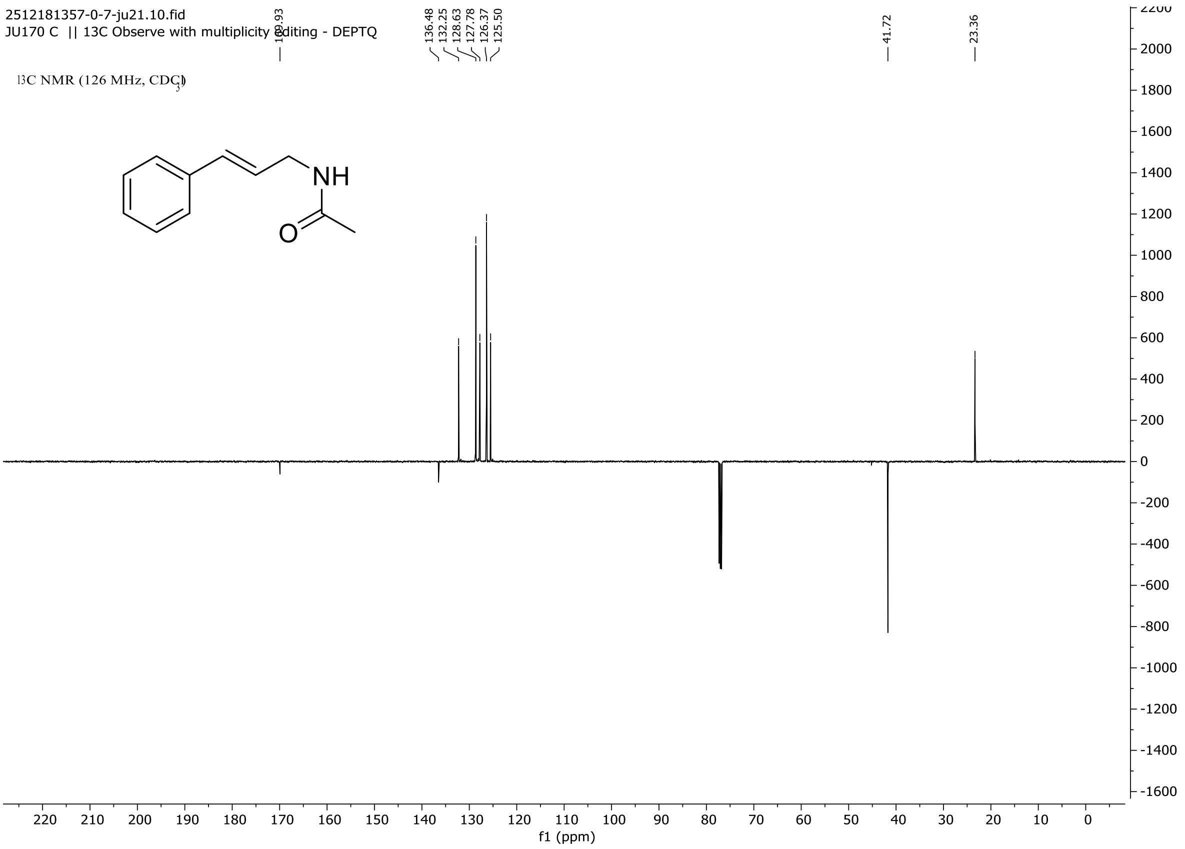

tert-Butyl (*E*)-methyl(4-phenylbut-3-en-2-yl)carbamate (6)

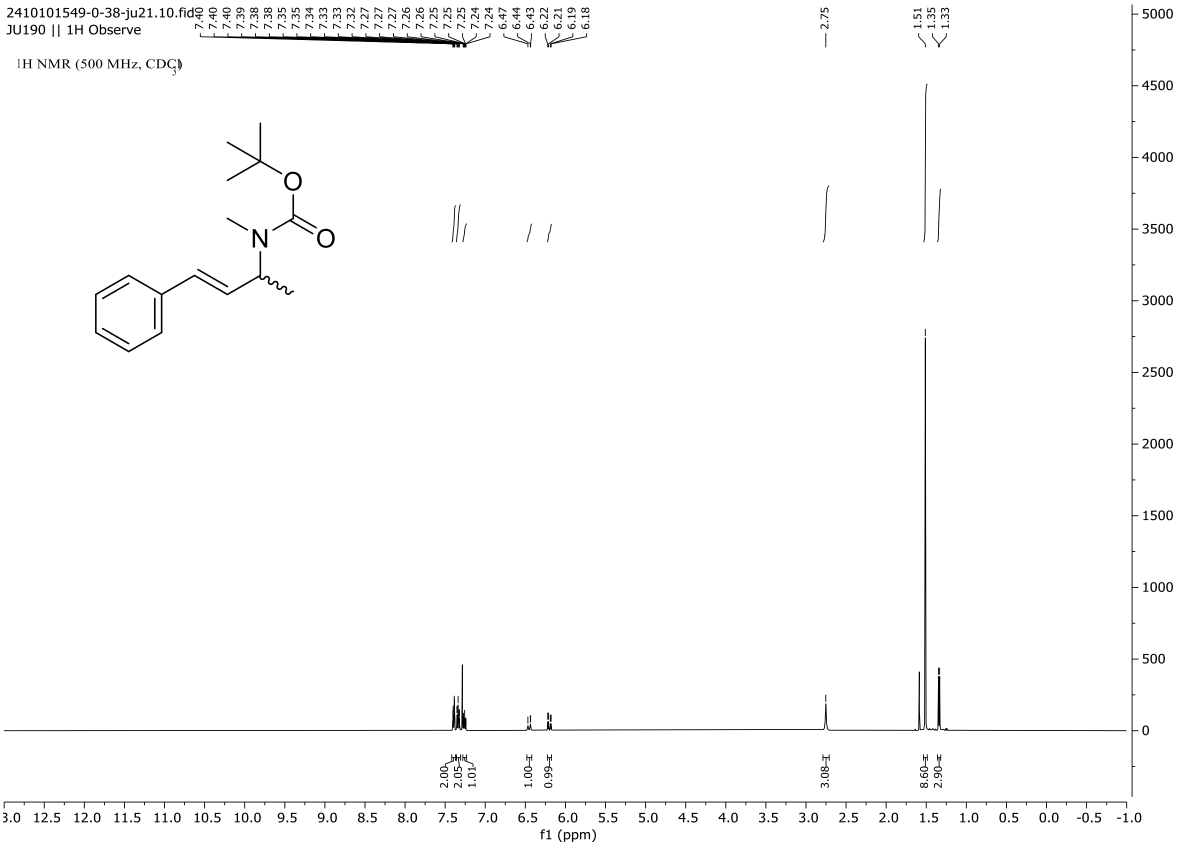

tert-Butyl (*E*)-(3-(4-chlorophenyl)allyl)carbamate (7)

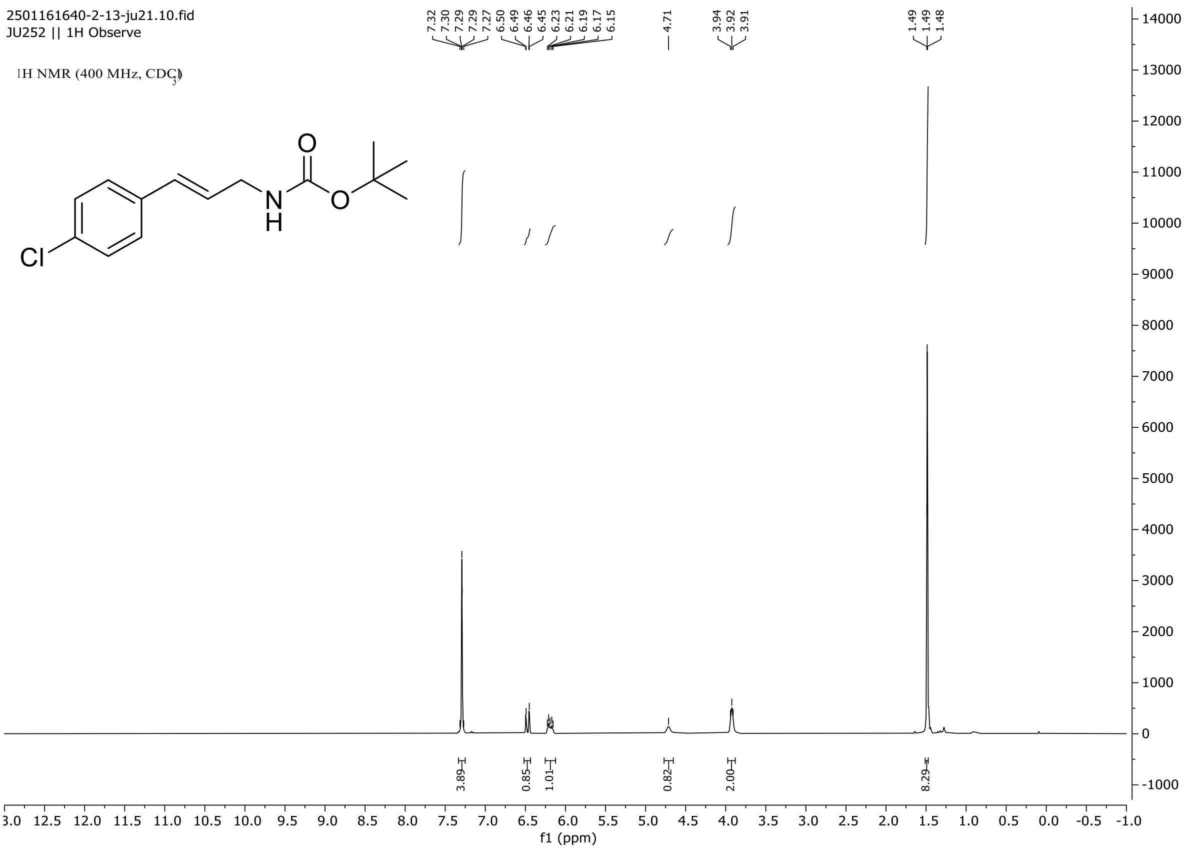

tert-Butyl (*E*)-(3-(4-methoxyphenyl)allyl)carbamate (8)

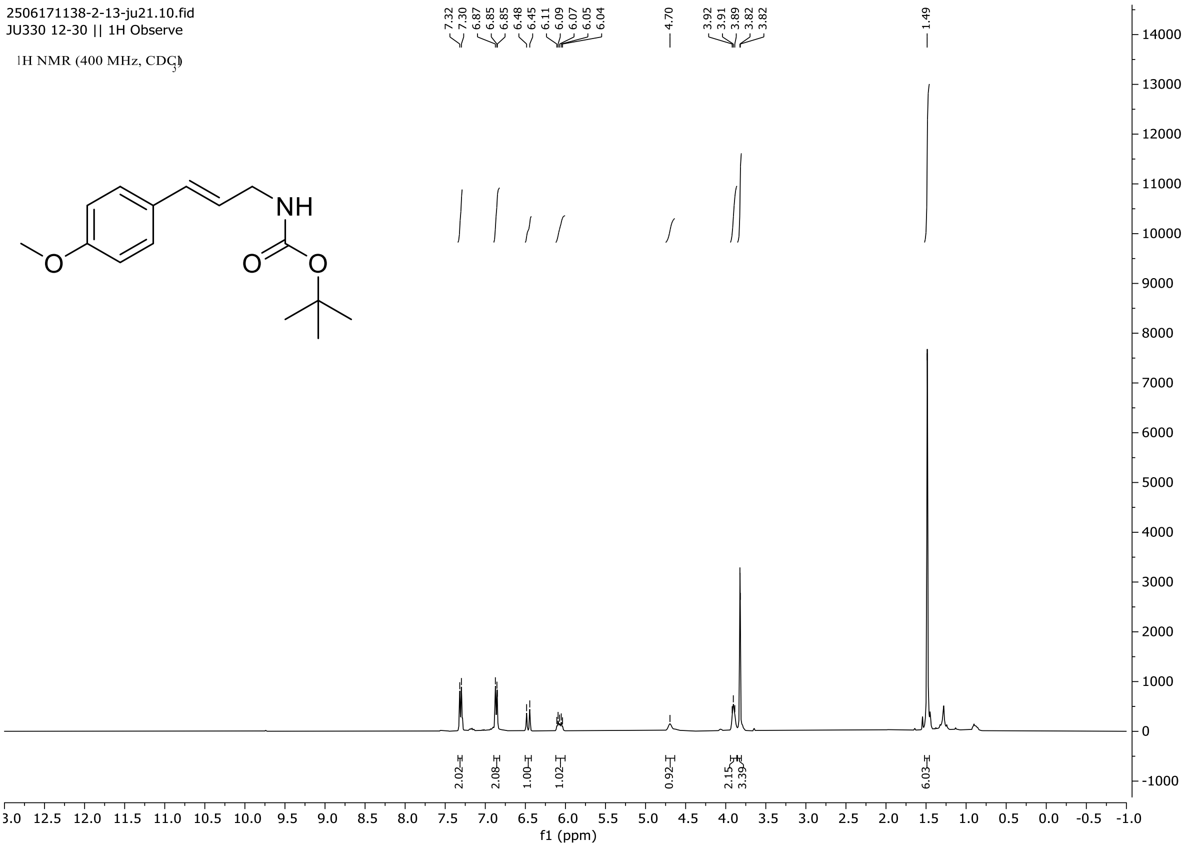

tert-Butyl (*E*)-(1,4-diphenylbut-3-en-2-yl)carbamate (10)

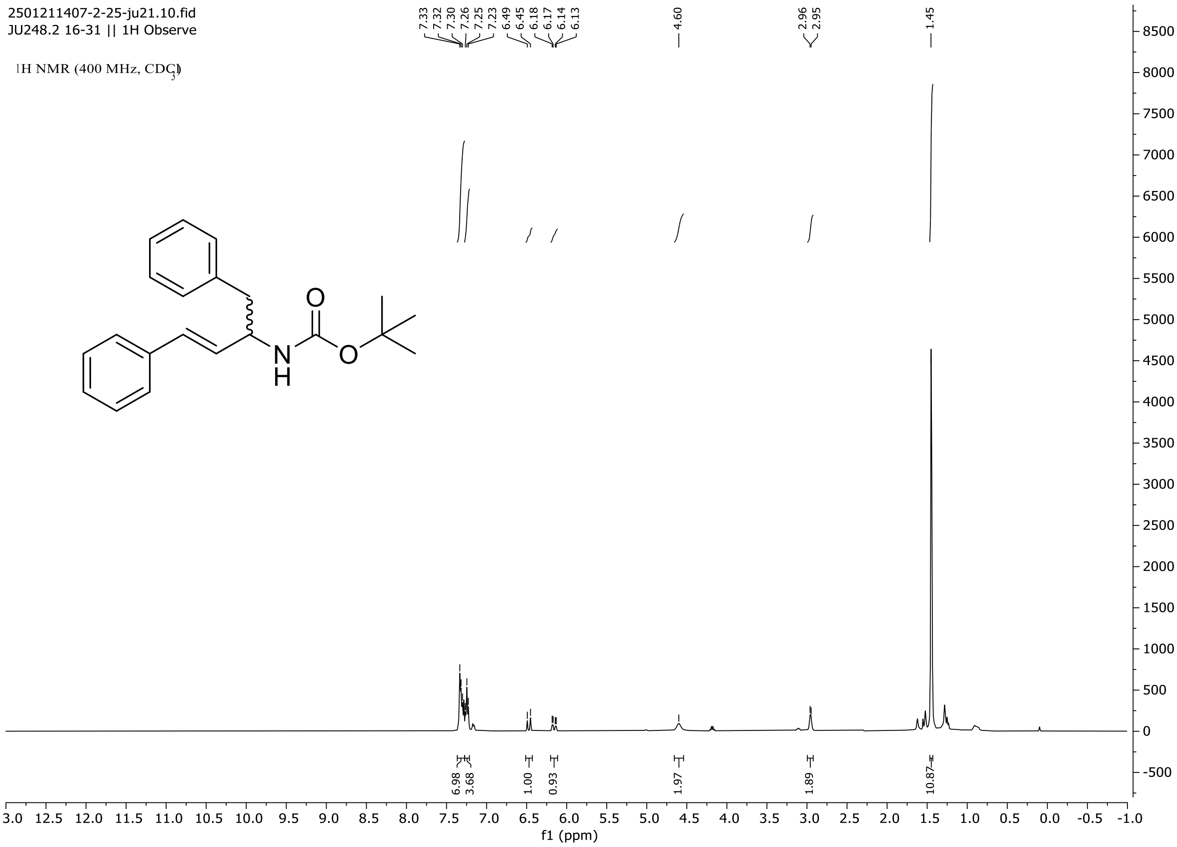

(*E*)-*N*-(1,4-Diphenylbut-3-en-2-yl)acetamide (11)

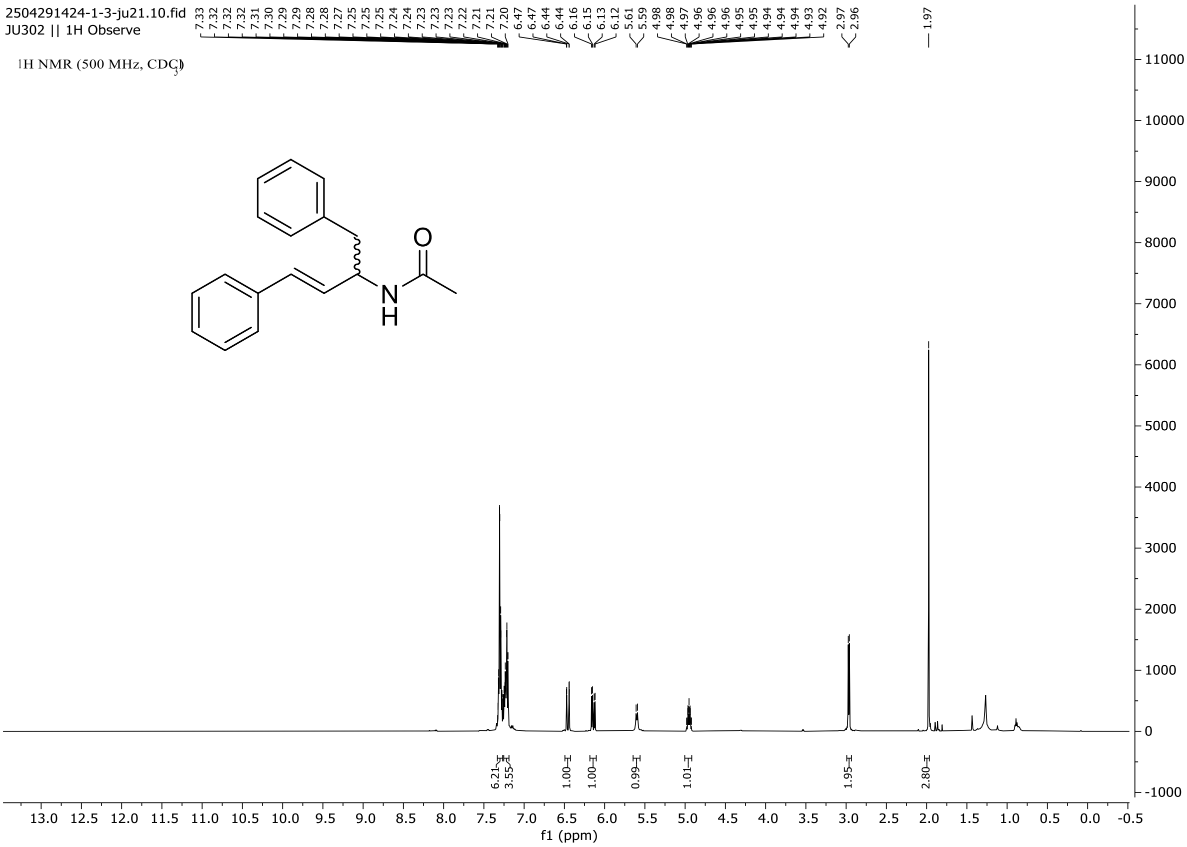

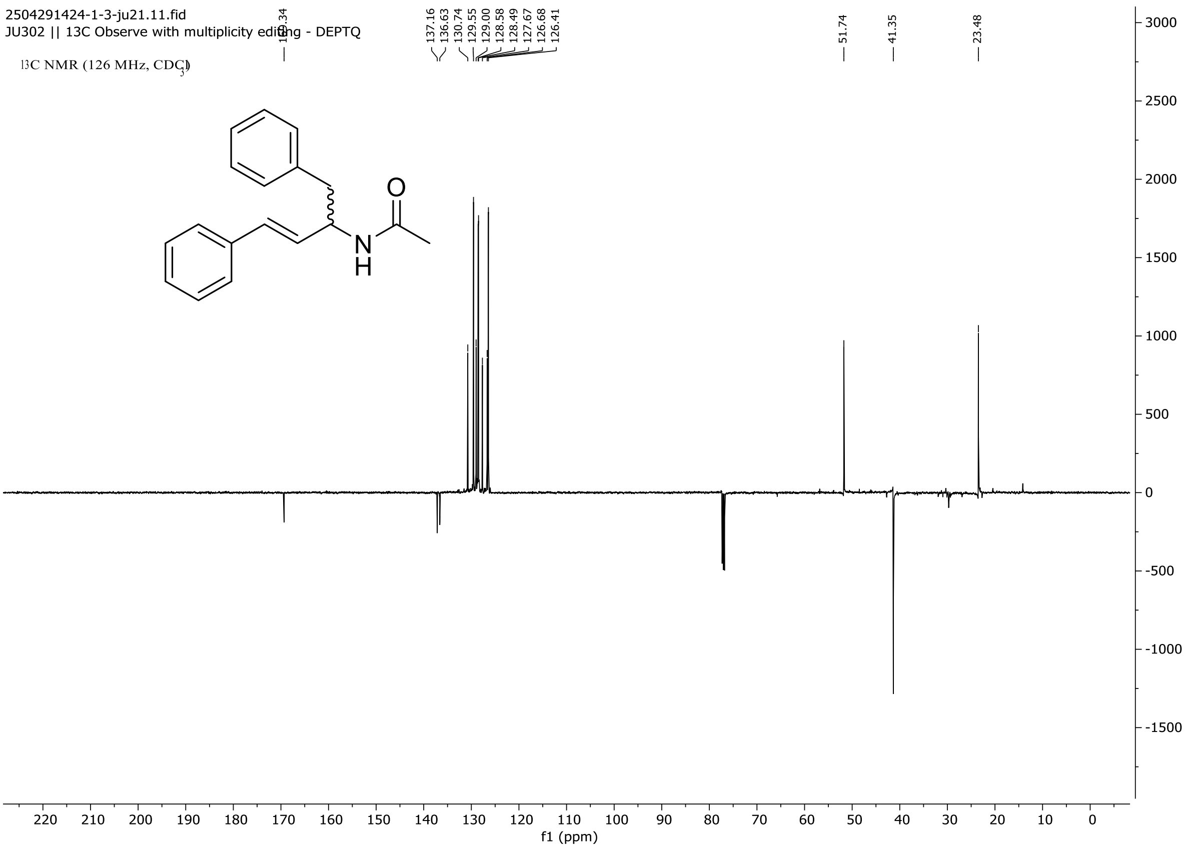

tert-Butyl cinnamyl(methyl)carbamate (12)

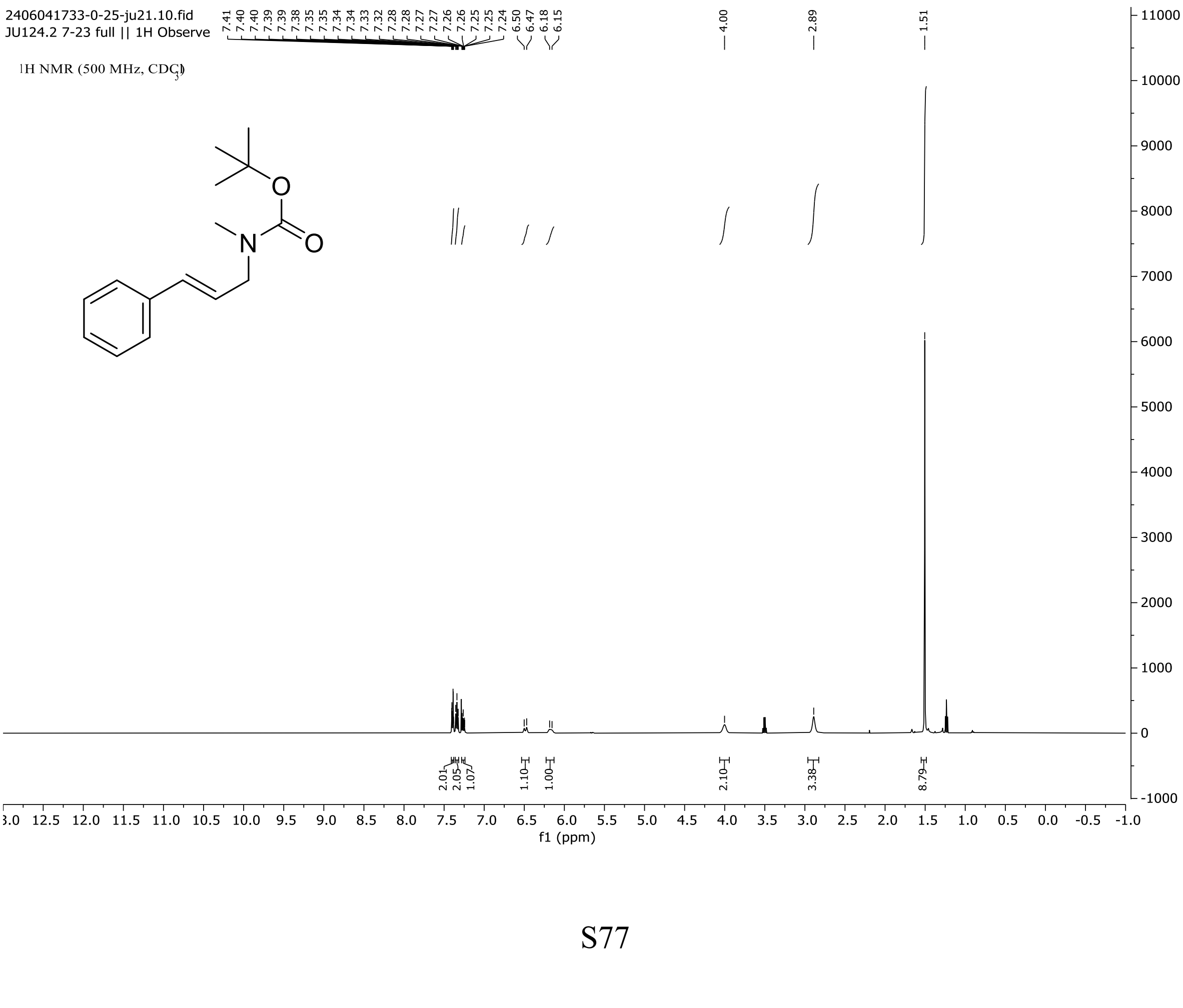

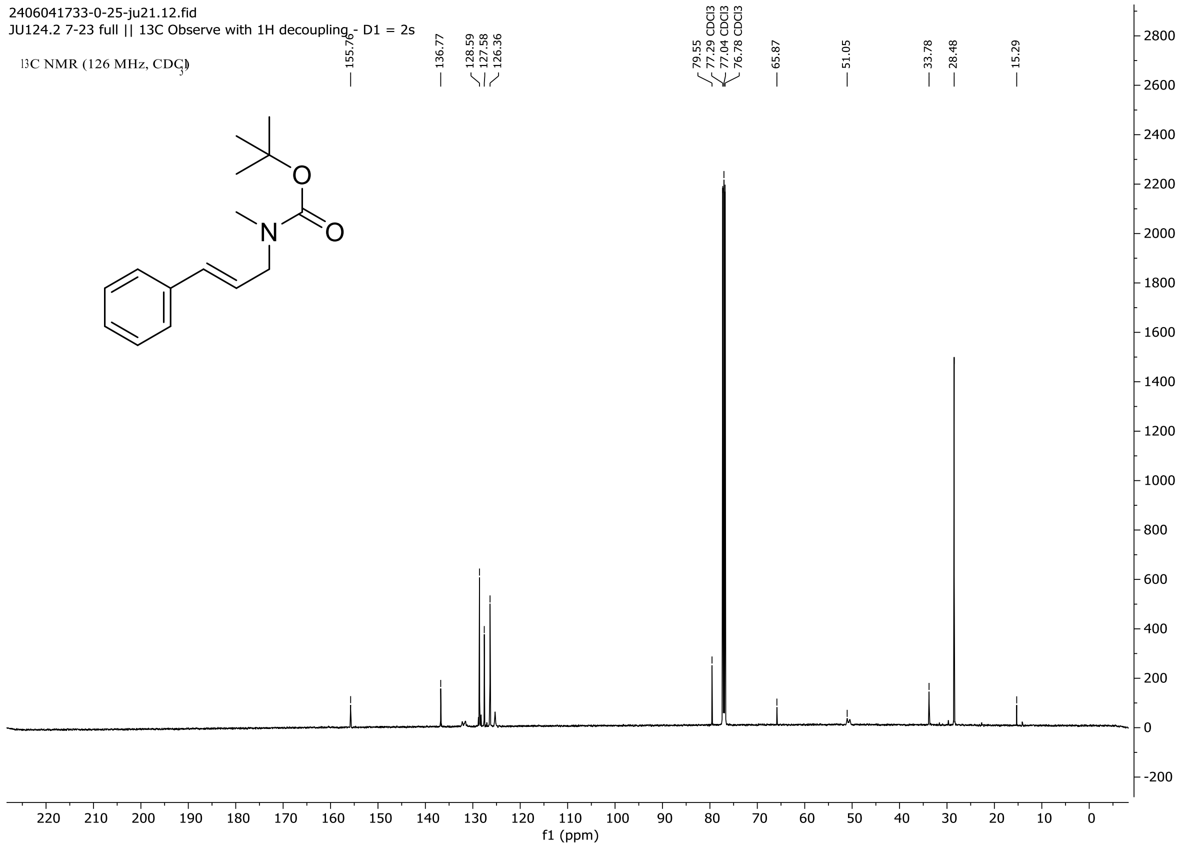

tert-Butyl (*E*)-(3-(4-fluorophenyl)allyl)(methyl)carbamate (13)

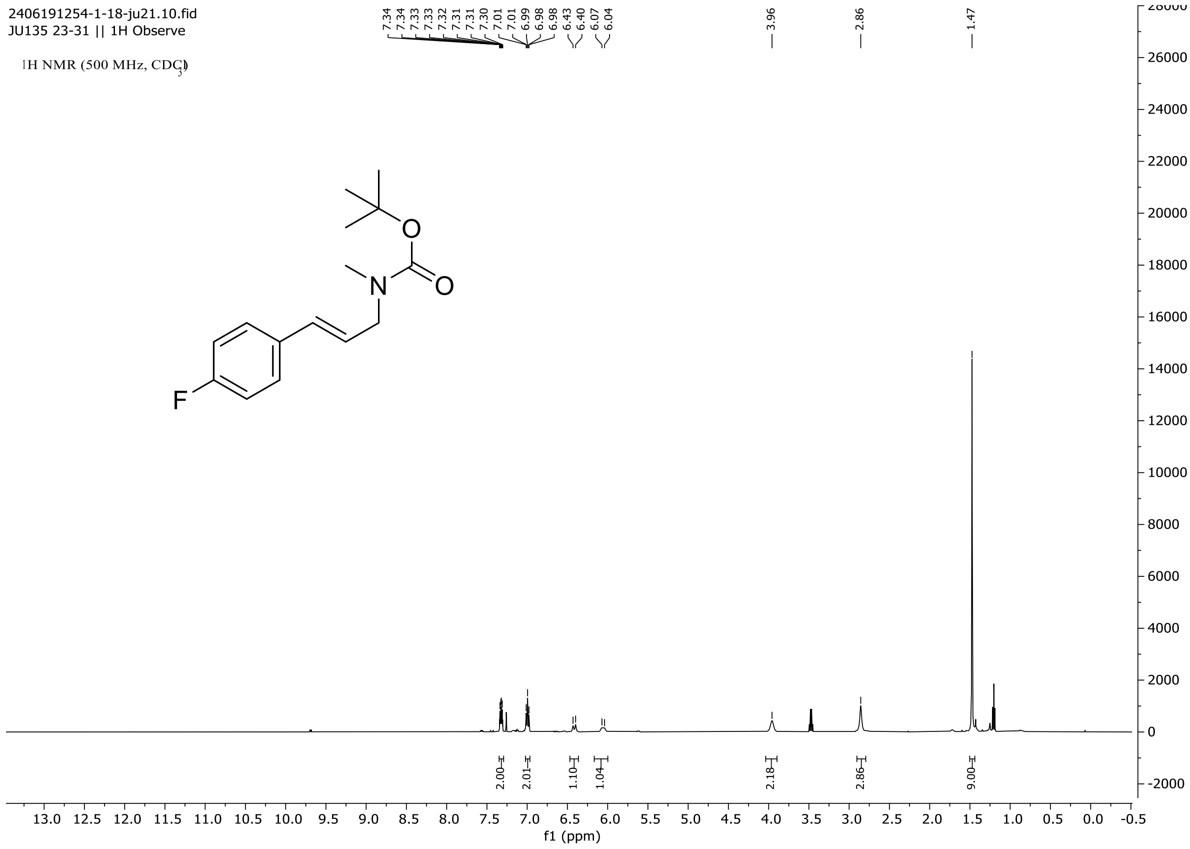

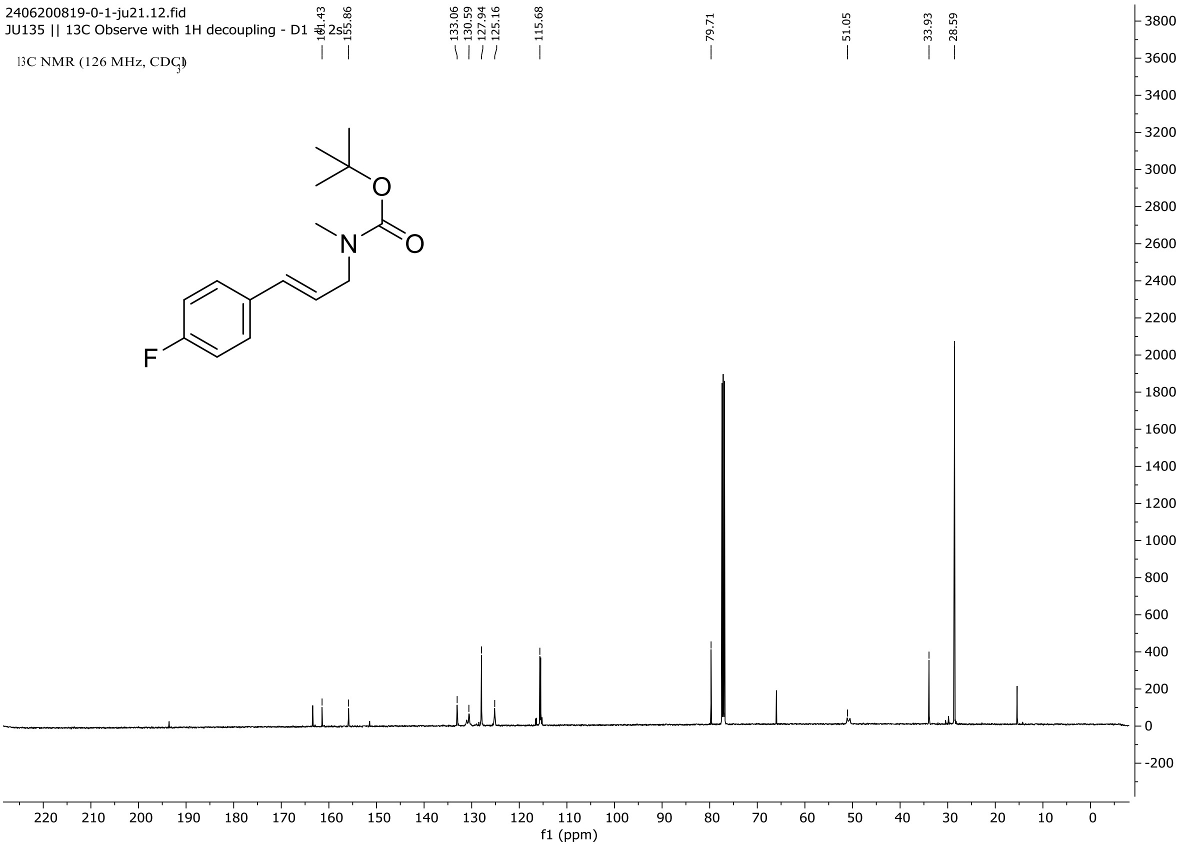
2406200819-0-1-ju21.12.fid
JU135 || 13C Observe with 1H decoupling - D1 = 2s
13C NMR (126 MHz, CDCl3)
155.86
133.06
130.59
127.94
125.16
115.68
79.71
51.05
33.93
28.59
F
N
O
O
f1 (ppm)

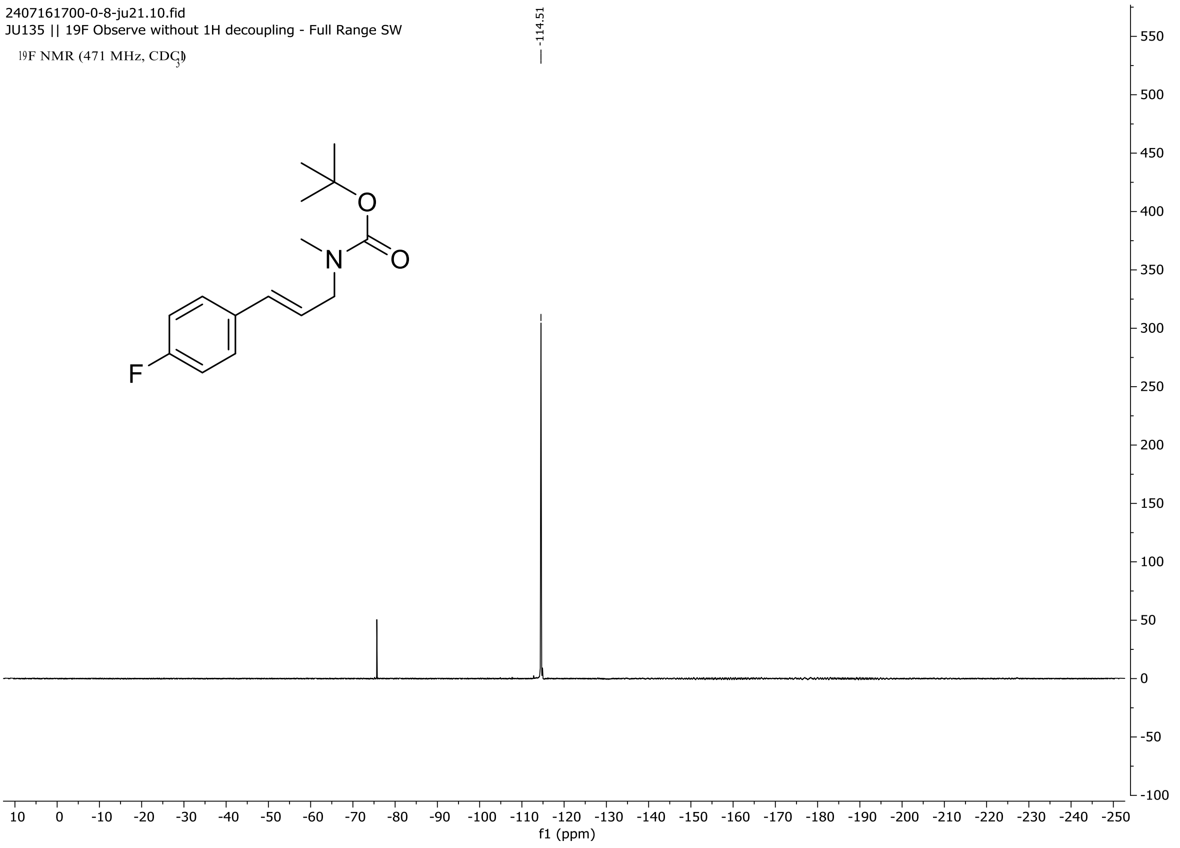
2407161700-0-8-ju21.10.fid
JU135 || 19F Observe without 1H decoupling - Full Range SW
19F NMR (471 MHz, CDCl3)
-114.51
F
N
O
O
f1 (ppm)

tert-Butyl (*E*)-methyl(3-(pyridine-2-yl)allyl)carbamate (14)

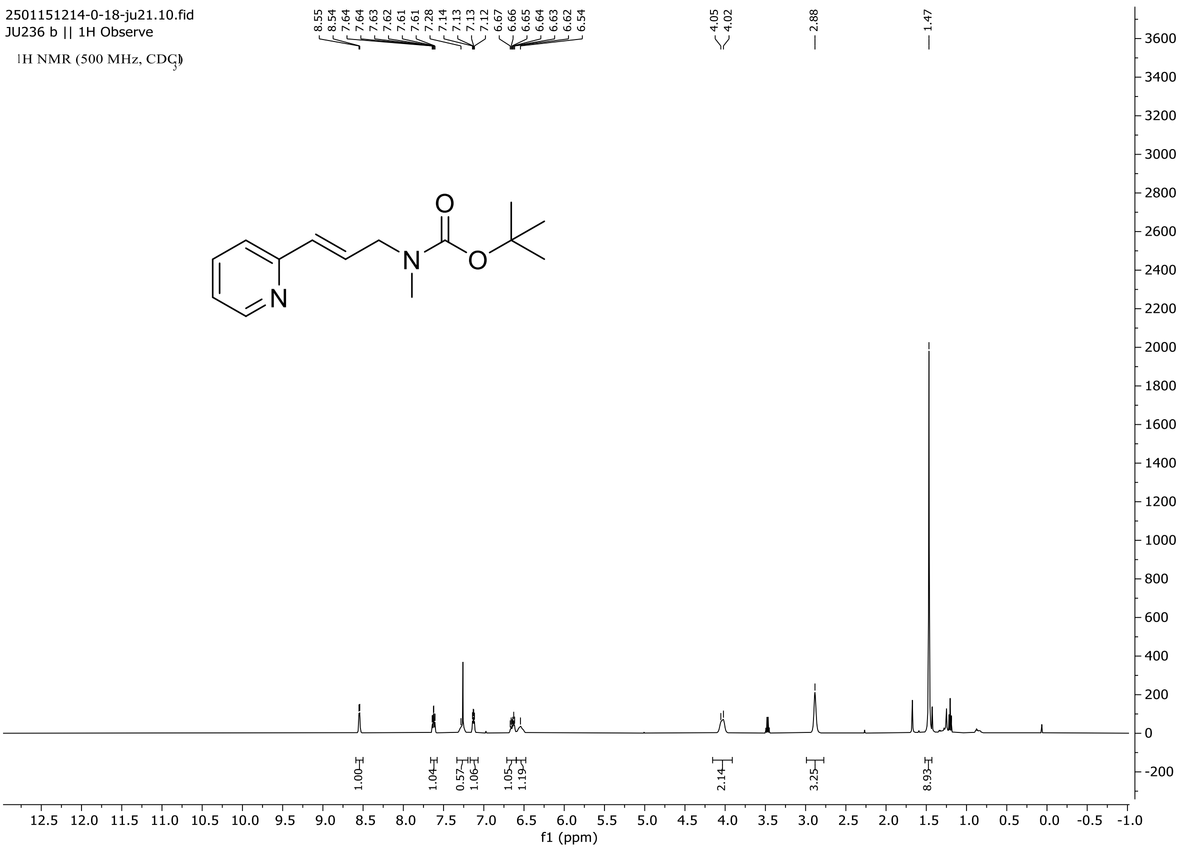

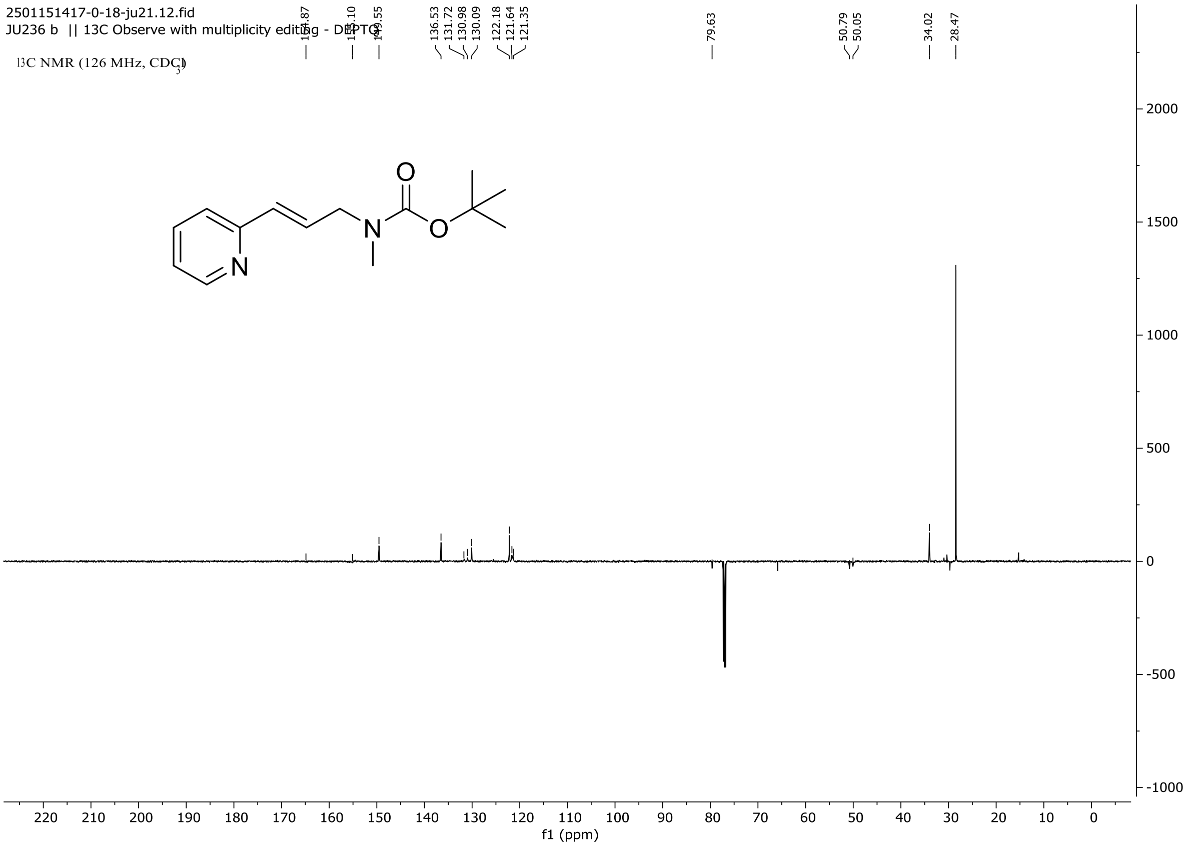

***tert*-Butyl (*E*)-4-(2-((*tert*-butoxycarbonyl)amino)-4-phenylbut-3-en-1-yl)-1*H*-imidazole-1-carboxylate (15)**

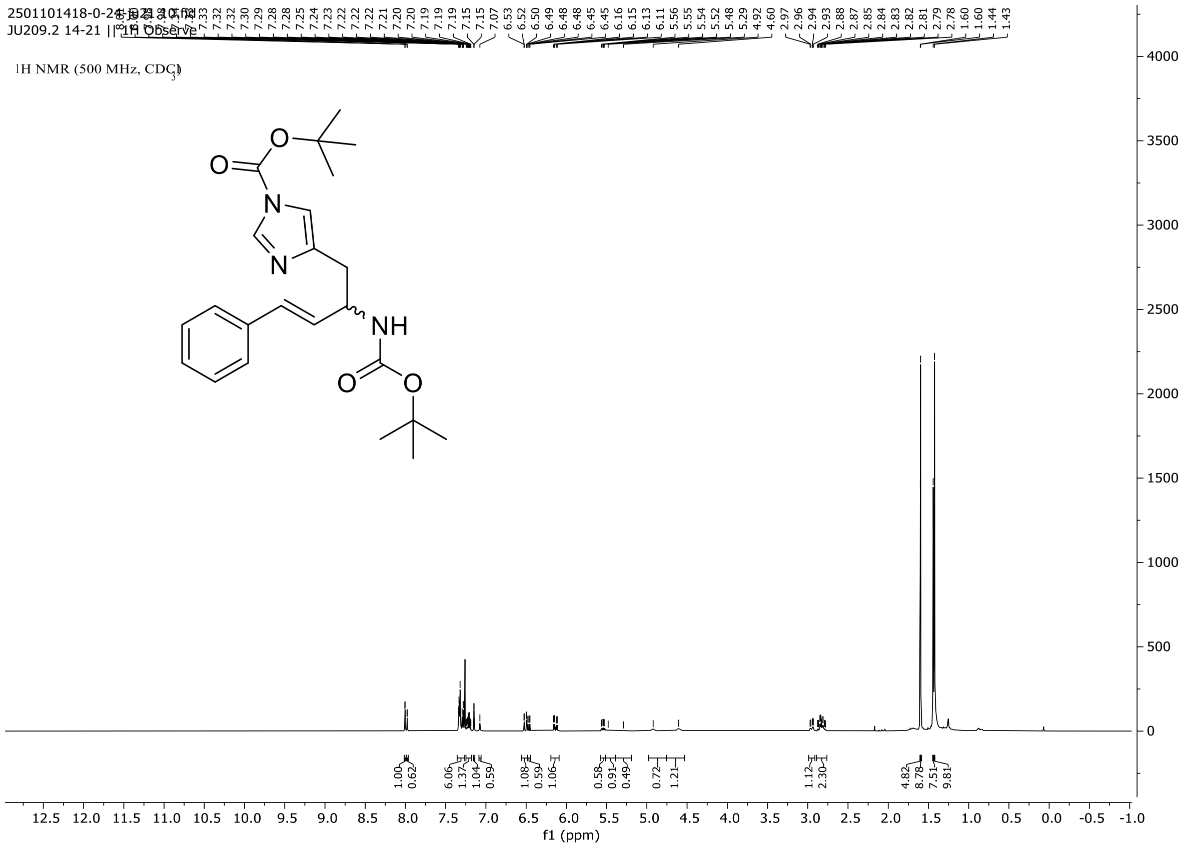

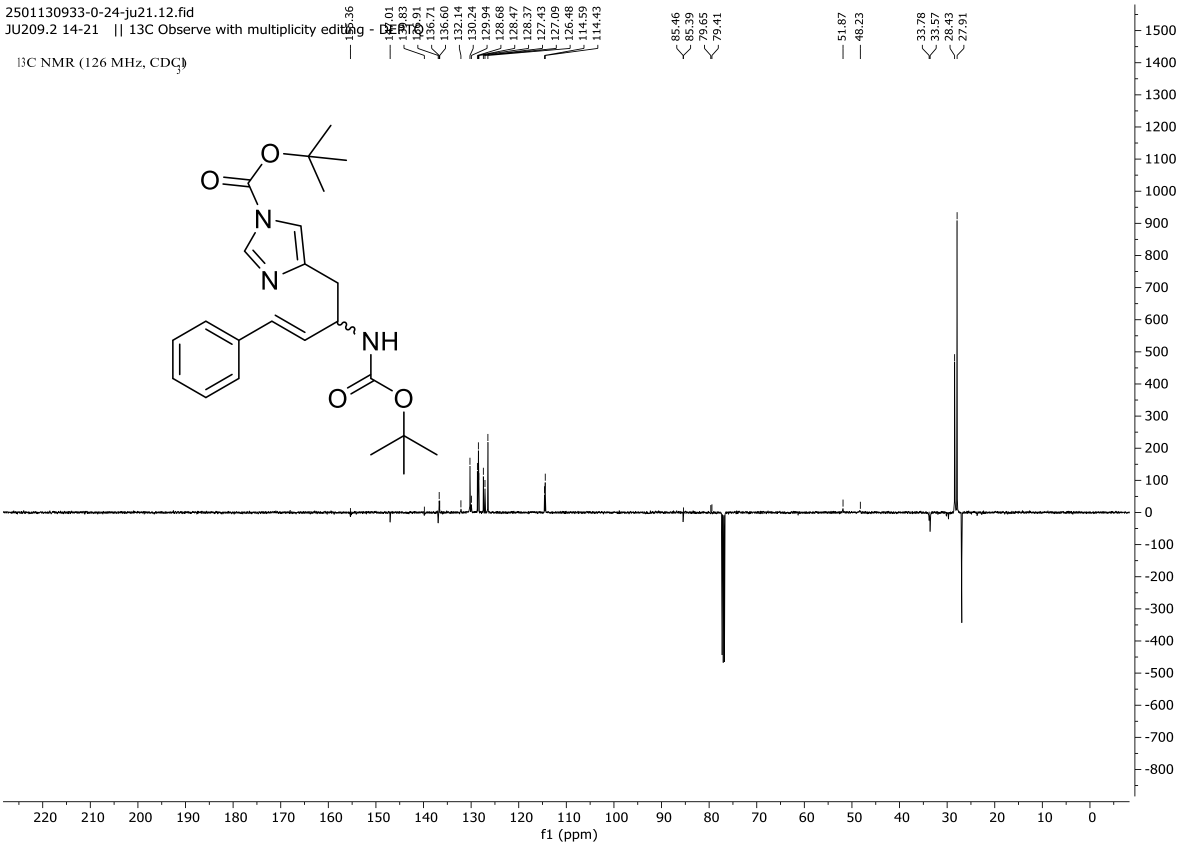

Methyl (*E*)-3-(3-((*tert*-butoxycarbonyl)amino)prop-1-1en-1-yl)benzoate (16)

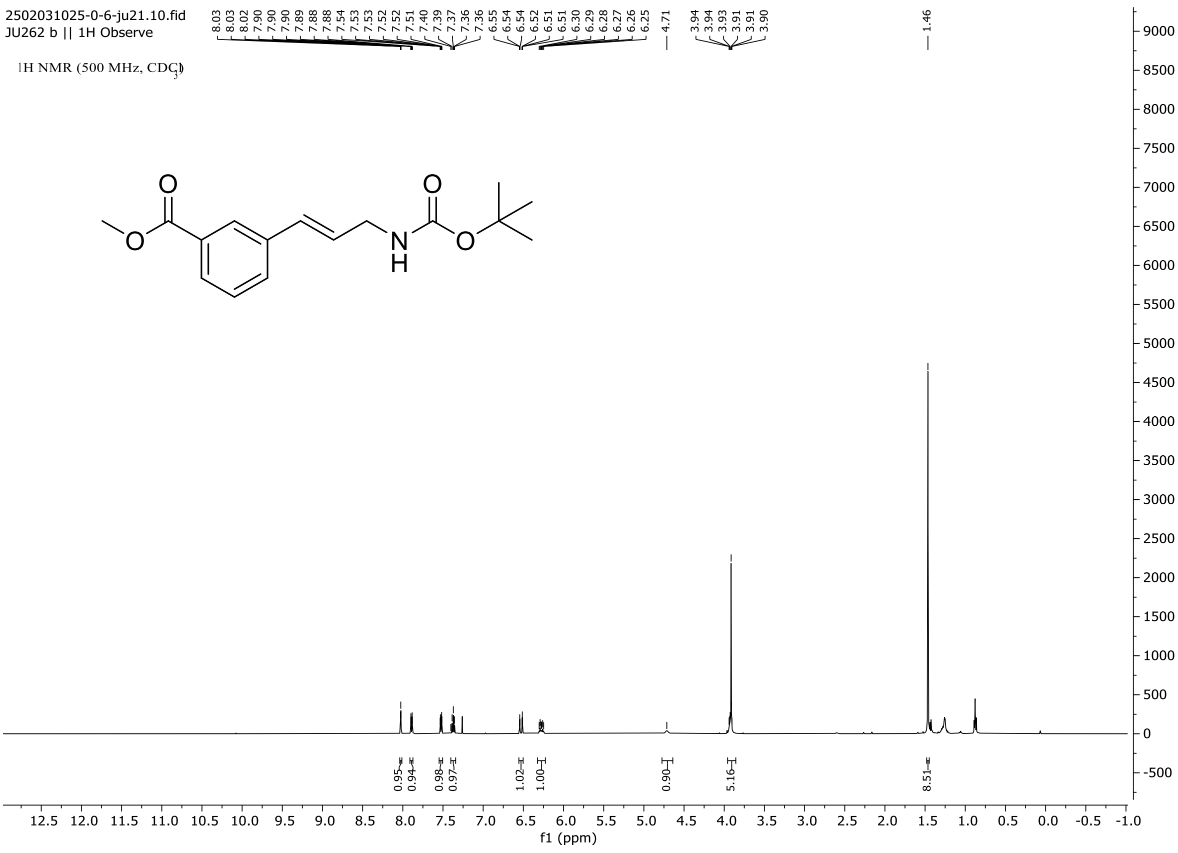

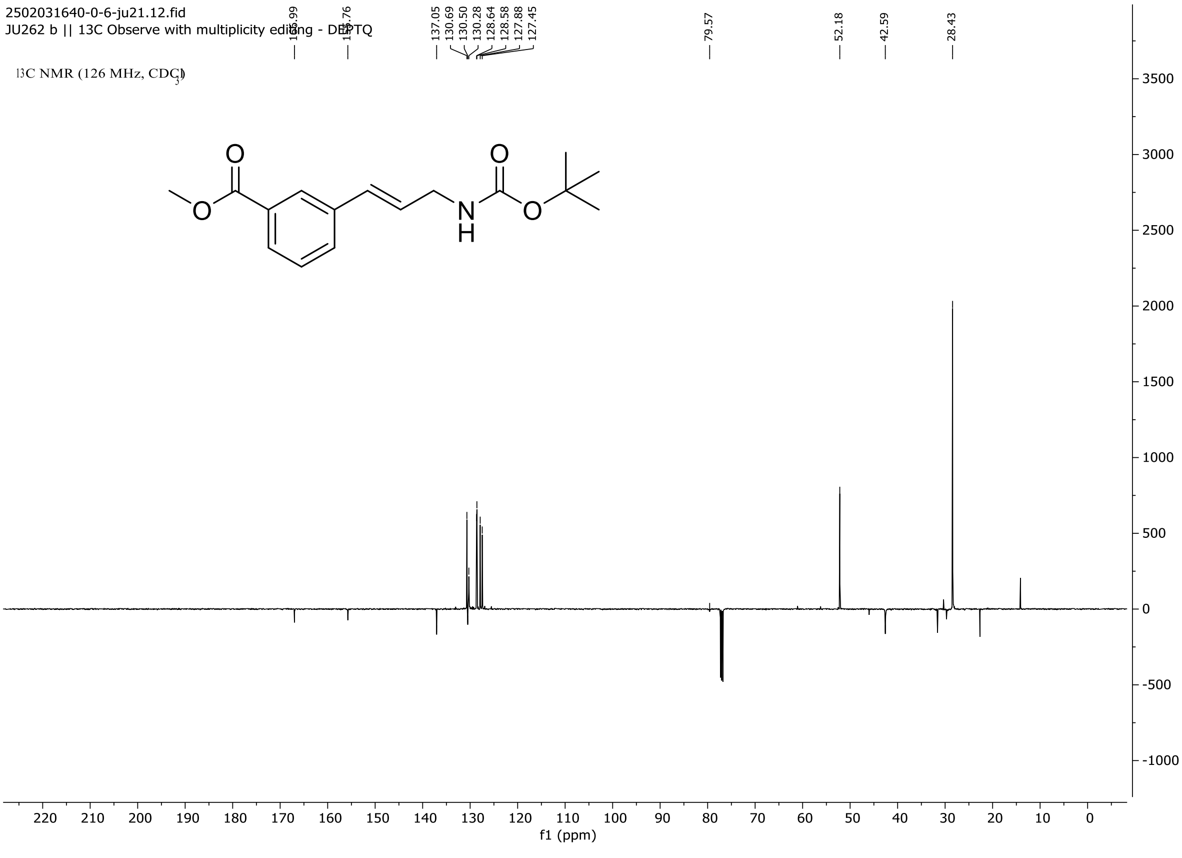

tert-Butyl (*E*)-(4-phenylbut-3-en-1-yl)carbamate (17)

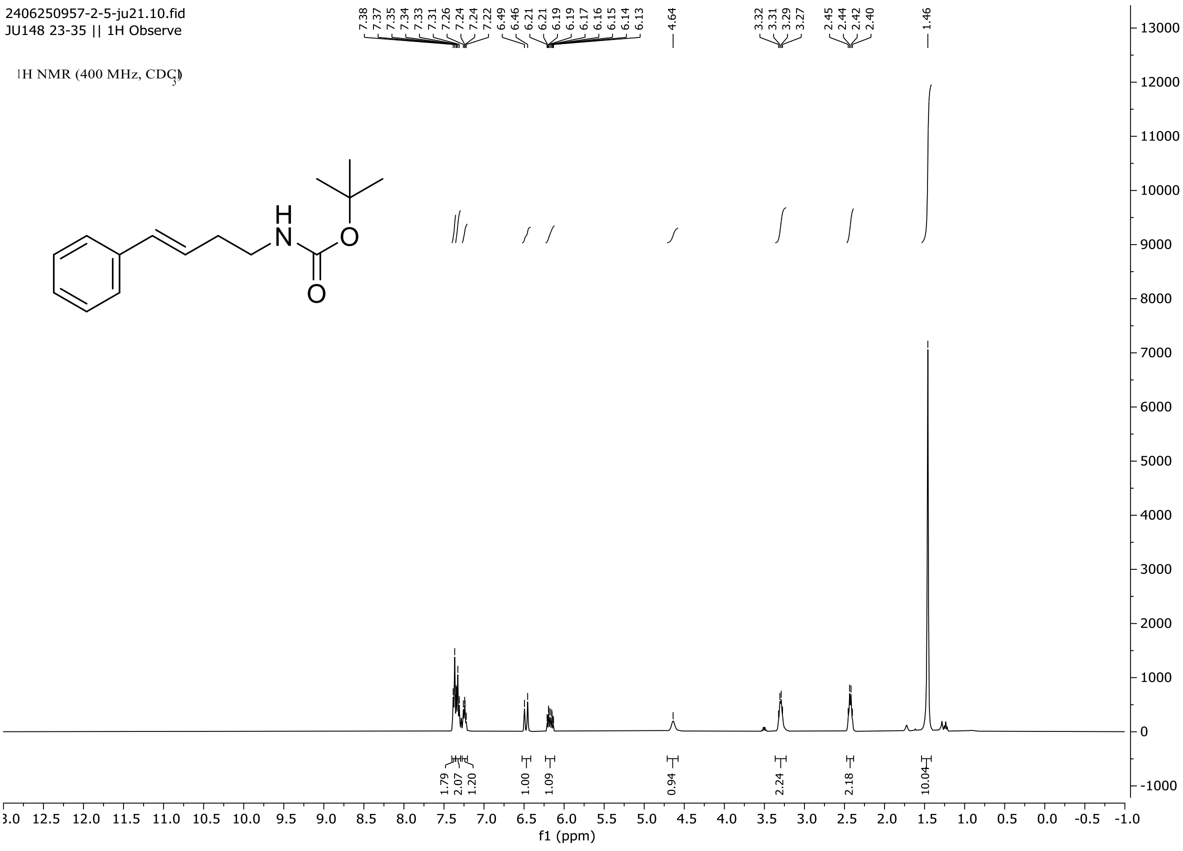

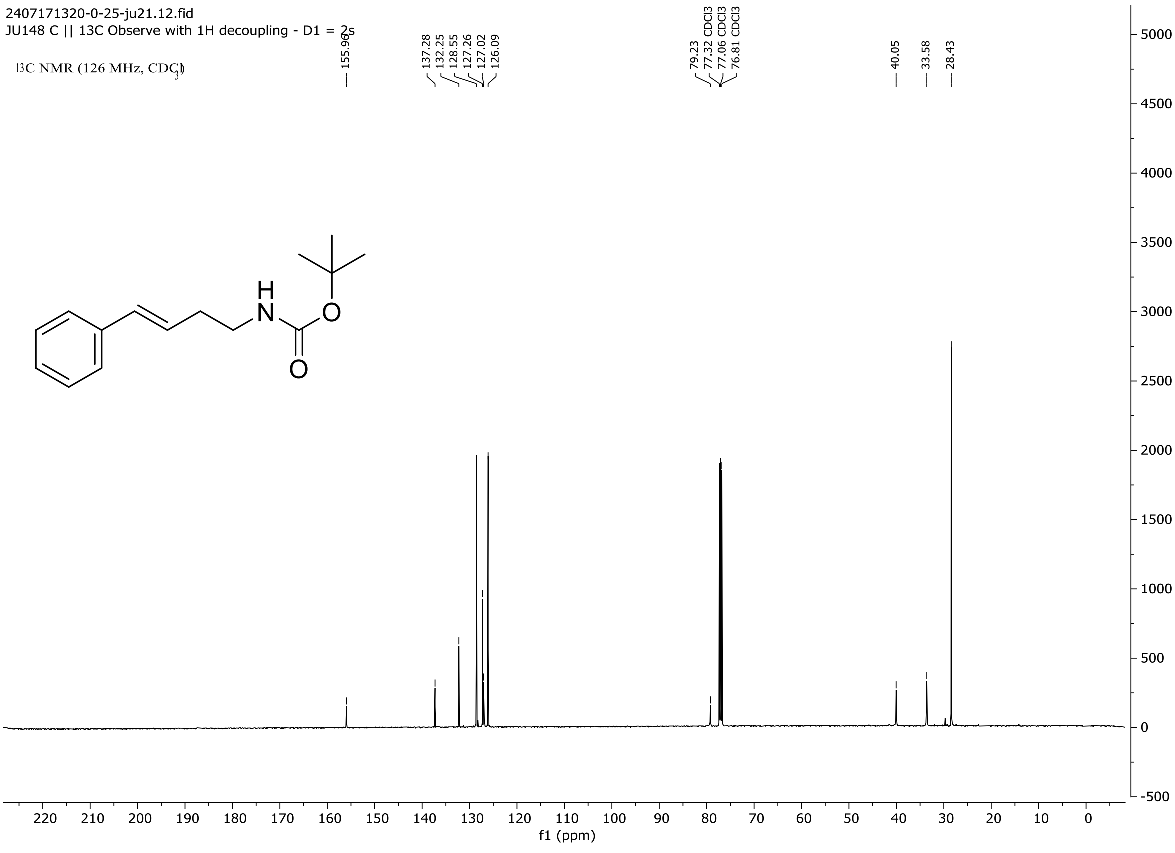

tert-Butyl (*R*,*E*)-(5-(4-methoxyphenyl)pent-4-en-2-yl)carbamate (19)

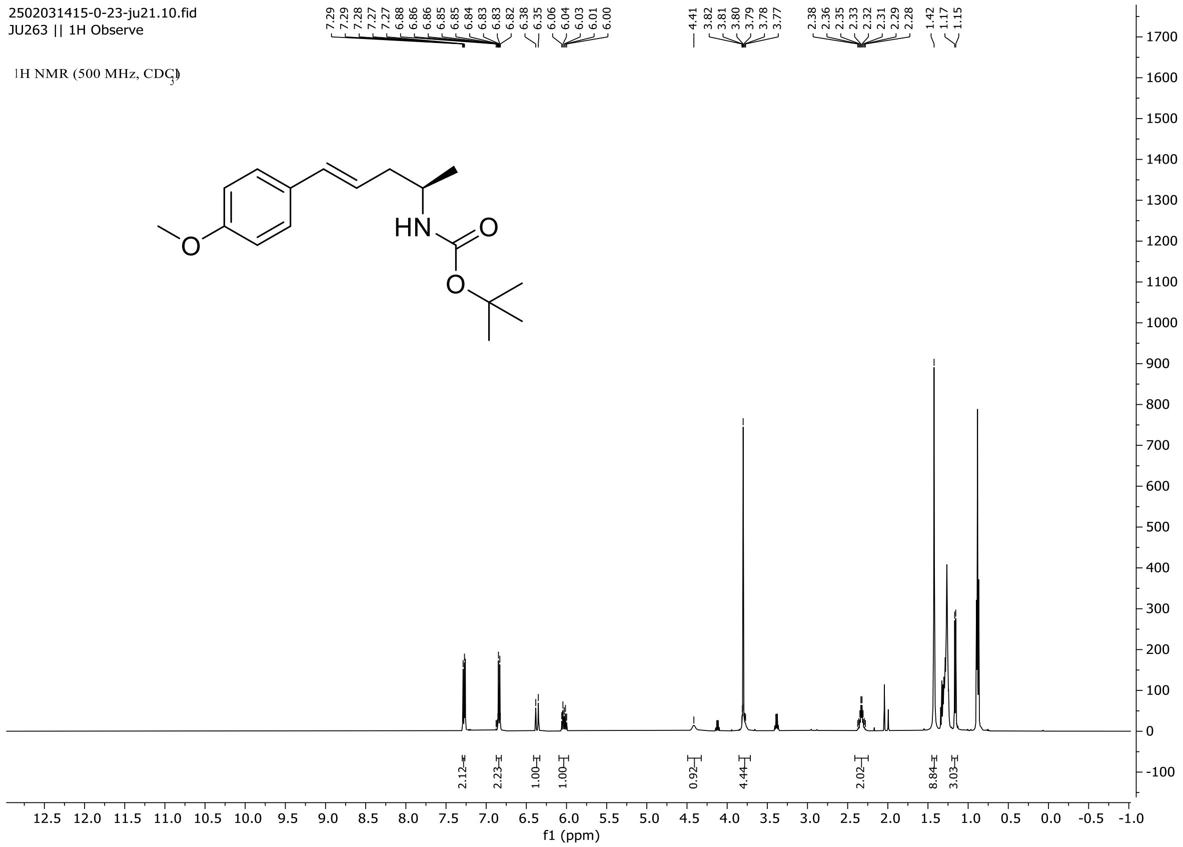

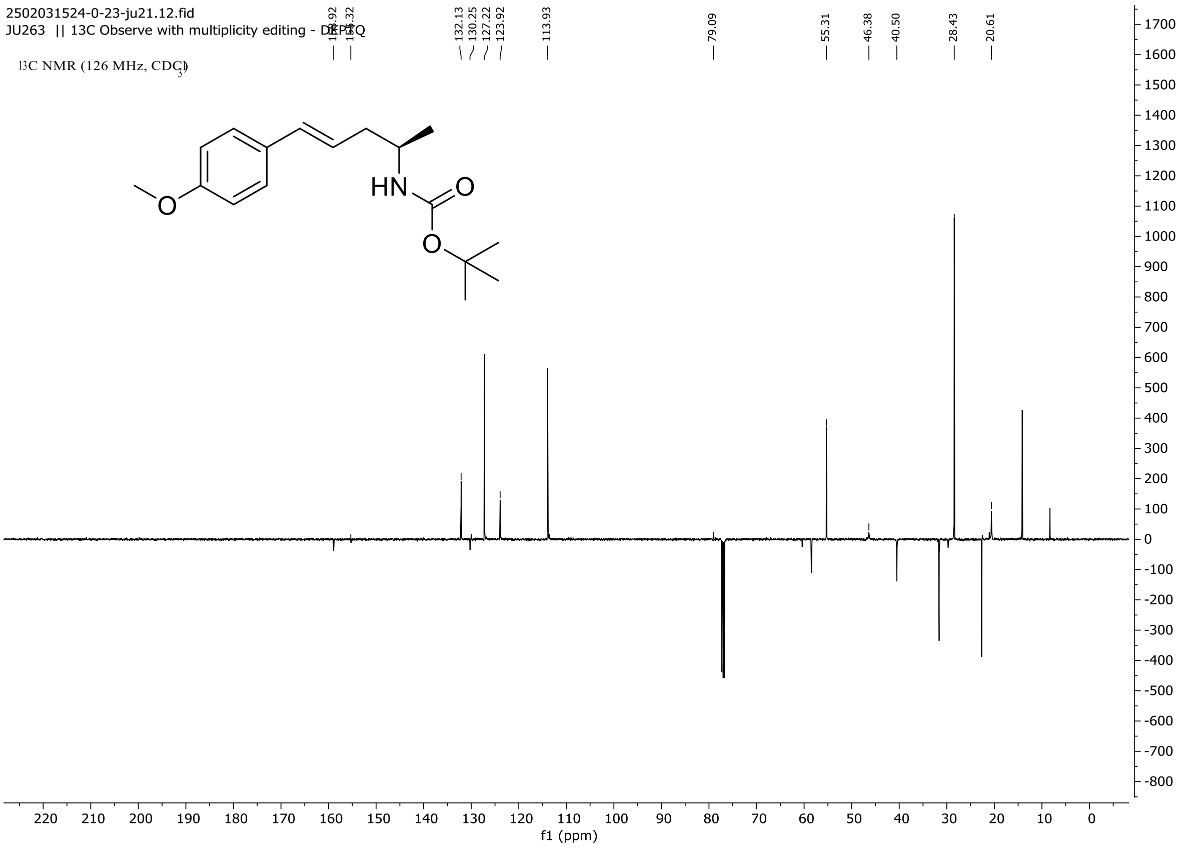

tert-Butyl (*E*)-(5-(phenyl)pent-4-en-2-yl)carbamate (20)

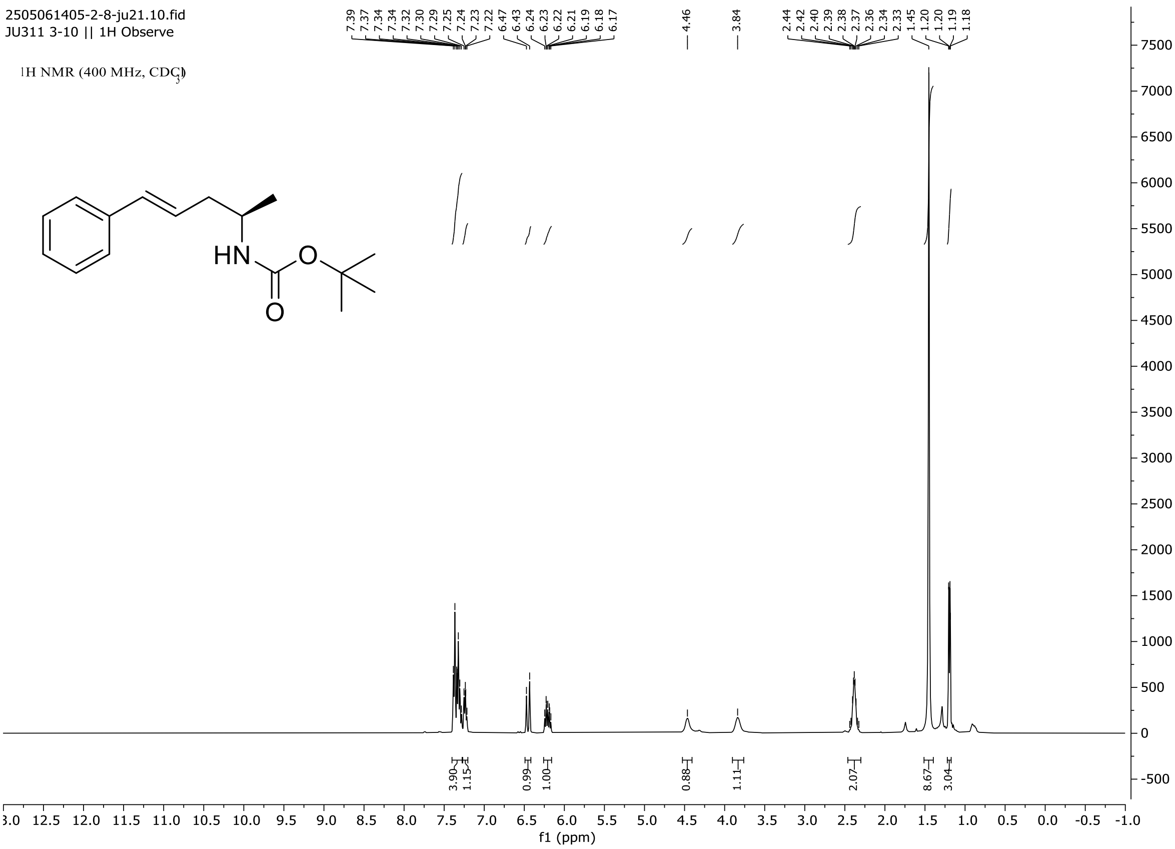

tert-Butyl (*R*,*E*)-(5-(2-bromophenyl)pent-4-en-2-yl)carbamate (21)

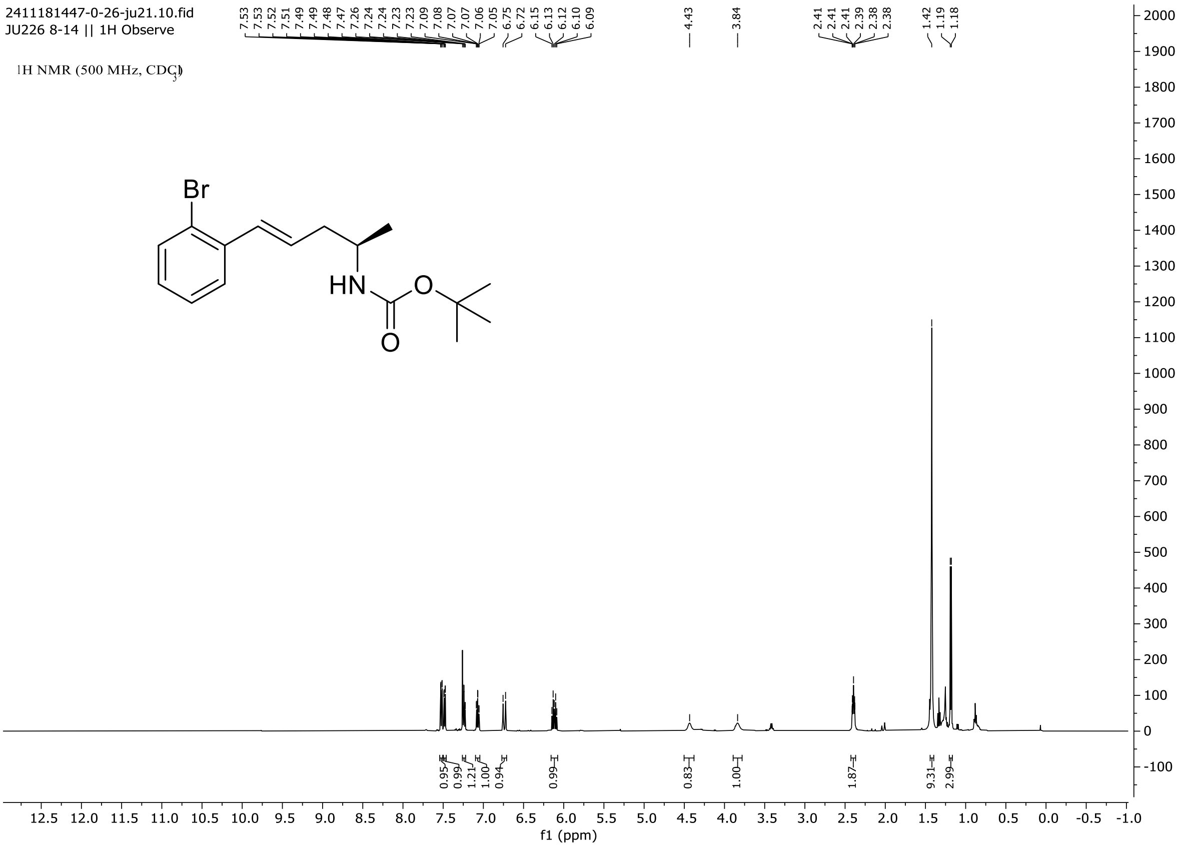

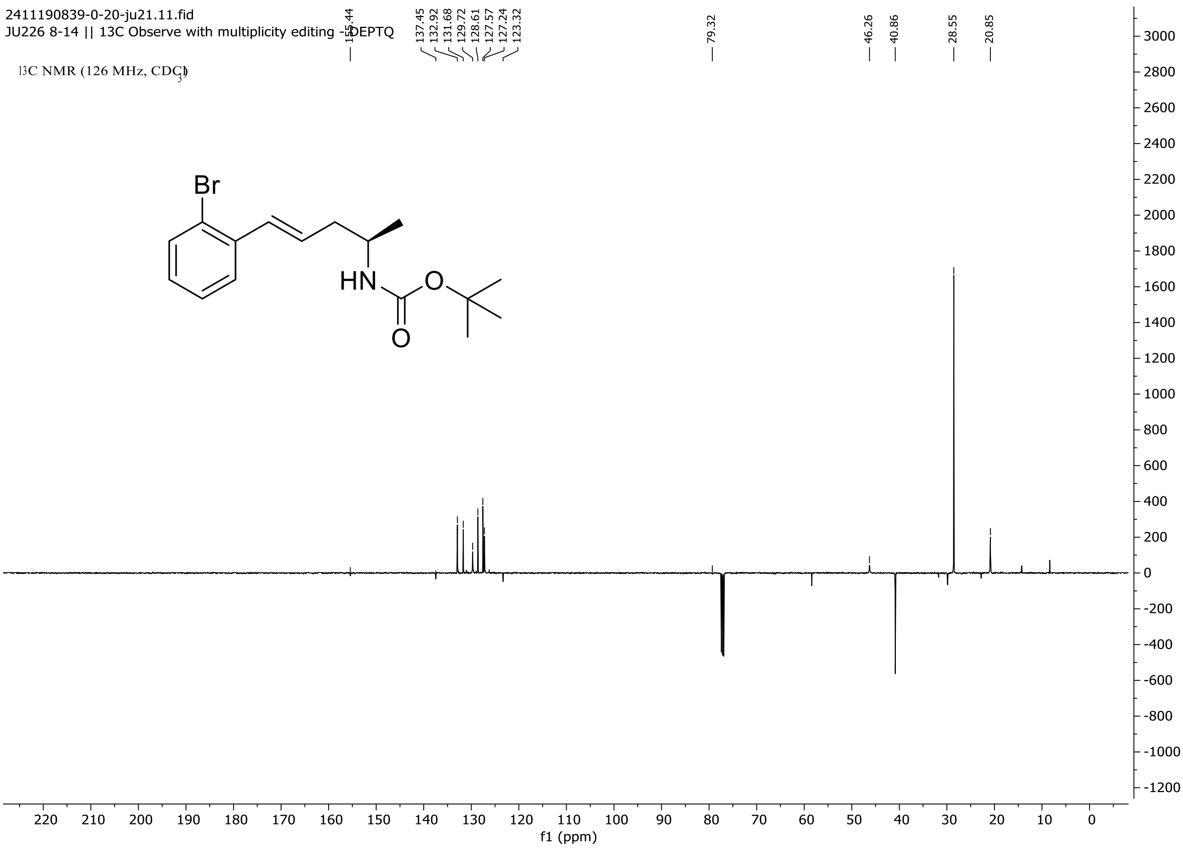

tert-Butyl (*E*)-(4-phenyl-1-(*p*-tolyl)but-3-en-1-yl)carbamate (22)

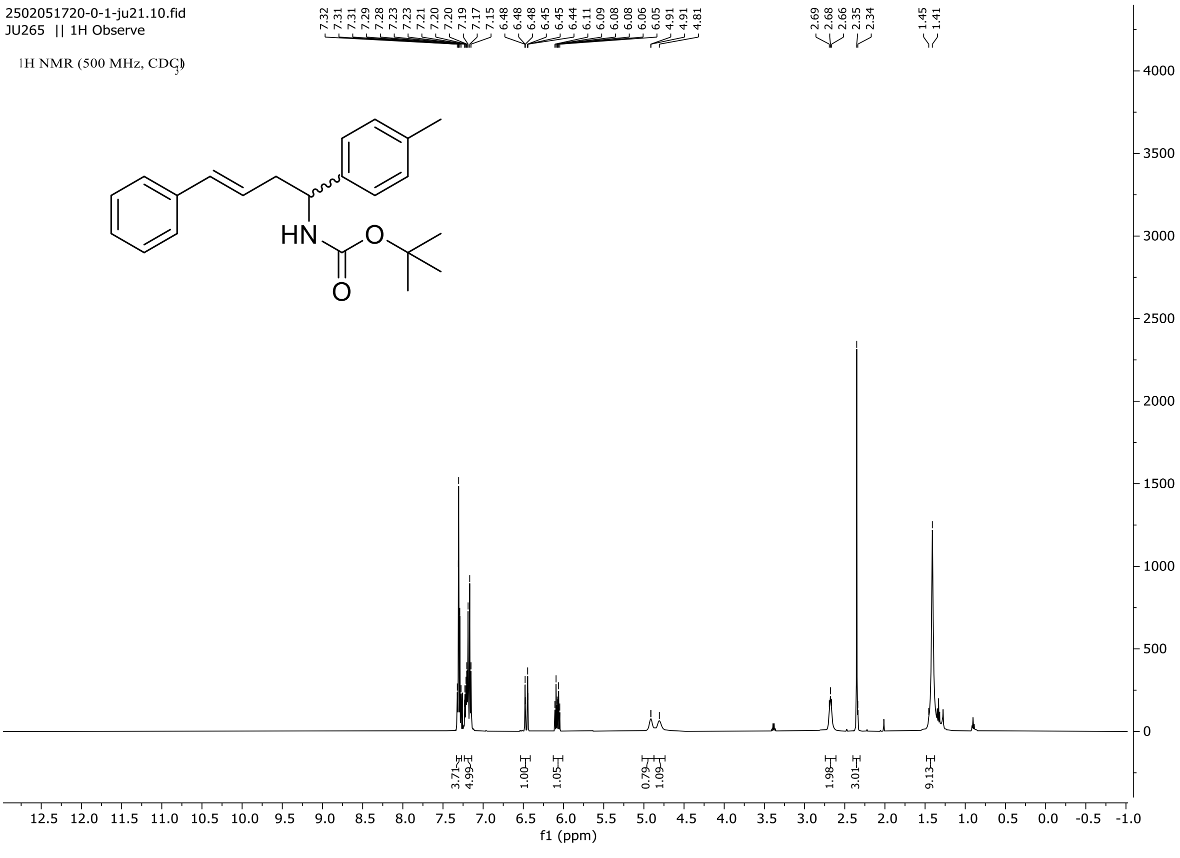

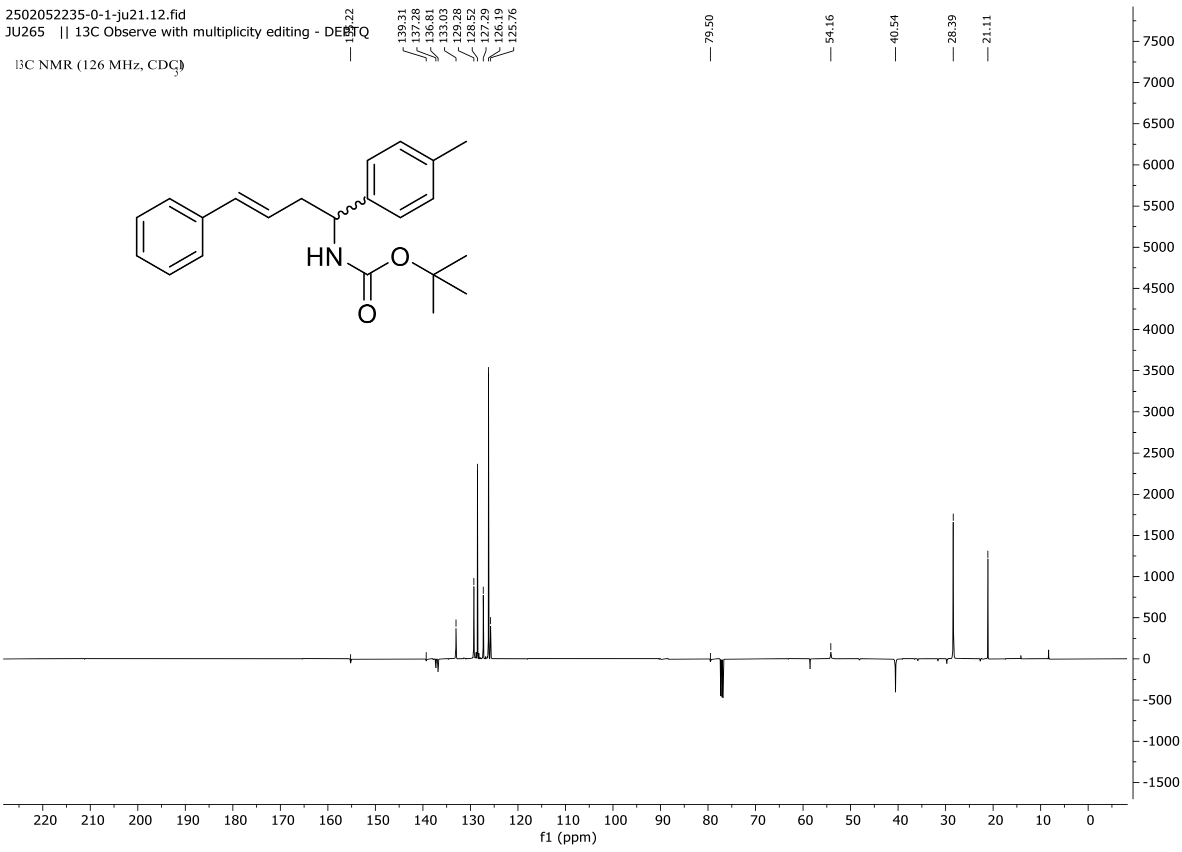

tert-Butyl (*E*)-(4-(4-chlorophenyl)-2-(*p*-tolyl)but-3-en-1-yl)carbamate (23)

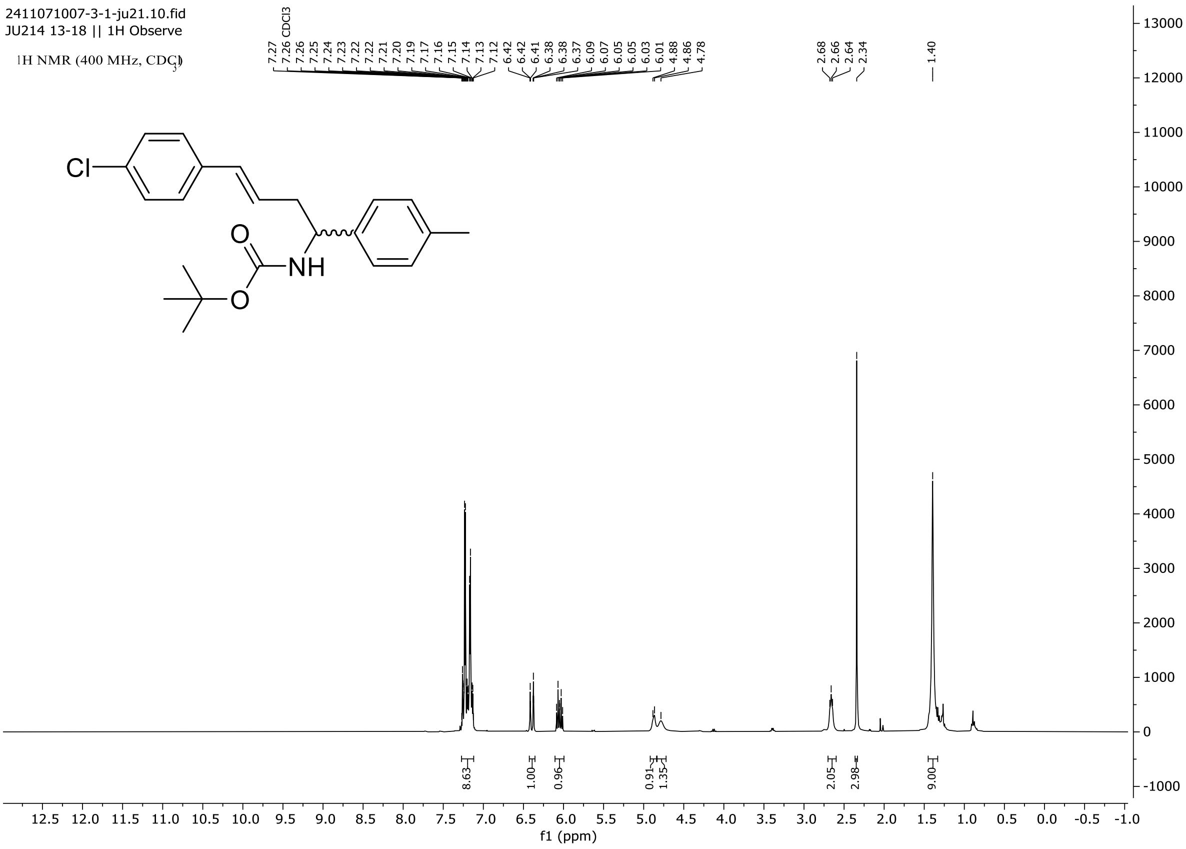

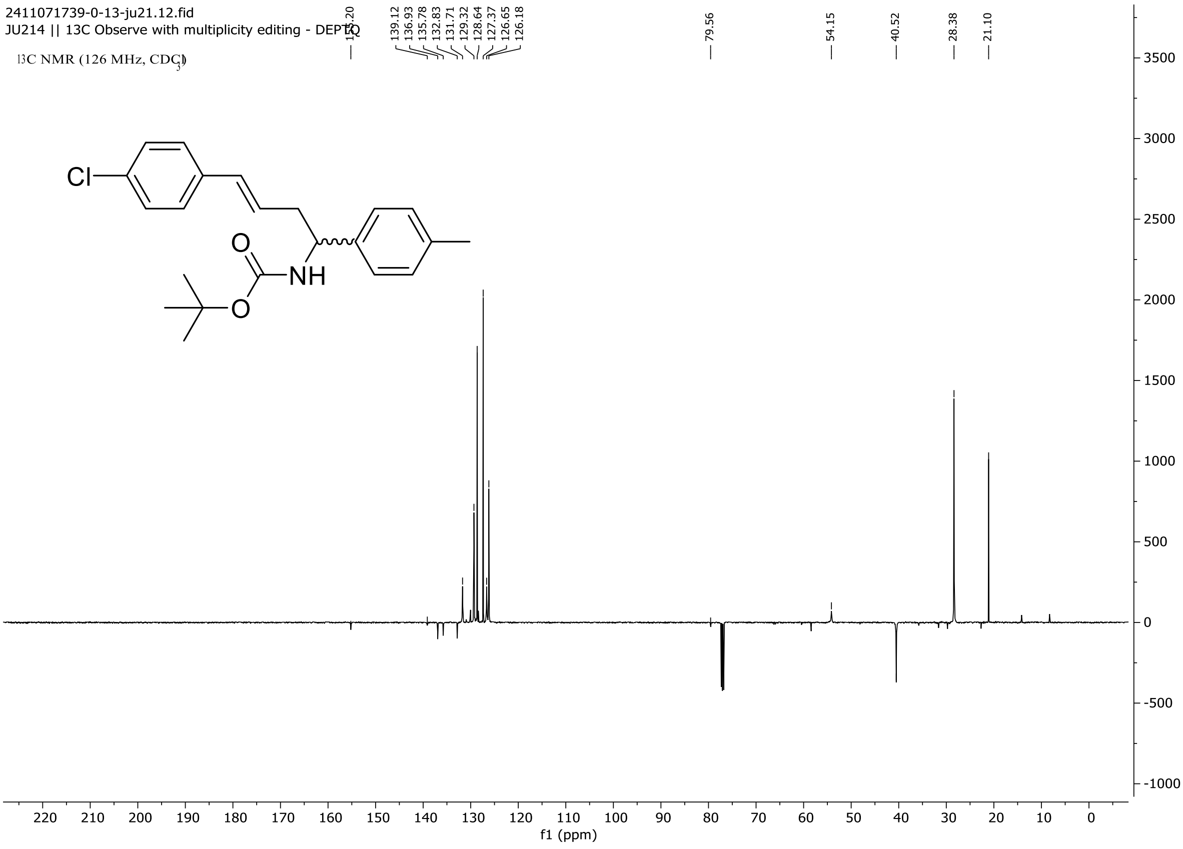

tert-Butyl (*E*)-(2-styrylcyclopentyl)carbamate (25)

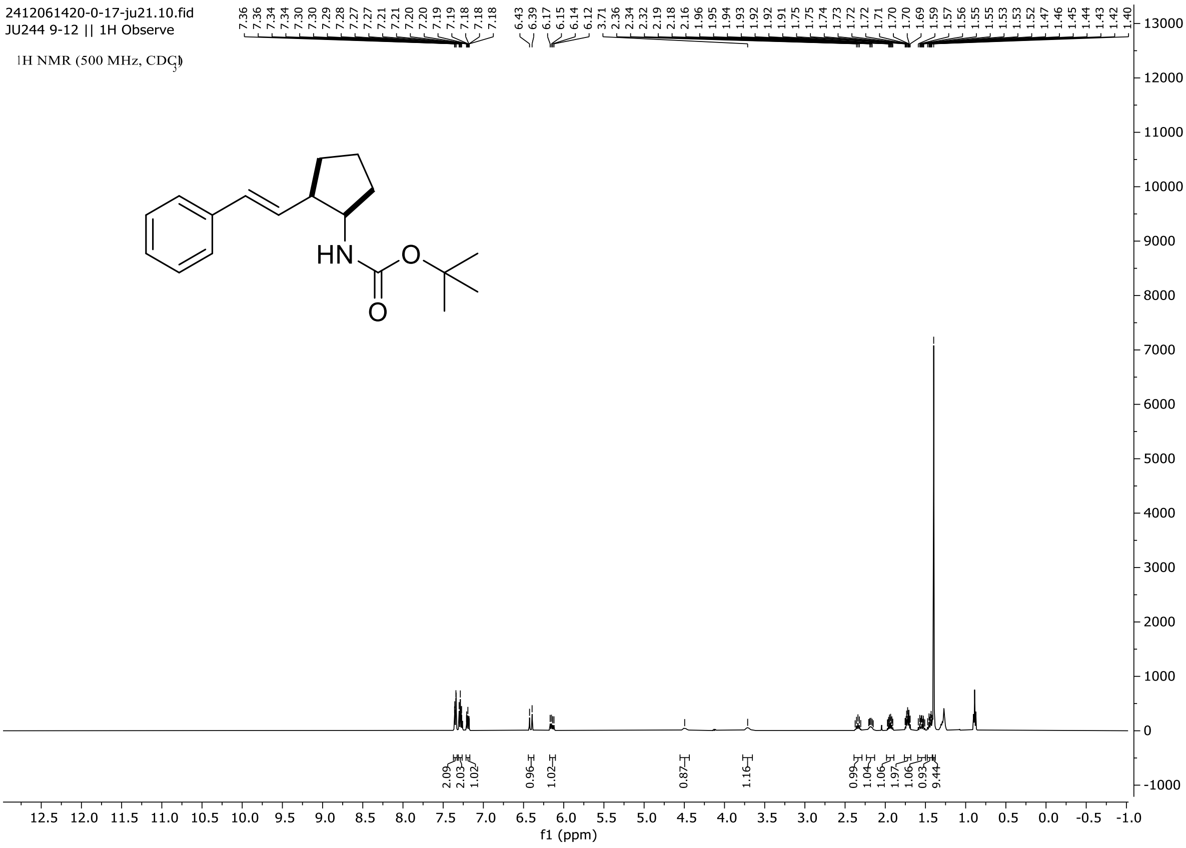

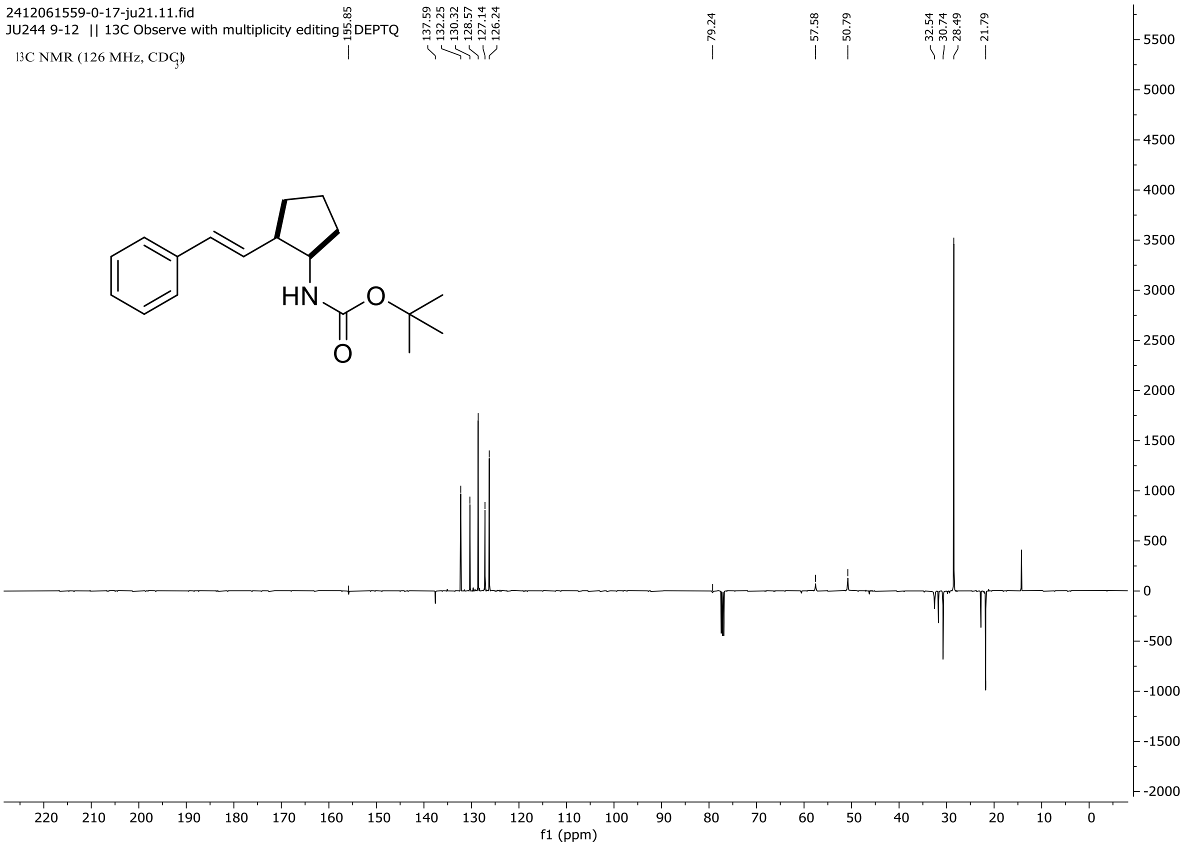

***tert*-Butyl (*R*)-(1-(3,4-dihydronaphthalen-2-yl)propan-2-yl)carbamate (26)**

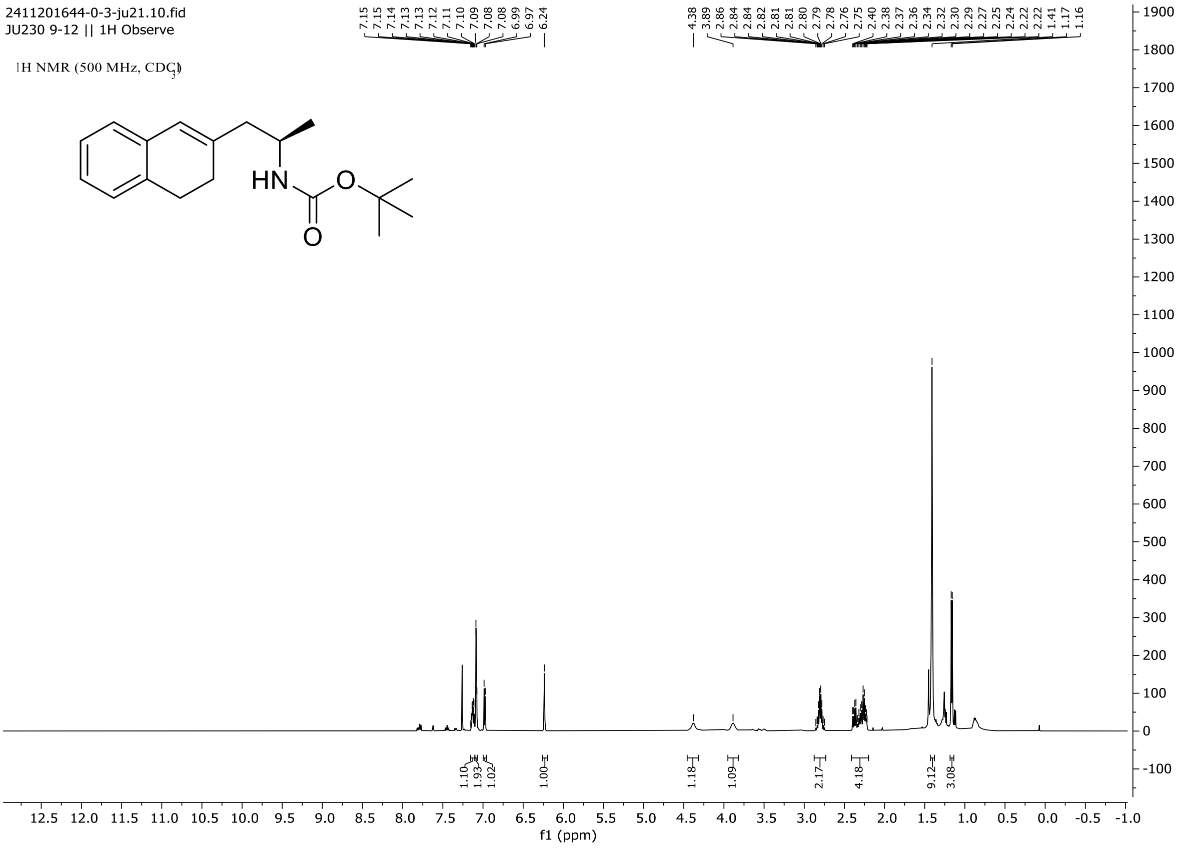

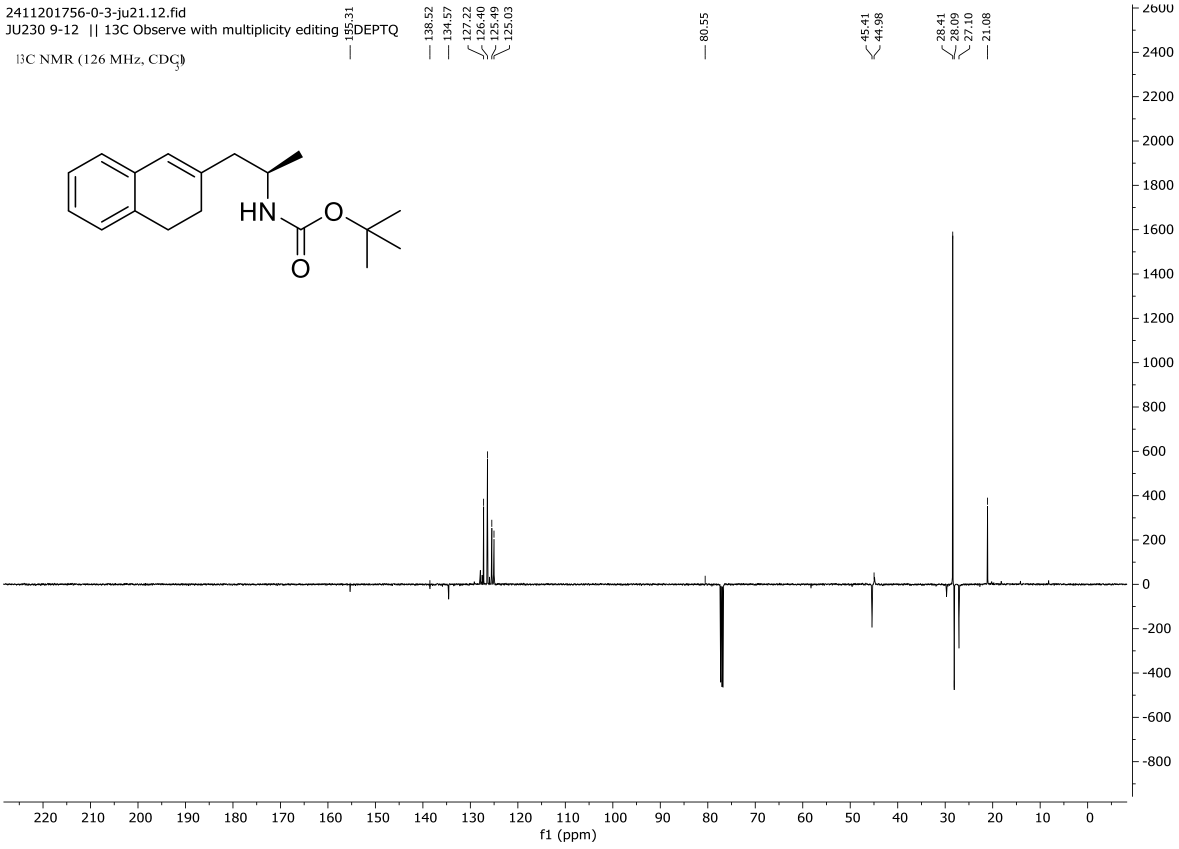

(*E*)-*N*-(3-(4-chlorophenyl)allyl)acetamide (9)

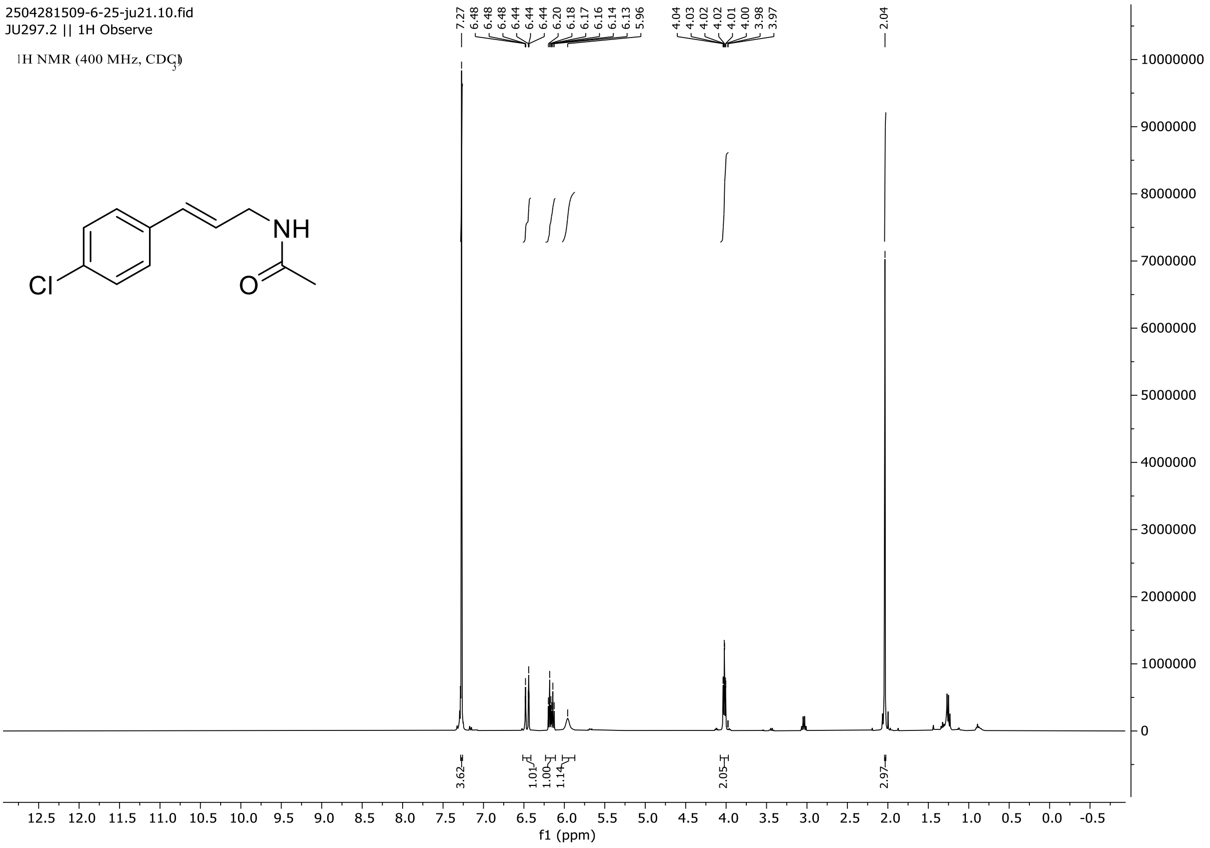

(*E*)-*N*-(4-phenylbut-3-en-1-yl)acetamide (18)

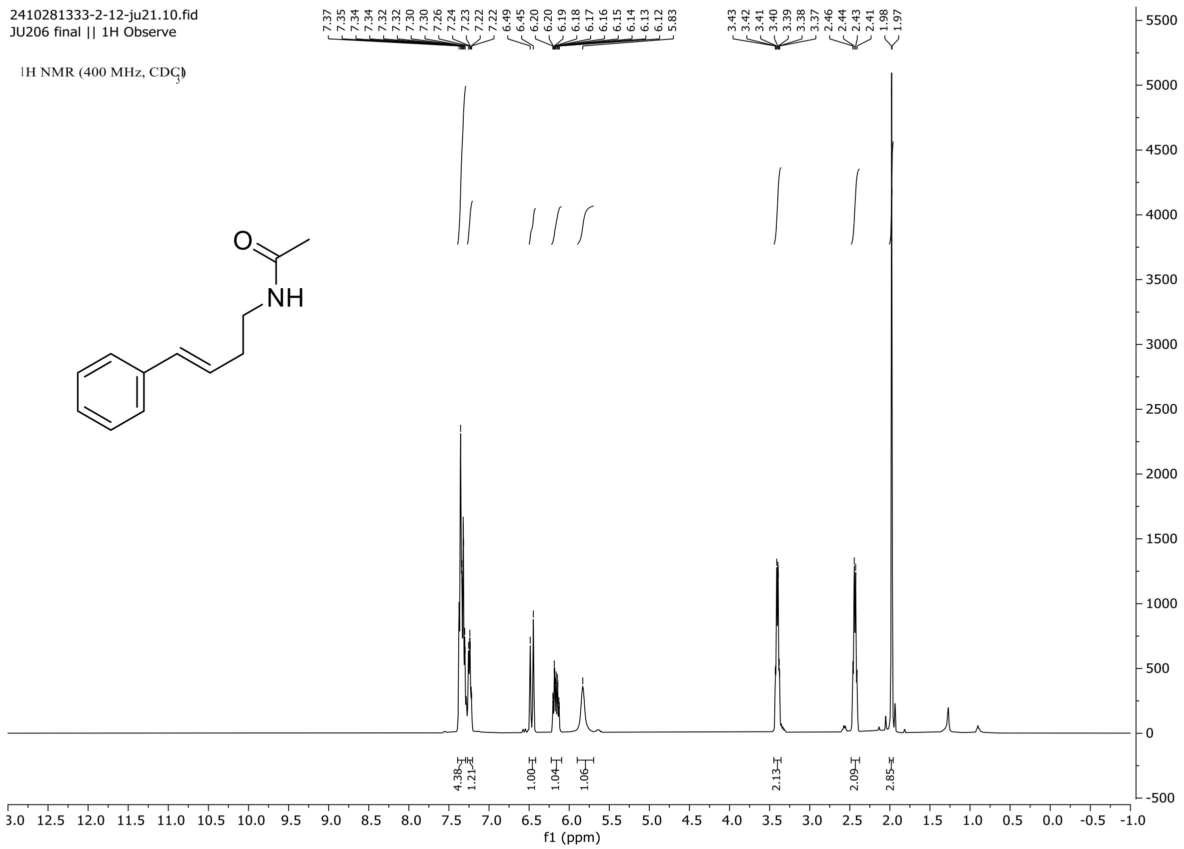

(*E*)-*N*-(4-(4-Chlorophenyl)-1-(*p*-tolyl)but-3-en-1-yl)acetamide (24)

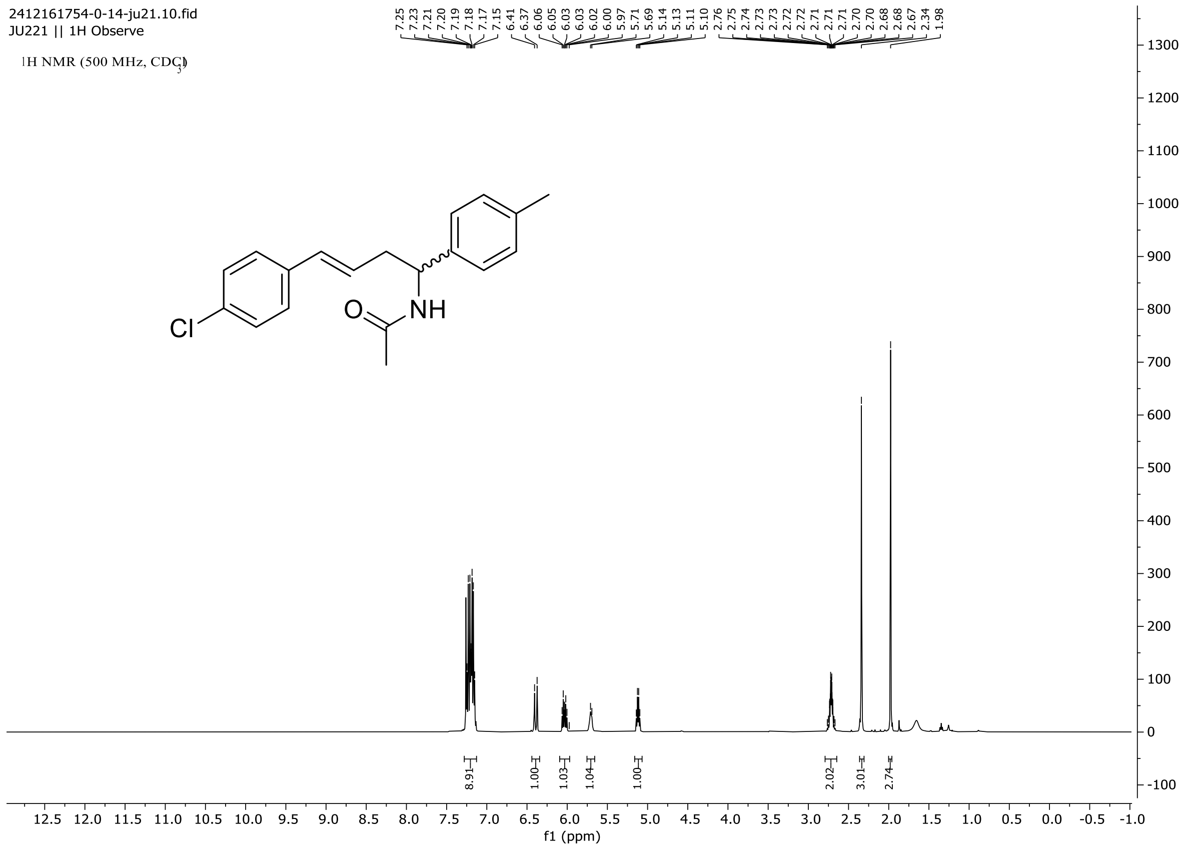

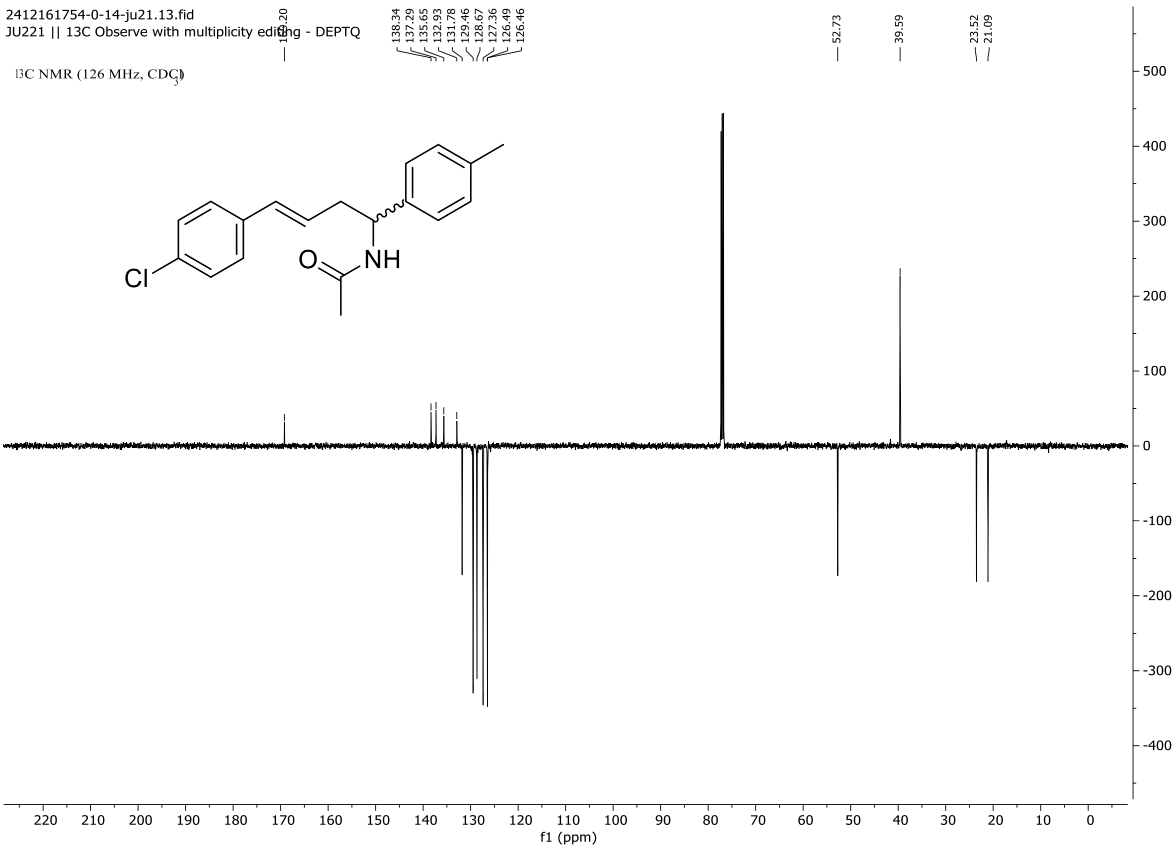

5-Iodo-6-phenyl-1,3-oxazinan-2-one (27)

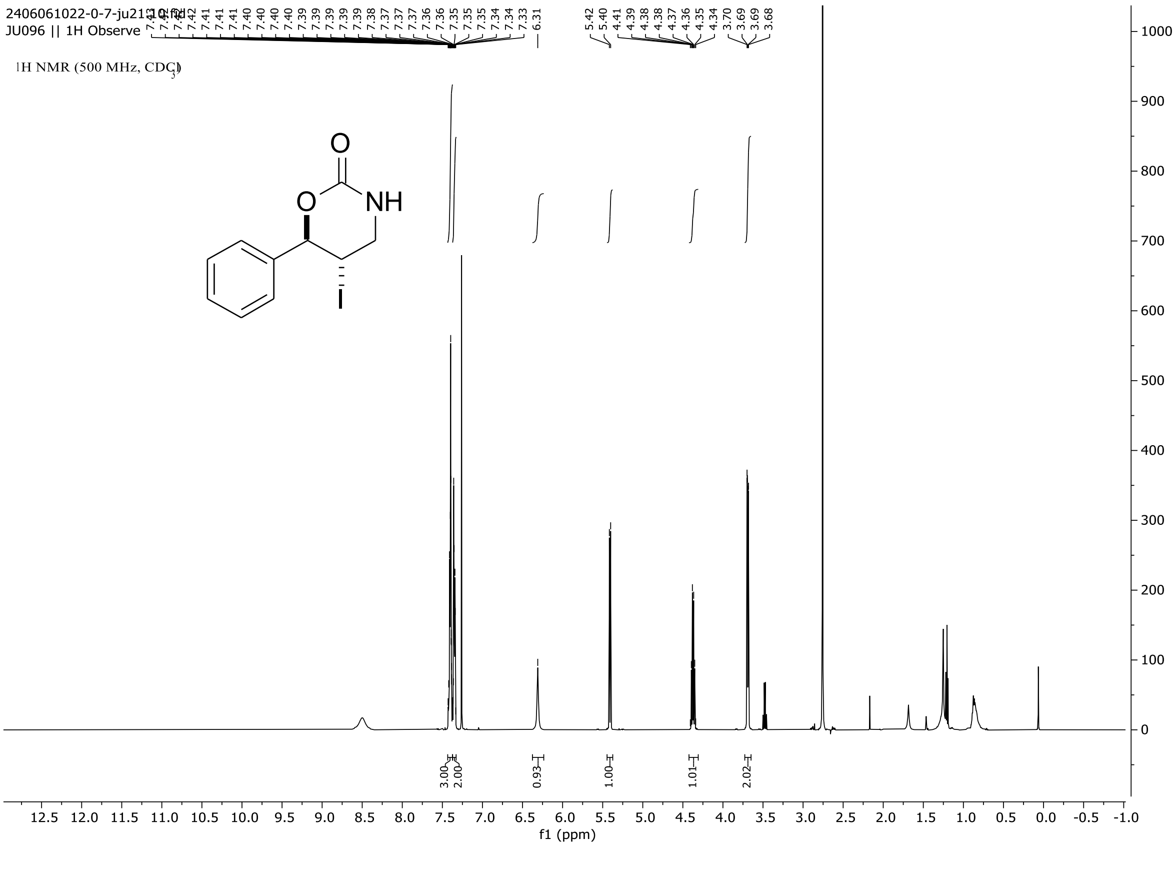

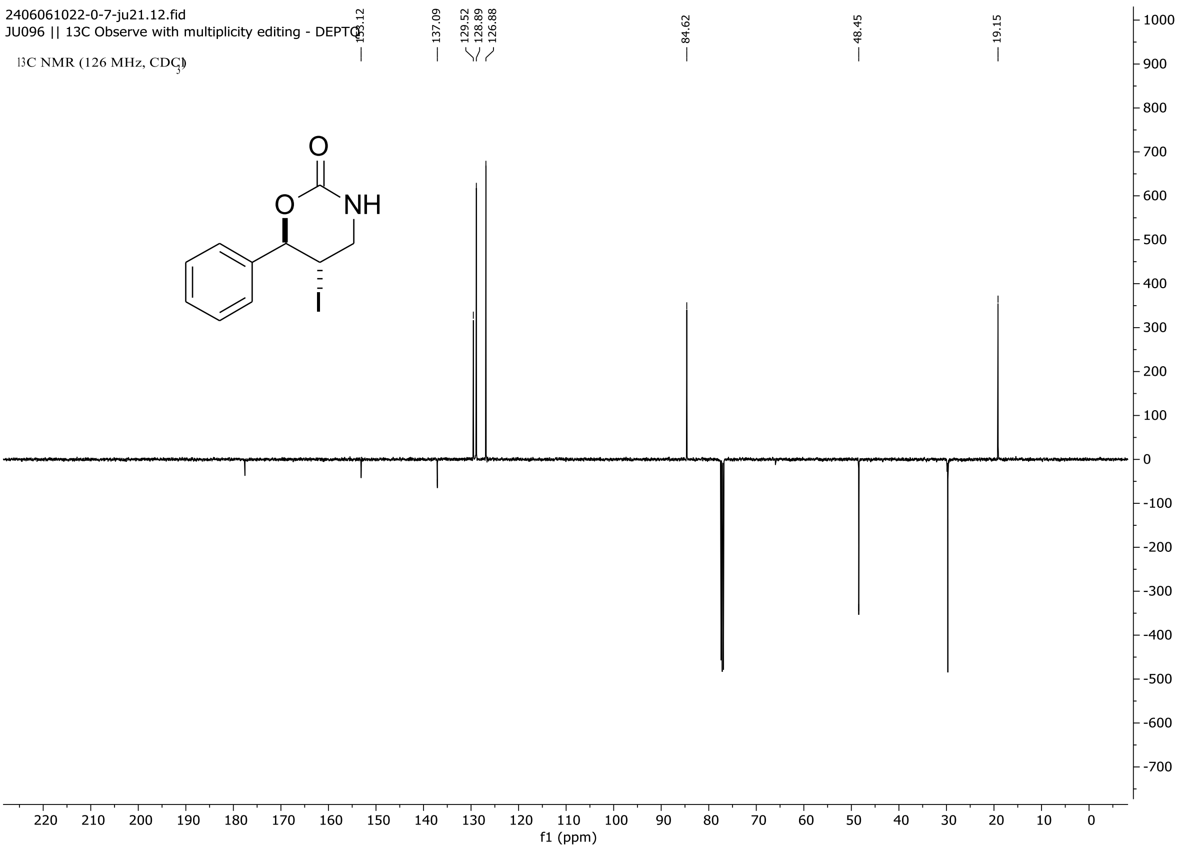

5-Bromo-6-phenyl-1,3-oxazinan-2-one (28)

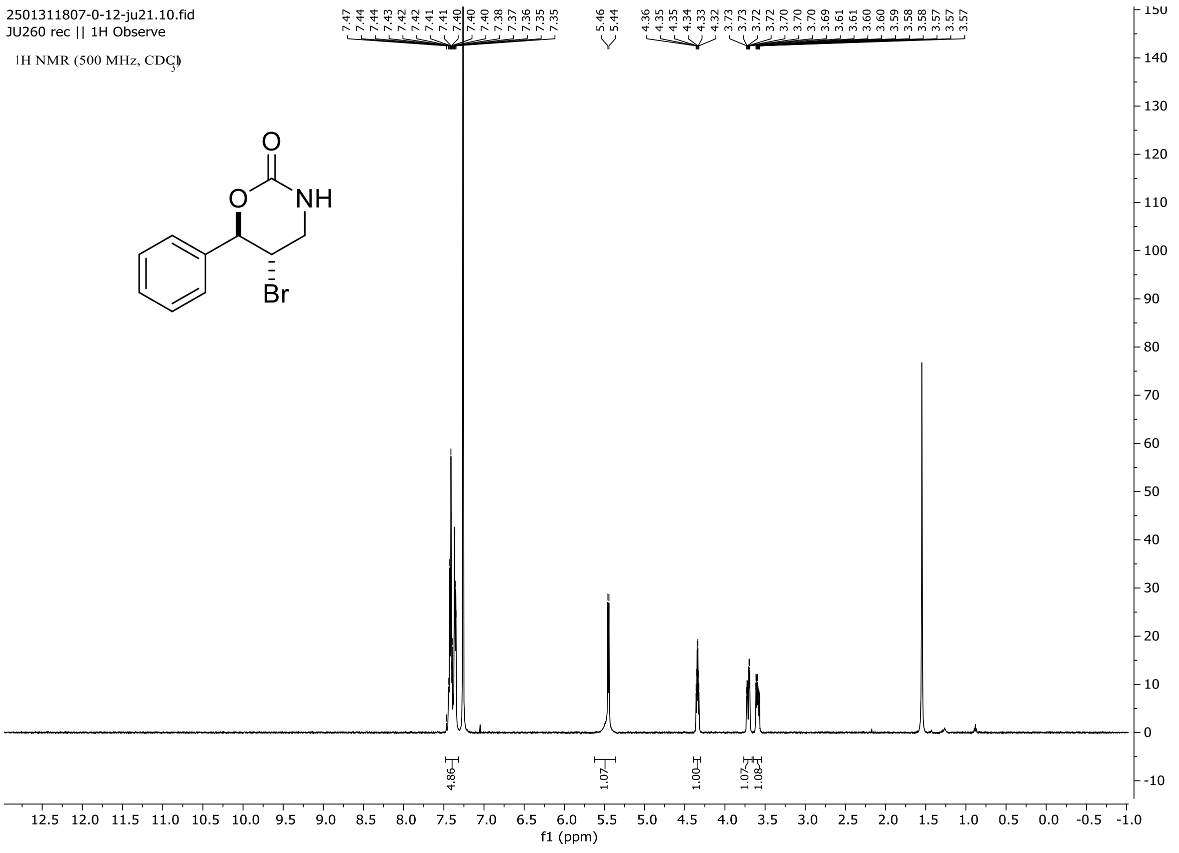

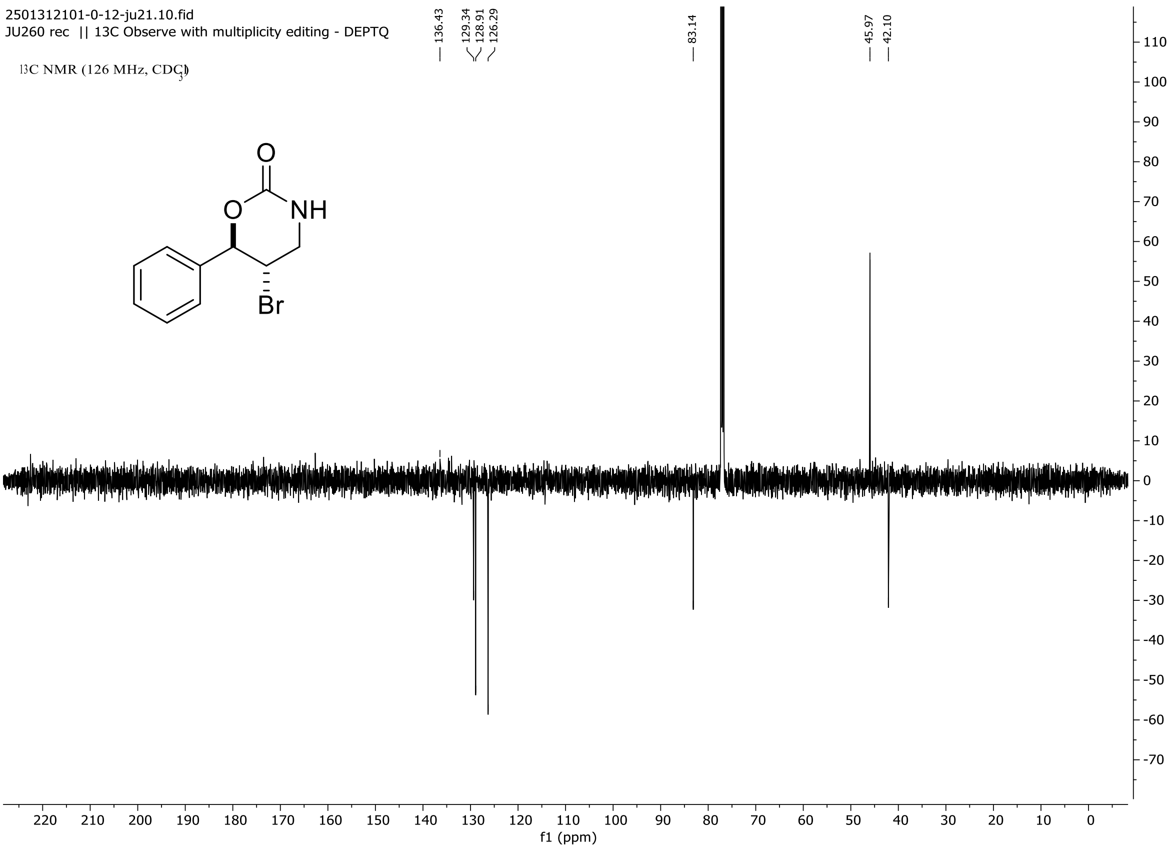

6-(4-Chlorophenyl)-5-iodo-1,3-oxazinan-2-one (29)

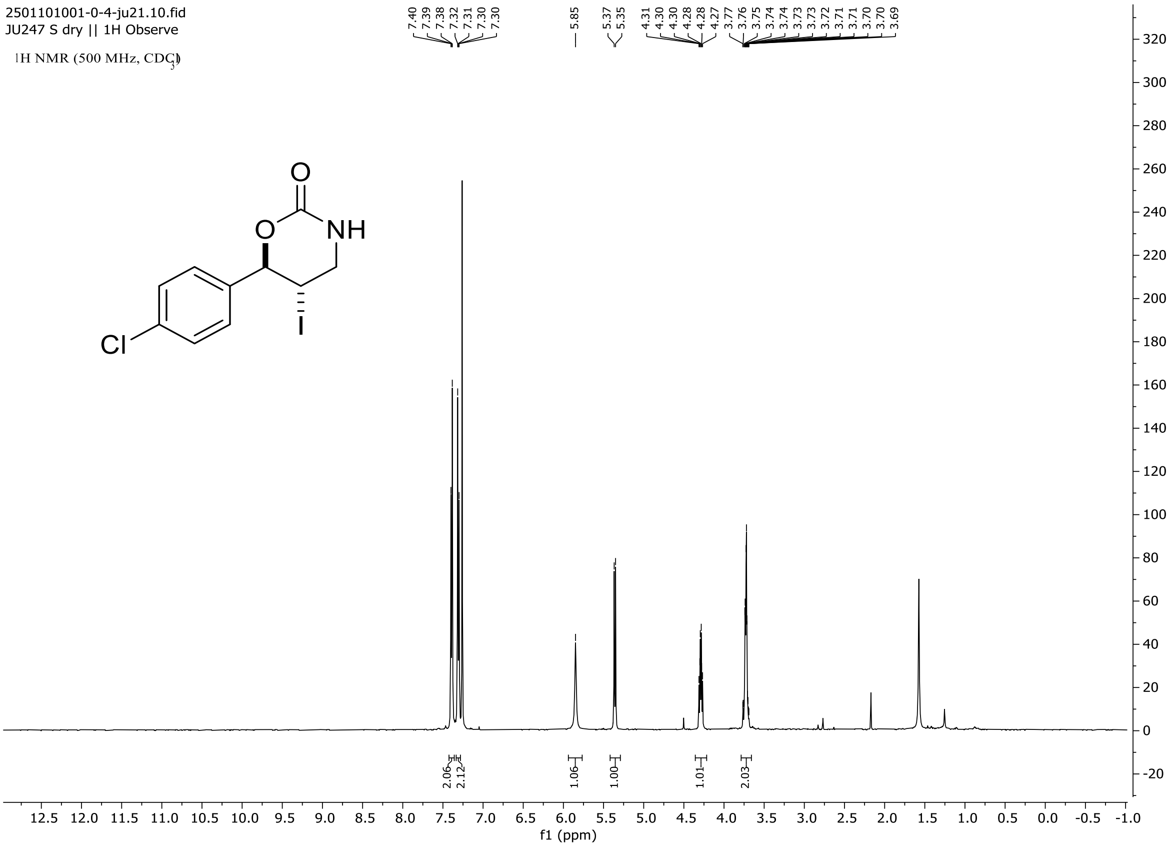

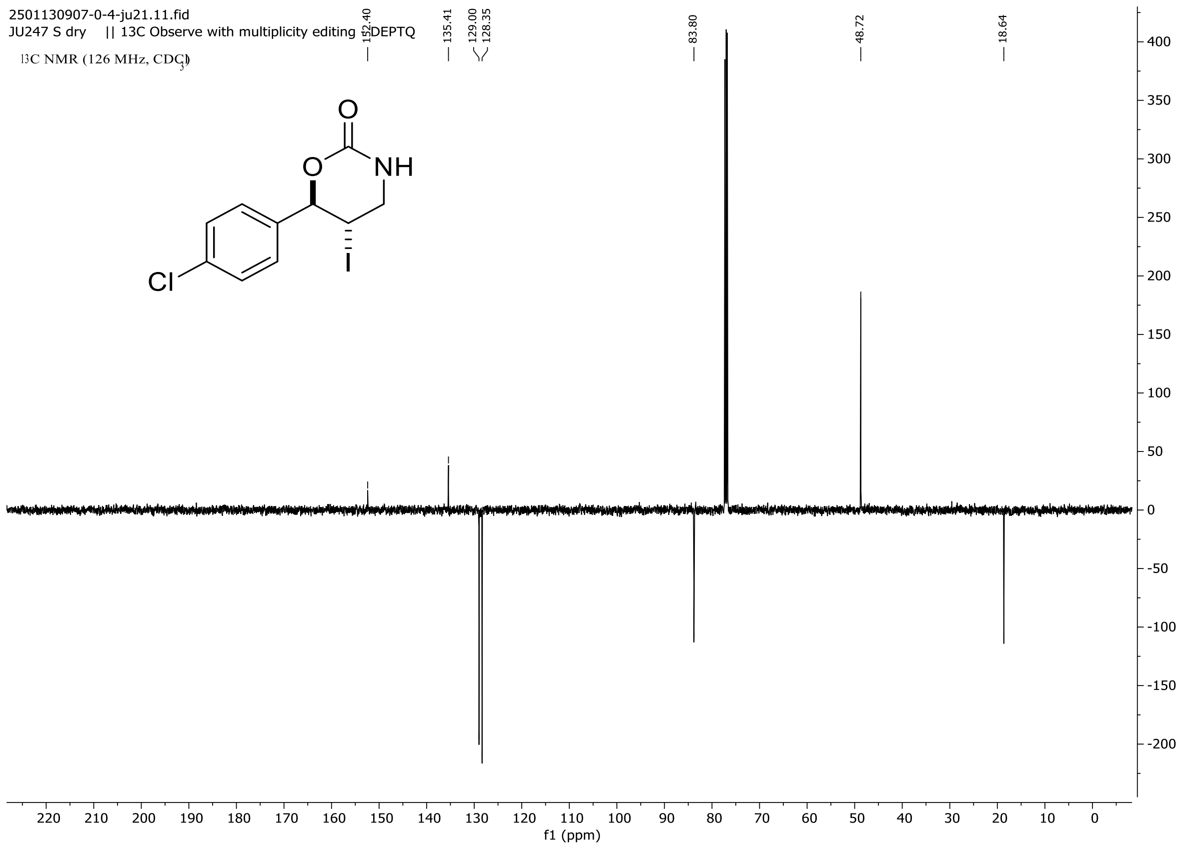

5-Iodo-3-methyl-6-phenyl-1,3-oxazinan-2-one (30)

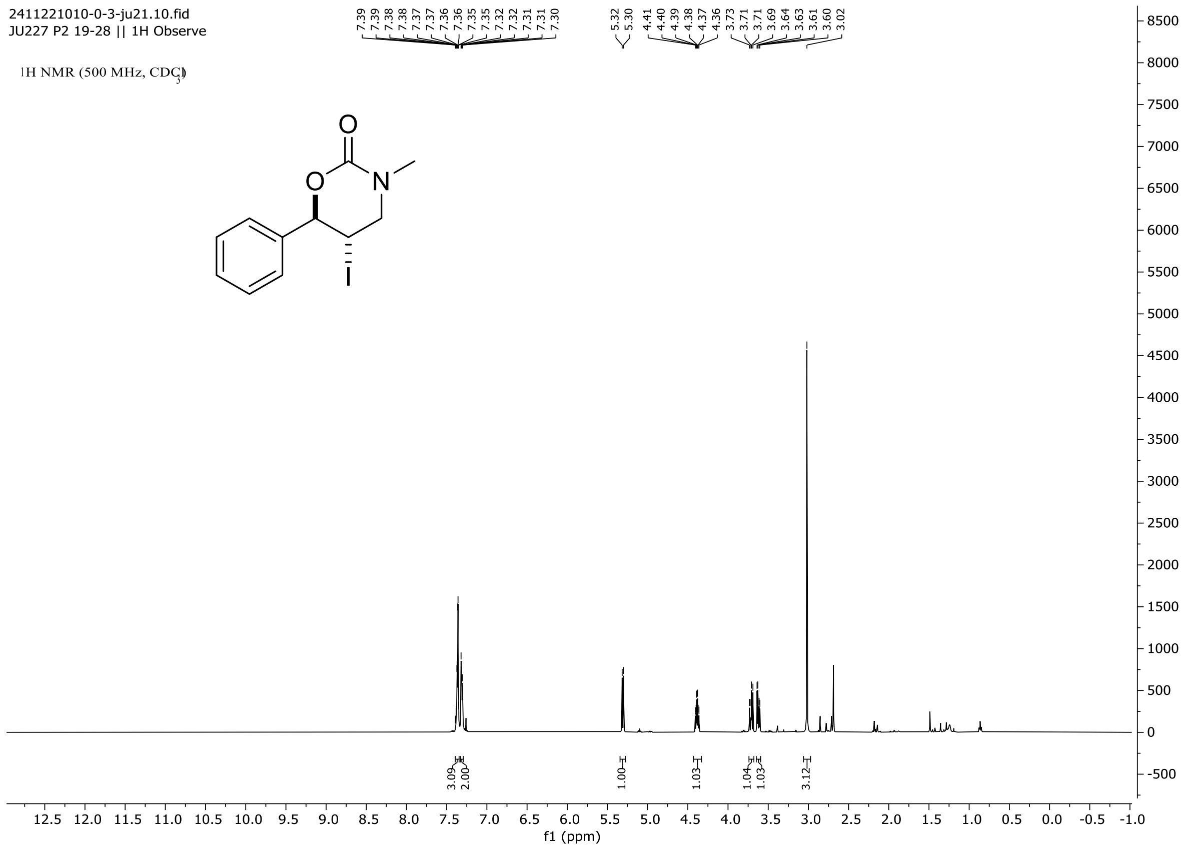

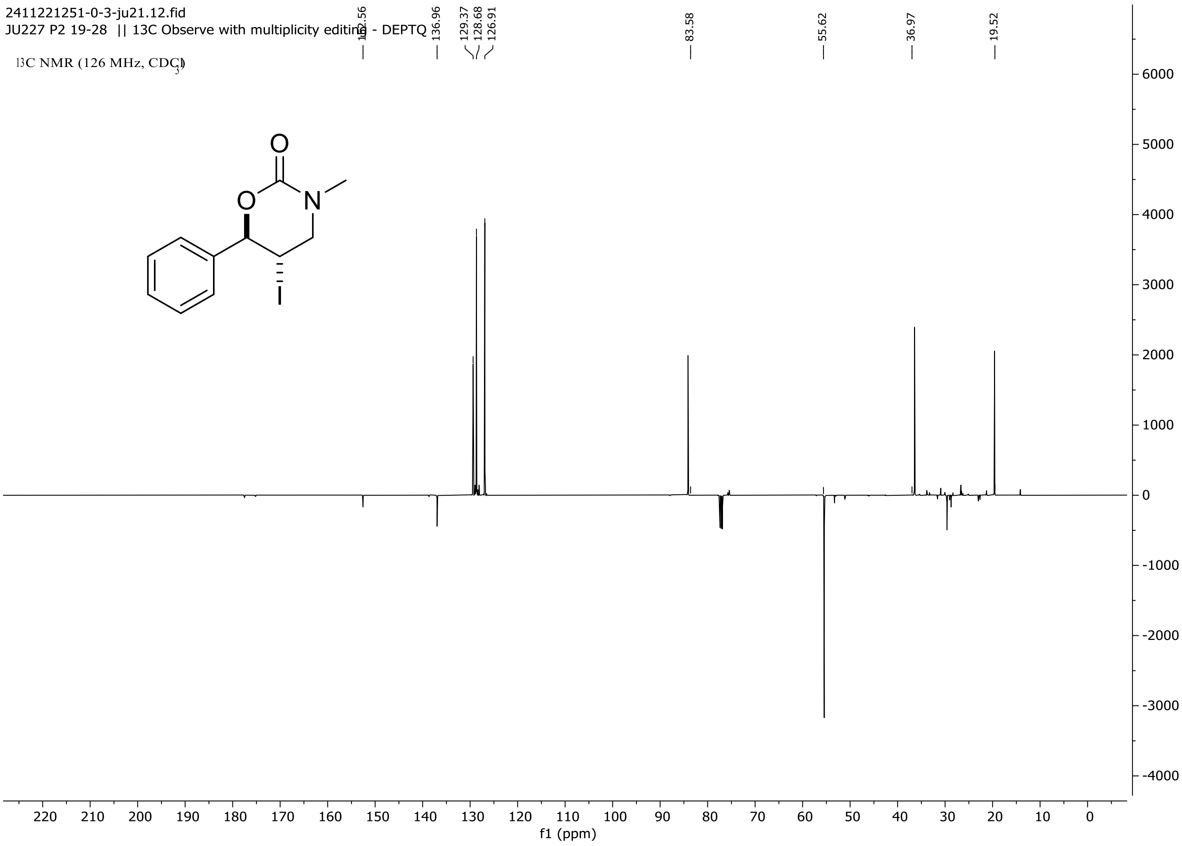

1-(2-(Bromo(phenyl)methyl)aziridin-1-yl)ethan-1-one (32)

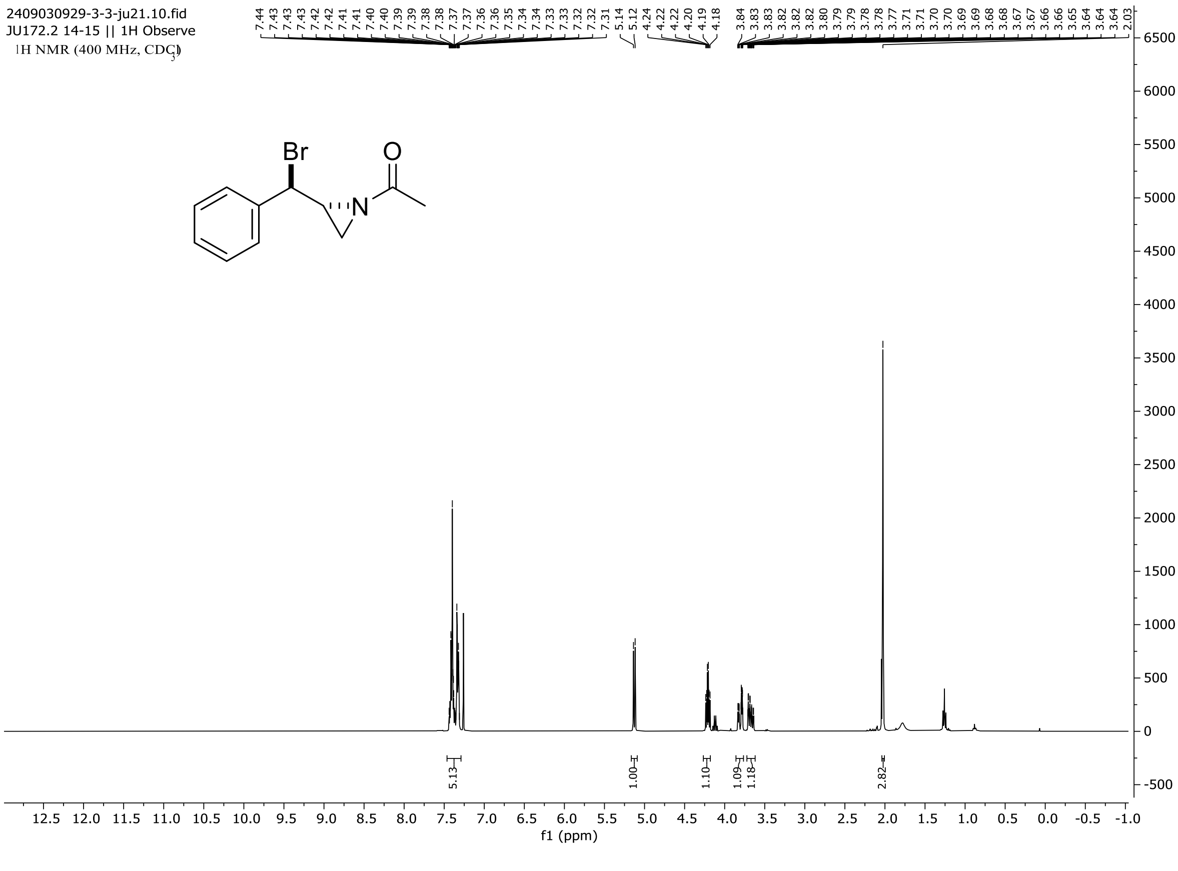

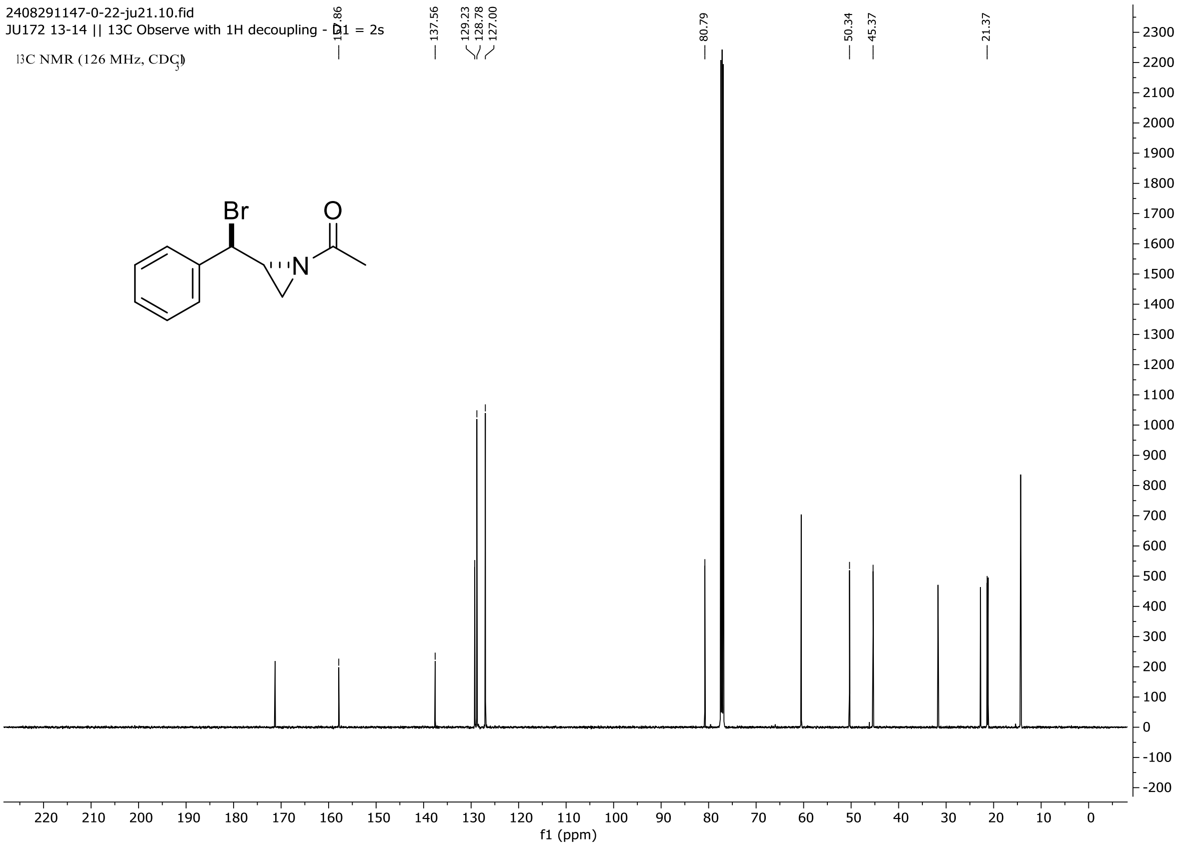

1-(2-(Iodo(phenyl)methyl)aziridin-1-yl)etha-1-one (33)

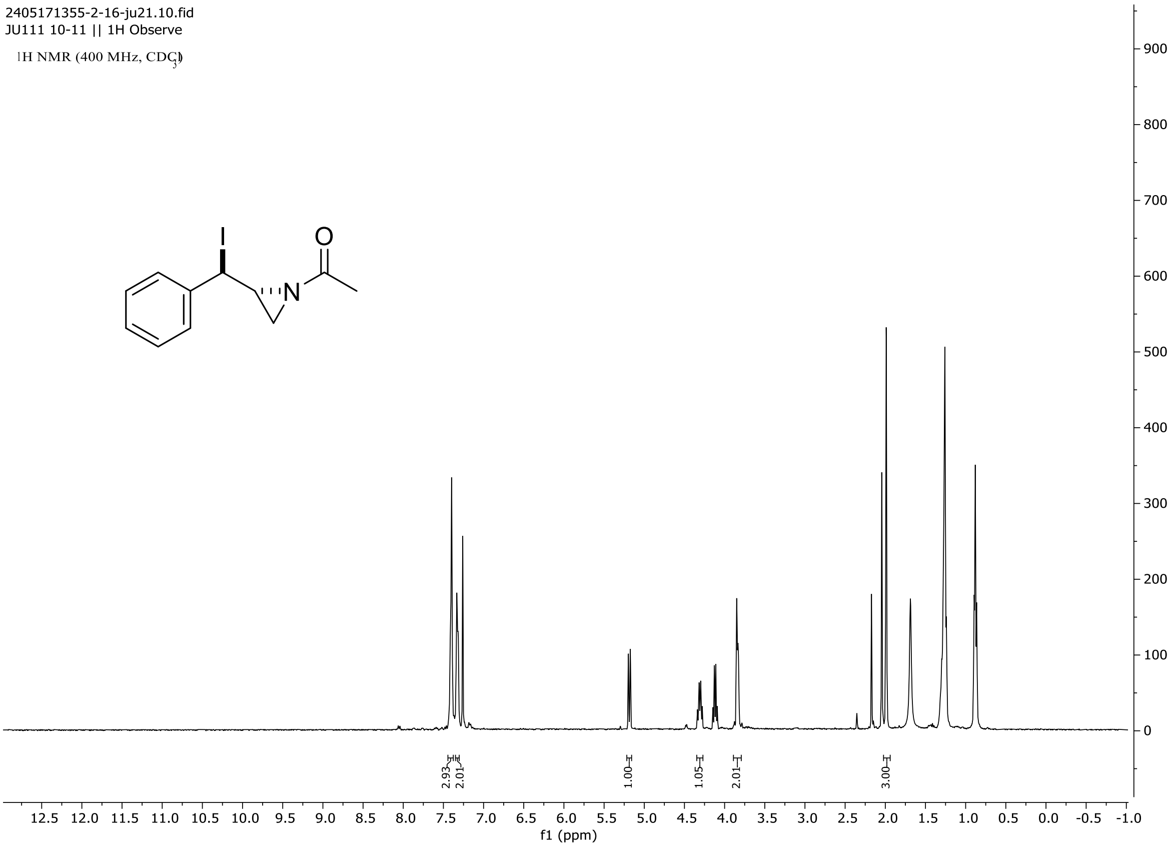

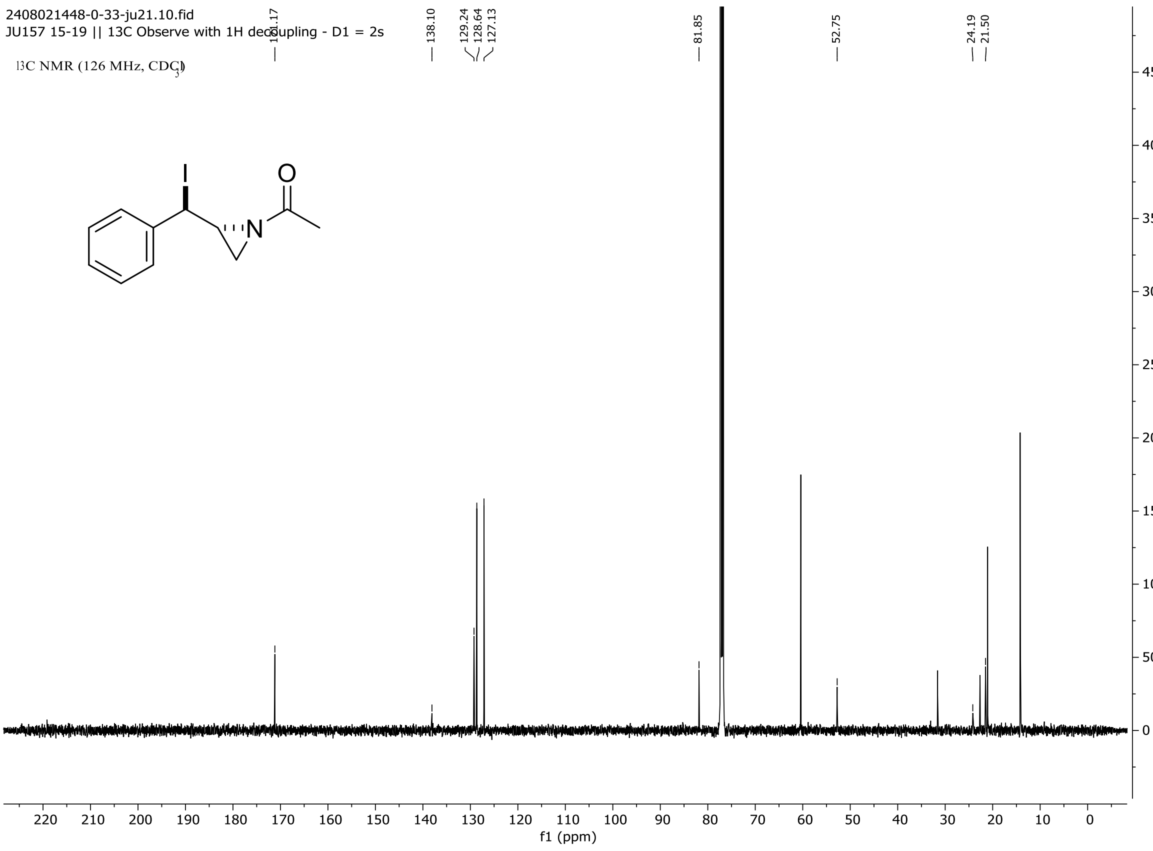

1-(2-Benzyl-3-(bromo(phenyl)methyl)aziridine-1-yl)ethan-1-one (34)

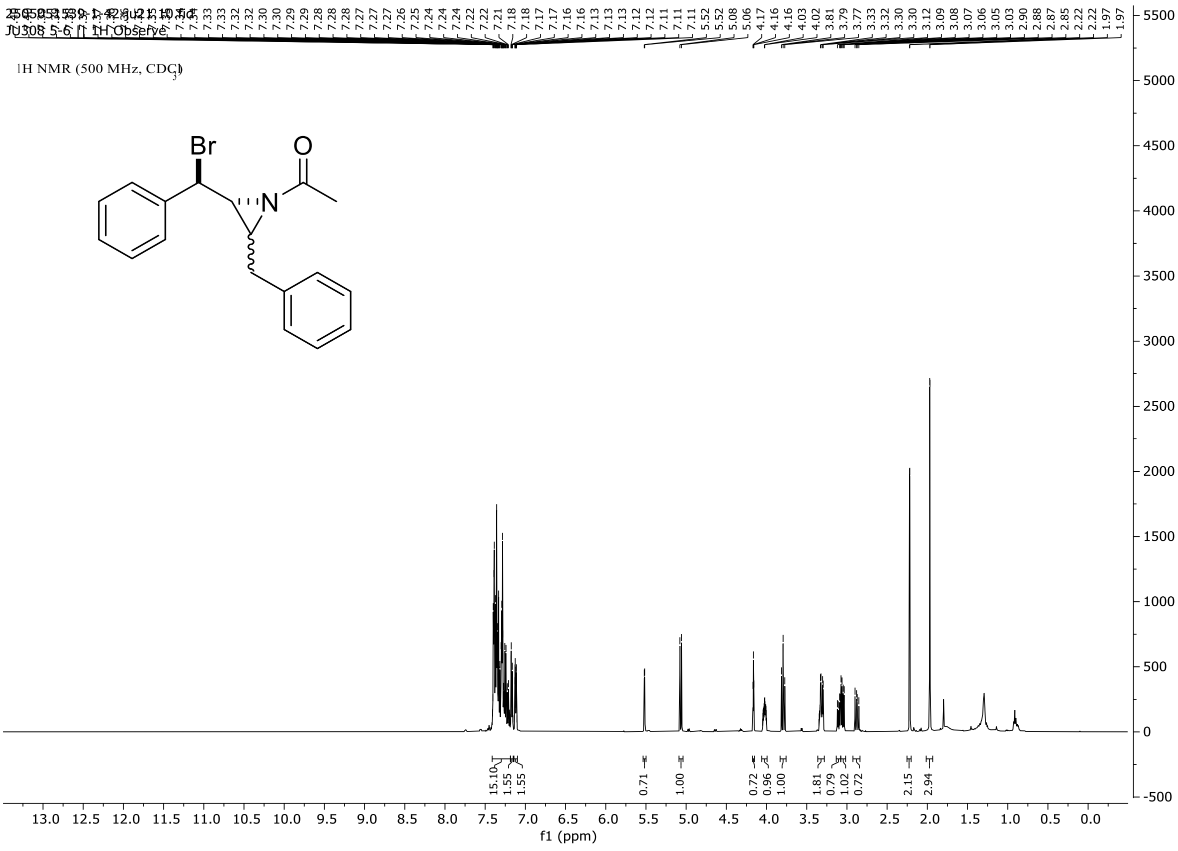

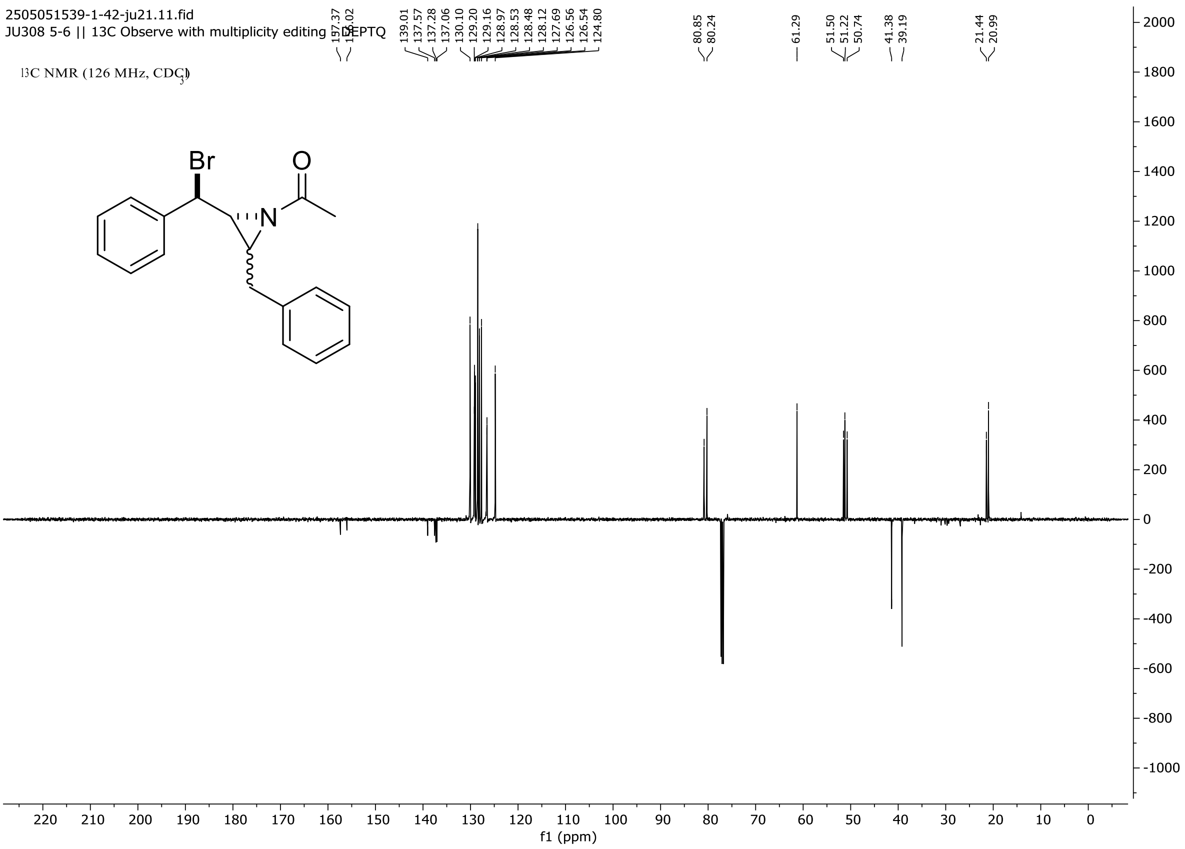

1-(2-(Bromo(4-chlorophenyl)methyl)aziridine-1-yl)ethan-1-one (35)

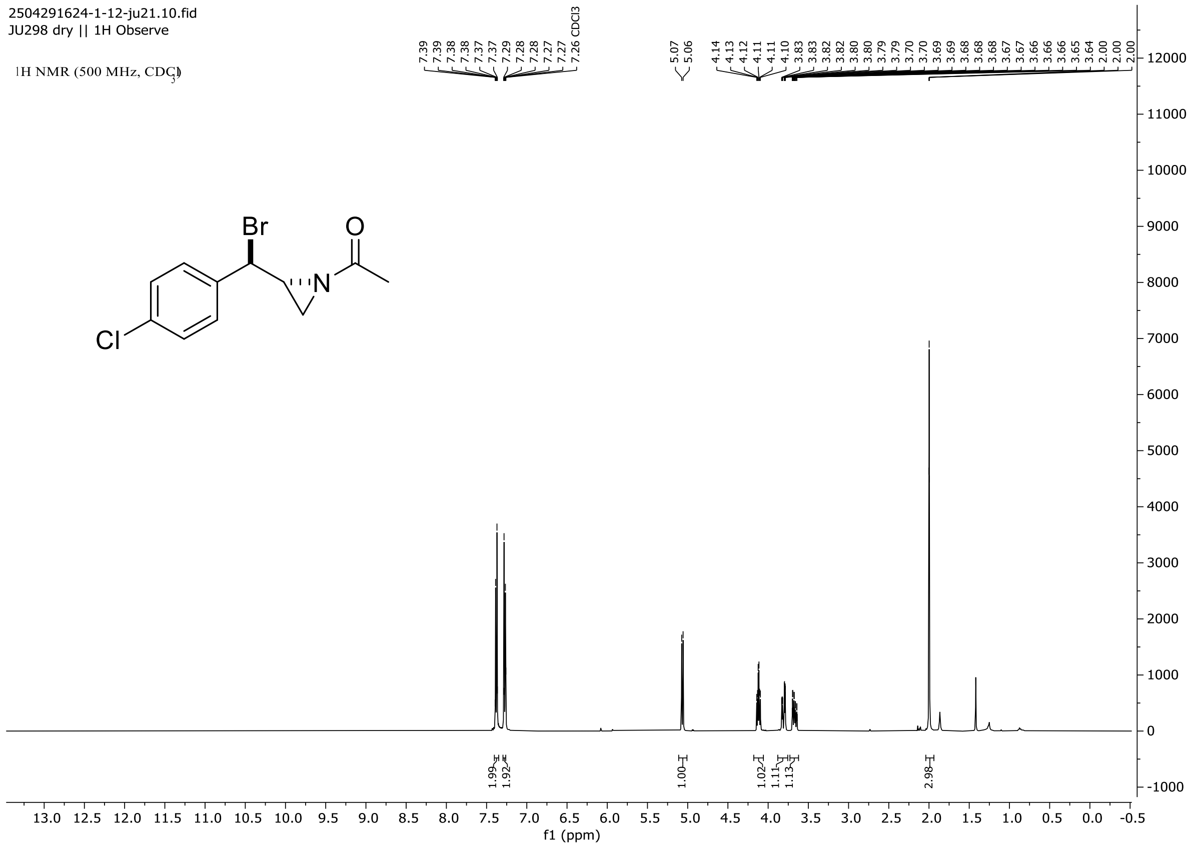

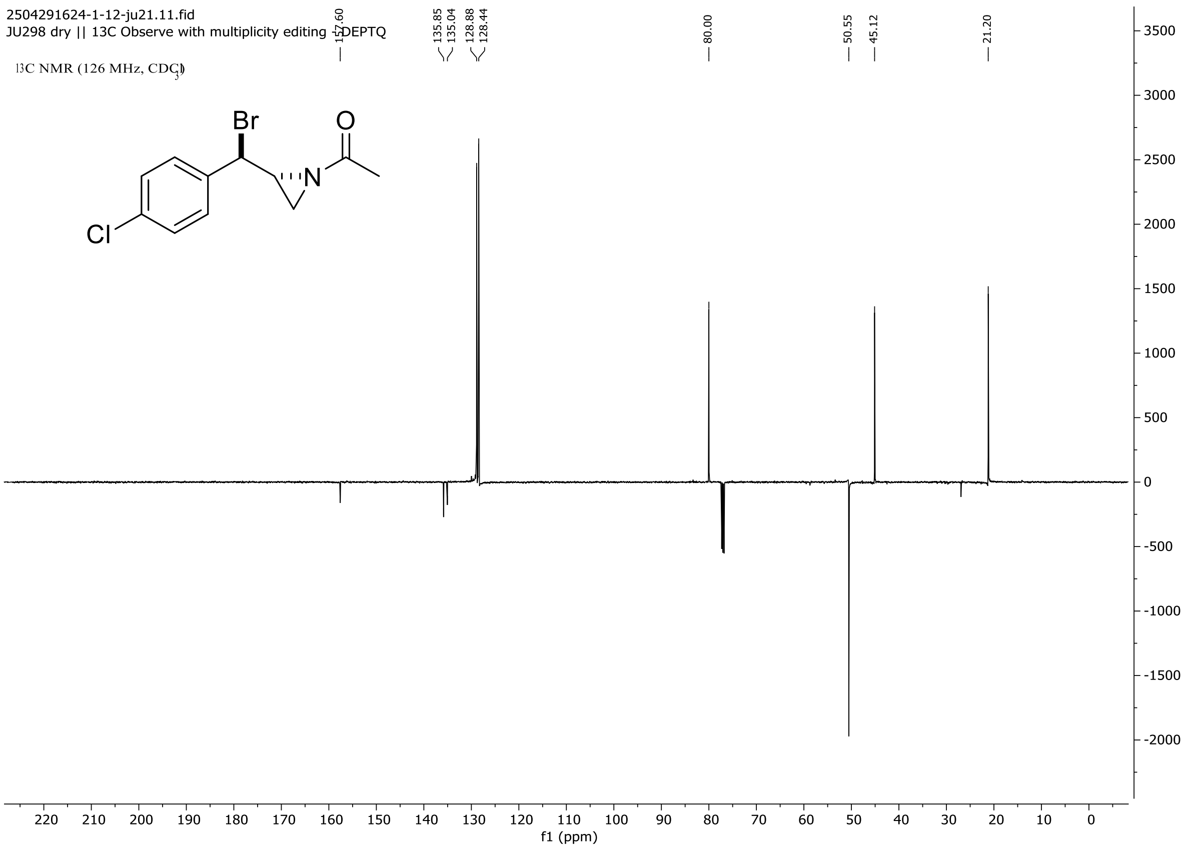

tert-Butyl 3-bromo-2-phenylpyrrolidine-1-carboxylate (36)

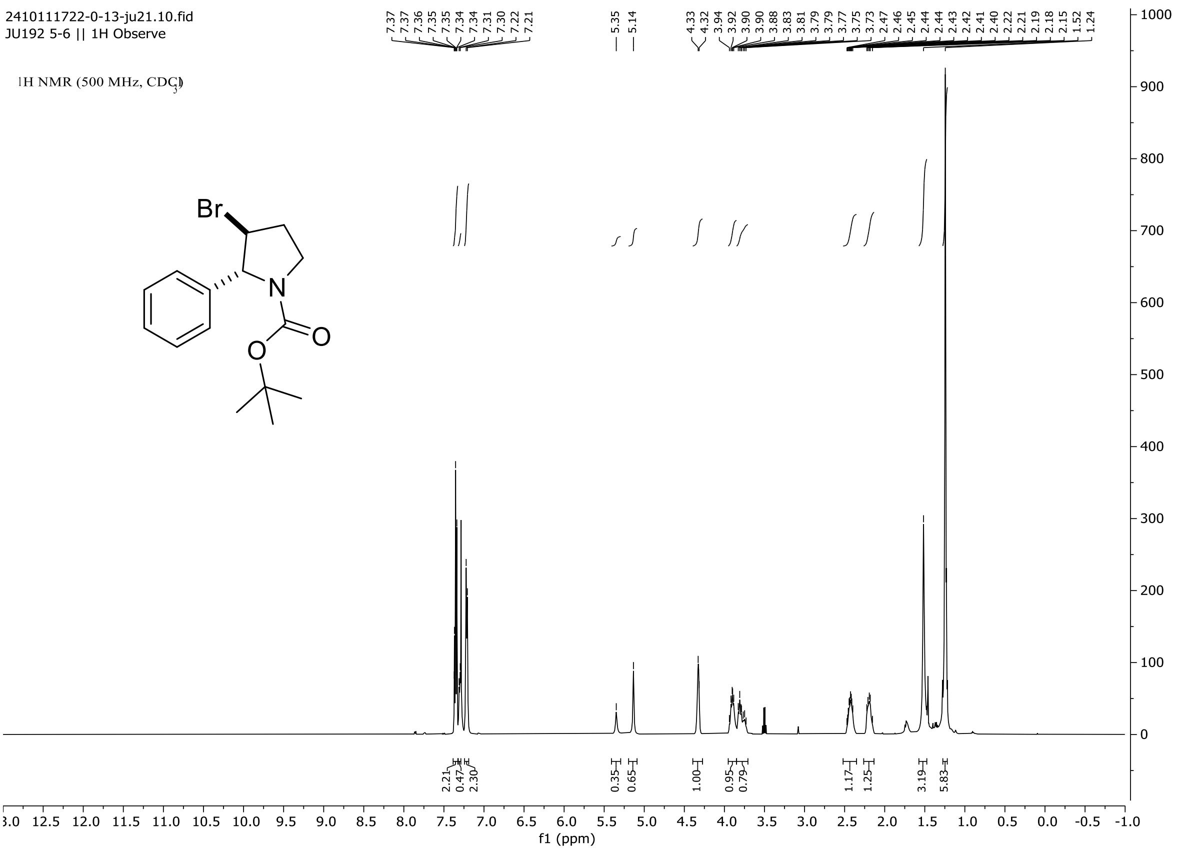

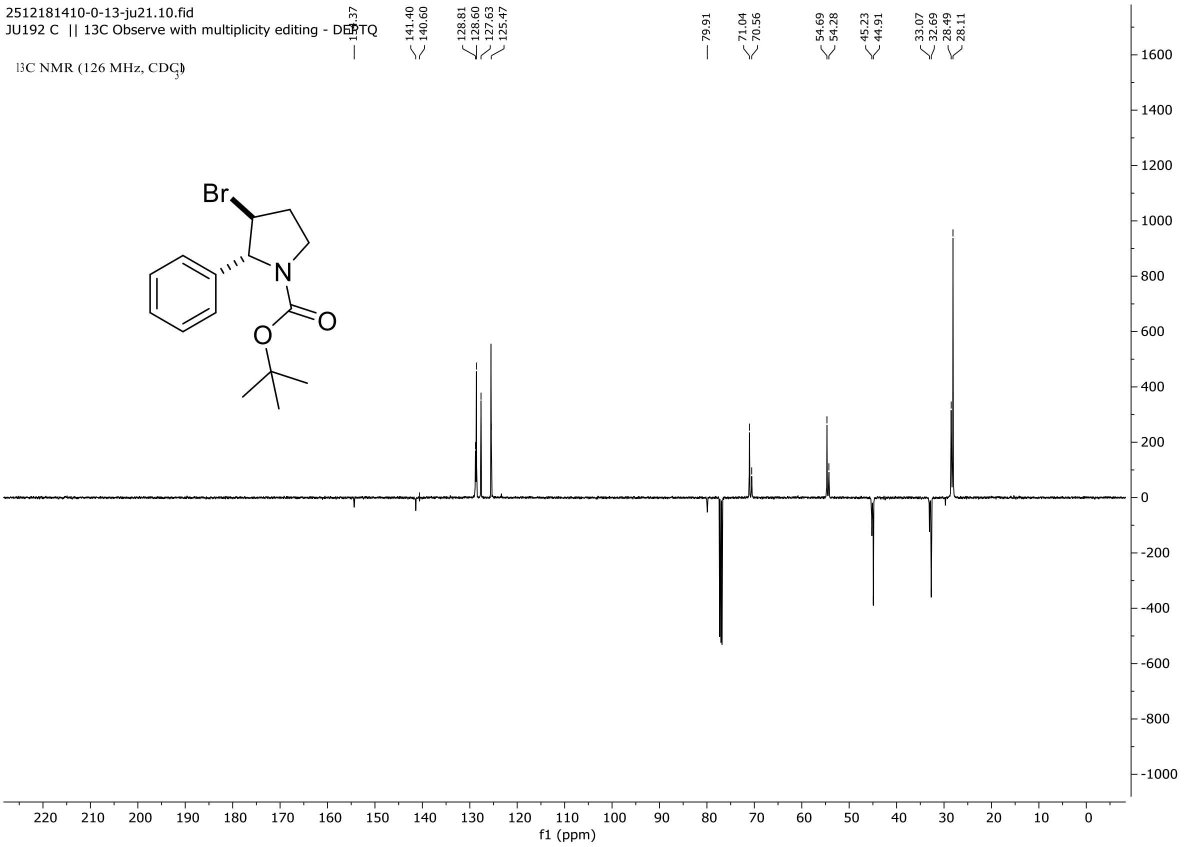

1-(3-Bromo-2-phenylpyrrolidin-1-yl)ethan-1-one (37)

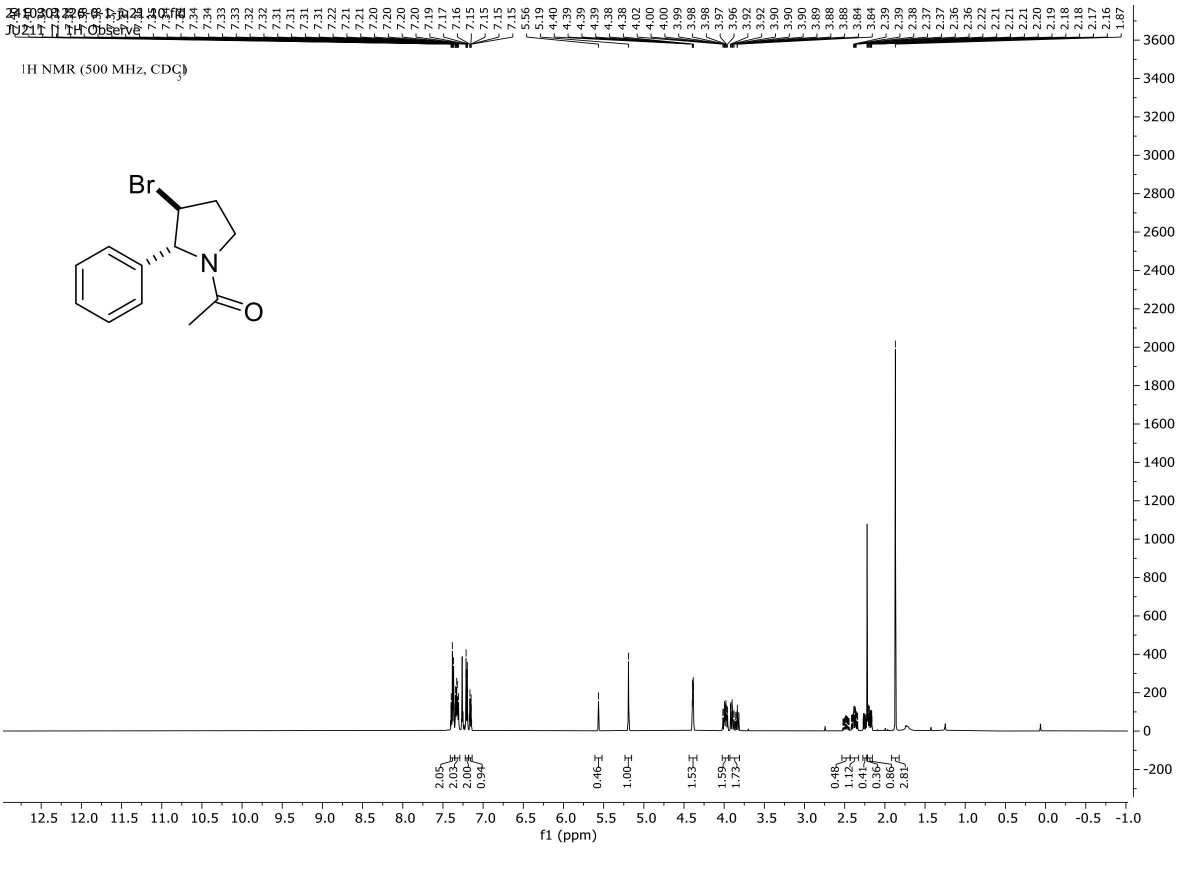

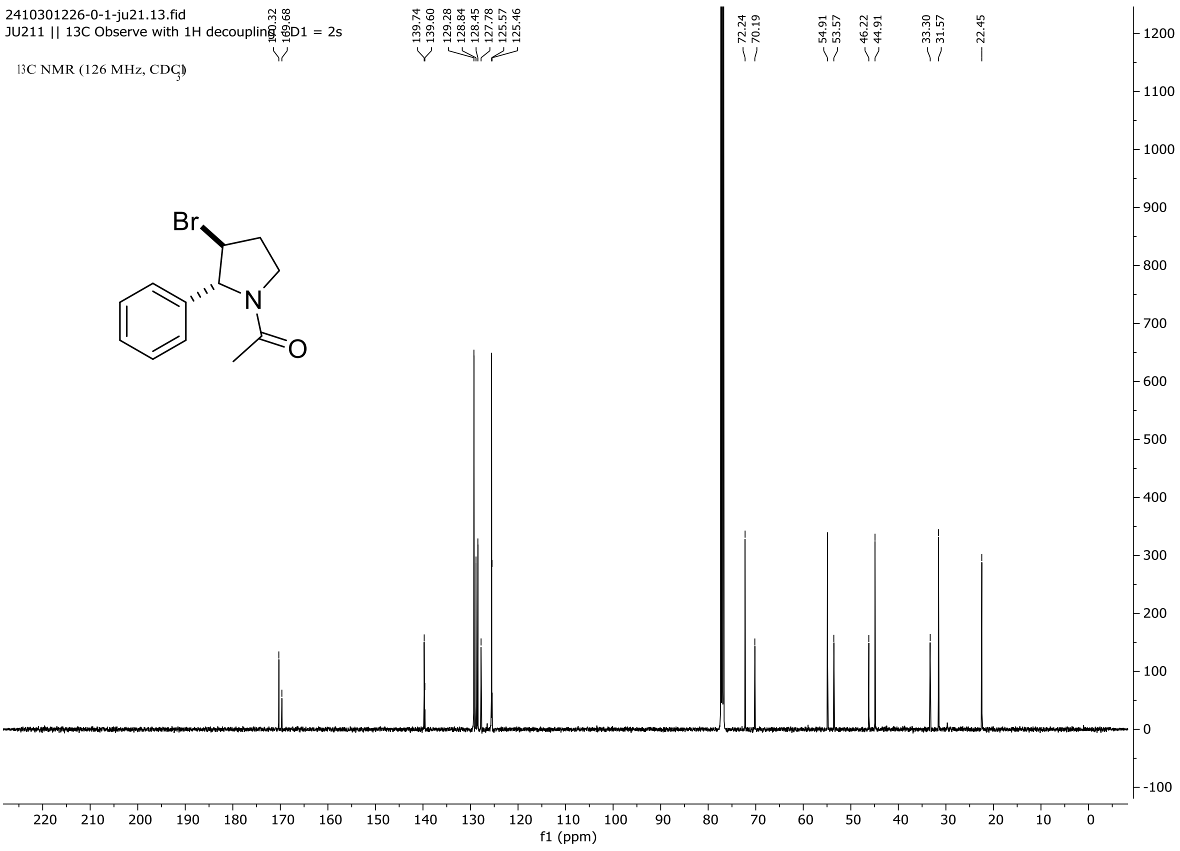

tert-Butyl 3-bromo-5-methyl-2-phenylpyrrolidine-1-carboxylate (38)

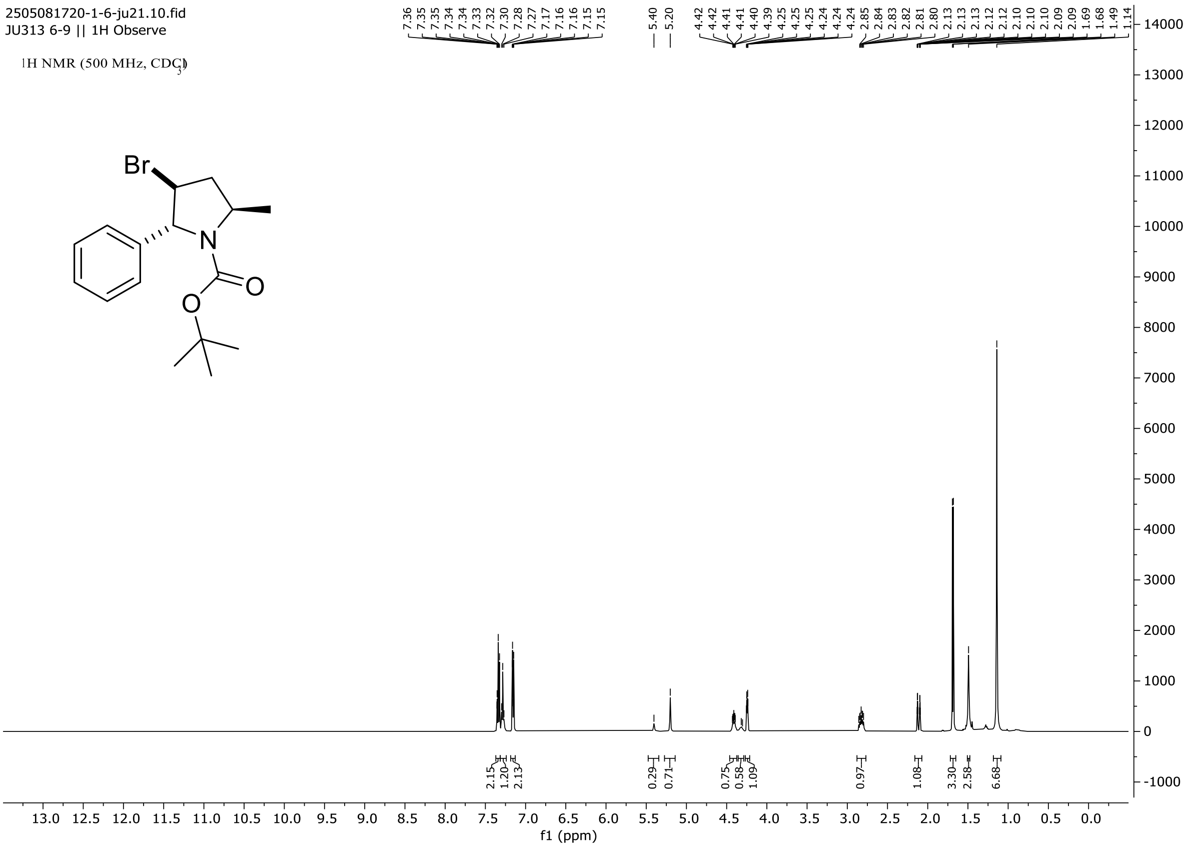

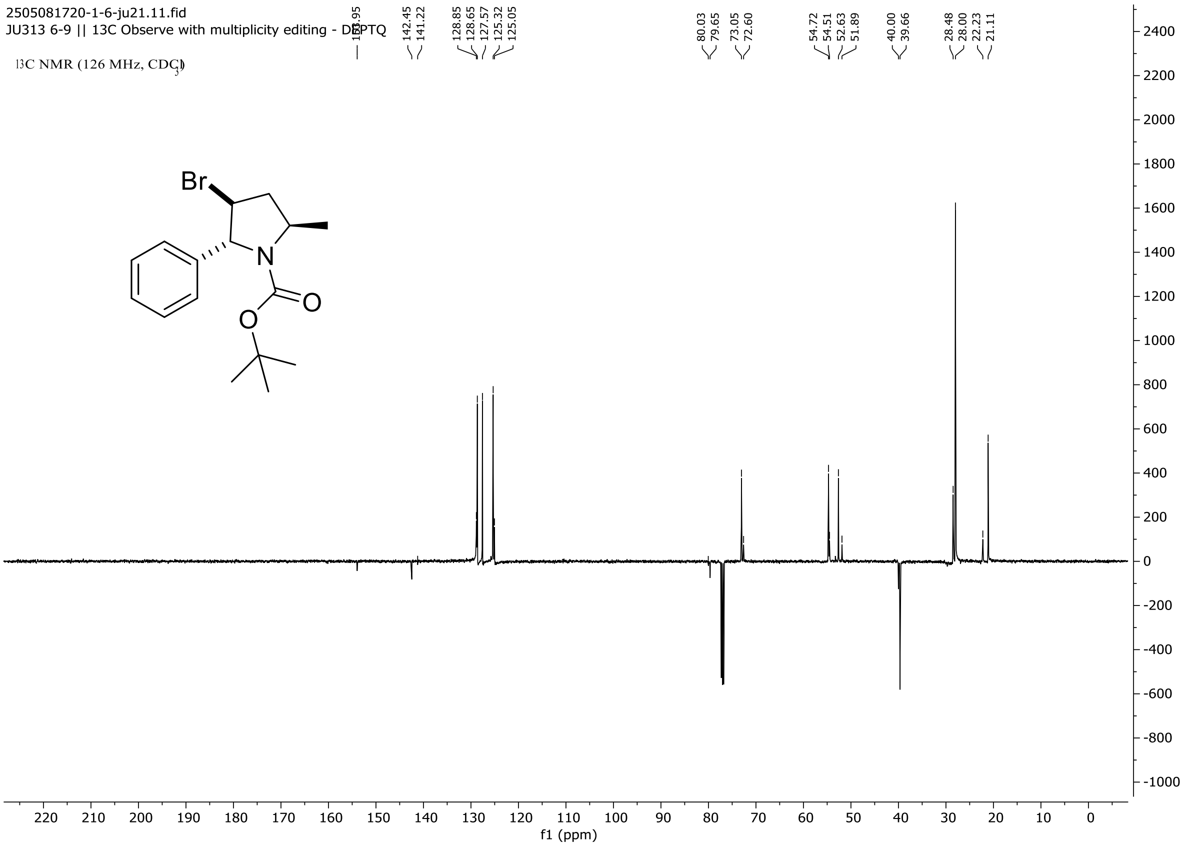

tert-Butyl 3-iodo-5-methyl-2-phenylpyrrolidine-1-carboxylate (39)

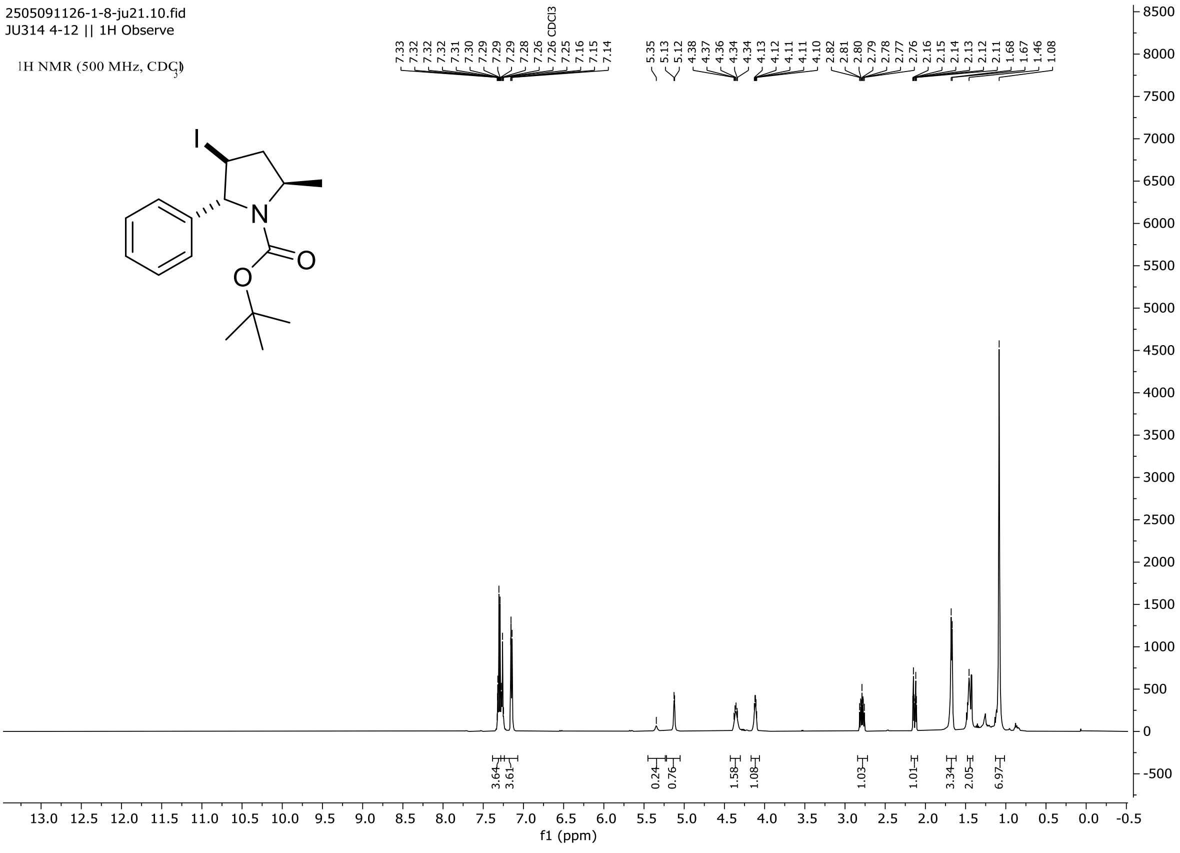

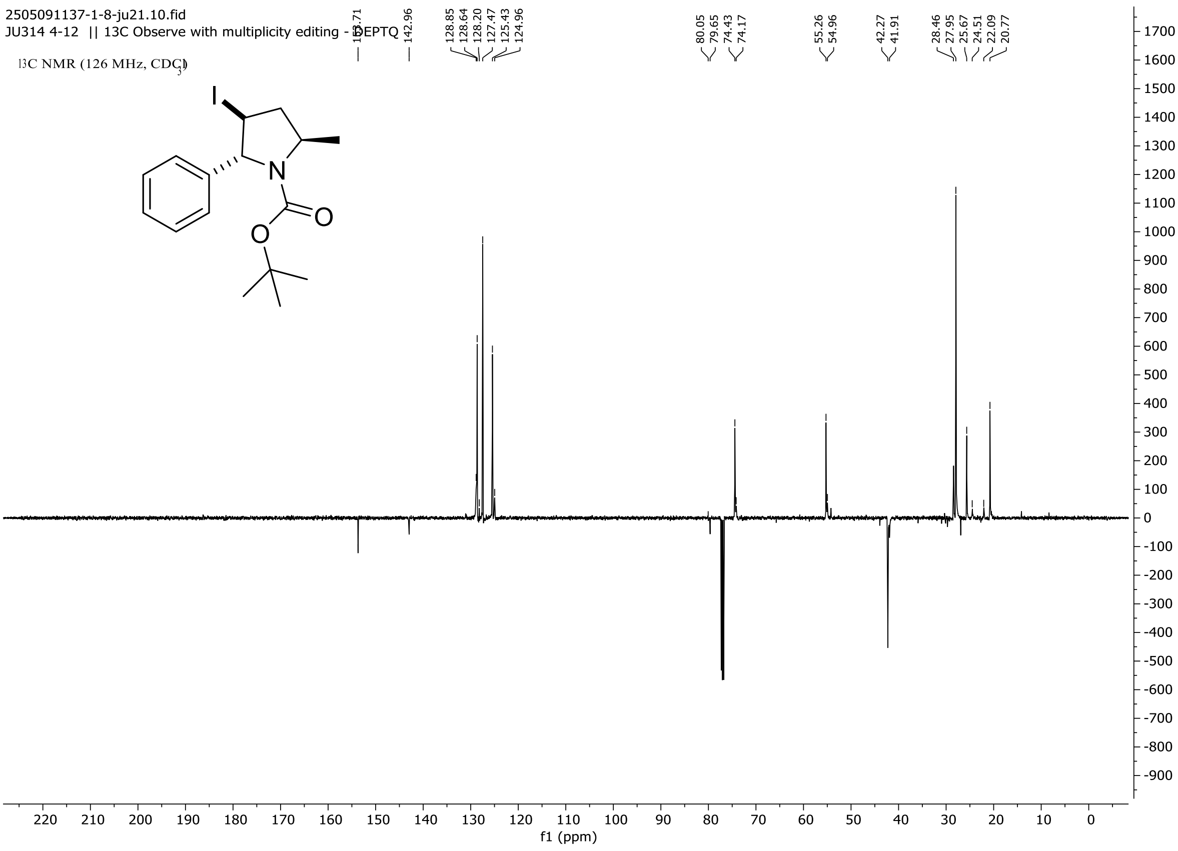

tert-Butyl (5*R*)-3-bromo-2-(4-methoxyphenyl)-5-methylpyrrolidine-1-carboxylate (40)

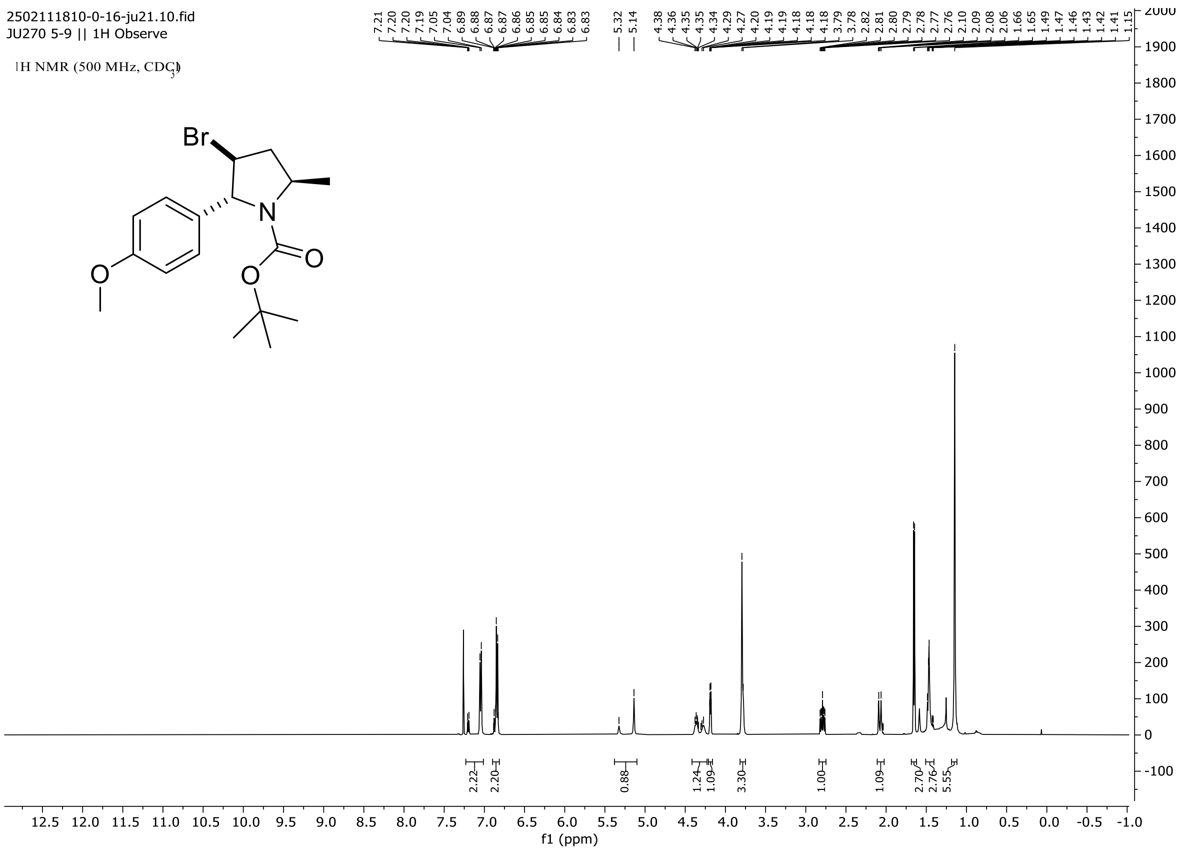

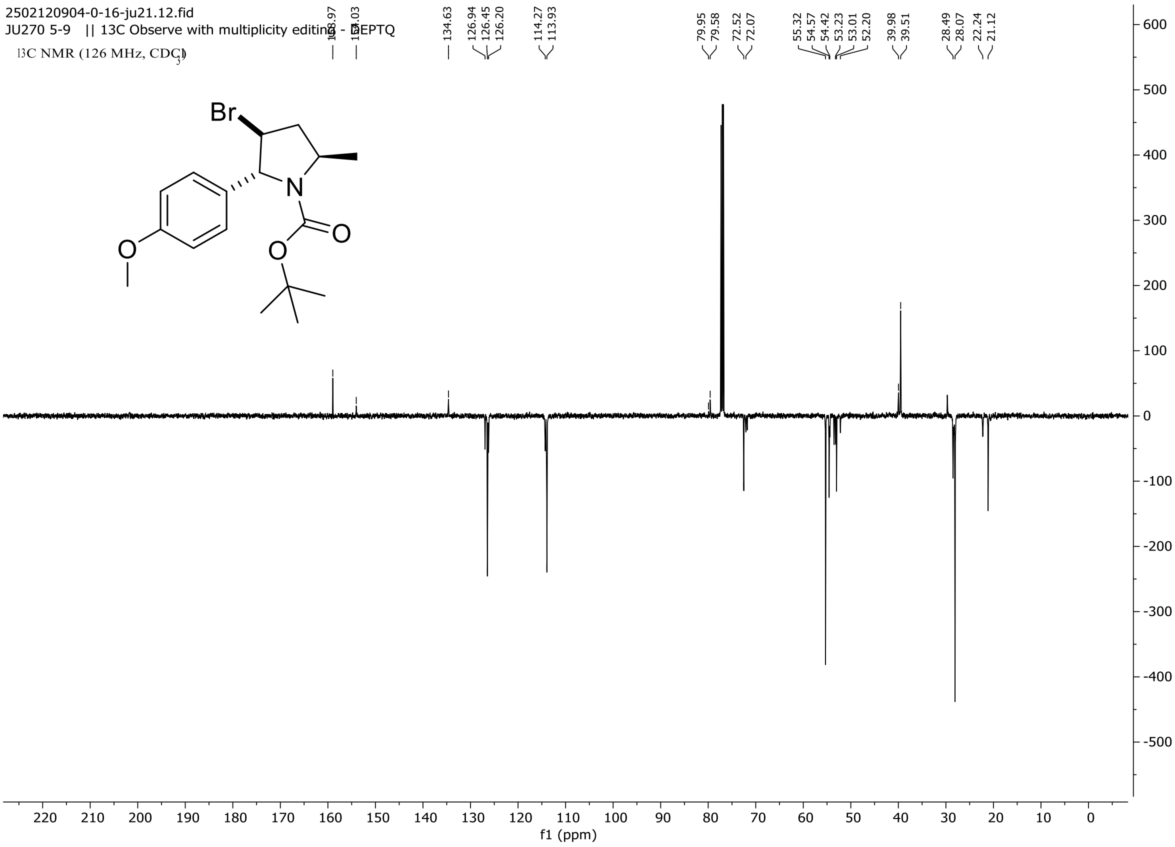

tert-Butyl (5*R*)-3-bromo-2-(2-bromophenyl)-5-methylpyrrolidine-1-carboxylate (41)

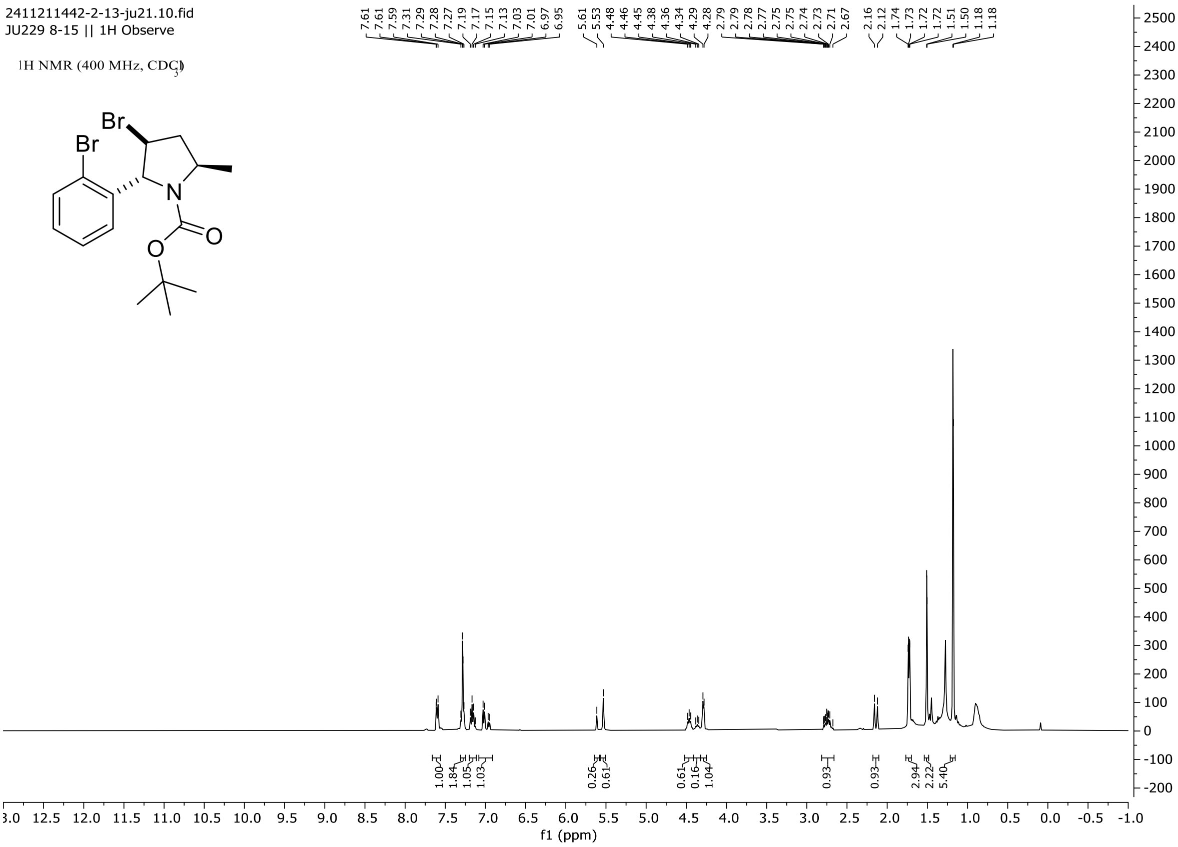

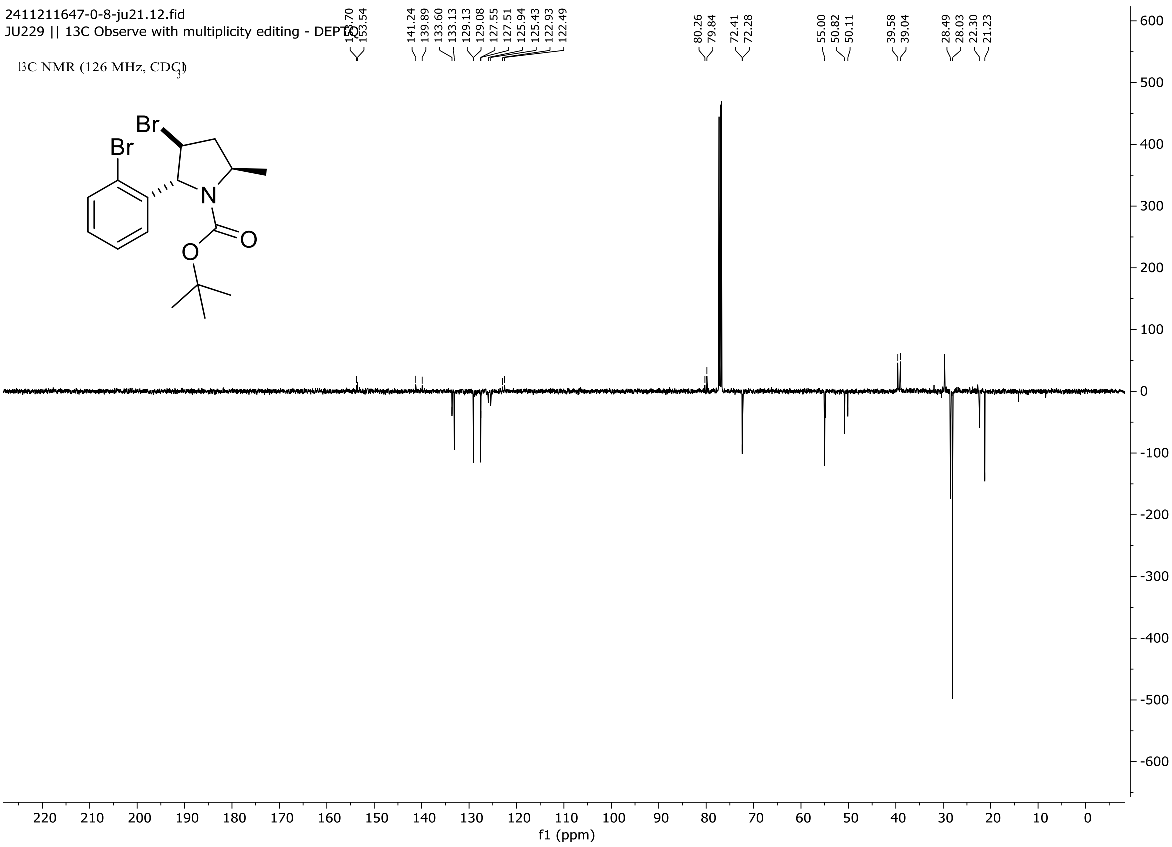

tert-Butyl 3-bromo-2-(4-chlorophenyl)-5-(*p*-tolyl)pyrrolidine-1-carboxylate (42)

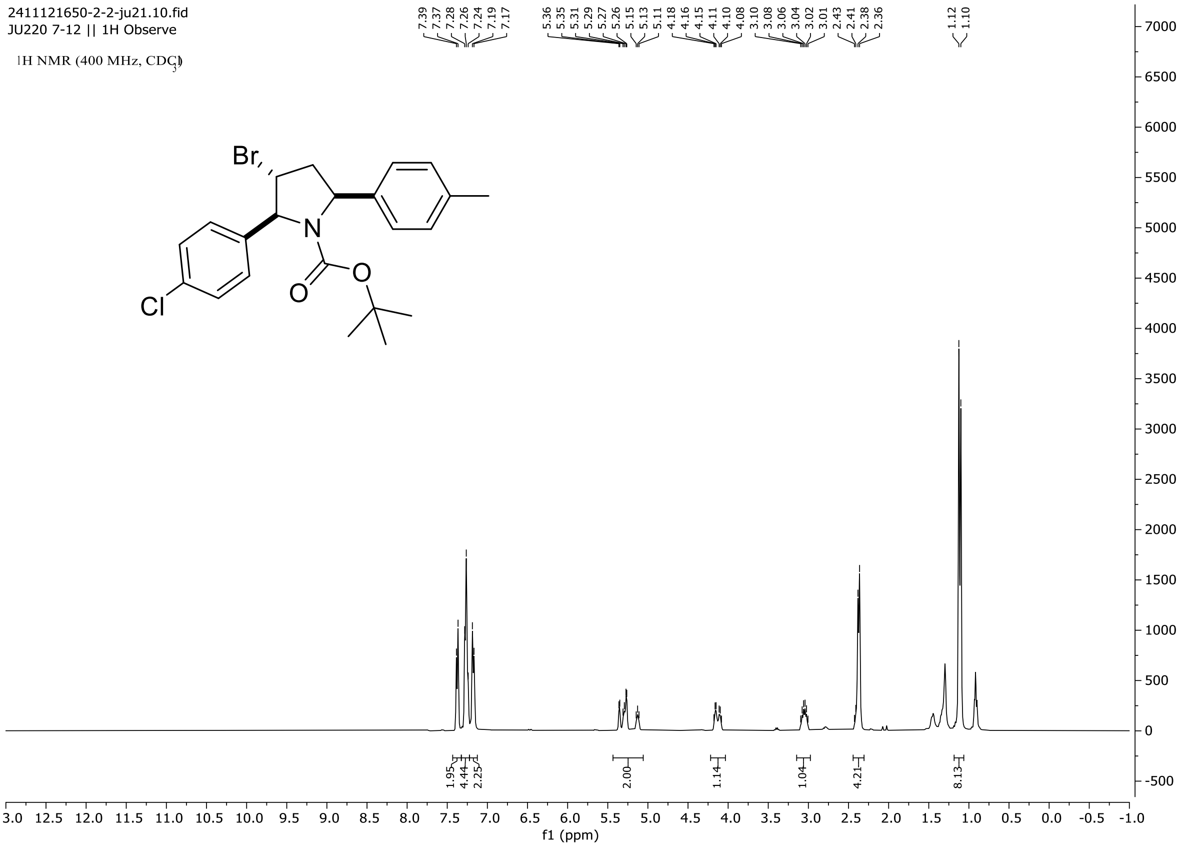

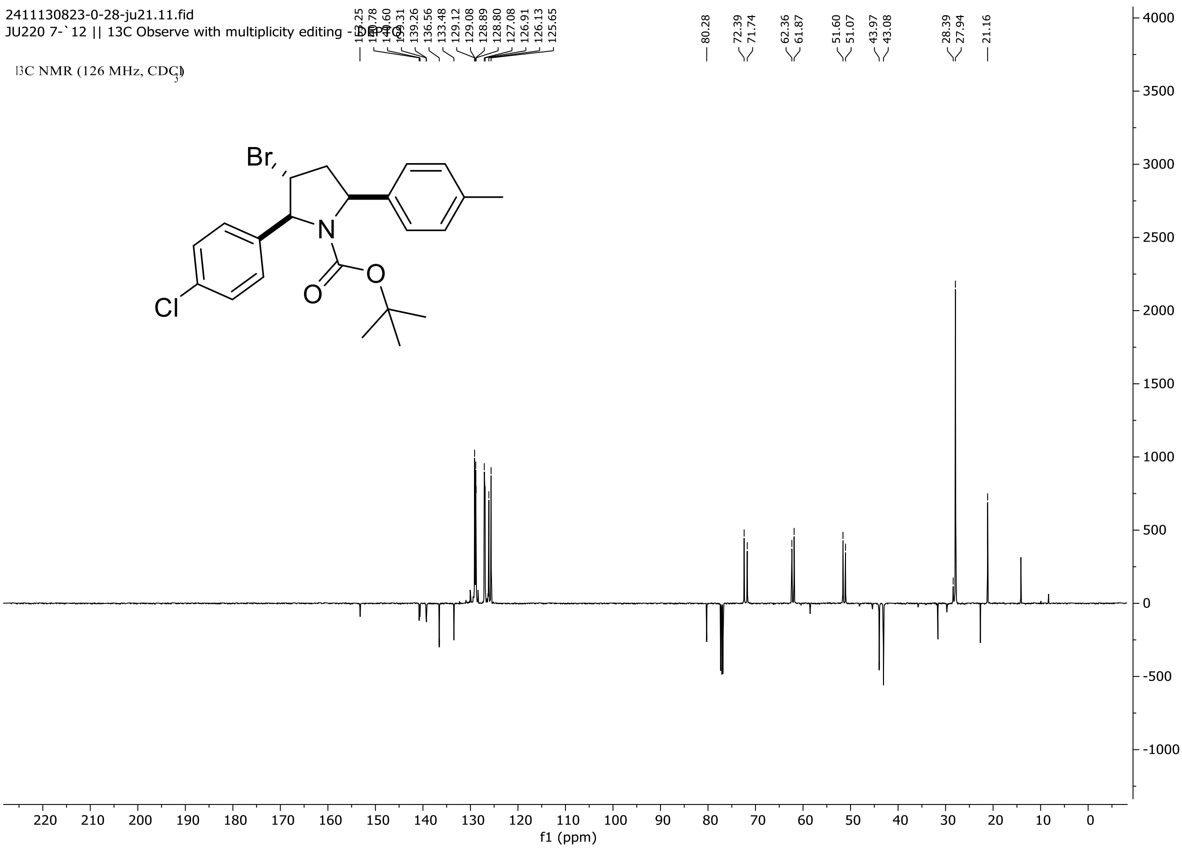

tert-Butyl 3-bromo-2-phenylhexahydrocyclopenta[*b*]pyrrole-1(2*H*)-carboxylate (43)

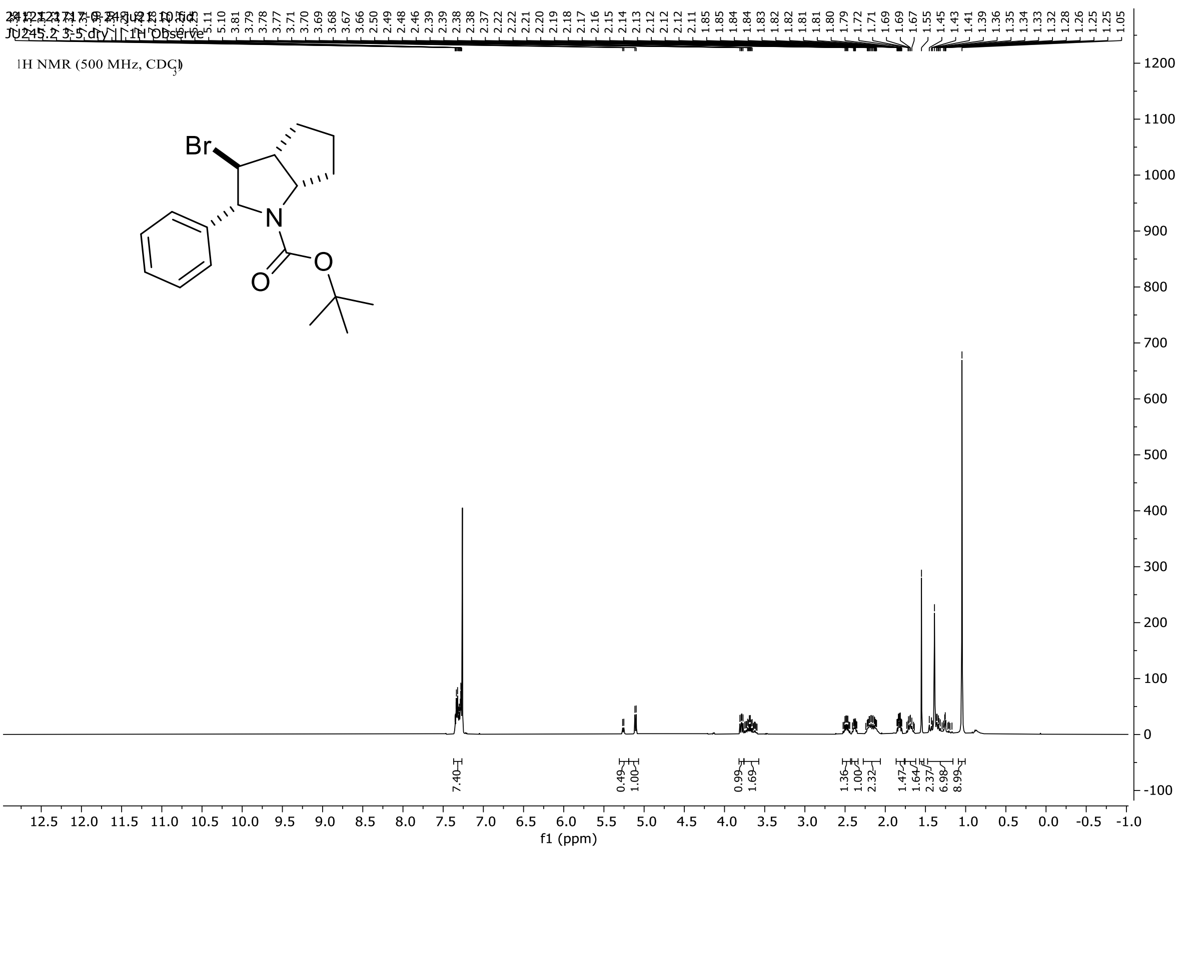

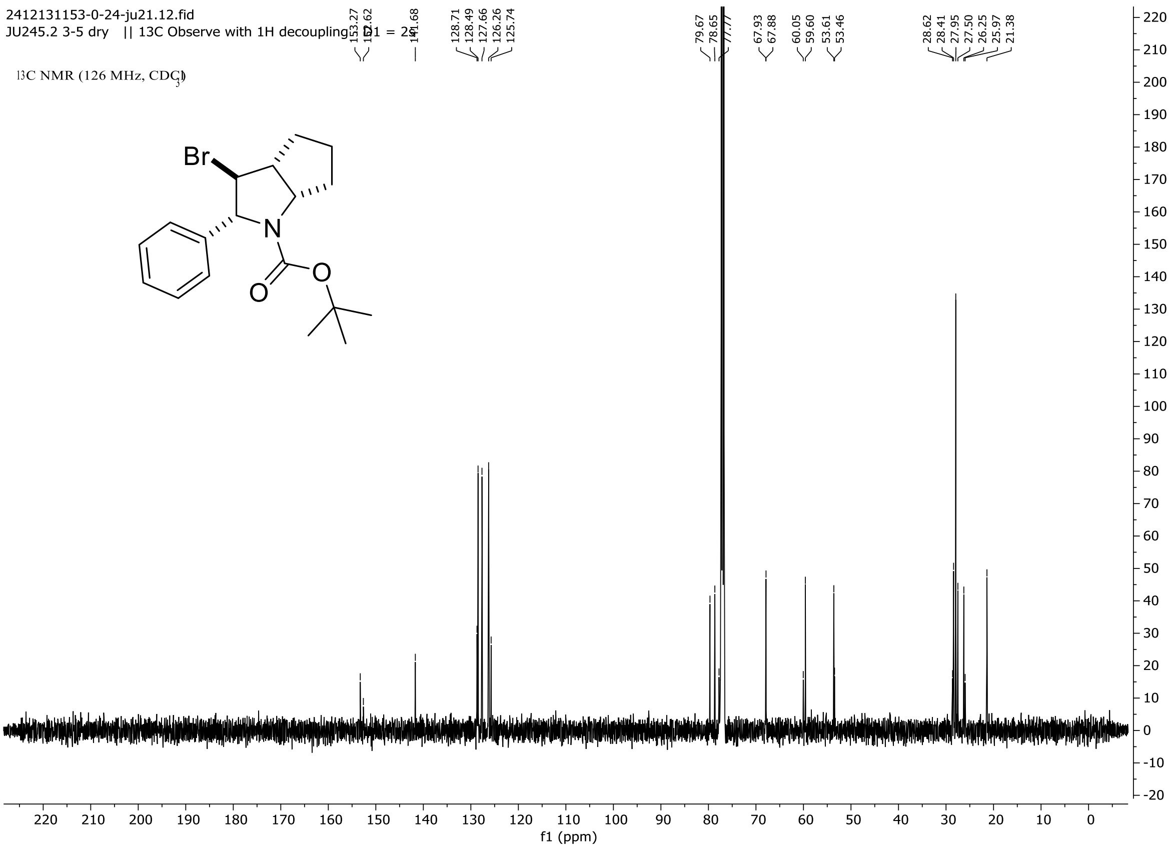

tert-Butyl 3a-bromo-2-methyl-2,3,3a,4,5,9b-hexahydro-1H-benzo[g]indole-1-carboxylate (44)

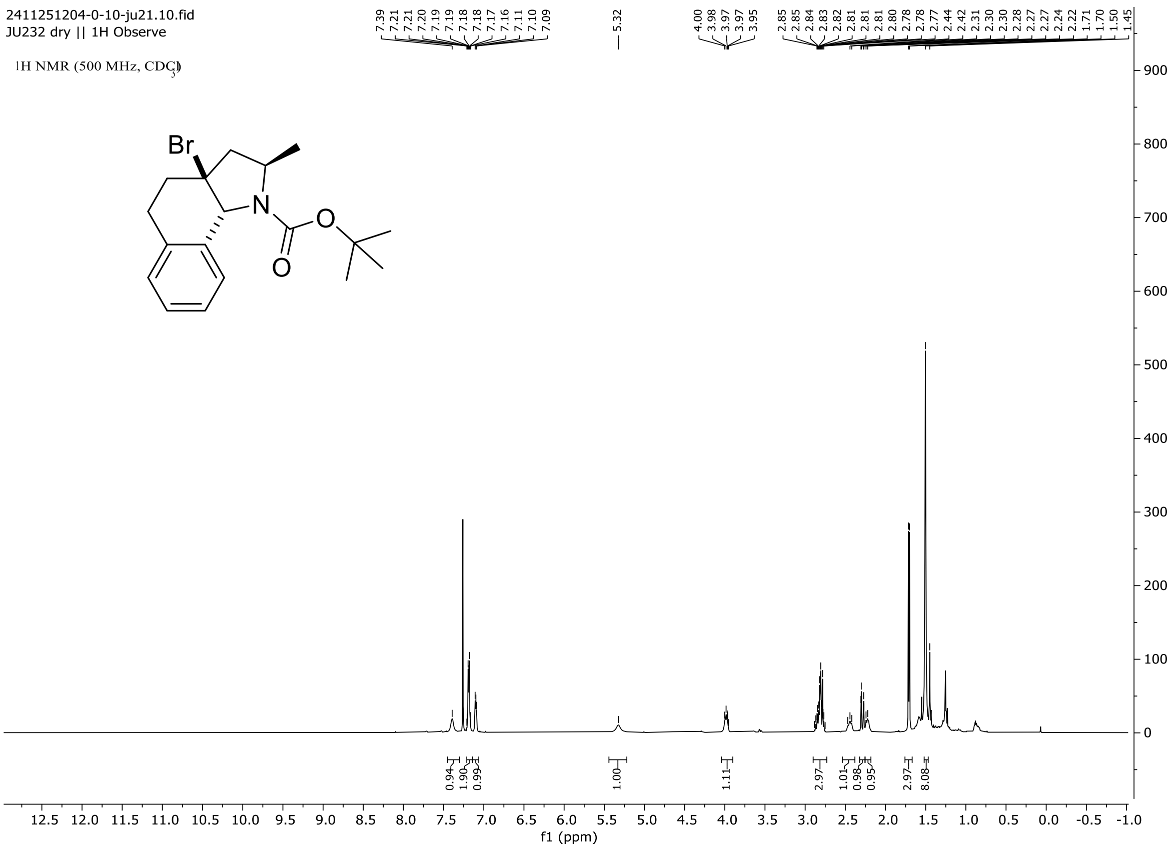

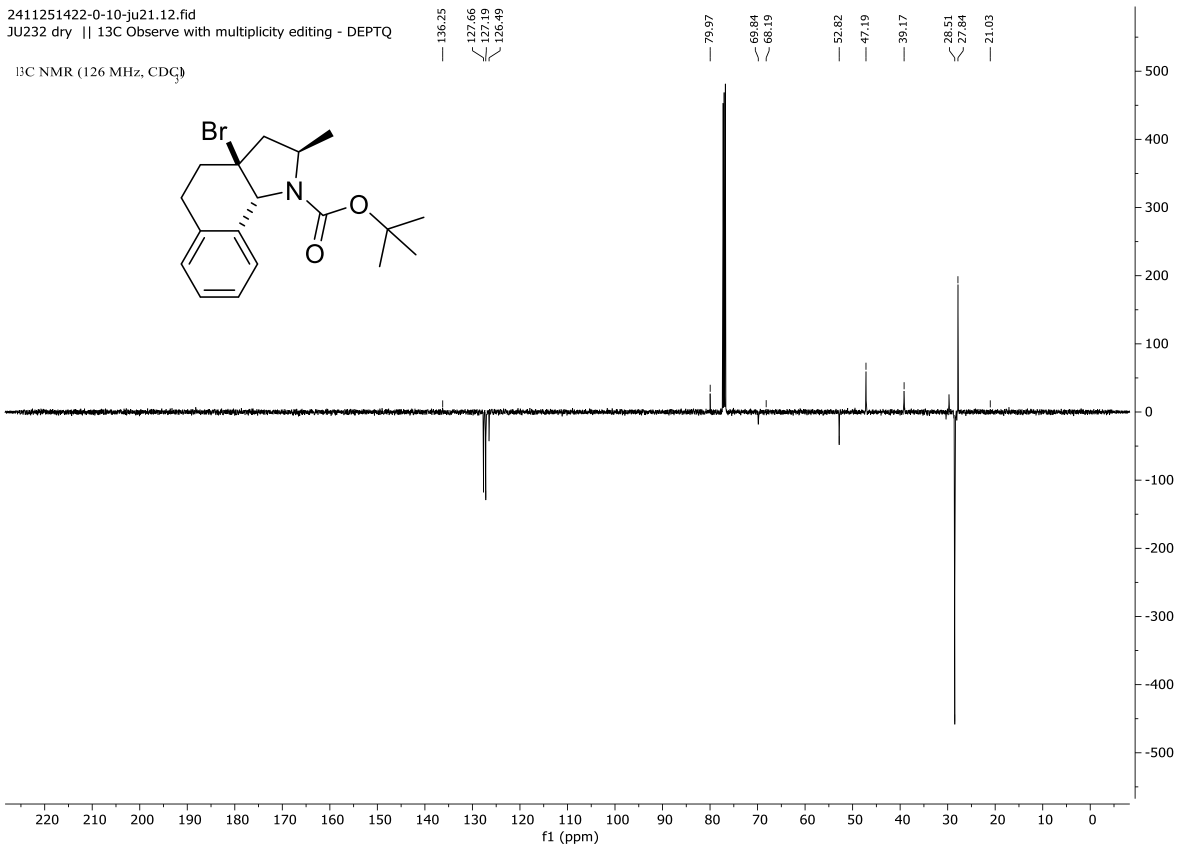

tert-Butyl (2-(3-phenyloxiran-2-yl)ethyl) carbamate (45)

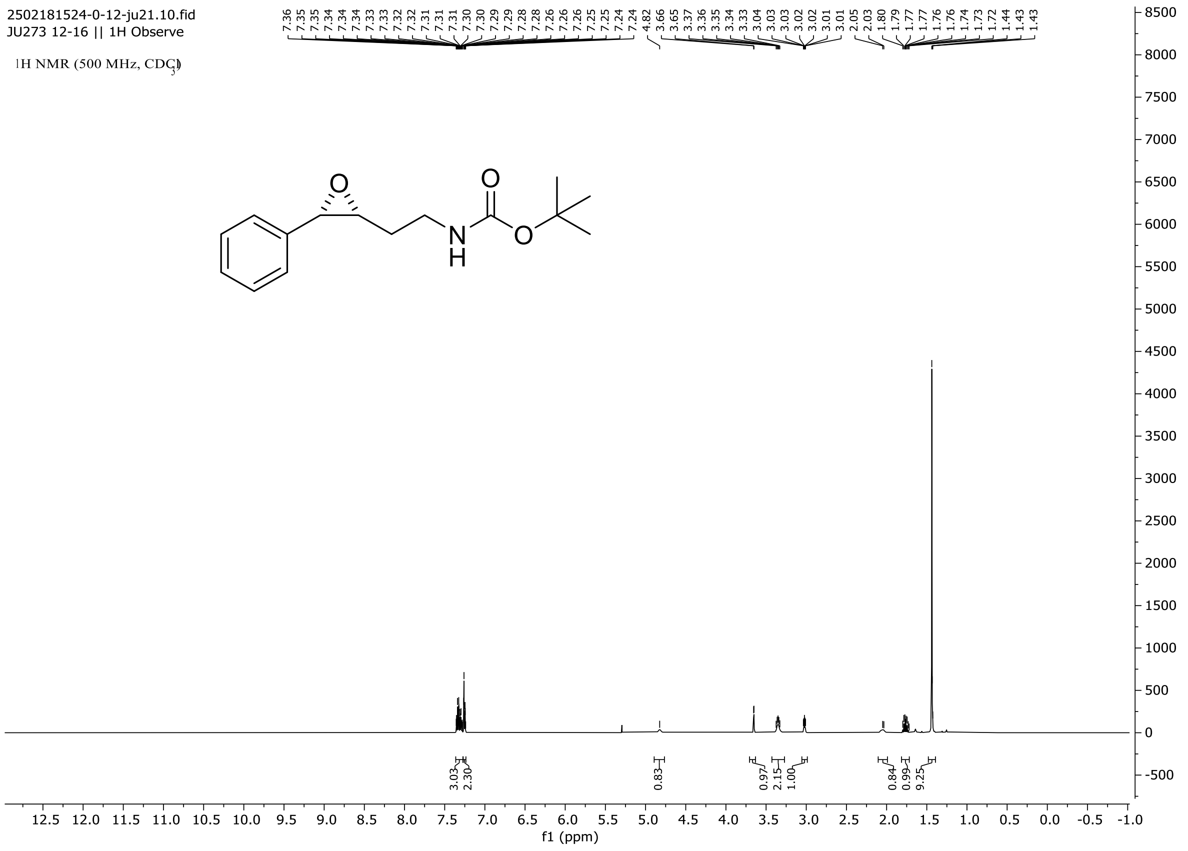

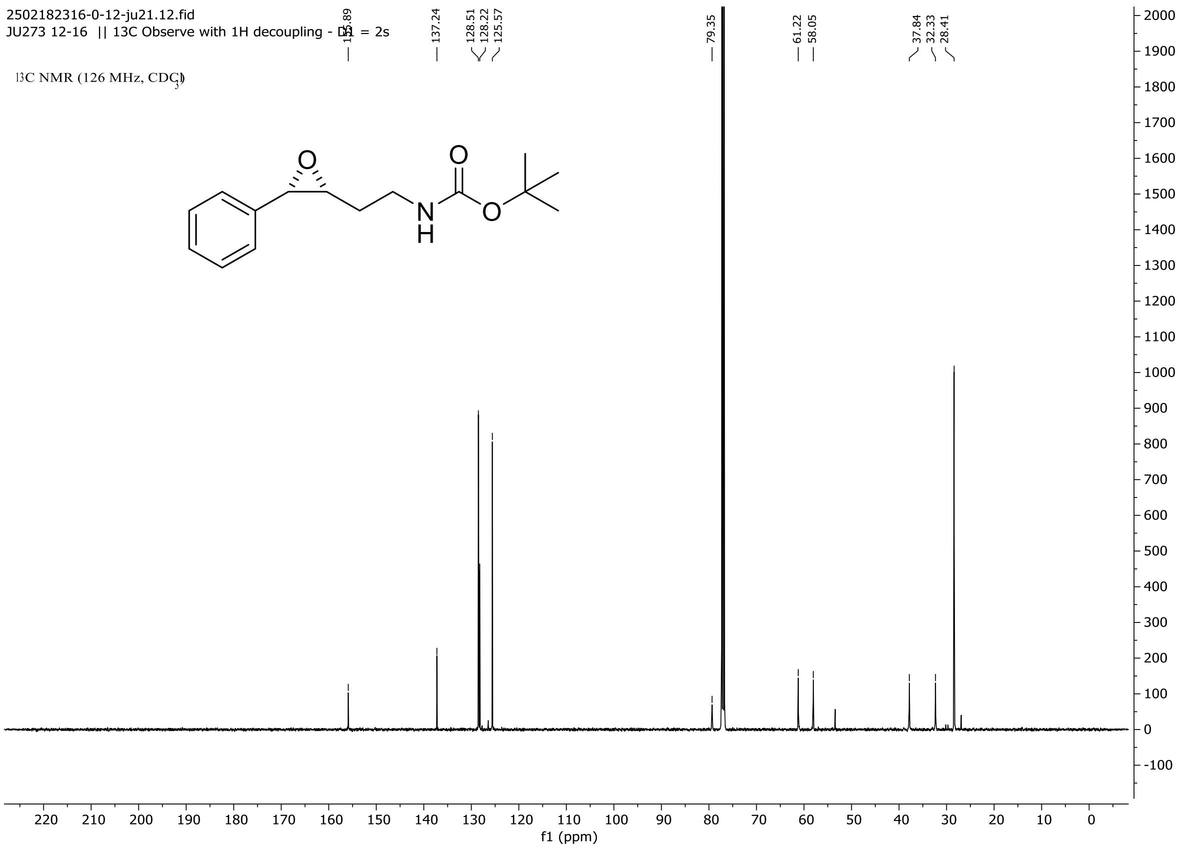

tert-Butyl ((2*R*)-1-(3-phenyloxiran-2-yl)propan-2-yl) carbamate (46)

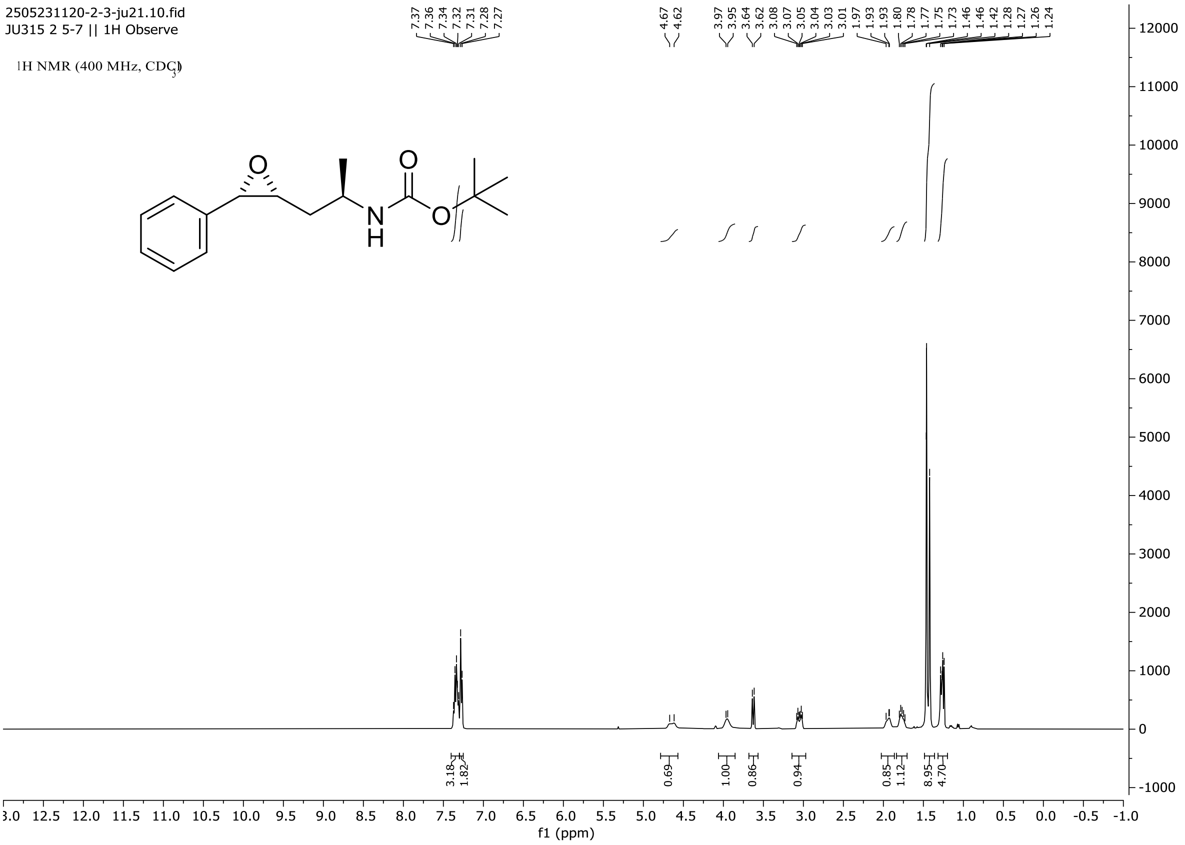

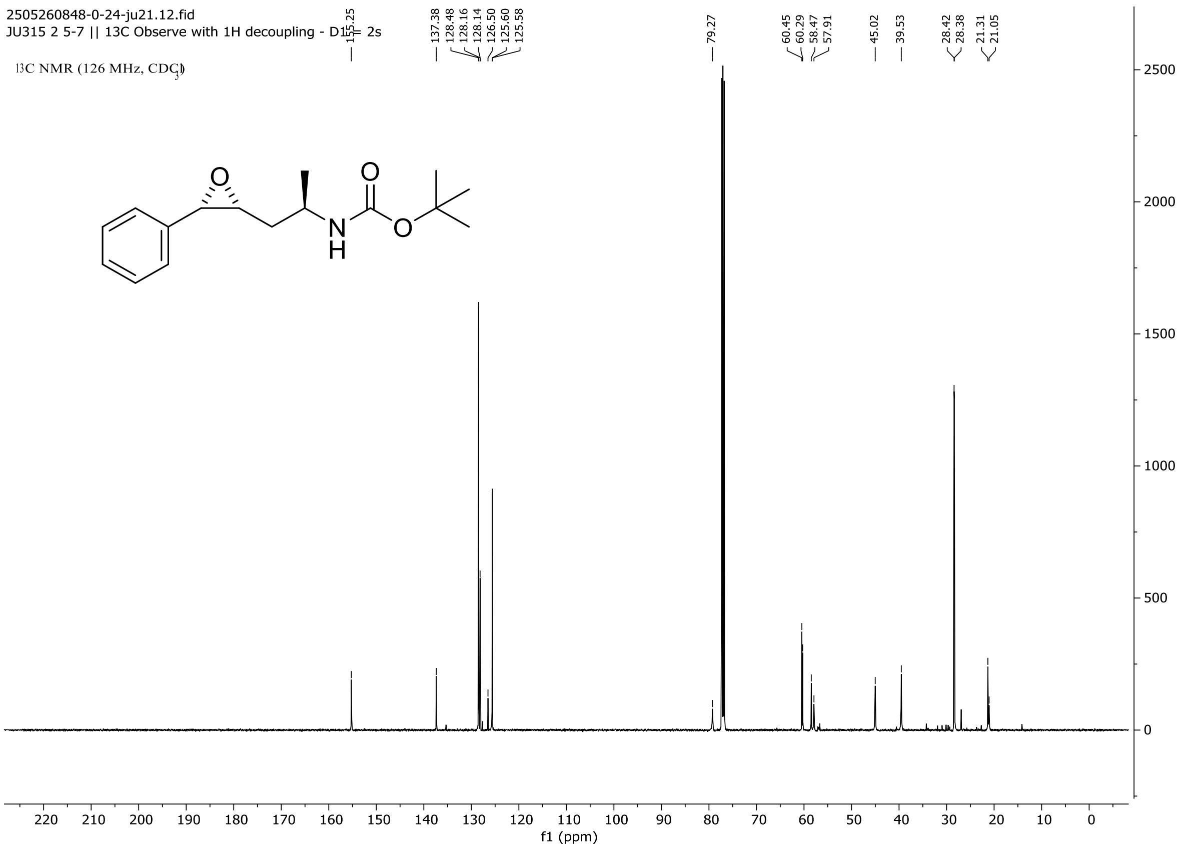

tert-Butyl (2-(3-phenyloxiran-2-yl)-1-(*p*-tolyl)ethyl) carbamate (47)

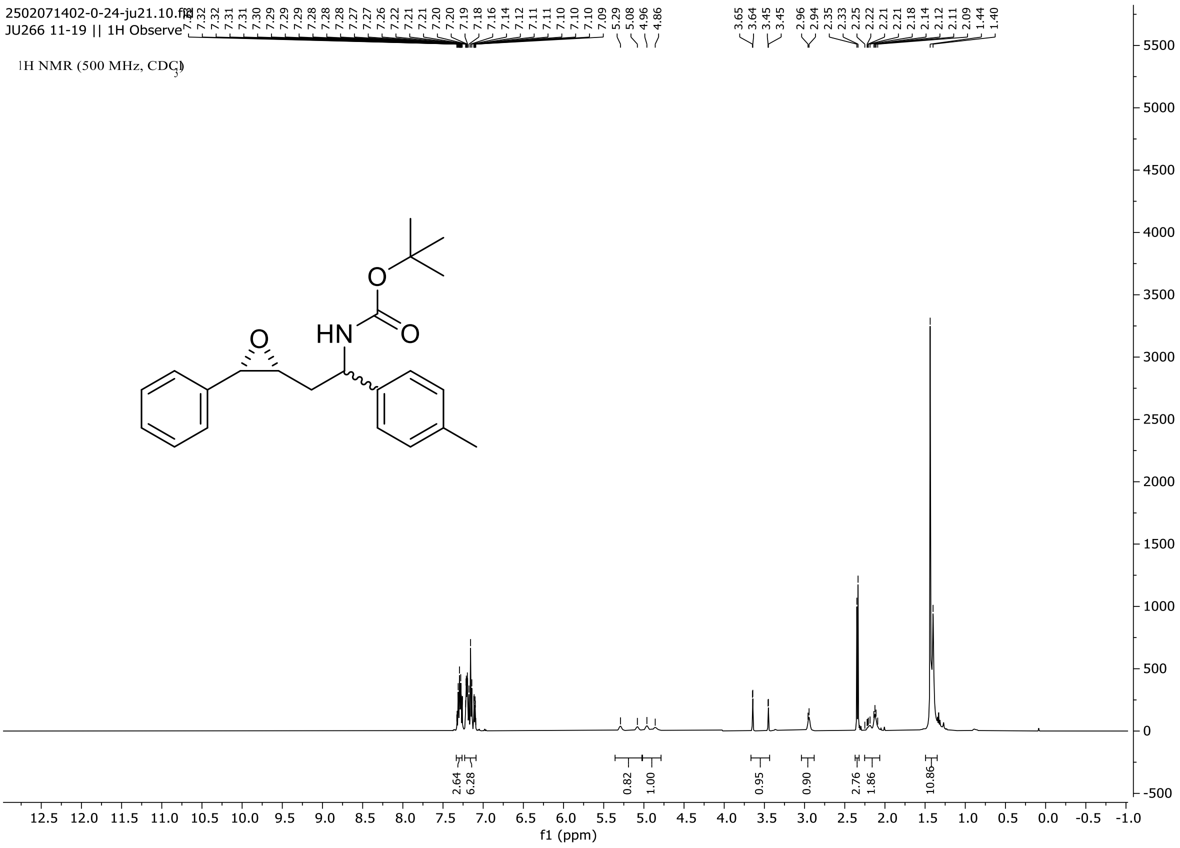

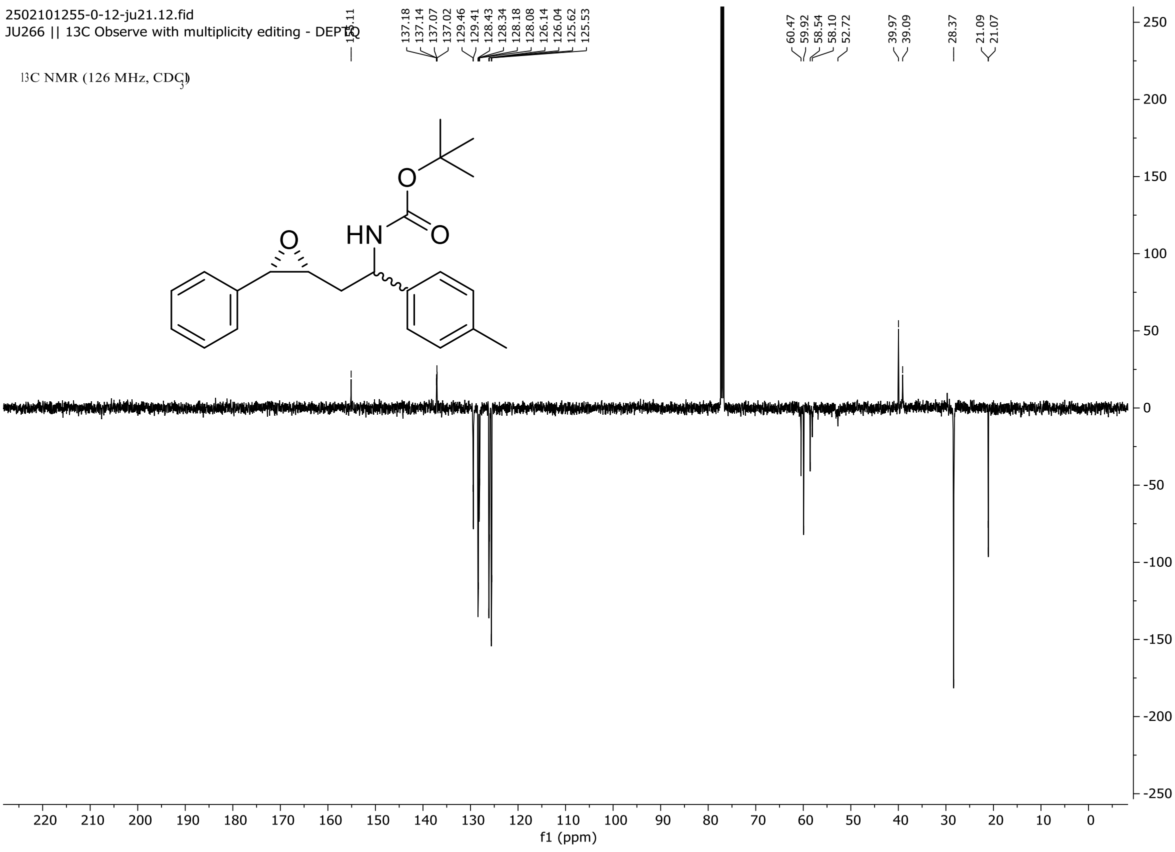

tert-Butyl (2-(3-(4-chlorophenyl)oxiran-2-yl)-1-(*p*-tolyl)ethyl)carbamate (48)

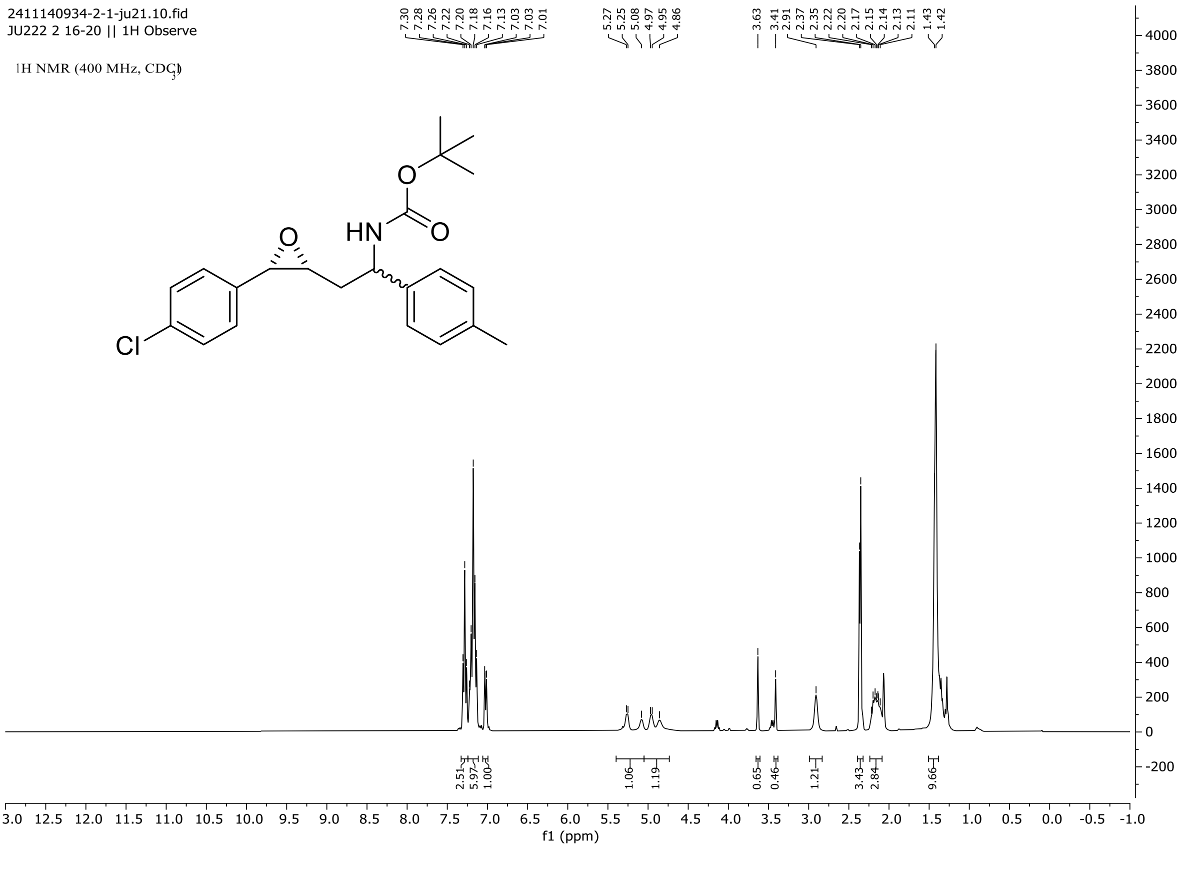

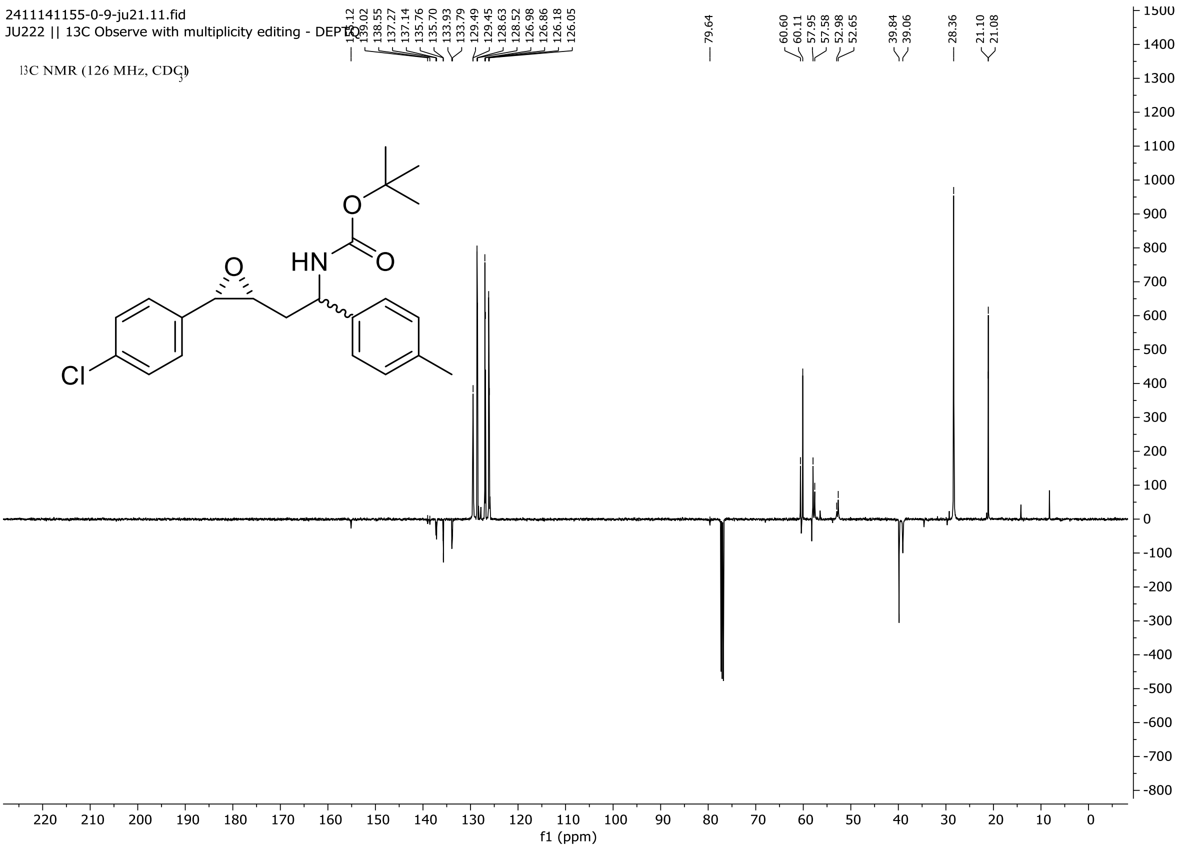

tert-Butyl (2-(3-phenyloxiran-2-yl)cyclopentyl) carbamate (49)

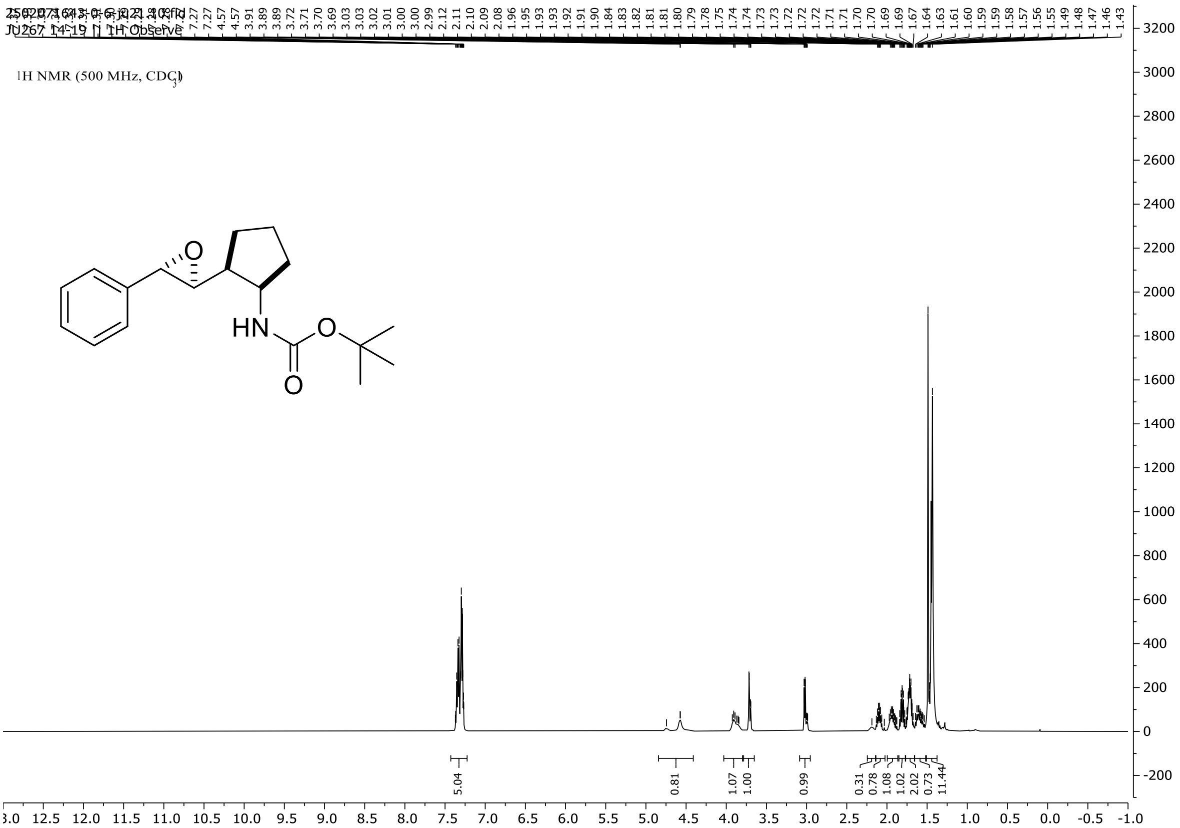

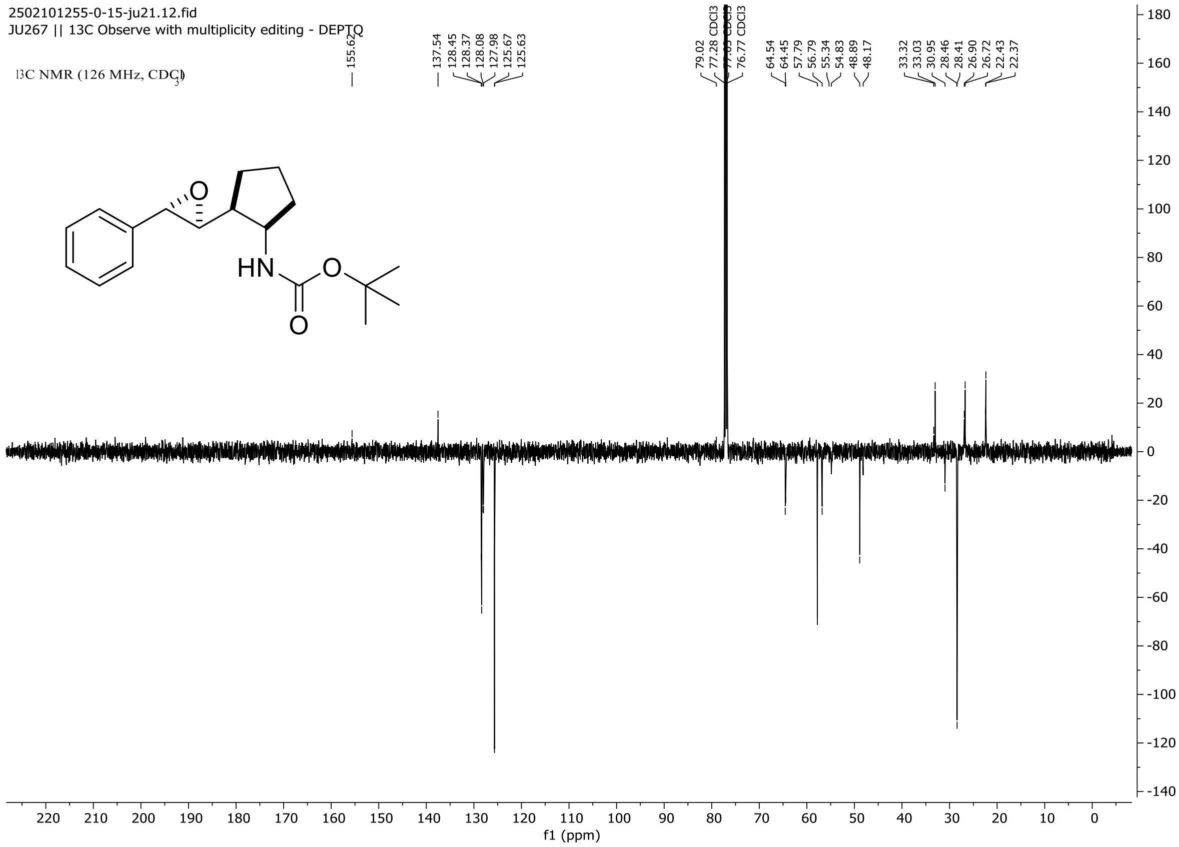

tert-Butyl ((3-(4-chlorophenyl)oxiran-2-yl)methyl)carbamate (50)

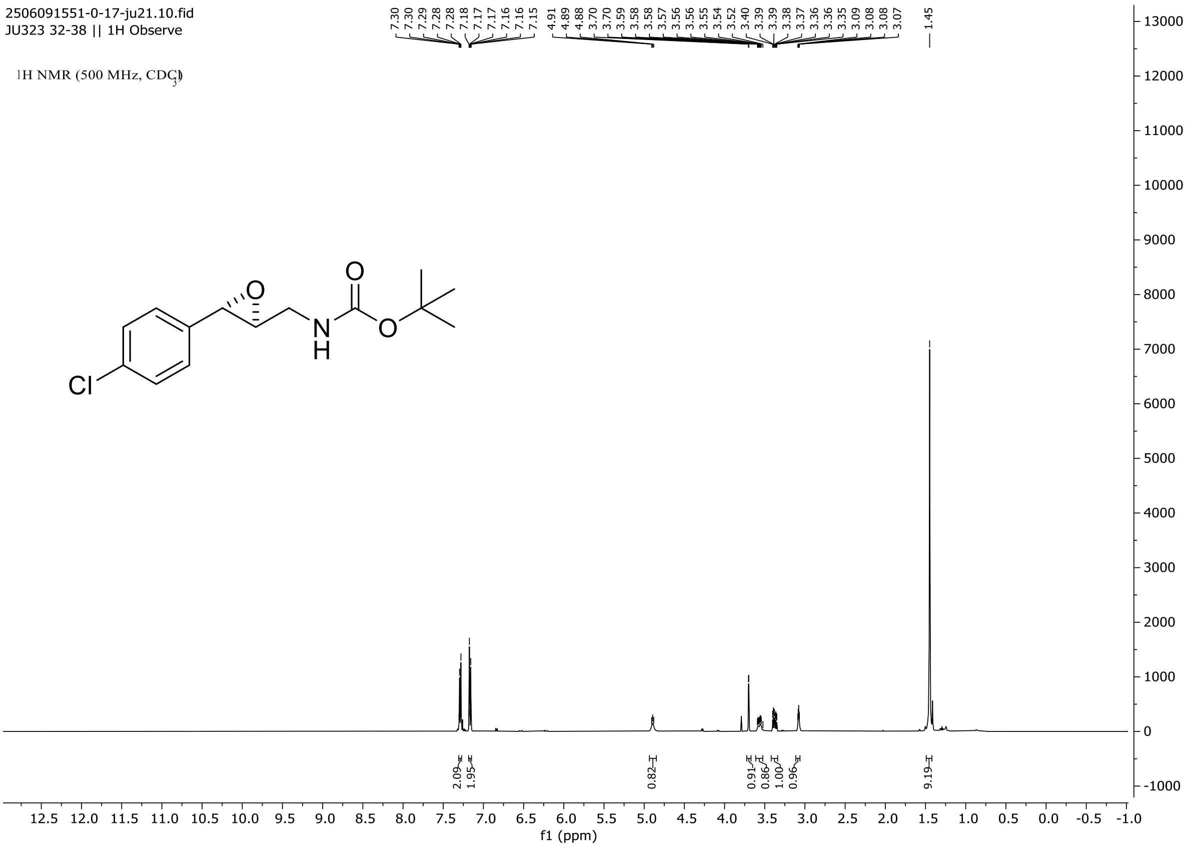

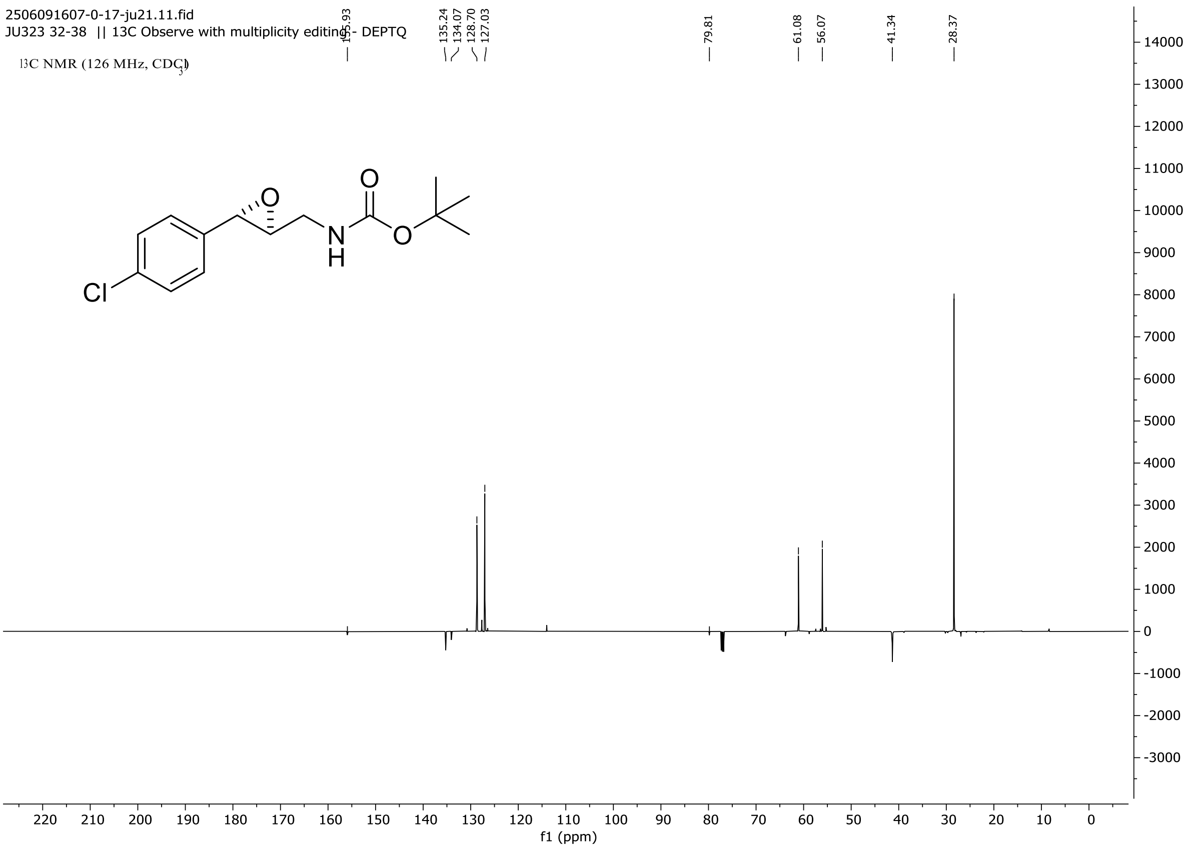

tert-Butyl ((3-phenyloxiran-2-yl)methyl)carbamate (51)

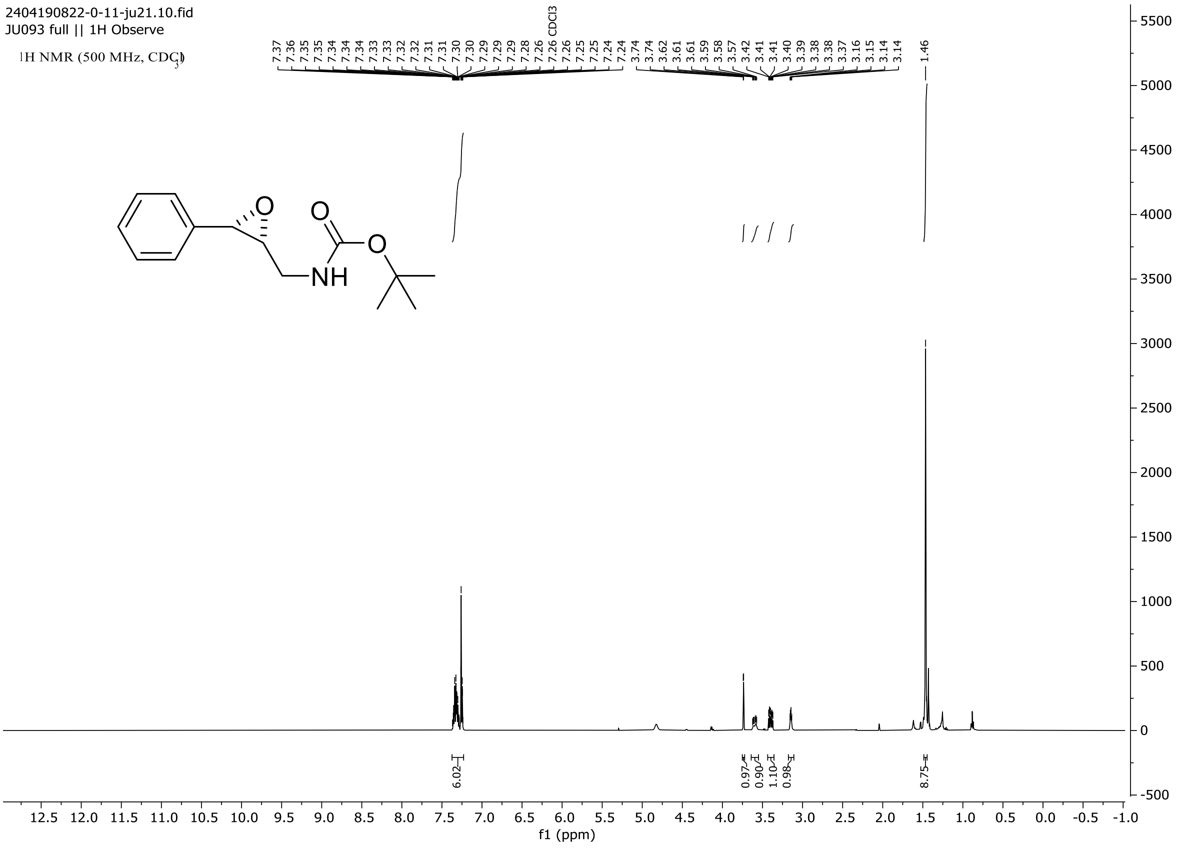

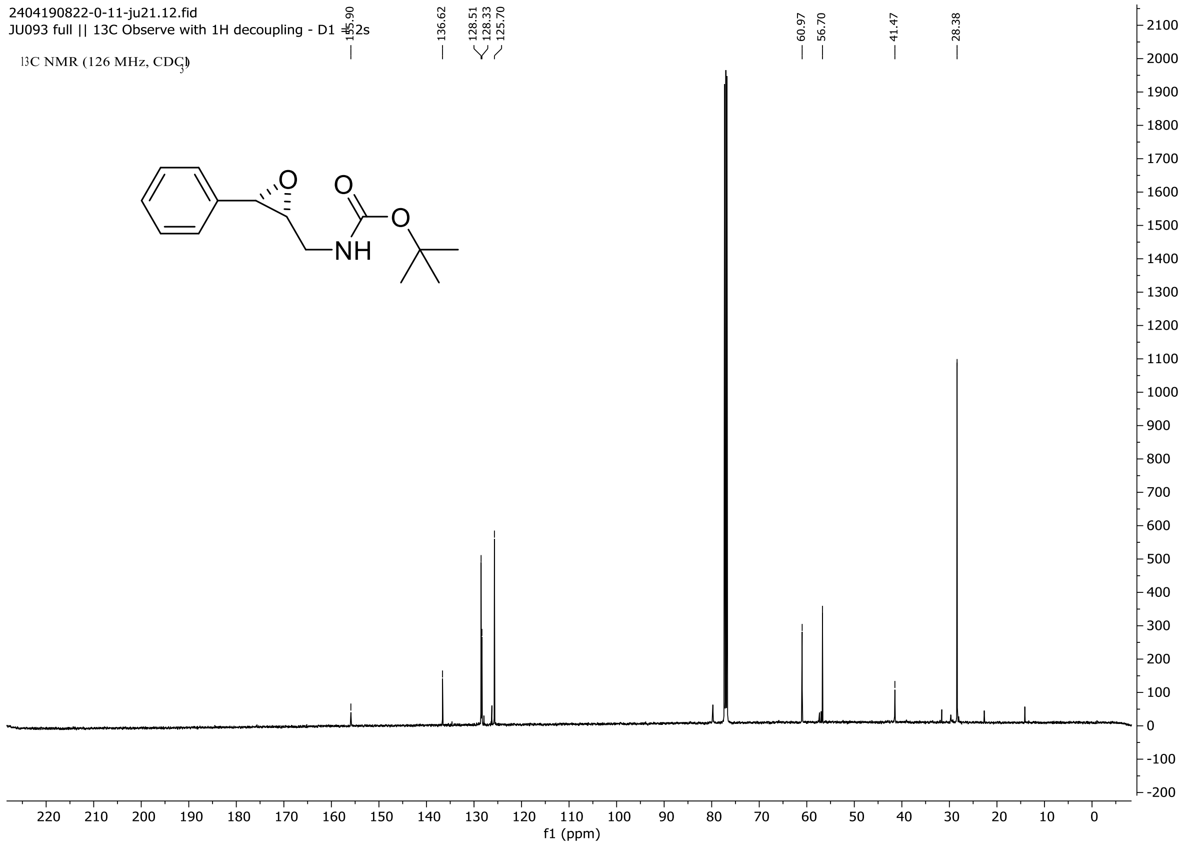

tert-Butyl (3-phenyloxirane-2-carbonyl)carbamate (52)

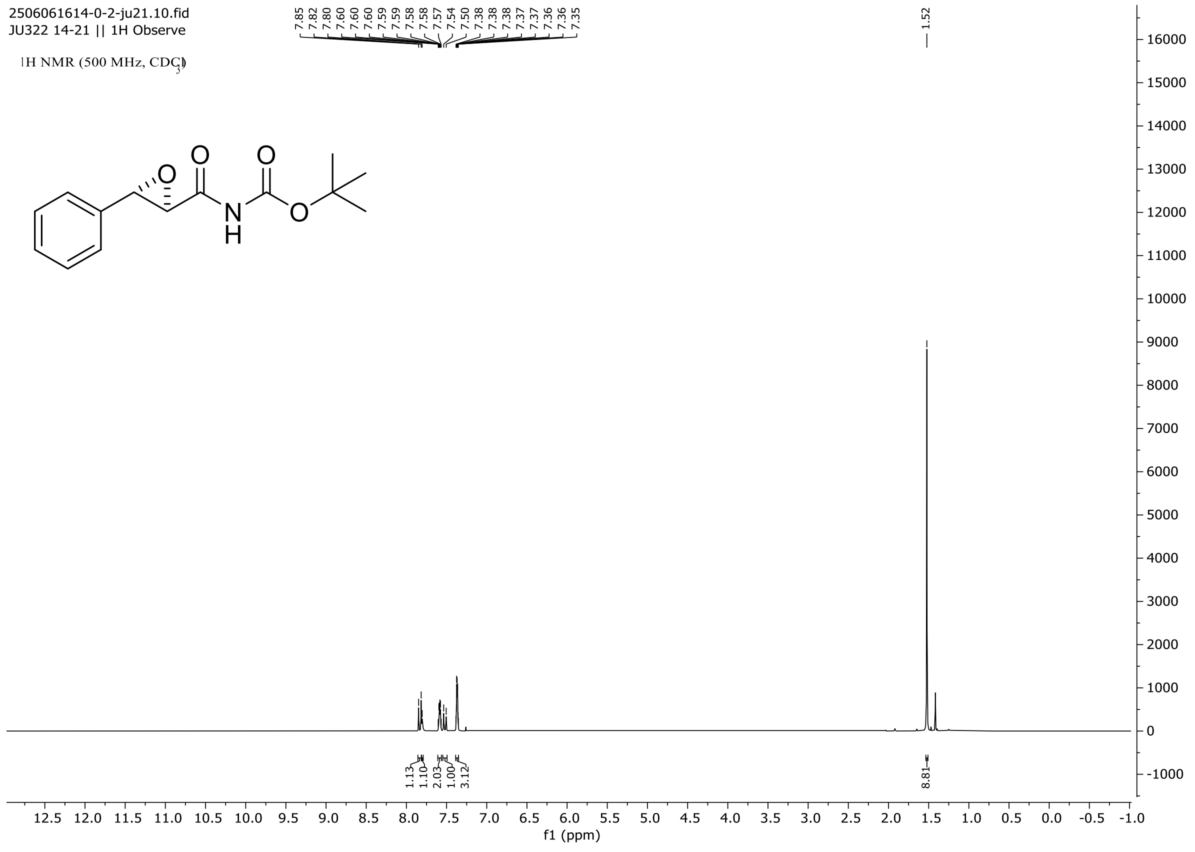

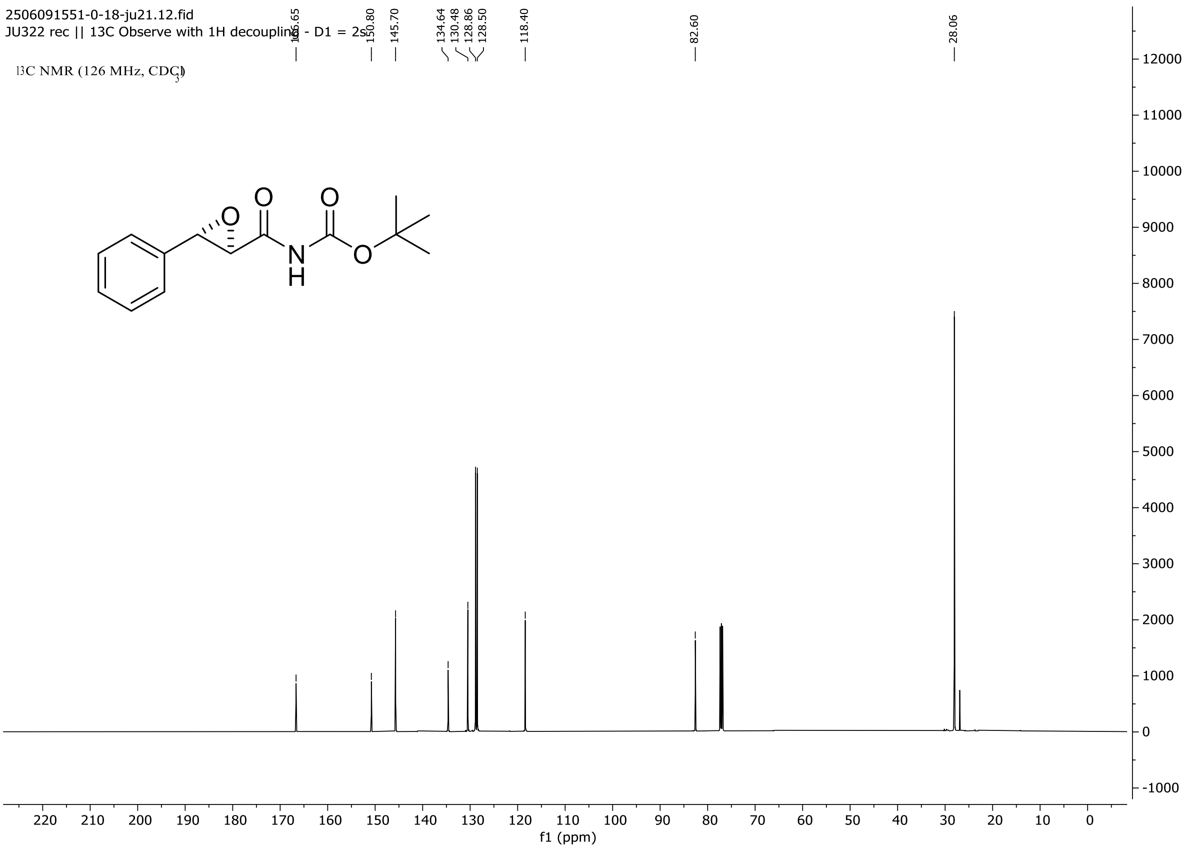

5-Hydroxy-6-phenyl-1,3-oxazinan-2-one (53)

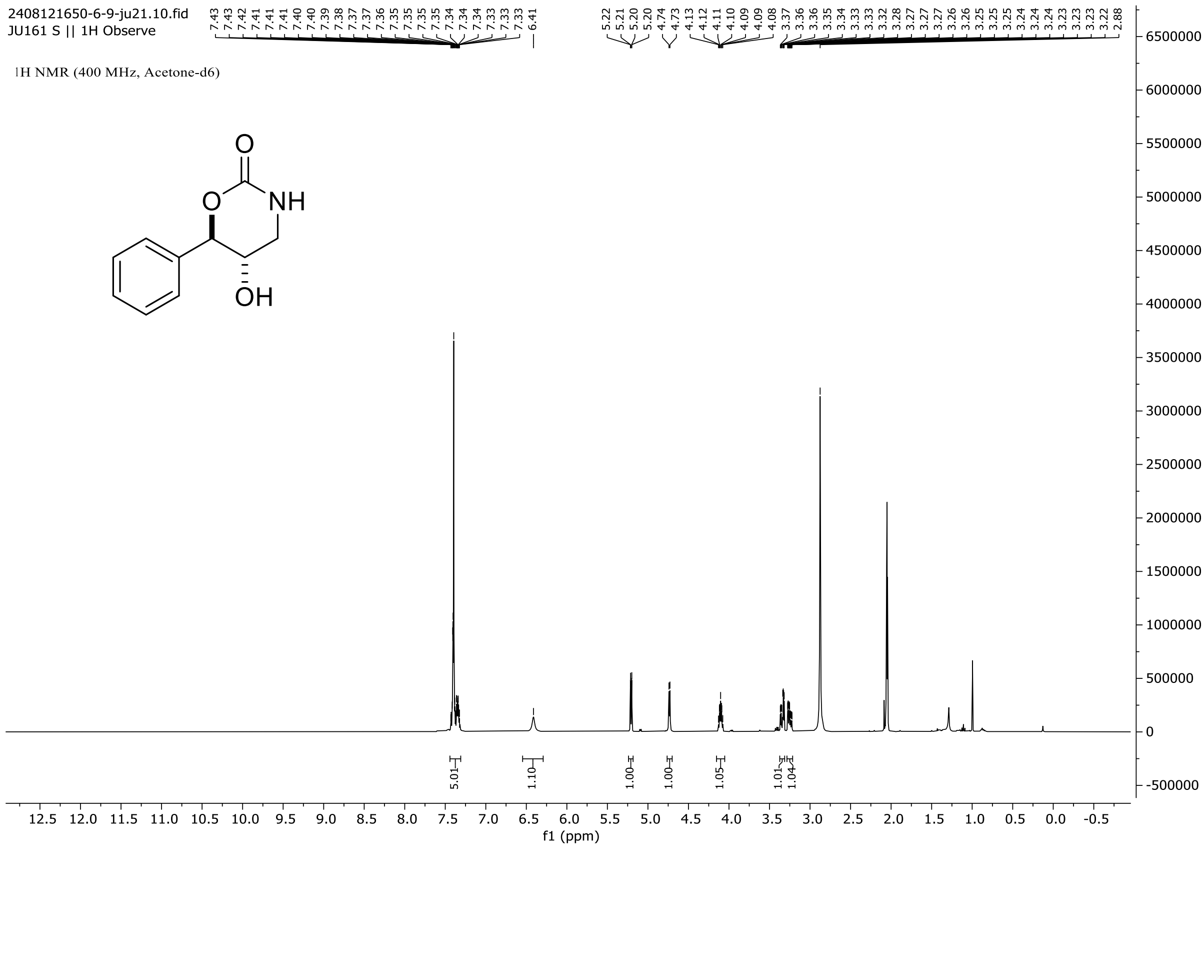

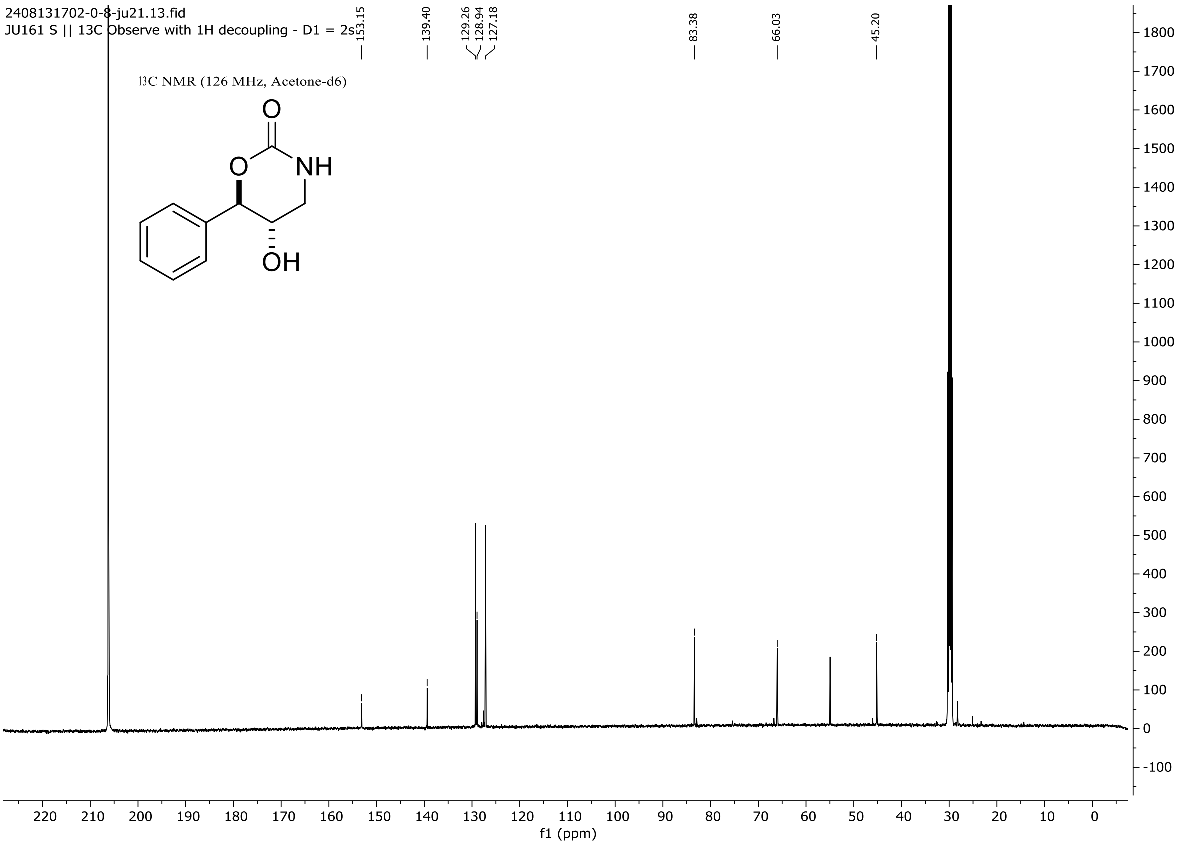

6-(4-Chlorophenyl)-5-hydroxy-1,3-oxazinan-2,4-dione (54)

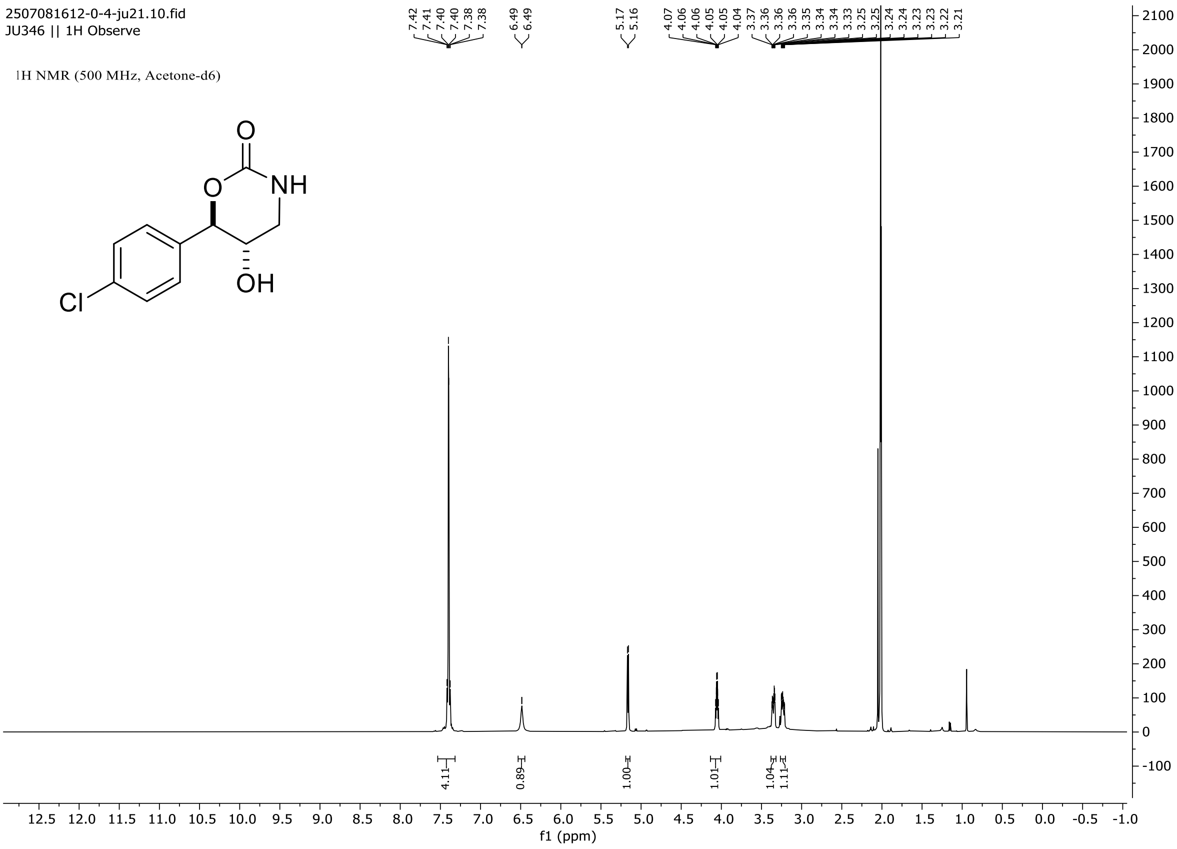

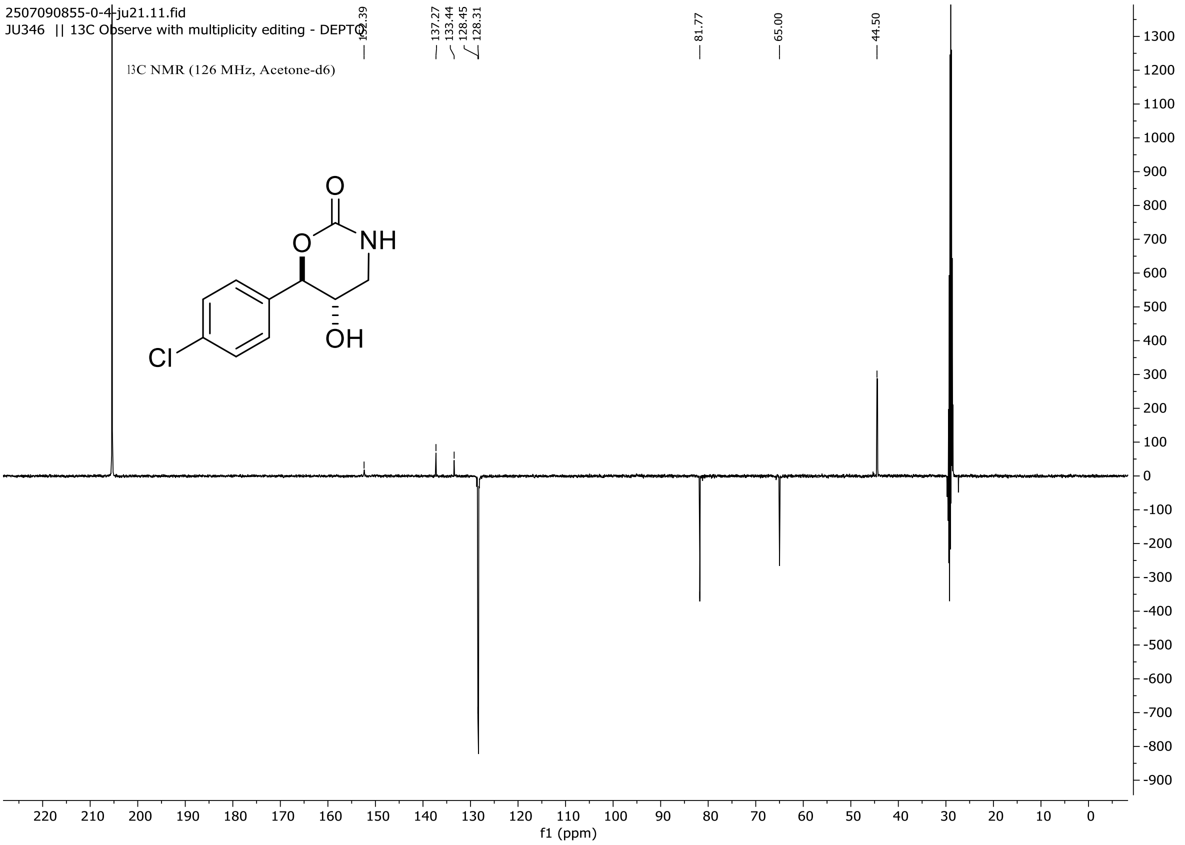

5-Hydroxy-6-phenyl-1,3-oxazinane-2,4-dione (55)

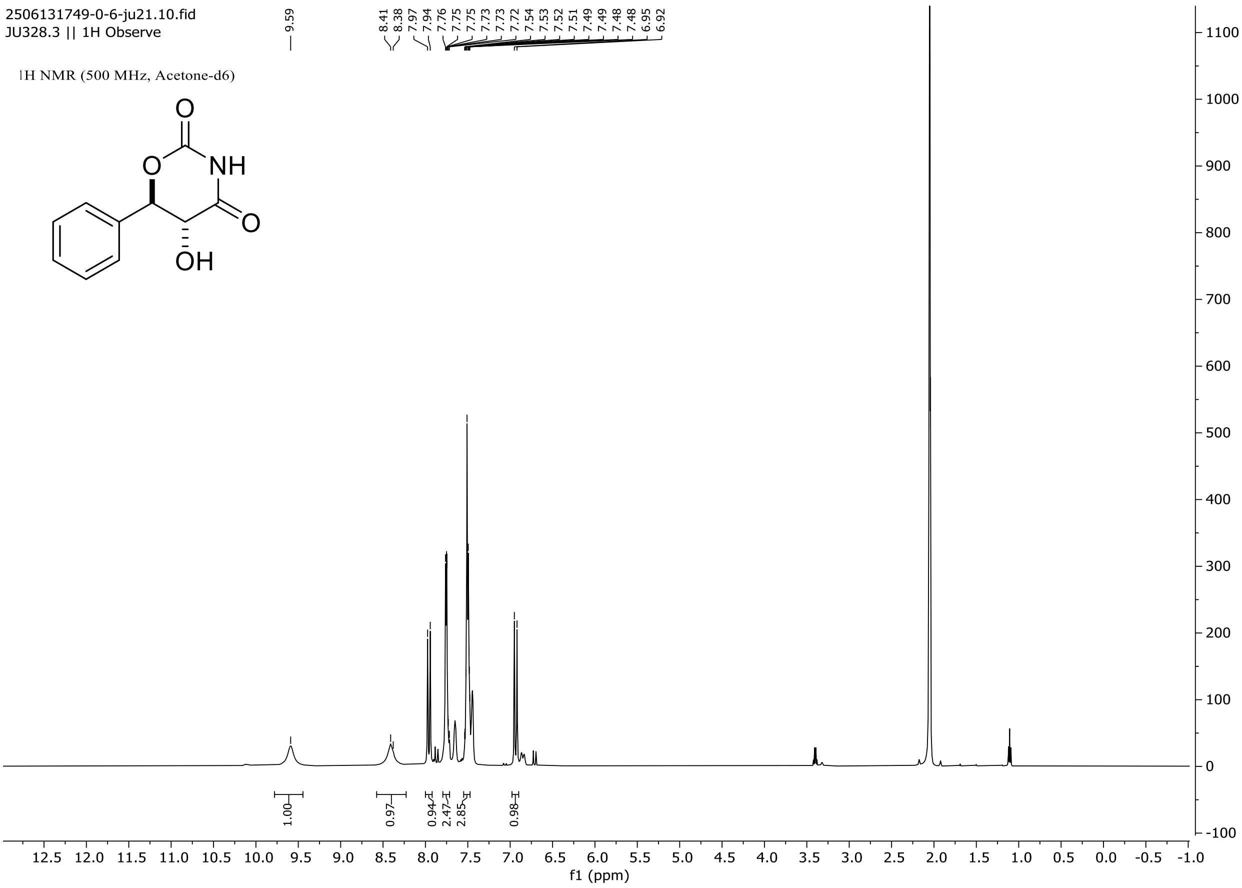

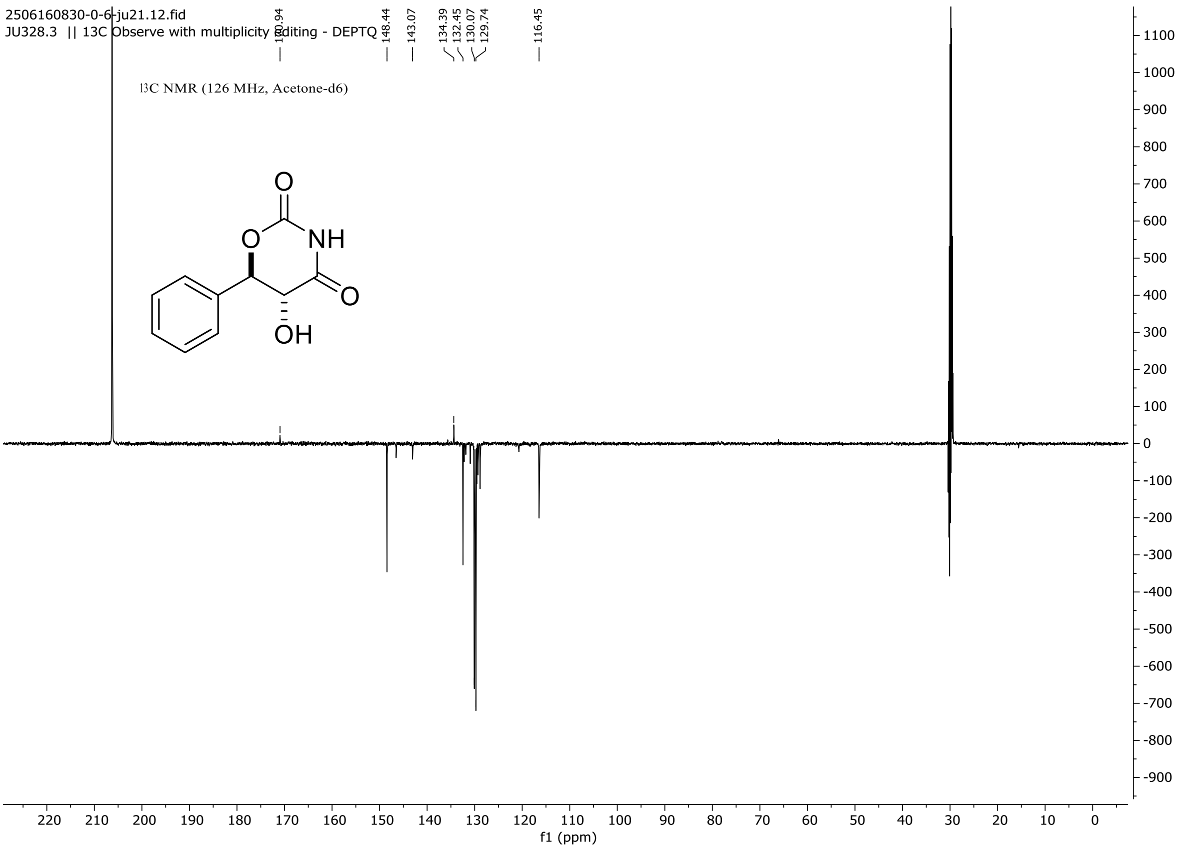

tert-Butyl 3-hydroxy-2-phenyl-5-(*p*-tolyl)pyrrolidine-1-carboxylate (56)

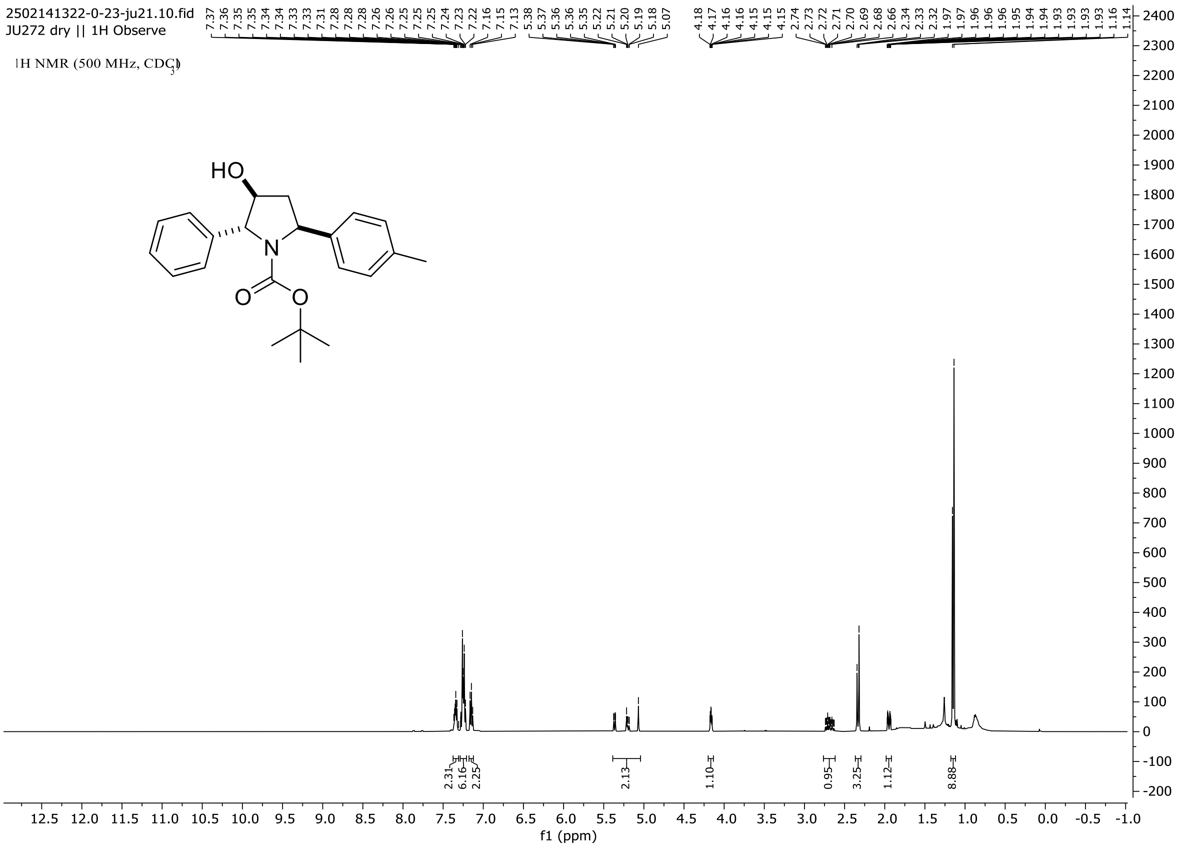

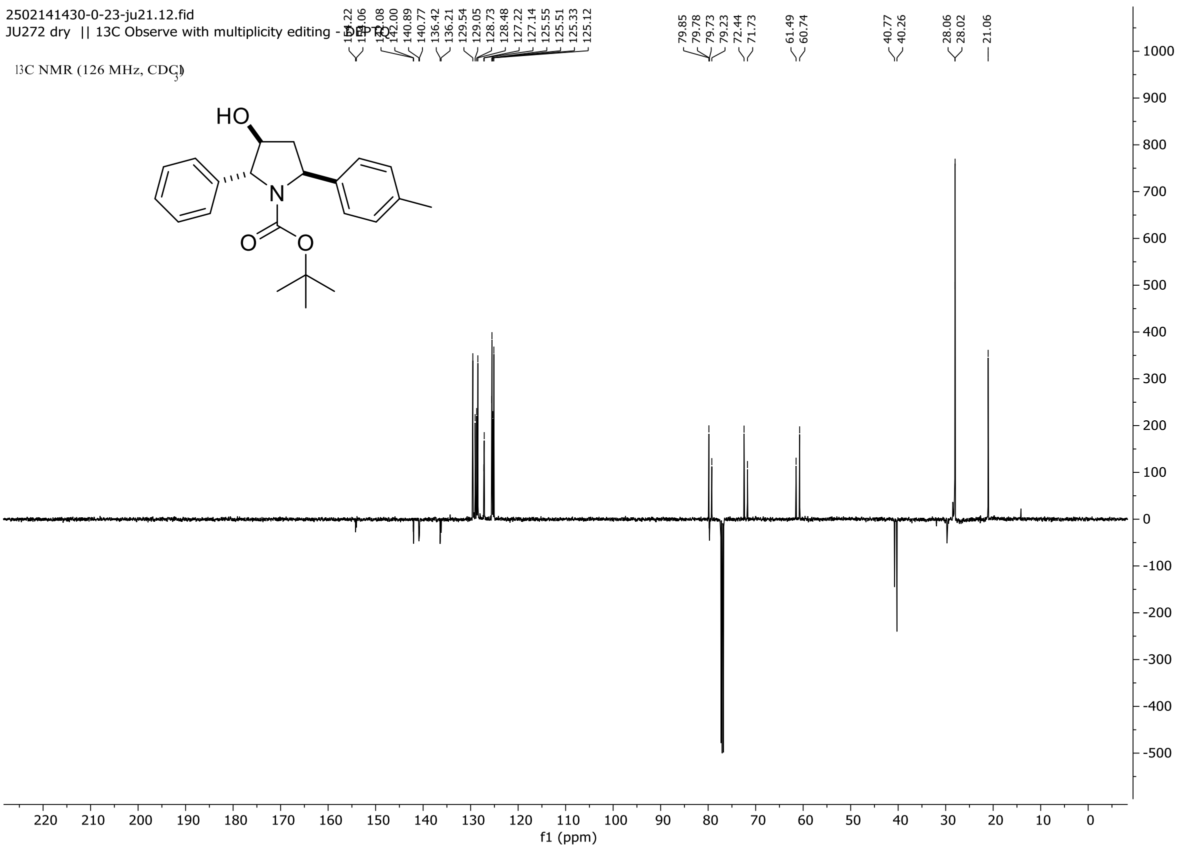

tert-Butyl 3-hydroxy-2-phenylpyrrolidine-1-carboxylate (57)

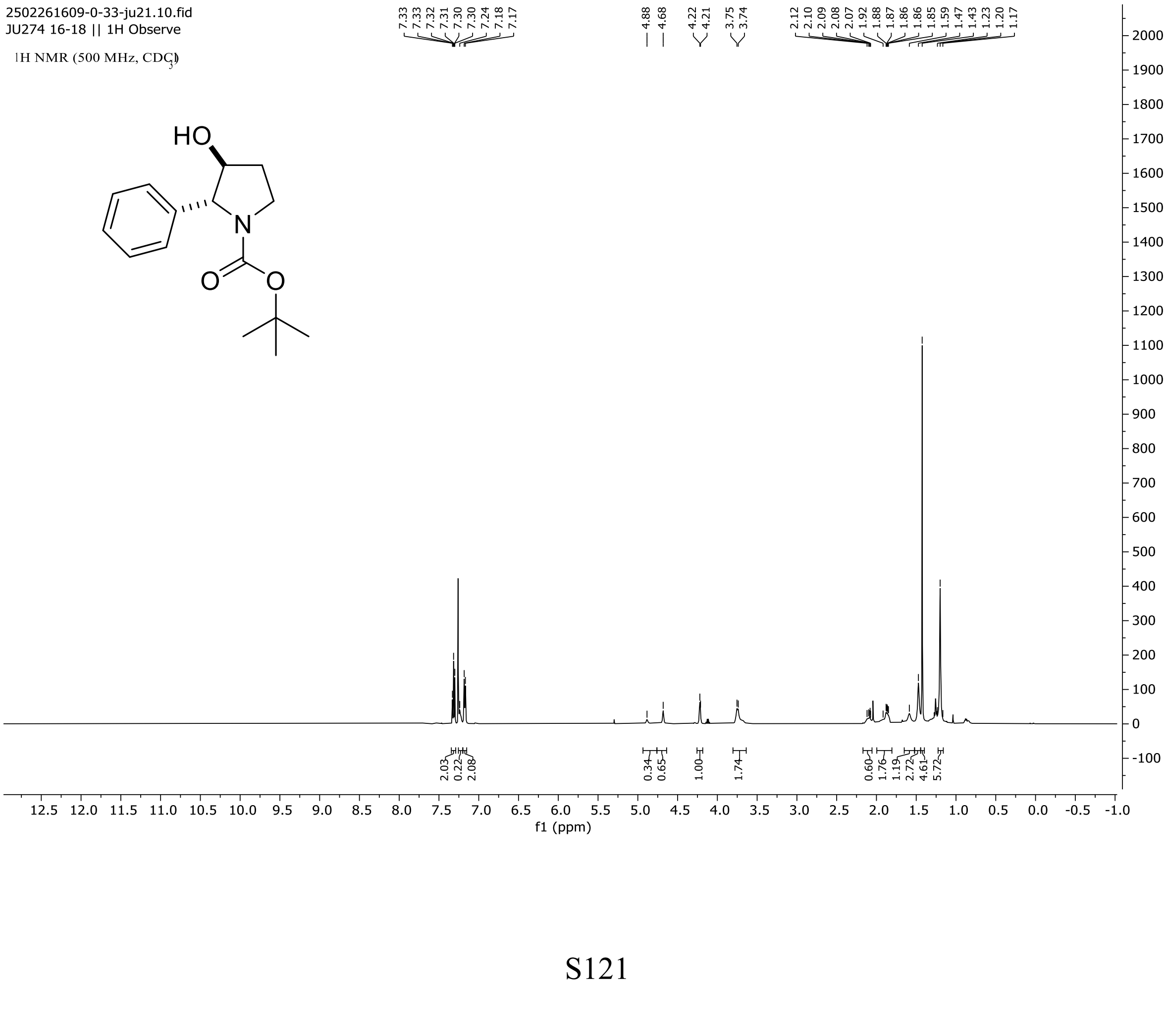

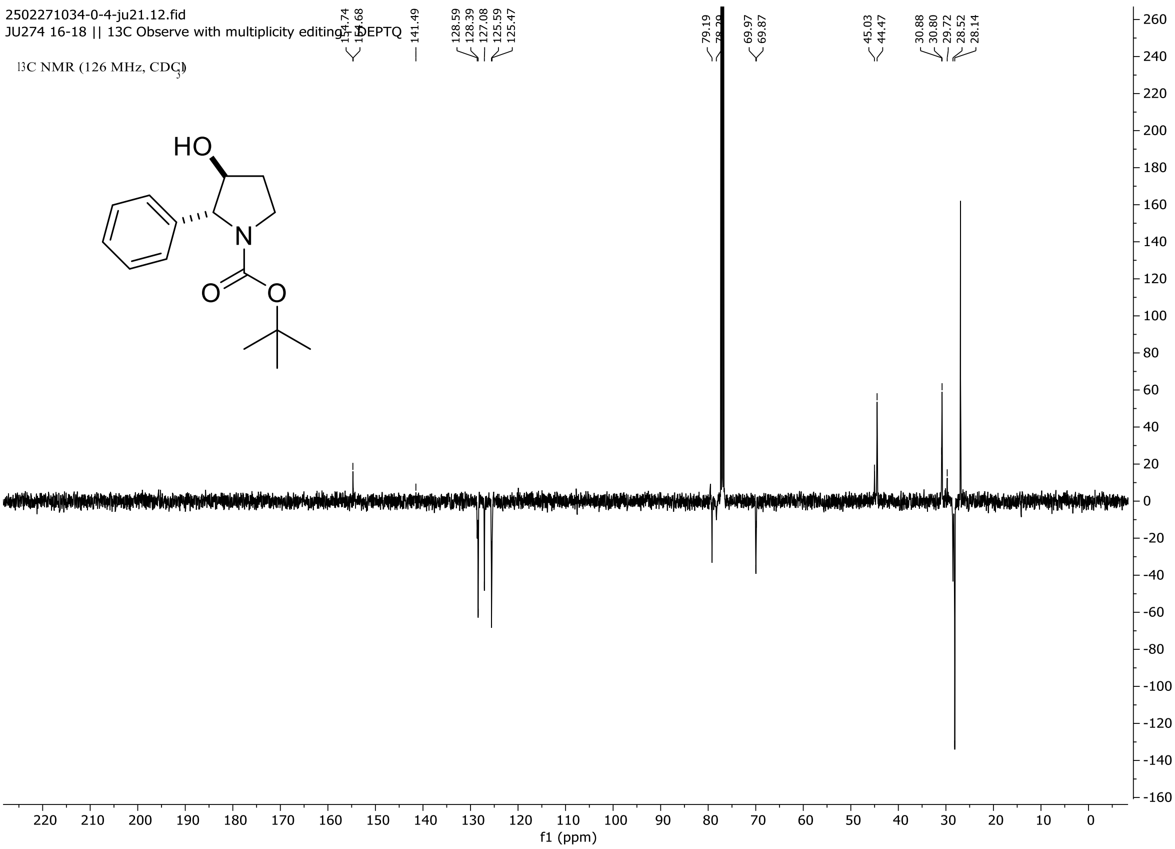

tert-Butyl 3-hydroxy-2-phenylhexahydrocyclopenta[*b*]pyrrole-1(2*H*)-carboxylate (58)

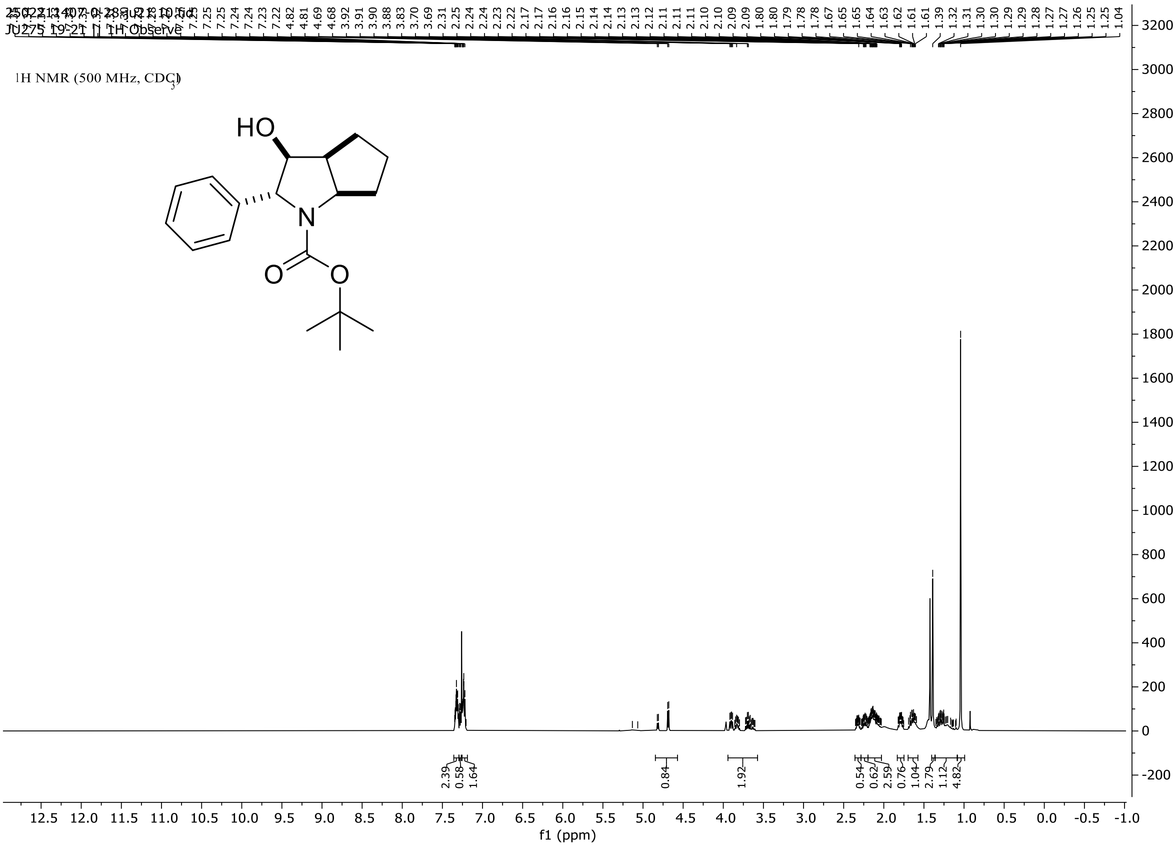

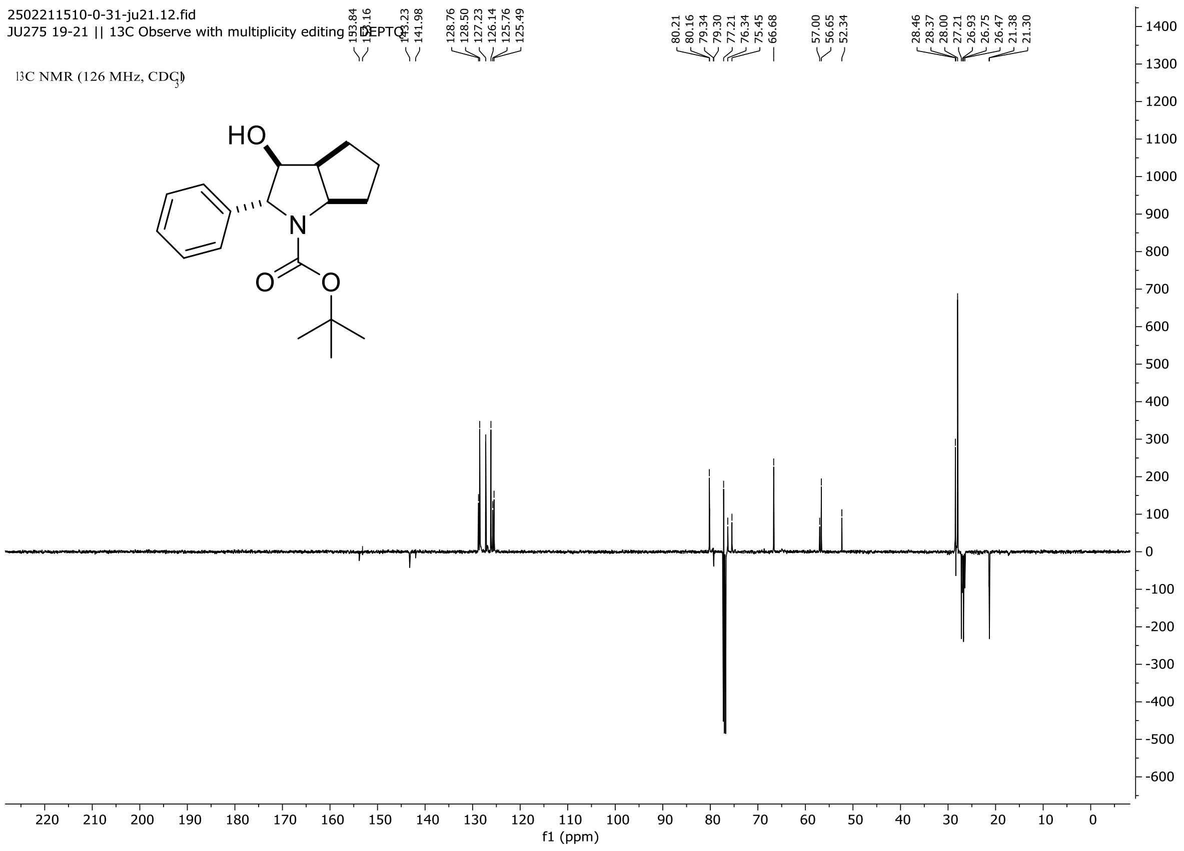
2502211510-0-31-ju21.12.fid
JU275 19-21 || 13C Observe with multiplicity editing - DEPTQ
13C NMR (126 MHz, CDCl3)
153.84
153.16
143.23
141.98
128.76
128.50
127.23
126.14
125.76
125.49
80.21
80.16
79.34
79.30
77.21
76.34
75.45
66.68
57.00
56.65
52.34
28.46
28.37
28.00
27.21
26.93
26.75
26.47
21.38
21.30
HO
N
O
O
f1 (ppm)

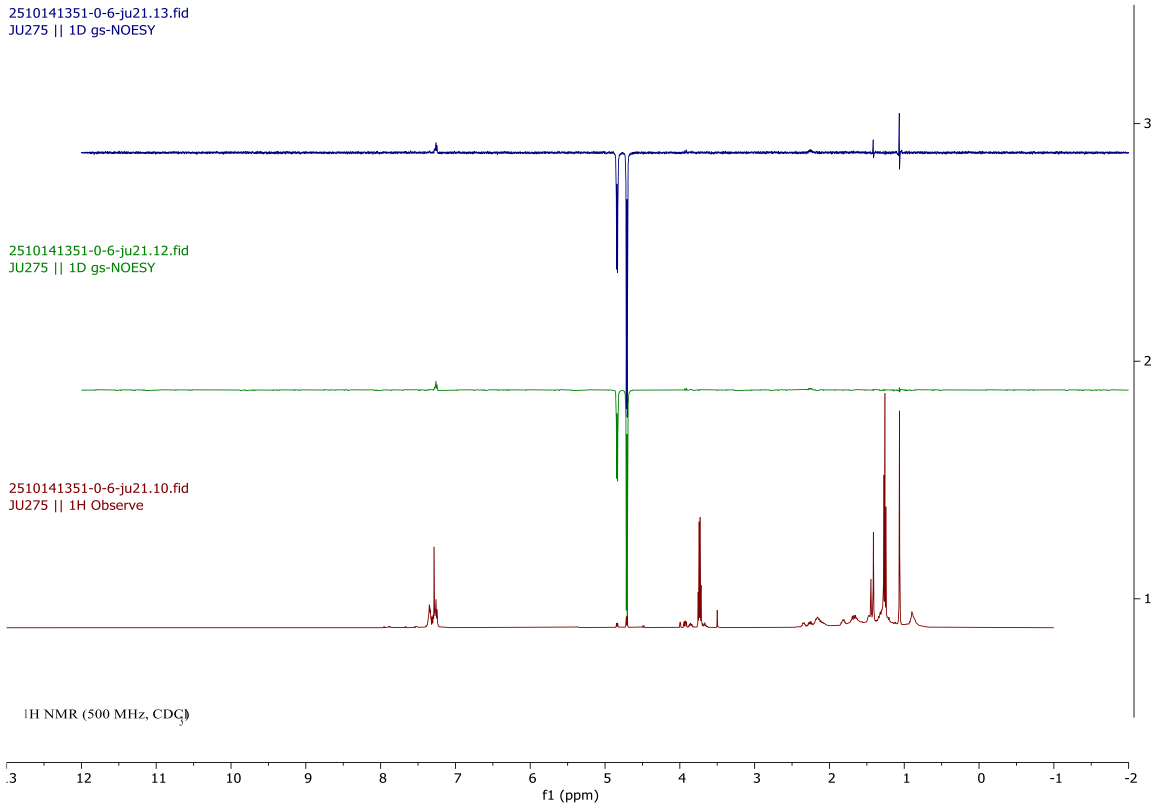
2510141351-0-6-ju21.13.fid
JU275 || 1D gs-NOESY
2510141351-0-6-ju21.12.fid
JU275 || 1D gs-NOESY
2510141351-0-6-ju21.10.fid
JU275 || 1H Observe
1H NMR (500 MHz, CDCl3)
f1 (ppm)

***tert*-Butyl (2*R*,3*S*)-2-(4-chlorophenyl)-3-hydroxy-5-(*p*-tolyl)pyrrolidine-1-carboxylate (59)**

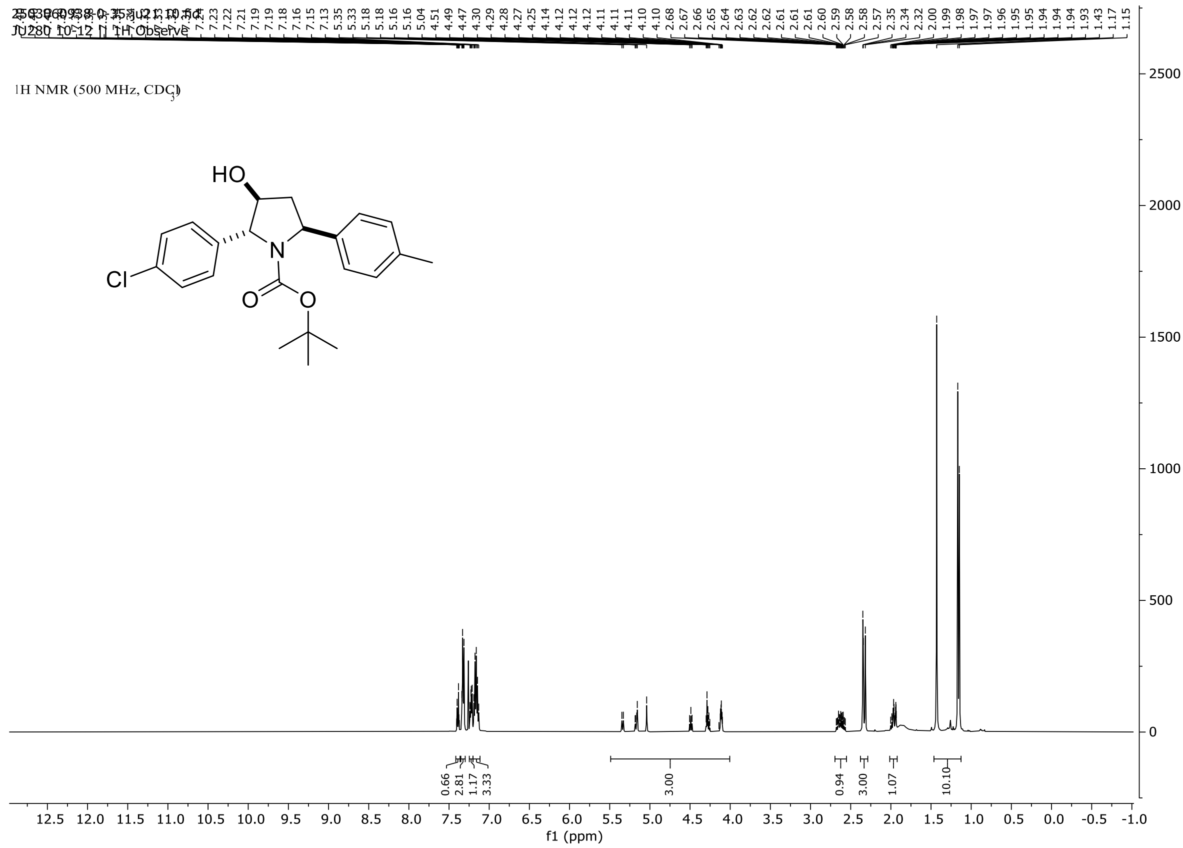

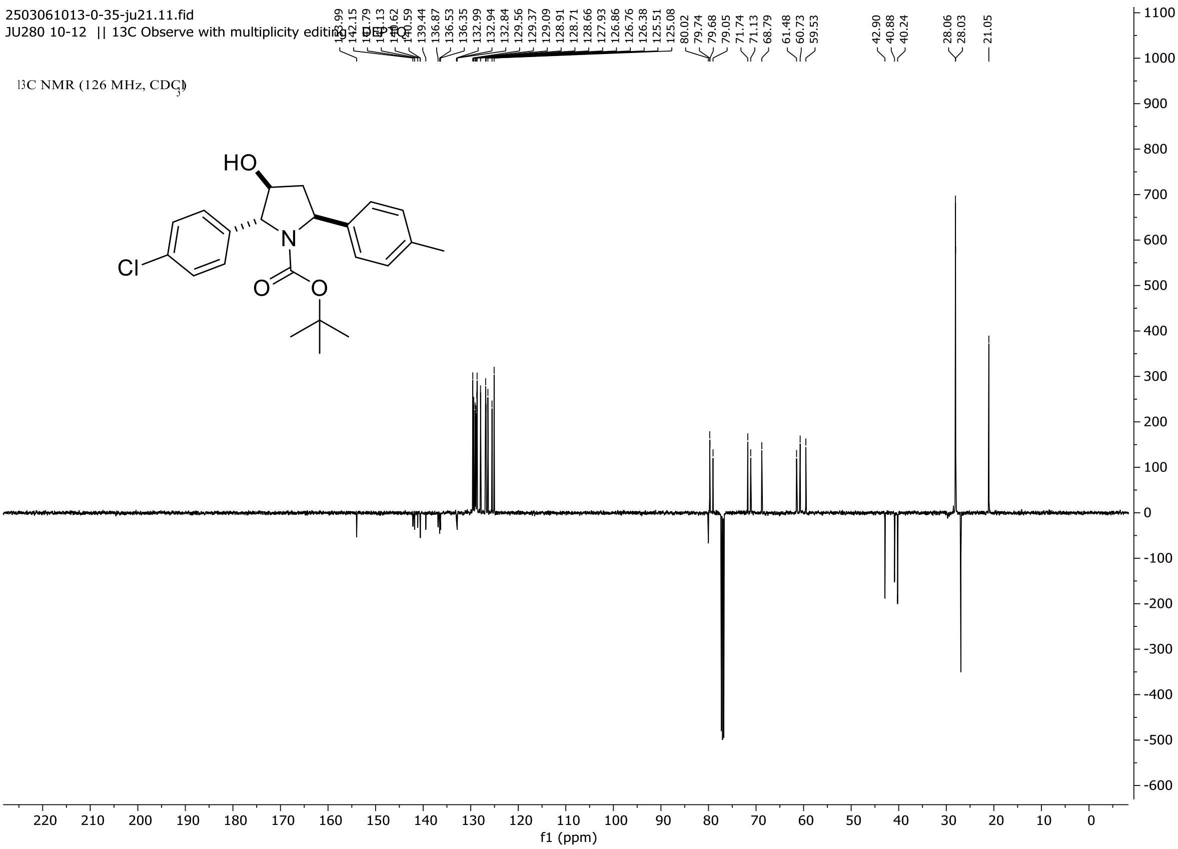

tert-Butyl 3-hydroxy-5-methyl-2-phenylpyrrolidine-1-carboxylate (60)

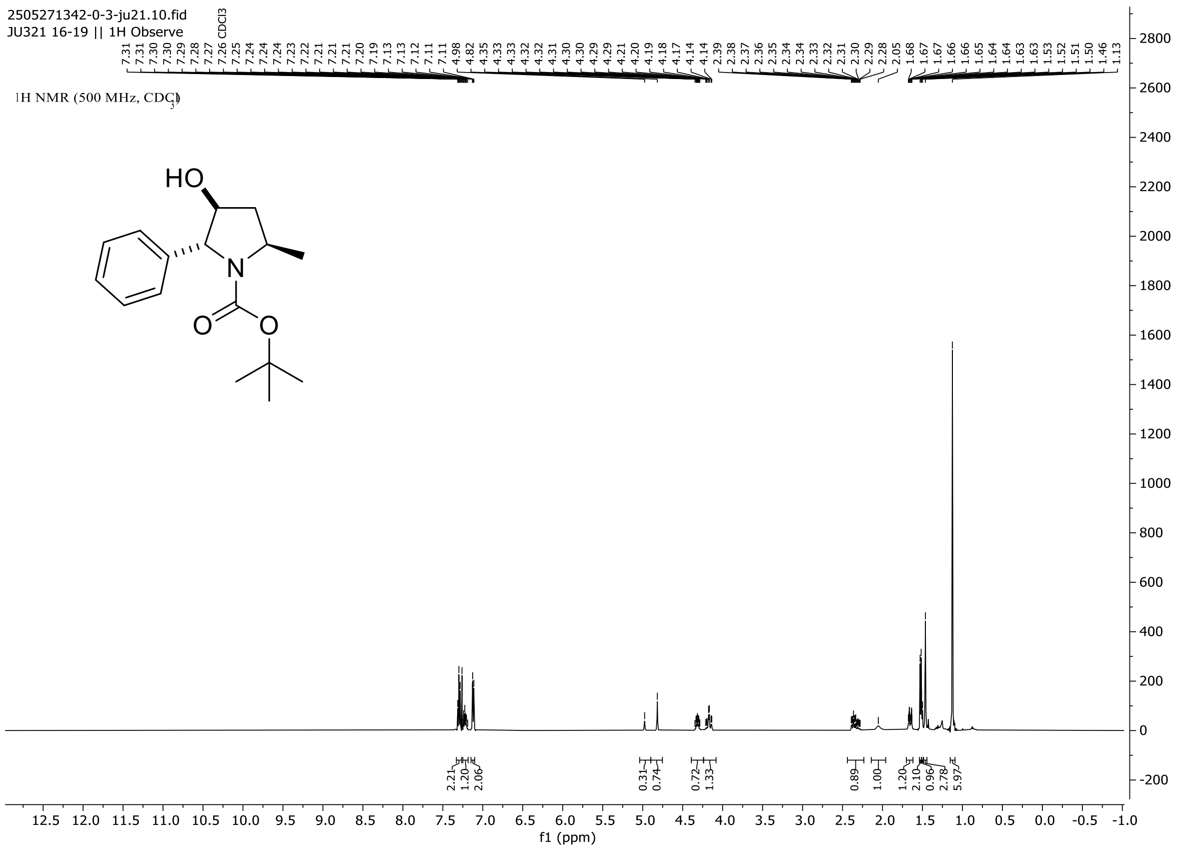

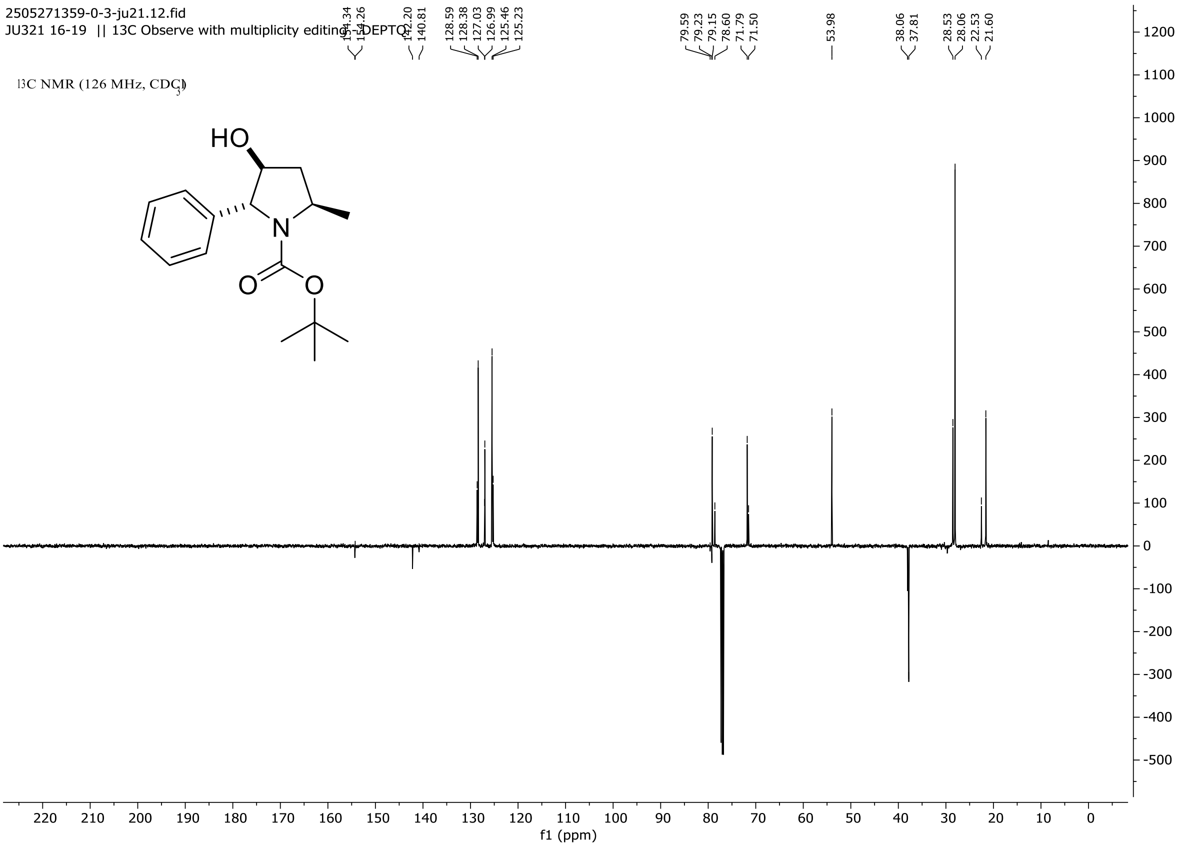

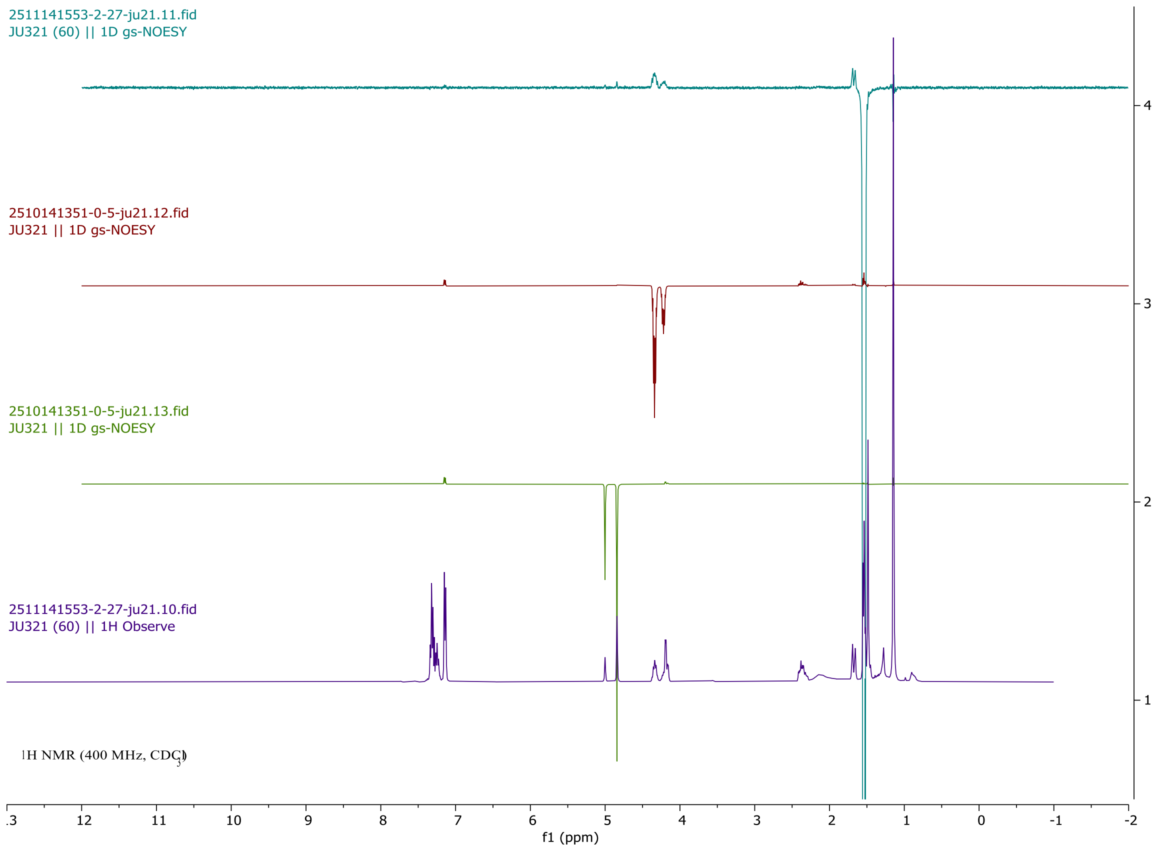
2511141553-2-27-ju21.11.fid
JU321 (60) || 1D gs-NOESY
2510141351-0-5-ju21.12.fid
JU321 || 1D gs-NOESY
2510141351-0-5-ju21.13.fid
JU321 || 1D gs-NOESY
2511141553-2-27-ju21.10.fid
JU321 (60) || 1H Observe
1H NMR (400 MHz, CDCl3)
f1 (ppm)
